# Supplementary material for: Chimeric natural products derived from medermycin and the nature-inspired construction of their polycyclic skeletons
Source: Nat Commun. 2022 Sep 2;13:5169. doi: 10.1038/s41467-022-32901-0 (PMC9440243; doi:10.1038/s41467-022-32901-0)
Supplement: Supplementary file 1 — Supplementary Information [file 41467_2022_32901_MOESM1_ESM.pdf]

Supplementary Information

Chimeric Natural Products Derived from Medermycin and the Nature-Inspired Construction of Their Polycyclic Skeletons

Shupeng Yin[1,‡], Zhi Liu[1,2,‡], Jingjing Shen[1,2], Yuwei Xia[1,2], Weihong Wang[1], Pengyan Gui[1], Qian Jia[1], Konthorn Kachanuban[1,3], Weiming Zhu[1,2,*] & Peng Fu[1,2,*]

[1] Key Laboratory of Marine Drugs, Ministry of Education of China, School of Medicine and Pharmacy, Ocean University of China, Qingdao 266003, China

[2] Laboratory for Marine Drugs and Bioproducts, Pilot National Laboratory for Marine Science and Technology (Qingdao), Qingdao 266237, China

[3] Department of Fishery Products, Faculty of Fisheries, Kasetsart University, Bangkok 10900, Thailand

‡ These authors contributed equally: Shupeng Yin, Zhi Liu

Supplementary Methods

Experimental Details

Cultivation and extraction of OUCMDZ-4982. The *Streptomyces* sp. OUCMDZ-4982 was cultured in 150 mL of a liquid medium (10 g of starch, 4 g of yeast extract, 2 g of peptone, 1 g of $CaCO_3$, 43 mg of $Fe_2(SO_4)_3{\cdot}4H_2O$, 0.1 g of KBr, and 1 L of seawater) in Erlenmeyer flasks (500 mL) and shaken for 3 days (28 °C, 180 rpm), which was used as the seed liquid. Then, the seed liquid (15 mL) was transferred to an Erlenmeyer flask (1 L) containing 120 g of rice and 80 mL of liquid medium with the same formula as seed liquid. A total of 220 Erlenmeyer flasks were used in this fermentation. After incubation in static cultivation for 60 days at 20 °C, the whole culture was extracted with EtOAc for three times. The resulting EtOAc part was concentrated *in vacuo* to yield 74.8 g of EtOAc extract.

LC-MS/MS and molecular networking analysis. LC-MS/MS was performed on a Waters series 2695 HPLC instrument, coupled with an amaZon SL ion trap Mass spectrometer (Bruker), with a YMC ODS-A column (250 mm × 4.6 mm, 5 μm). The organic portion was dissolved in MeOH at 1 mg/mL, filtered through a Gracepure C18 SPE cartridge, and analyzed by LC-MS/MS. A 10 μL aliquot of each sample was injected and eluted with a gradient program of MeOH-H_2O (0.1% formic acid) [0−20 min 10−100%, 21−25 min 100%; 1.0 mL/min; MS scan 100−1000 Da]. Mass spectra were obtained in positive ESI mode and with an automated full dependent MS/MS scan. The MS/MS data were converted digitally to .mzXML files using MSConvert software. The molecular networking was performed using the GNPS data analysis workflow using the spectral clustering algorithm. The spectral networks were imported into Cytoscape 3.7.1 and visualized using the force-directed layout.[1]

Purification. The EtOAc extract (74.8 g) was separated into nine fractions (Fr.1−Fr.9) on a silica gel VLC column using step gradient elution with CH_2Cl_2−petroleum ether (PE) (0−50%) and then MeOH−CH_2Cl_2 (0−50%). Fraction 8 (12.9 g) was fractionated into nine subfractions (Fr.8.1−Fr.8.9) on a reversed-phase silica gel column, eluting with a step gradient of MeOH−H_2O (10−100%). Fr.8.1 (1.0 g) was separated into eight fractions (Fr.8.1.1−Fr.8.1.8) on a silica gel column using step gradient elution with MeOH−CH_2Cl_2 (0−50%). Fr.8.1.6 (44 mg) was separated into six fractions (Fr.8.1.6.1−Fr.8.1.6.6) on Sephadex LH-20 eluting with MeOH. Fr.8.1.6.3 (12 mg) was further purified by semipreparative HPLC on a Phenyl column (YMC-Pack Ph, 10 × 250 mm, 5 μm, 2.5 mL/min) eluting with 26% MeCN (0.5‰ TFA) to yield compounds **6** (2.5 mg, t_R = 8.0 min) and **7** (2.8 mg, t_R = 15.5 min). Fr.8.3 (730 mg) was separated into nine fractions (Fr.8.3.1−Fr.8.3.9) on a silica gel column using step gradient elution with MeOH−CH_2Cl_2 (0−50%). Fr.8.3.3 (81 mg) was further purified by semipreparative HPLC on a C18-PFP column (ACE C18-PFP, 10 × 250 mm, 5 μm, 3.0 mL/min) eluting with 30% MeCN (0.5‰ TFA) to yield compound **9** (33.7 mg, t_R = 10.5 min). Fr.8.3.4 (500 mg) was separated into six fractions (Fr.8.3.4.1−Fr.8.3.4.6) on Sephadex LH-20 eluting with MeOH. Fr.8.3.4.4 (23 mg) was further purified by semipreparative HPLC on a π-NAP column (COSIMOSIL π-NAP, 10 × 250 mm, 5 μm, 3.0 mL/min) eluting with 40% MeCN (0.5‰ TFA) to yield compound **8** (3.5 mg, t_R = 15.0 min). Fr.8.4 (1.2 g) was separated into seven fractions (Fr.8.4.1−Fr.8.4.7) on a silica gel column using step gradient elution with MeOH−CH_2Cl_2 (0−50%). Fr.8.4.5 (87 mg) was separated into four fractions (Fr.8.4.5.1−Fr.8.4.5.4) on Sephadex LH-20 eluting with MeOH. Fr.8.4.5.1 (9 mg) was further purified by semipreparative HPLC on a C18-PFP column (ACE C18-PFP, 10 × 250 mm, 5 μm, 3.0 mL/min) eluting with 25% MeCN (0.5‰ TFA) to yield compound **5** (2.9 mg, t_R = 46.0 min). Fr.8.5 (335 mg) was further separated into four fractions (Fr.8.5.1−Fr.8.5.4) on Sephadex LH-20 eluting with MeOH−CH_2Cl_2 (1:1). Fr.8.5.1 (85 mg) was further purified by semipreparative HPLC on a C18-PFP column (ACE C18-PFP, 10 × 250 mm, 5 μm, 3.0 mL/min) eluting with 60% MeOH (0.5‰ TFA) to yield compound **1** (6.3 mg, t_R = 17.0 min). Fr.8.6 (2.3 g) was separated into nine fractions (Fr.8.6.1−Fr.8.6.9) on a silica gel column using step gradient elution with MeOH−CH_2Cl_2 (0−50%). Fr.8.6.5 (170 mg) was separated into four fractions (Fr.8.6.5.1−Fr.8.6.5.4) on Sephadex LH-20 eluting with MeOH. Fr.8.6.5.2

(90 mg) was further purified by semipreparative HPLC on a C18-PFP column (ACE C18-PFP, 10 × 250 mm, 5 μm, 3.0 mL/min) using a step gradient solvent system from 40% to 55% MeCN (0.5‰ TFA) over 30 min to afford compounds **2** (5.3 mg, t_R = 11.0 min), **3** (5.4 mg, t_R = 13.0 min), and **4** (3.6 mg, t_R = 22.0 min).

Chimedermycin A (1): yellow solid; $[\alpha]_D^{27}$ −173.5 (*c* 0.02, MeOH); UV (MeOH) λ_{max} (log ε) 237 (4.53), 345 (3.86) nm; ECD (0.67 *m*M, MeOH) λ_{max} ($\Delta\varepsilon$) 230 (+72.4), 244 (−77.2), 297 (+10.1), 344 (−24.3) nm; IR (KBr) ν_{max} 3442, 2930, 1682, 1648, 1574, 1540, 1422, 1386, 1205, 1132, 839, 804, 724 cm^{-1}; ^{1}H and ^{13}C NMR, see Supplementary Table 1; HRESIMS *m/z* 746.2798 $[M + H]^+$ (calcd for $C_{40}H_{44}O_{13}N$, 746.2807).

Chimedermycin B (2): yellow solid; $[\alpha]_D^{27}$ −307.0 (*c* 0.02, MeOH); UV (MeOH) λ_{max} (log ε) 237 (4.52), 346 (3.86) nm; ECD (0.66 *m*M, MeOH) λ_{max} ($\Delta\varepsilon$) 230 (+51.5), 244 (−61.4), 295 (+9.0), 345 (−19.7) nm; IR (KBr) ν_{max} 3421, 2936, 1682, 1643, 1575, 1429, 1384, 1265, 1205, 1132, 838, 803, 723 cm^{-1}; ^{1}H and ^{13}C NMR, see Supplementary Table 1; HRESIMS *m/z* 760.2980 $[M + H]^+$ (calcd for $C_{41}H_{46}O_{13}N$, 760.2964).

Chimedermycin C (3): yellow solid; $[\alpha]_D^{27}$ −363.5 (*c* 0.02, MeOH); UV (MeOH) λ_{max} (log ε) 238 (4.53), 349 (3.90) nm; ECD (0.66 *m*M, MeOH) λ_{max} ($\Delta\varepsilon$) 230 (+43.0), 245 (−52.3), 295 (+7.5), 348 (−17.5) nm; IR (KBr) ν_{max} 3430, 2933, 1681, 1575, 1428, 1384, 1269, 1204, 1134, 841, 802, 719 cm^{-1}; ^{1}H and ^{13}C NMR, see Supplementary Table 1; HRESIMS *m/z* 760.2961 $[M + H]^+$ (calcd for $C_{41}H_{46}O_{13}N$, 760.2964).

Chimedermycin D (4): yellow solid; $[\alpha]_D^{27}$ −441.0 (*c* 0.02, MeOH); UV (MeOH) λ_{max} (log ε) 238 (4.47), 352 (3.86) nm; ECD (0.65 *m*M, MeOH) λ_{max} ($\Delta\varepsilon$) 230 (+32.3), 245 (−40.8), 277 (+6.2), 349 (−13.3) nm; IR (KBr) ν_{max} 3421, 2940, 1682, 1575, 1428, 1382, 1269, 1205, 1131, 838, 803, 722, 667 cm^{-1}; ^{1}H and ^{13}C NMR, see Supplementary Table 2; HRESIMS *m/z* 774.3115 $[M + H]^+$ (calcd for $C_{42}H_{48}O_{13}N$, 774.3120).

Chimedermycin E (5): yellow solid; $[\alpha]_D^{27}$ −226.4 (*c* 0.01, MeOH); UV (MeOH) λ_{max} (log ε) 225 (5.40), 236 (5.37), 341 (4.70) nm; ECD (0.74 *m*M, MeOH) λ_{max} ($\Delta\varepsilon$) 229 (+29.4), 248 (−21.4), 278 (+6.8), 330 (−13.6) nm; IR (KBr) ν_{max} 3422, 2932, 1684, 1427, 1384, 1246, 1207, 1135, 1031, 840, 803, 724 cm^{-1}; ^{1}H and ^{13}C NMR, see Supplementary Table 2; HRESIMS *m/z* 674.2609 $[M + H]^+$ (calcd for $C_{37}H_{40}O_{11}N$, 674.2596).

Chimedermycin F (6): yellow solid; $[\alpha]_D^{26}$ +61.4 (*c* 0.05, MeOH); UV (MeOH) λ_{max} (log ε) 200 (3.98), 237 (4.10), 350 (3.54) nm; ECD (0.89 *m*M, MeOH) λ_{max} ($\Delta\varepsilon$) 228 (−6.3), 256 (+4.3), 275 (−0.5), 341 (+3.7), 380 (−0.4) nm; IR (KBr) ν_{max} 3422, 2926, 1681, 1429, 1383, 1256, 1206, 1132, 1015, 835, 809, 718 cm^{-1}; ^{1}H and ^{13}C NMR, see Supplementary Table 3; HRESIMS *m/z* 562.2273 $[M + H]^+$ (calcd for $C_{28}H_{36}O_{11}N$, 562.2283).

Chimedermycin G (7): yellow solid; $[\alpha]_D^{26}$ +67.0 (*c* 0.05, MeOH); UV (MeOH) λ_{max} (log ε) 200 (4.03), 237 (4.17), 350 (3.61) nm; ECD (0.87 *m*M, MeOH) λ_{max} ($\Delta\varepsilon$) 228 (−6.8), 256 (+4.9), 276 (−0.4), 342 (+4.1), 380 (−0.6) nm; IR (KBr) ν_{max} 3423, 2925, 2855, 1681, 1436, 1384, 1259, 1207, 1133, 1016, 842, 797, 720 cm^{-1}; ^{1}H and ^{13}C NMR, see Supplementary Table 3; HRESIMS *m/z* 576.2426 $[M + H]^+$ (calcd for $C_{29}H_{38}O_{11}N$, 576.2439).

Chimedermycin H (8): red solid; $[\alpha]_D^{26}$ +458.0 (*c* 0.005, MeOH); UV (MeOH) λ_{max} (log ε) 200 (4.51), 294 (3.90), 489 (3.96) nm; ECD (0.65 *m*M, MeOH) λ_{max} ($\Delta\varepsilon$) 222 (+6.69), 286 (+8.77), 383 (−1.60) nm; IR (KBr) ν_{max} 3421, 2926, 2856, 1787, 1683, 1604, 1457, 1383, 1275, 1207, 1136, 837, 799, 718 cm^{-1}; ^{1}H and ^{13}C NMR, see Supplementary Table 3; HRESIMS *m/z* 590.2016 $[M + H]^+$ (calcd for $C_{32}H_{32}O_{10}N$, 590.2021).

Medermycin (9): yellow solid; $[\alpha]_D^{23}$ +77.7 (*c* 0.1, MeOH); ECD (1.1 *m*M, MeOH) λ_{max} ($\Delta\varepsilon$) 215 (+11.8), 276 (−0.9), 342 (+2.6) nm; ESIMS *m/z* 458 $[M + H]^+$. It was identified by comparison of the spectroscopic data and specific rotation with those reported.[2–4]

Detailed structural elucidation. Chimedermycin A (**1**) was obtained as a yellow solid. Its molecular formula was determined as $C_{40}H_{43}NO_{13}$ based on the HRESIMS peak at *m/z* 746.2798 $[M + H]^+$ (Supplementary Fig. 8). The ^{13}C

NMR spectrum (Supplementary Fig. 10) showed 40 signals which were classified by HSQC (Supplementary Fig. 11) as two keto carbonyls (C-6/13), two carboxyl carbons (C-1/1″), three oxygenated sp^2 nonprotonated carbons (C-11/11″/13″), eight nonoxygenated sp^2 nonprotonated carbons (C-7/10/12/5″/7″/10″/12″/14″), one nonoxygenated sp^3 nonprotonated carbon (C-14), five sp^2 methine carbons (C-8/9/6″/8″/9″), eight oxygenated sp^3 methine carbons (C-3/4/15/1′/4′/5′/3″/15″), two nonoxygenated sp^3 methine carbons (C-5/3′), four methylene carbons (C-2/2′/2″/4″), and five methyl carbons (C-16/6′/7′/8′/16″). The ^{1}H NMR spectrum (Supplementary Fig. 9) showed two pairs of aromatic proton spin couplings at 7.51 (d, J = 7.9 Hz)/7.84 (d, J = 7.9 Hz) and 7.09 (d, J = 8.8 Hz)/6.84 (d, J = 8.7 Hz), revealing two characteristic 1,2,3,4-tetrasubstituted benzene rings. Further analysis of ^{1}H NMR data (Supplementary Table 1) revealed the presence of a 3-(*N*, *N*-dimethylamino)-2,6-dideoxyhexapyranose unit, which was confirmed by the contiguous COSY correlations extending from H-1′ to H_3-6′, and the key HMBC correlations of H_3-6′ to C-5′/C-4′, H_3-7′ to C-3′, and H_3-8′ to C-3′/C-7′ (Supplementary Fig. 2). In addition, the comparison of its ^{1}H and ^{13}C NMR spectra with those of medermycin (**9**) suggested that compound **1** contains a similar pyranonaphthoquinone moiety with medermycin.[3–5] The other part of compound **1** was determined as 6-deoxy-dihydrokalafungin (DDHK)[6,7] by analysis of remaining signals in its ^{1}H and ^{13}C NMR spectra, together with the COSY correlations of H-15″/H_3-16″ and H_2-2″/H-3″/H_2-4″, and the key HMBC correlations of H_2-2″ to C-1″/C-4″, H_2-4″ to C-5″/C-6″/C-14″, H-6″ to C-12″/C-14″, H-8″ to C-10″/C-12″, H-9″ to C-7″/C-11″, H-15″ to C-3″/C-13″, and H_3-16″ to C-14″ (Supplementary Fig. 2). The connection between medermycin moiety and DDHK moiety was confirmed by the HMBC correlations of H-4 to C-11″, H-5 to C-10″, and H-9″ to C-14 (Supplementary Fig. 2).

The relative configuration of the amino sugar was confirmed by the NOESY correlations of H-3′/H-5′, H-3′/H-2′a, H-4′/H-2′b, H-4′/H_3-6′, and H_3-6′/H-9 (Supplementary Fig. 2), together with the analysis of coupling constants (Supplementary Table 1). The NOESY correlations of H-3/H-4, H-4/H_3-16, H-3/H_3-16, H-5/H_3-16, and H-15/H-9″ (Supplementary Fig. 2) indicated the relative configuration around the bridged-ring system as (3*R**, 4*R**, 5*R**, 14*S**, 15*R**). The *trans*- configuration between H-3″ and H-15″ was determined by the NOESY correlation of H-3″/H_3-16″ (Supplementary Fig. 2). To determine the relative configuration of the whole molecule, δ_C values of four plausible stereoisomers (Supplementary Fig. 3a) were calculated at the B3LYP/6-311++G(2d,p) level (Supplementary Data 1−4).[8,9] The DP4+ probability analysis (Supplementary Fig. 3b)[10] suggests the relative configuration of chimedermycin A as **1A**. The absolute configuration of medermycin (**9**) has been confirmed by total synthesis.[2] Moreover, the stereochemistry of angolosamine ring in the natural medermycin-type derivatives isolated from *Streptomyces* species seems to be constant so far, because the genes related to this angolosamine ring are highly conserved (Fig. 2).[3–5,11–14] So, the absolute configuration of compound **1** could be speculated based on the shared biosynthetic origin with medermycin (**9**) (Fig. 3a). This deduction was verified by ECD calculations of **1A**−**1D** (Supplementary Fig. 3a) using the TDDFT [B3LYP/6-31G(d)] method[15] (Supplementary Fig. 3c). Furthermore, the absolute configuration of **1** was confirmed by the ECD exciton chirality method.[16] The ECD spectrum of **1** showed a negative Cotton effect at 344 nm and a positive Cotton effect at 297 nm (Supplementary Fig. 3c), which was indicative of negative chirality between the two chromophores (Supplementary Fig. 3a). Thus, the absolute configuration of compound **1** was determined as (3*R*, 4*R*, 5*R*, 14*S*, 15*R*, 1′*R*, 3′*R*, 4′*S*, 5′*R*, 3″*S*, 15″*R*), which was further confirmed by the semisynthesis of **1** from **9** and **10** (Fig. 3b).

The molecular formulas of chimedermycins B−D (**2**−**4**) were determined as $C_{41}H_{45}NO_{13}$, $C_{41}H_{45}NO_{13}$, and $C_{42}H_{47}NO_{13}$, respectively, based on their HRESIMS spectra (Supplementary Figs. 15, 22, 29). Comparison of their NMR spectra with those of **1** revealed that chimedermycins B−D (**2**−**4**) were different methyl ester derivatives of chimedermycin A (**1**) at two carboxyl groups (Fig. 1). Their structures were further confirmed by the chemical transformations from **2** to **1**, from **1** to **4**, and from **3** to **4**.

Chimedermycin E (**5**) was assigned a molecular formula of $C_{37}H_{39}NO_{11}$ based on the HRESIMS spectrum (Supplementary Fig. 36). Comparison of its ^{1}H and ^{13}C NMR spectra with those of compound **1** revealed that compound **5** contains a similar moiety derived from medermycin (**9**). The remaining NMR signals were attributed to

dehydroxy-GTRI-02,[17] which was confirmed by the COSY correlation of H-7″/H-8″, and the key HMBC correlations of H-1″ to C-2″/C-3″, H-5″ to C-3″/C-11″, H-7″ to C-5″/C-9″/C-11″, H-8″ to C-6″/C-10″, and H_3-13″ to C-3″/C-11″/C-12″ (Supplementary Fig. 2). The connection between medermycin moiety and dehydroxy-GTRI-02 moiety was confirmed by the HMBC correlations of H-4 to C-10″, H-5 to C-9″, and H-8″ to C-14 (Supplementary Fig. 2). The NOESY correlations (Supplementary Fig. 2) indicated that chimedermycin E (**5**) has the same relative configuration as compound **1**. The ECD Cotton effects of compound **5** were nearly identical to compound **1** (Supplementary Fig. 4), indicating the same absolute configuration. Medermycin (**9**) and dehydroxy-GTRI-02 may be the biosynthetic precursors of chimedermycin E (**5**).

Chimedermycin F (**6**) was obtained as a yellow solid. Its molecular formula was determined as $C_{28}H_{35}NO_{11}$ according to the HRESIMS spectrum (Supplementary Fig. 43). Analysis of the NMR data (Supplementary Fig. 2 and Supplementary Table 3) revealed that compound **6** contains the same moiety derived from medermycin (**9**) as compound **1**. The remaining polycyclic system was determined by the COSY correlations of H-15/H_3-16 and H_2-2/H-3/H-4, and the key HMBC correlations of H-1″ to C-2″/C-3″/C-13, H-4″ to C-2″/C-3″/C-5, H-15 to C-1″/C-3/C-5, and H-4 to C-6/C-14 (Supplementary Fig. 2). The amino sugar of **6** has the same relative configuration as compound **1**, according to the NOESY correlations of H-1′/H-3′, H-1′/H-5′, H-2′b/H-4′, H-2′b/H-9, and H-4′/H_3-6′ (Supplementary Fig. 2). Meanwhile, the NOESY correlations of H-4/H-4″, H-3/H-4, H-3/H_3-16, and H-1″/H-15 (Supplementary Fig. 2) suggested the relative configuration of the polycyclic skeleton as shown.

The molecular formula of chimedermycin G (**7**) was assigned to be $C_{29}H_{37}NO_{11}$ based on the HRESIMS spectrum (Supplementary Fig. 50), which was only one $-CH_2$ more than that of **6**. Analysis of its ^{1}H and ^{13}C NMR data (Supplementary Table 3) revealed that compound **7** was the methyl ester derivative of **6**. Its structure was further confirmed by the chemical transformation from **6** to **7**.

The molecular formula of chimedermycin H (**8**) was determined as $C_{32}H_{31}NO_{10}$ by HRESIMS (Supplementary Fig. 57). Comparison of its ^{1}H and ^{13}C NMR data (Supplementary Table 3) with those of medermycin (**9**) revealed that compound **8** has the same sugar unit, γ-lactone ring, and pyran ring as **9**. The core structural unit derived from medermycin was further confirmed by the COSY correlations of H-1′/H_2-2′/H-3′/H-4′/H-5′/H_3-6′, H-15/H_3-16, and H_2-2/H-3/H-4, as well as the key HMBC correlations of H_2-2 to C-1, H-3 to C-1, H-4 to C-6/C-14, H-9 to C-7/C-8/C-10/C-11/C-1′, H-15 to C-3/C-5/C-13, and H_3-16 to C-14 (Supplementary Fig. 2). In addition, the coupled ^{1}H NMR signals at δ_H 6.92 (d, J = 8.2 Hz) and 7.35 (d, J = 8.2 Hz) (Supplementary Table 3) revealed the presence of a 1,4-disubstituted benzene ring that was further identified as a 4-OH phenyl unit from the HMBC correlations of H-4″ to C-6″ and H-5″ to C-3″ (Supplementary Fig. 2). The HMBC correlations of H-4″ to C-2″ and H-9 to C-2″ (Supplementary Fig. 2), together with the analysis of the remaining NMR signals and the molecular formula indicated that the 4-OH phenyl group was attached to an additional δ-lactone ring. Therefore, the planar part of the structure of chimedermycin H (**8**) was elucidated as shown (Fig. 1). Its relative configuration was determined to be identical to that of medermycin (**9**) by the NOESY correlations of H-3/H-4, H-3/H_3-16, H-1′/H-3′, H-1′/H-5′, H-4′/H-2′b, H-2′b/H-9, and H-4′/H_3-6′ (Supplementary Fig. 2). The same absolute configuration of compound **8** as compound **9** was determined by the semisynthesis of **8** from **9** (Fig. 3b).

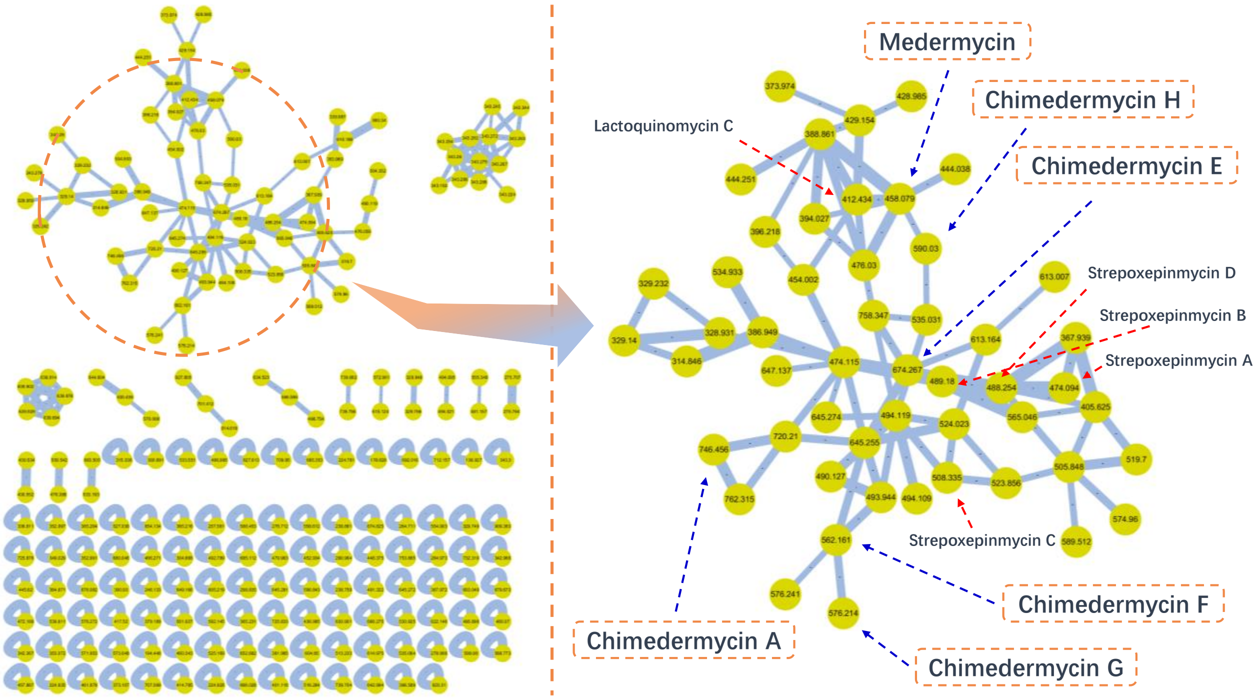

Supplementary Fig. 1. LC-MS/MS-based molecular networking of fraction 8 from *Streptomyces* sp. OUCMDZ-4982.

Chemical correlations among chimedermycins A−D (1−4)

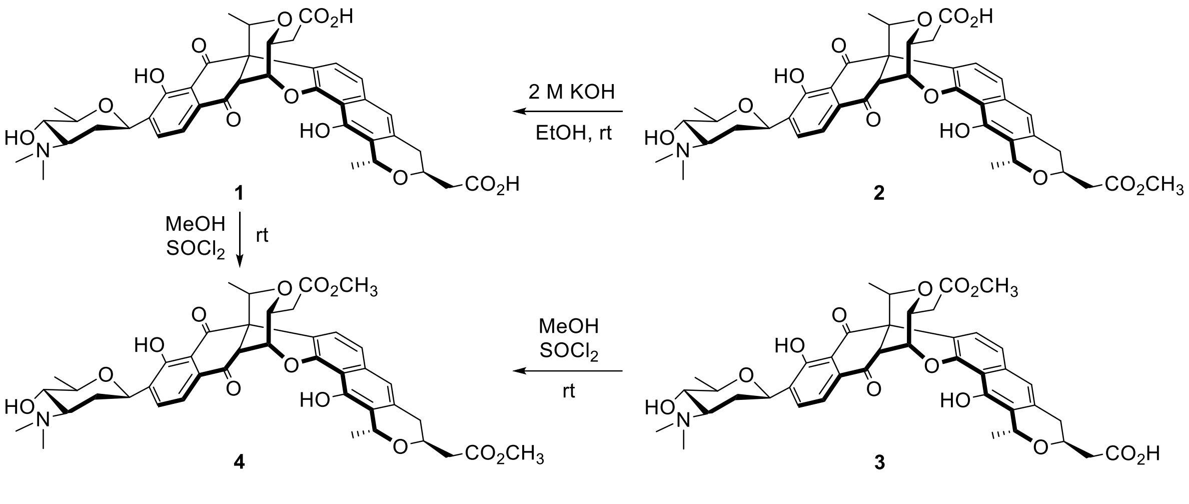

Chimedermycin A (**1**, 1.0 mg, 1.34 μmol) was dissolved in methanol (1.0 mL). Then, 10 μL of $SOCl_2$ was added at 0 °C. The mixture was stirred at room temperature (rt) for 13.5 h, and then was dried *in vacuo*. The product was purified by HPLC on a C18-PFP column (ACE C18-PFP, 10 × 250 mm, 5 μm, 3.0 mL/min) eluting with 47% MeCN (0.5‰ TFA) to yield chimedermycin D (**4**, 0.6 mg, t_R = 15.5 min). Chimedermycin B (**2**, 1.0 mg, 1.32 μmol) was dissolved in ethanol (500 μL). Then, 10 μL of aqueous KOH solution (2 M) was added at 0 °C. The mixture was stirred at rt for 6 h, and then was purified by HPLC on a C18-PFP column (ACE C18-PFP, 10 × 250 mm, 5 μm, 3.0 mL/min) eluting with 40% MeCN (0.5‰ TFA) to yield chimedermycin A (**1**, 0.5 mg, t_R = 7.0 min). Chimedermycin C (**3**, 0.5 mg, 0.66 μmol) was dissolved in methanol (500 μL). Then, 7 μL of $SOCl_2$ was added at 0 °C. The mixture was stirred at rt for 4.5 h, and then was dried *in vacuo* to yield chimedermycin D (**4**, 0.5 mg). All the products of reactions were identified to be consistent with the corresponding isolated natural products by LC-MS, ^{1}H NMR, and specific rotation.

Chemical correlation between chimedermycin F (6) and chimedermycin G (7)

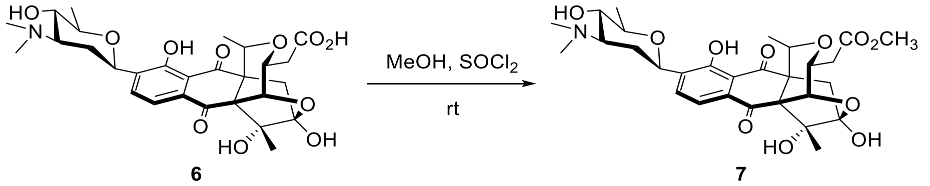

Chimedermycin F (**6**, 0.5 mg, 0.89 μmol) was dissolved in methanol (0.5 mL). Then, 7 μL of $SOCl_2$ was added at 0 °C. The mixture was stirred at rt for 12 h, and then was dried *in vacuo*. The product was purified by HPLC on a Phenyl column (YMC-Pack Ph, 10 × 250 mm, 5 μm, 3.0 mL/min) eluting with 30% MeCN (0.5‰ TFA) to yield chimedermycin G (**7**, 0.2 mg, t_R = 11.0 min), which was identified to be consistent with the corresponding isolated natural product by LC-MS, ^{1}H NMR, and specific rotation.

Semisynthesis of chimedermycin A (1) in two steps

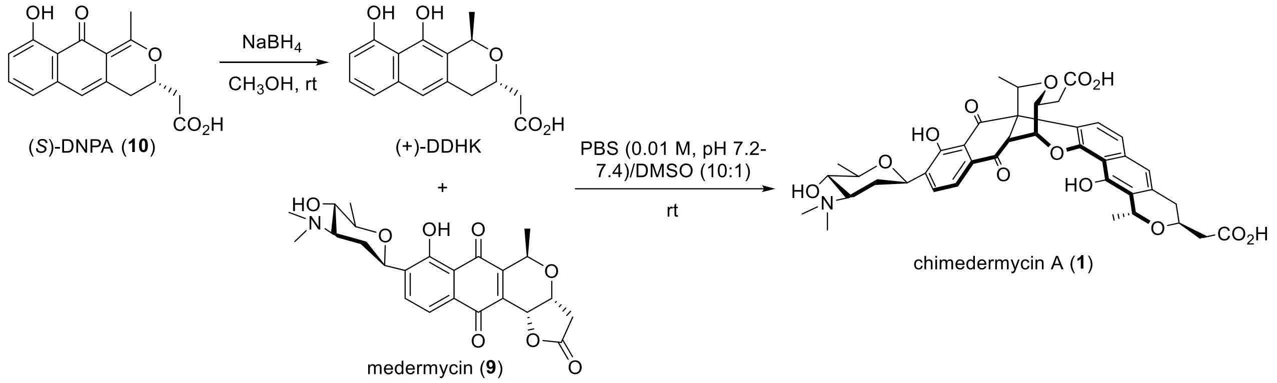

Isolation of (*S*)-DNPA (10) from the *Streptomyces* sp. OUCMDZ-4182. The *Streptomyces* sp. OUCMDZ-4182 was cultured in 150 mL of a liquid medium (10 g starch, 4 g yeast extract, 2 g peptone, 1 g $CaCO_3$, 43 mg $Fe_2(SO_4)_3{\cdot}4H_2O$, 0.1 g KBr, and 1 L seawater) in Erlenmeyer flasks (500 mL) and shaken for 3 days (28 °C, 180 rpm), which was used as the seed liquid. Then, the seed liquid (10 mL) was transferred to a mushroom spawn bag containing 120 g of rice and 80 mL of liquid medium with the same formula as seed liquid. A total of 200 bags were used in this fermentation. After incubation in static cultivation for 50 days at 25 °C, the whole culture was extracted with EtOAc for three times. The resulting EtOAc part was concentrated *in vacuo* to yield 30.2 g of EtOAc extract. The EtOAc extract (30.2 g) was separated into 10 fractions (Fr.1−Fr.10) on a silica gel VLC column using step gradient elution with CH_2Cl_2−PE (0−50%) and then MeOH−CH_2Cl_2 (0−50%). Fraction 6 (11.0 g) was further fractionated into 7 subfractions (Fr.6.1−Fr.6.7) on a silica gel VLC column using step gradient elution with CH_2Cl_2−PE (0−50%) and then MeOH−CH_2Cl_2 (0−50%). Fr.6.7 (1.4 g) was fractionated into ten fractions (Fr.6.7.1−Fr.6.7.10) on a reversed-phase silica gel column, eluting with a step gradient of MeOH−H_2O (10−100%). Fr.6.7.9 (40 mg) was further separated into three fractions (Fr.6.7.9.1−Fr.6.7.9.3) on Sephadex LH-20 eluting with MeOH. Fr.6.7.9.3 (10 mg) was further purified by semipreparative HPLC on a π-NAP column (COSIMOSIL π-NAP, 10 × 250 mm, 5 μm, 4.0 mL/min) eluting with 48% MeCN (0.5‰ TFA) to yield compound **10** (1.0 mg, t_R = 14.5 min). $[\alpha]_D^{28}$ +291.3 (*c* 0.005, MeOH); ^{1}H NMR (600 MHz, DMSO-d_6) δ 14.05 (s, 1H), 12.56 (brs, 1H), 7.46 (t, *J* = 7.9 Hz, 1H), 6.87 (d, *J* = 7.6 Hz, 1H), 6.70 (d, *J* = 8.0 Hz, 1H), 6.43 (s, 1H), 4.72 (m, 1H), 2.95 (dd, *J* = 16.1, 3.1 Hz, 1H), 2.81 (overlapped, 2H), 2.76 (dd, *J* = 16.4, 7.6 Hz, 1H), 2.58 (s, 3H); ^{13}C NMR (150 MHz, DMSO-d_6) δ 188.0, 178.5, 171.0, 162.8, 138.9, 135.5, 129.6, 117.0, 115.4, 115.0, 113.5, 110.6, 76.2, 38.4, 31.9, 23.4; ESIMS *m/z* 287 $[M + H]^+$. The specific rotation and spectroscopic data correspond to previously reported data.[18,19]

Preparation of chimedermycin A (1) from (*S*)-DNPA (10). (*S*)-DNPA (**10**, 1.0 mg, 3.50 μmol) was dissolved in methanol (1.0 mL). Then, 5.0 mg of $NaBH_4$ (0.13 mmol) was added. The reaction was allowed to proceed at rt for 10 min and detected using LC-MS. The substrate was exhausted, and the desired product (+)-DDHK was observed. Then, 0.06 M HCl was added to adjust the pH to 7.0. The mixture was dried *in vacuo* and purified by HPLC on a π-NAP column (COSIMOSIL π-NAP, 10 × 250 mm, 5 μm, 2.5 mL/min) eluting with 40% MeCN to yield (+)-DDHK (0.4 mg, t_R = 20.5 min, 40% yield). (+)-DDHK was not stable, so it was directly put into the next step without identification. (+)-DDHK (0.4 mg, 0.39 μmol) and medermycin (**9**, 0.2 mg, 0.44 μmol) were dissolved in DMSO (100 μL). Then, 1.0 mL of PBS (0.01 M, pH 7.2−7.4) was added. The reaction was allowed to proceed at rt for 1 h. The mixture was dried *in vacuo* and further purified by semipreparative HPLC on a C18-PFP column (ACE C18-PFP, 10 × 250 mm, 5 μm, 3.0 mL/min) eluting with 60% MeOH (0.5‰ TFA) to yield compound **1** (0.2 mg, t_R = 17.0 min, 19% yield).

Semisynthesis of chimedermycin F (6) in two steps

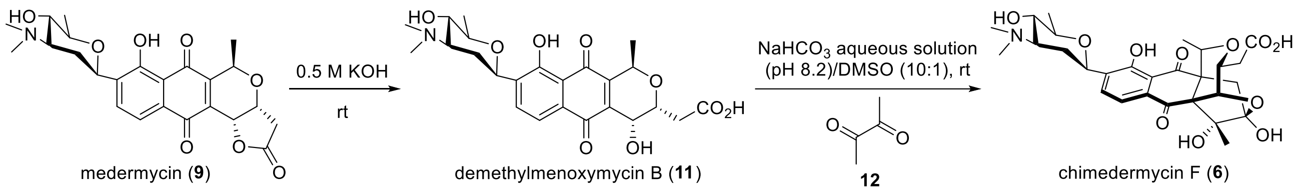

Medermycin (**9**, 3.0 mg, 6.56 μmol) was dissolved in 0.5 M KOH aqueous solution (500 μL). The reaction mixture was stirred at room temperature for 30 min. Then, 0.6 M HCl was added to adjust the pH to 7.0. The mixture was dried *in vacuo* and purified by HPLC on a C18-PFP column (ACE C18-PFP, 10 × 250 mm, 5 μm, 3.0 mL/min) eluting with 25% MeCN to yield demethylmenoxymycin B (**11**) (1.8 mg, t_R = 15.0 min, 58% yield). $[\alpha]_D^{26}$ +119.2 (*c* 0.01, MeOH); UV (MeOH) λ_{max} (log ε) 215 (4.35), 246 (3.82), 425 (3.46) nm; ECD (1.10 *m*M, MeOH) λ_{max} ($\Delta\varepsilon$) 215 (+5.9), 260 (5.7), 300 (−3.9), 356 (+2.4) nm; IR (KBr) ν_{max} 3430, 2925, 1645, 1613, 1575, 1386, 1279, 1086, 1030 cm^{-1}; ^{1}H NMR (500 MHz, methanol-d_4) δ 7.87 (d, *J* = 7.9 Hz, 1H, H-9), 7.64 (d, *J* = 7.9 Hz, 1H, H-8), 4.96 (d, *J* = 9.8 Hz, 1H, H-1′), 4.92 (q, *J* = 6.8 Hz, 1H, H-15), 4.62 (d, *J* = 1.6 Hz, 1H, H-4), 4.25 (ddd, *J* = 7.6, 6.0, 1.6 Hz, 1H, H-3), 3.55 (dq, *J* = 8.3, 6.1 Hz, 1H, H-5′), 3.41 (dd, *J* = 10.0, 8.3 Hz, 1H, H-4′), 3.39 (m, 1H, H-3′), 2.73 (s, 6H, H-7′&8′), 2.66 (dd, *J* = 15.1, 6.1 Hz, 1H, H-2a), 2.58 (dd, *J* = 15.1, 7.4 Hz, 1H, H-2b), 2.48 (m, 1H, H-2′a), 1.59 (m, 1H, H-2′b), 1.41 (d, *J* = 6.1 Hz, 3H, H-6′), 1.54 (d, *J* = 6.8 Hz, 3H, H-16); ^{13}C NMR (125 MHz, methanol-d_4) δ 191.3 (C-13), 183.5 (C-6), 178.6 (C-1), 158.6 (C-11), 147.9 (C-14), 143.2 (C-5), 137.9 (C-10), 134.2 (C-9), 132.3 (C-7), 119.7 (C-8), 115.8 (C-12), 78.7 (C-5′), 72.7 (C-1′), 71.9 (C-4′), 70.3 (C-3), 68.2 (C-15&3′), 60.7 (C-4), 40.2 (C-7′&8′), 40.0 (C-2), 30.8 (C-2′), 18.4 (C-16&6′); HRESIMS *m/z* 476.1906 [M + H]$^+$ (calcd for $C_{24}H_{30}O_9N$, 476.1915).

Demethylmenoxymycin B (**11**, 1.8 mg, 3.79 μmol) and butane-2,3-dione (**12**, 2 μL, 22.91 μmol) were dissolved in DMSO (150 μL). Then, 1.5 mL of $NaHCO_3$ aqueous solution (pH 8.2) was added. The reaction was allowed to proceed at rt for 24 h. Then, 0.6 M HCl was added to adjust the pH to 6.5. The mixture was extracted with n-butanol for three times (5 mL for each). The organic layers were combined and dried *in vacuo*. The product was further purified by semipreparative HPLC on a Phenyl column (YMC-Pack Ph, 10 × 250 mm, 5 μm, 2.5 mL/min) eluting with 26% MeCN (0.5‰ TFA) to yield chimedermycin F (**6**) (1.0 mg, t_R = 9.0 min, 47% yield).

Semisynthesis of chimedermycin H (8)

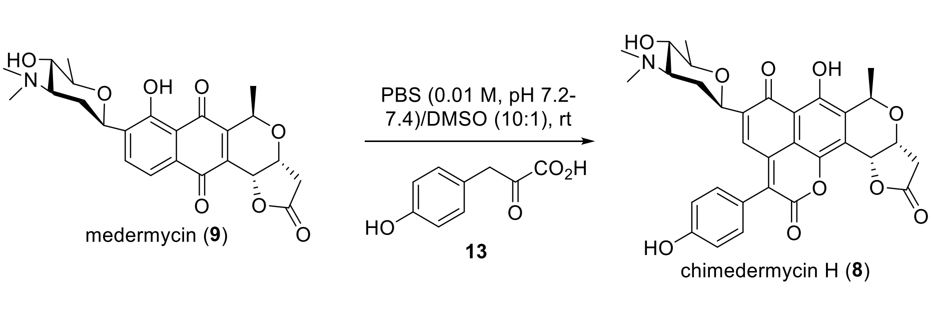

Medermycin (**9**, 2.0 mg, 4.38 μmol) and 4-hydroxyphenylpyruvic acid (**13**, 2.2 mg, 12.22 μmol) were dissolved in DMSO (150 μL). Then, 1.5 mL of PBS (0.01 M, pH 7.2−7.4) was added. The reaction was allowed to proceed at rt for 6 d. The reaction mixture was extracted with n-butanol for three times (5 mL for each). The organic layers were combined and dried *in vacuo*. The product was further purified by semipreparative HPLC on a π-NAP column (COSIMOSIL π-NAP, 10 × 250 mm, 5 μm, 3.0 mL/min) eluting with 40% MeCN (0.5‰ TFA) to yield chimedermycin H (**8**) (1.3 mg, t_R = 9.0 min, 50% yield).

Supplementary Table 1. ^{1}H and ^{13}C NMR data for chimedermycins A−C (**1**−**3**) in methanol-d_4

No.	**1**[a]		**2**[b]		**3**[c]	
	δ_C	δ_H, mult. (*J* in Hz)	δ_C	δ_H, mult. (*J* in Hz)	δ_C	δ_H, mult. (*J* in Hz)
1	174.1, C		174.0, C		172.7, C	
2	38.0, CH_2	2.90, dd (15.7, 4.9);	37.9, CH_2	2.89, dd (15.6, 4.9);	37.8, CH_2	2.91, dd (15.5, 5.0);
		2.64, dd (15.7, 8.5)		2.63, dd (15.6, 8.5)		2.65, dd (15.7, 8.5)
3	72.1, CH	4.52, dd (8.7, 4.8)	72.1, CH	4.51, dd (8.5, 4.9)	71.9, CH	4.52, dd (8.6, 4.9)
4	71.1, CH	5.41, s	71.2, CH	5.40, s	71.1, CH	5.37, s
5	43.8, CH	4.25, d (1.8)	43.8, CH	4.24, d (1.9)	43.8, CH	4.24, d (1.9)
6	192.2, C		192.2, C		192.2, C	
7	133.7, C		133.7, C		133.7, C	
8	119.2, CH	7.51, d (7.9)	119.2, CH	7.51, d (7.9)	119.2, CH	7.52, d (7.9)
9	135.1, CH	7.84, d (7.9)	135.1, CH	7.84, d (7.9)	135.2, CH	7.86, d (7.9)
10	137.9, C		137.9, C		137.9, C	
11	159.2, C		159.2, C		159.2, C	
12	117.3, C		117.3, C		117.3, C	
13	204.1, C		204.1, C		204.1, C	
14	52.8, C		52.9, C		52.8, C	
15	76.1, CH	4.43, q (6.5)	76.2, CH	4.43, q (6.4)	76.2, CH	4.41, q (6.6)
16	14.6, CH_3	1.61, d (6.5)	14.5, CH_3	1.60, d (6.6)	14.5, CH_3	1.60, d (6.6)
1′	72.3, CH	4.88, overlapped	72.3, CH	4.93, d (11.2)	72.3, CH	4.99, d (10.5)
2′	30.1, CH_2	2.48, m;	30.2, CH_2	2.49, m;	30.1, CH_2	2.50, m;
		1.56, overlapped		1.59, overlapped		1.60, overlapped
3′	68.4, CH	3.57, m	68.5, CH	3.58, m	68.5, CH	3.59, m
4′	71.2, CH	3.39, dd (10.2, 8.6)	71.2, CH	3.40, dd (10.2, 8.6)	71.1, CH	3.41, dd (10.3, 8.8)
5′	78.5, CH	3.48, dq (8.7, 6.1)	78.5, CH	3.51, dq (8.7, 6.2)	78.5, CH	3.53, dq (8.6, 6.2)
6′	18.2, CH_3	1.38, d (6.1)	18.2, CH_3	1.38, d (6.1)	18.2, CH_3	1.39, d (6.1)
7′	42.2, CH_3	2.89, s	42.2, CH_3	2.90, s	42.2, CH_3	2.91, s
8′	37.3, CH_3	2.73, s	37.5, CH_3	2.74, s	37.3, CH_3	2.75, s
1″	174.8, C		173.3, C		174.8, C	
2″	41.9, CH_2	2.59, dd (15.3, 4.4);	41.7, CH_2	2.63, dd (15.6, 4.5);	41.9, CH_2	2.61, dd (15.5, 4.6);
		2.50, dd (15.4, 8.6)		2.53, dd (15.4, 8.7)		2.53, dd (15.7, 8.3)
3″	65.4, CH	4.41, m	65.4, CH	4.43, m	65.4, CH	4.45, m
4″	34.8, CH_2	2.84, dd (16.7, 3.1);	34.8, CH_2	2.83, dd (16.8, 3.2);	34.8, CH_2	2.88, dd (16.5, 3.1);
		2.66, dd (16.8, 11.0)		2.67, dd (16.8, 11.1)		2.72, dd (16.5, 11.3)
5″	135.4, C		135.4, C		135.4, C	
6″	118.8, CH	6.94, s	118.9, CH	6.94, s	118.9, CH	6.98, s
7″	136.0, C		136.1, C		136.1, C	
8″	122.1, CH	7.09, d (8.8)	122.1, CH	7.09, d (8.9)	122.2, CH	7.11, d (8.9)
9″	122.9, CH	6.84, d (8.7)	122.9, CH	6.84, d (8.8)	122.8, CH	6.84, d (8.8)
10″	114.9, C		114.9, C		114.9, C	
11″	151.9, C		151.9, C		151.9, C	
12″	113.4, C		113.5, C		113.4, C	
13″	150.5, C		150.5, C		150.5, C	
14″	123.1, C		123.1, C		123.1, C	
15″	70.1, CH	5.23, q (6.5)	70.1, CH	5.22, q (6.5)	70.1, CH	5.26, q (6.6)
16″	19.2, CH_3	1.56, d (6.5)	19.3, CH_3	1.55, d (6.6)	19.3, CH_3	1.59, d (6.6)
1-OCH_3					52.4, CH_3	3.71, s
1″-OCH_3			52.2, CH_3	3.72, s		

[a] Recorded at 600 MHz for ^{1}H and 150 MHz for ^{13}C. [b] Recorded at 500 MHz for ^{1}H and 125 MHz for ^{13}C. [c] Recorded at 500 MHz for ^{1}H and 150 MHz for ^{13}C.

Supplementary Table 2. ^{1}H and ^{13}C NMR data for chimedermycins D (**4**) and E (**5**) in methanol-d_4

No.	**4**[a]		**5**[b]	
	δ_C	δ_H, mult. (J in Hz)	δ_C	δ_H, mult. (J in Hz)
1	172.6, C		174.2, C	
2	37.8, CH_2	2.90, dd (15.6, 4.9);	38.0, CH_2	2.81, dd (15.9, 5.1)
		2.65, dd (15.6, 8.8)		2.59, dd (15.9, 8.4)
3	71.9, CH	4.52, dd (8.6, 4.9)	72.3, CH	4.45, dd (8.5, 5.3)
4	71.1, CH	5.37, s	69.7, CH	5.21, s
5	43.8, CH	4.24, d (1.9)	43.8, CH	4.13, d (1.8)
6	192.1, C		192.5, C	
7	133.7, C		133.9, C	
8	119.2, CH	7.52, d (7.9)	119.0, CH	7.48, d (7.9)
9	135.1, CH	7.87, d (8.0)	135.3, CH	7.85, d (7.9)
10	137.9, C		137.7, C	
11	159.2, C		159.2, C	
12	117.3, C		117.4, C	
13	204.0, C		204.7, C	
14	52.9, C		53.2, C	
15	76.2, CH	4.41, q (6.6)	76.4, CH	4.42, q (6.6)
16	14.5, CH_3	1.59, d (6.6)	14.6, CH_3	1.59, d (6.6)
1′	72.3, CH	5.01, d (10.5)	72.3, CH	5.01, d (10.4)
2′	30.2, CH_2	2.51, m;	30.2, CH_2	2.49, m;
		1.60, overlapped		1.60, overlapped
3′	68.6, CH	3.60, m	68.5, CH	3.61, m
4′	71.2, CH	3.42, dd (9.9, 9.0)	71.2, CH	3.42, dd (9.8, 9.0)
5′	78.5, CH	3.55, dq (8.5, 6.0)	78.5, CH	3.55, dq (8.6, 6.2)
6′	18.2, CH_3	1.39, d (6.1)	18.2, CH_3	1.39, d (6.1)
7′	42.2, CH_3	2.91, s	42.1, CH_3	2.90, brs
8′	37.4, CH_3	2.75, s	37.2, CH_3	2.77, brs
1″	173.3, C		32.7, CH_3	2.53, s
2″	41.7, CH_2	2.65, dd (15.6, 4.8);	209.7, C	
		2.57, dd (15.6, 8.6)		
3″	65.4, CH	4.46, m	135.3, C	
4″	34.8, CH_2	2.88, dd (16.4, 3.2);	152.9, C	
		2.70, dd (16.9, 11.4)		
5″	135.5, C		108.8, CH	6.81, s
6″	118.9, CH	6.99, s	138.5, C	
7″	136.1, C		120.5, CH	6.96, d (8.7)
8″	122.2, CH	7.12, d (8.8)	124.2, CH	6.88, d (8.7)
9″	122.8, CH	6.85, d (8.8)	114.9, C	
10″	114.9, C		154.3, C	
11″	151.9, C		120.1, C	
12″	113.5, C		133.3, C	
13″	150.5, C		20.8, CH_3	2.71, s
14″	123.1, C			
15″	70.2, CH	5.26, q (6.6)		
16″	19.3, CH_3	1.58, d (6.6)		
1-OCH_3	52.4, CH_3	3.70, s		
1″-OCH_3	52.1, CH_3	3.72, s		

[a] Recorded at 500 MHz for ^{1}H and 125 MHz for ^{13}C. [b] Recorded at 500 MHz for ^{1}H and 150 MHz for ^{13}C.

Supplementary Table 3. ^{1}H (500 MHz) and ^{13}C (125 MHz) NMR data for chimedermycins F−H (**6**−**8**) in methanol-*d*$_4$

No.	**6**		**7**		**8**	
	δ_C	δ_H, mult. (*J* in Hz)	δ_C	δ_H, mult. (*J* in Hz)	δ_C	δ_H, mult. (*J* in Hz)
1	174.6, C		173.1, C		177.4, C	
2	37.7, CH_2	2.65, dd (15.9, 8.2); 2.61, dd (16.1, 4.9)	37.6, CH_2	2.70, dd (16.0, 8.9); 2.64, dd (16.0, 4.2)	37.9, CH_2	3.23, dd (17.8, 5.1); 2.62, d (17.8)
3	66.4, CH	4.06, dd (9.0, 5.0)	66.4, CH	4.06, dd (9.0, 4.5)	67.6, CH	4.94, dd (5.1, 3.1)
4	71.8, CH	4.47, s	71.7, CH	4.45, s	71.2, CH	5.70, d (2.9)
5	62.0, C		62.0, C		124.7, C	
6	195.4, C		195.4, C		144.5, C	
7	136.1, C		136.1, C		115.7, C	
8	118.5, CH	7.68, d (8.0)	118.5, CH	7.68, d (8.0)	111.9, C	
9	133.9, CH	7.90, d (8.0)	133.9, CH	7.91, d (8.0)	135.3, CH	7.89, d (1.4)
10	137.3, C		137.4, C		144.6, C	
11	158.4, C		158.4, C		187.9, C	
12	120.8, C		120.7, C		134.6, C	
13	207.8, C		207.7, C		156.5, C	
14	57.1, C		57.0, C		134.2, C	
15	75.3, CH	3.98, q (6.8)	75.3, CH	3.96, q (6.9)	68.4, CH	5.32, q (6.8)
16	17.1, CH_3	0.87, d (6.9)	17.1, CH_3	0.86, d (6.9)	18.1, CH_3	1.63, d (6.8)
1′	72.6, CH	5.00, dd (10.8, 2.1)	72.6, CH	5.00, dd (10.8, 2.0)	72.5, CH	4.83, dd (10.9, 1.4)
2′	30.2, CH_2	2.55, m; 1.67, m	30.2, CH_2	2.55, m; 1.67, m	30.3, CH_2	2.63, m; 1.57, m
3′	68.6, CH	3.61, m	68.6, CH	3.62, m	68.4, CH	3.59, m
4′	71.3, CH	3.47, dd (9.9, 9.0)	71.3, CH	3.47, dd (10.1, 8.8)	71.0, CH	3.39, dd (10.1, 9.0)
5′	78.6, CH	3.57, m	78.6, CH	3.57, m	78.4, CH	3.53, dq (8.7, 6.3)
6′	18.2, CH_3	1.42, d (6.0)	18.2, CH_3	1.42, d (6.1)	18.1, CH_3	1.34, d (6.1)
7′	42.1, CH_3	2.92, s	42.1, CH_3	2.92, s	42.2, CH_3	2.92, s
8′	37.6, CH_3	2.80, s	37.6, CH_3	2.80, s	37.4, CH_3	2.76, s
1″	44.0, CH_2	2.92, d (12.0); 2.37, d (12.3)	44.0, CH_2	2.91, d (12.2); 2.35, d (12.3)	161.8, C	
2″	109.1, C		109.1, C		133.5, C	
3″	86.0, C		86.0, C		123.9, C	
4″/8″	13.4, CH_3	1.18, s	13.4, CH_3	1.18, s	134.4, CH	7.35, d (8.2)
5″/7″					116.1, CH	6.92, d (8.2)
6″					160.7, C	
1-OCH_3			52.2, CH_3	3.68, s		

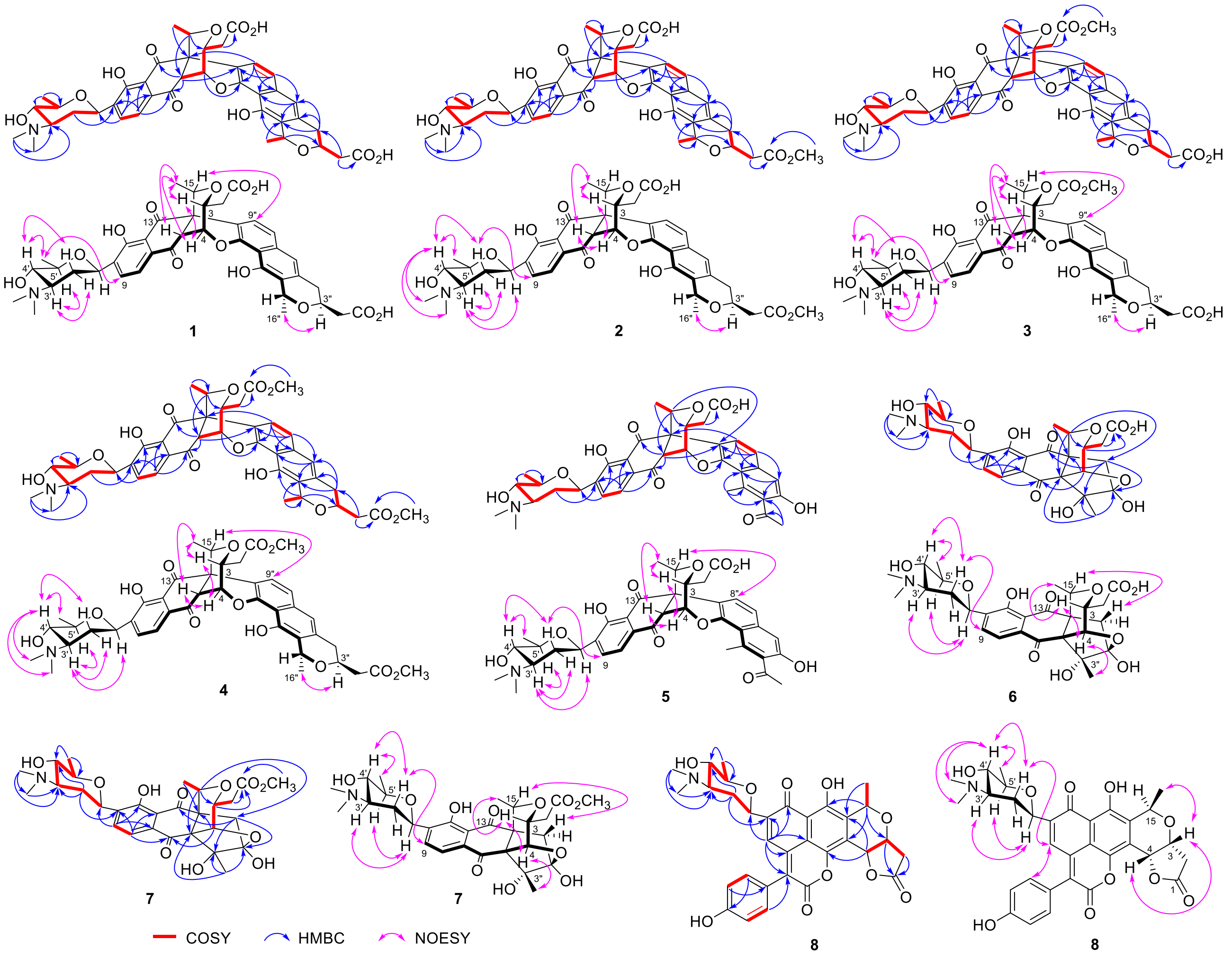

Supplementary Fig. 2. Key 2D NMR correlations of chimedermycins A−H (**1**−**8**).

a

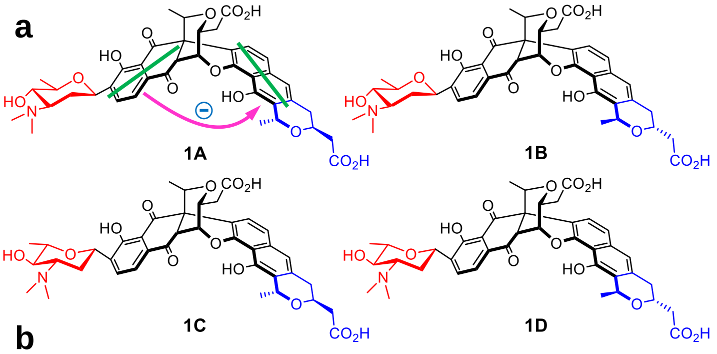

b

DP4+ probability of ^{13}C NMR of 1				
**Plausible isomer**	**1A**	**1B**	**1C**	**1D**
**DP4+**	**82.63%**	**0.04%**	**3.49%**	**13.84%**

c

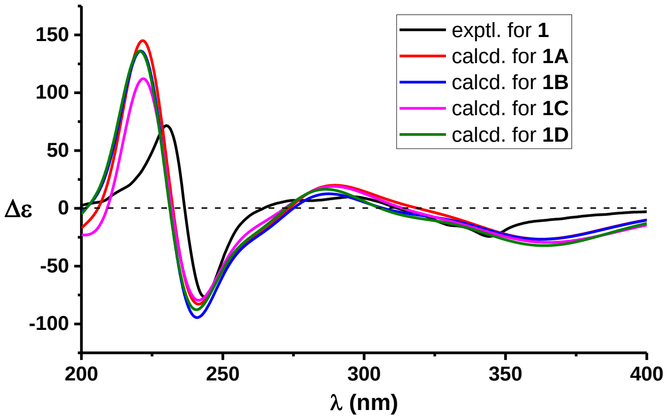

Supplementary Fig. 3. Assignment of absolute configuration for compound **1** using ^{13}C NMR and ECD calculations in combination with exciton chirality method. (a) Four plausible stereoisomers used in the calculations. Green bold lines denote the electric dipole of the chromophores. Pink arrow shows the counterclockwise array responsible for the negative exciton couplet. The sign of an exciton couplet consistent with the measured ECD Cotton effects can be directly correlated with the molecular stereochemistry. (b) The DP4+ probability of ^{13}C NMR chemical shifts. The results indicated that the most likely structure of compound **1** is **1A**. (c) Calculated and experimental ECD spectra. The calculated ECD curves for four plausible stereoisomers are almost same, which suggests that the configurations of sugar unit and the 6-deoxy-dihydrokalafungin (DDHK) moiety have little effect on the ECD Cotton effects. The deciding factor is the stereochemistry at the junction of the two fragments (medermycin and DDHK), which can be determined by the ECD calculations.

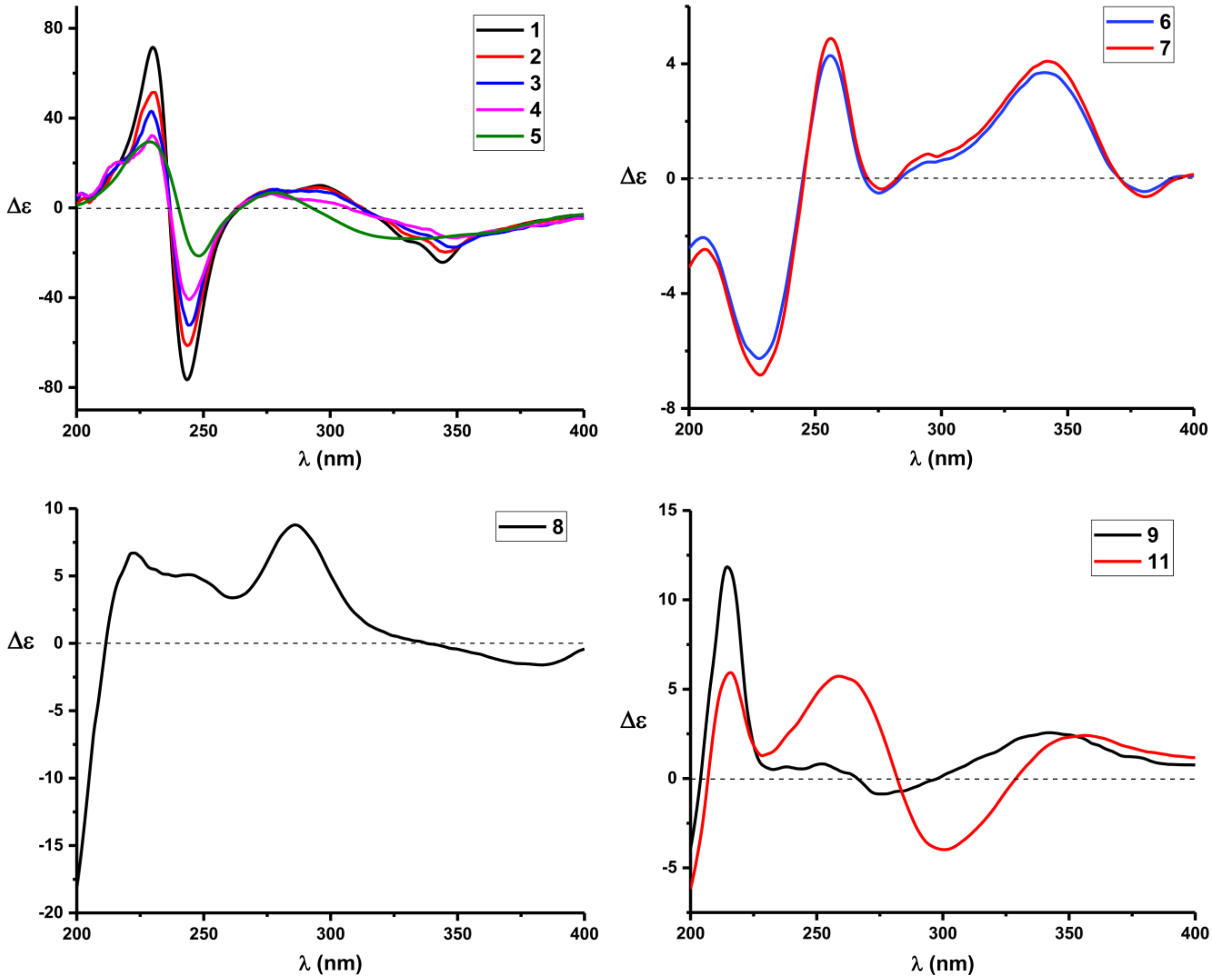

Supplementary Fig. 4. ECD curves of chimedermycins A−H (**1**−**8**), medermycin (**9**), and demethylmenoxymycin B (**11**).

Synthetic procedures for pyoluteorin (20)

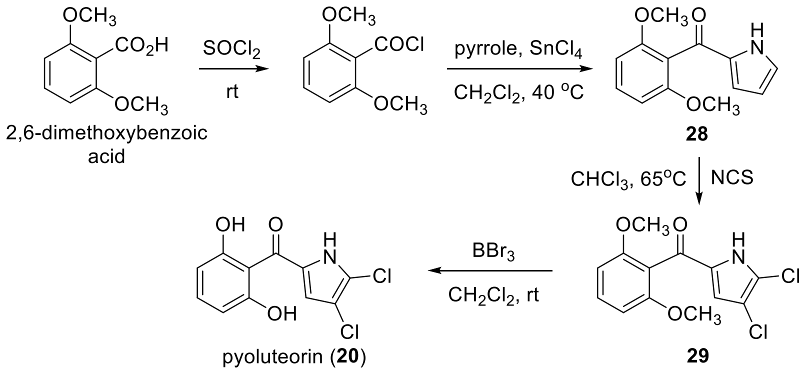

2,6-Dimethoxybenzoic acid (5.0 g, 27.47 mmol) was dissolved in $SOCl_2$ (10 mL, 137.65 mmol). The mixture was stirred at rt for 1.5 h. Then, the solution was concentrated on a rotary evaporator to give 2,6-dimethoxybenzoyl chloride as a yellow oil, which was dissolved in CH_2Cl_2 (40 mL) and cooled to 0 °C. $SnCl_4$ (16 mL, 136.98 mmol) and pyrrole (1.8 mL, 26.04 mmol) were added, and the mixture was allowed to rt for 1 h. The mixture was then refluxing for 3.5 h. After cooling to rt, 1 N aqueous H_2SO_4 (50 mL) was added. The reaction product was extracted with CH_2Cl_2 (150 mL). The organics were washed with brine and dried over Na_2SO_4, followed by purification by flash column chromatography eluting with 18% EtOAc−PE to give (1*H*-pyrrol-2-yl)-2,6-dimethoxyphenylmethanone (**28**) (4.20 g, 67% yield). ^{1}H NMR (400 MHz, DMSO-d_6) δ 11.86 (s, 1H), 7.35 (t, J = 8.4 Hz, 1H), 7.09 (m, 1H), 6.72 (d, J = 8.4 Hz, 2H), 6.29 (m, 1H), 6.11 (dt, J = 3.8, 2.3 Hz, 1H), 3.66 (s, 6H); ESIMS *m/z* 232 $[M + H]^+$. The spectroscopic data corresponds to previously reported data.[20]

Compound **28** (800 mg, 3.46 mmol) was dissolved in $CHCl_3$ (8 mL) and cooled to 0 °C. Then, the solution of *N*-chlorosuccinimide (1.0 g, 7.52 mmol) in $CHCl_3$ (7 mL) was added. The mixture was brought to reflux for 2 h. The reaction was quenched by adding saturated $NaHCO_3$ aqueous solution (20 mL). The product was extracted with CH_2Cl_2 (90 mL). The organics were washed with brine and dried over Na_2SO_4. It was further purified by flash column chromatography (10−20% EtOAc−PE) to give (4,5-dichloro-1*H*-pyrrol-2-yl)-2,6-dimethoxyphenyl methanone (**29**) (460 mg, 44% yield). ^{1}H NMR (400 MHz, DMSO-d_6) δ 13.24 (s, 1H), 7.38 (t, J = 8.4 Hz, 1H), 6.73 (d, J = 8.5 Hz, 2H), 6.38 (d, J = 2.8 Hz, 1H), 3.68 (s, 6H); ESIMS *m/z* 300 $[M + H]^+$. The spectroscopic data corresponds to previously reported data.[21]

Compound **29** (300 mg, 1.00 mmol) was dissolved in CH_2Cl_2 (20 mL) and cooled to −78 °C. Then, the solution of BBr_3 in CH_2Cl_2 (1.0 M, 4.00 mmol) was added dropwise. The mixture was stirred at rt for 1 h. Then, the reaction was quenched by adding saturated $NaHCO_3$ aqueous solution (10 mL). The product was extracted with CH_2Cl_2 (60 mL) and purified by flash column chromatography eluting with 20% EtOAc−PE to give pyoluteorin (**20**) (240 mg, 89% yield). ^{1}H NMR (400 MHz, methanol-d_4) δ 7.08 (t, J = 8.2 Hz, 1H), 6.55 (d, J = 1.8 Hz, 1H), 6.37 (d, J = 8.2 Hz, 2H); ESIMS *m/z* 272 $[M + H]^+$. The spectroscopic data corresponds to previously reported data.[21]

Synthetic procedures for chimedermycins I−N (15 and 21−25)

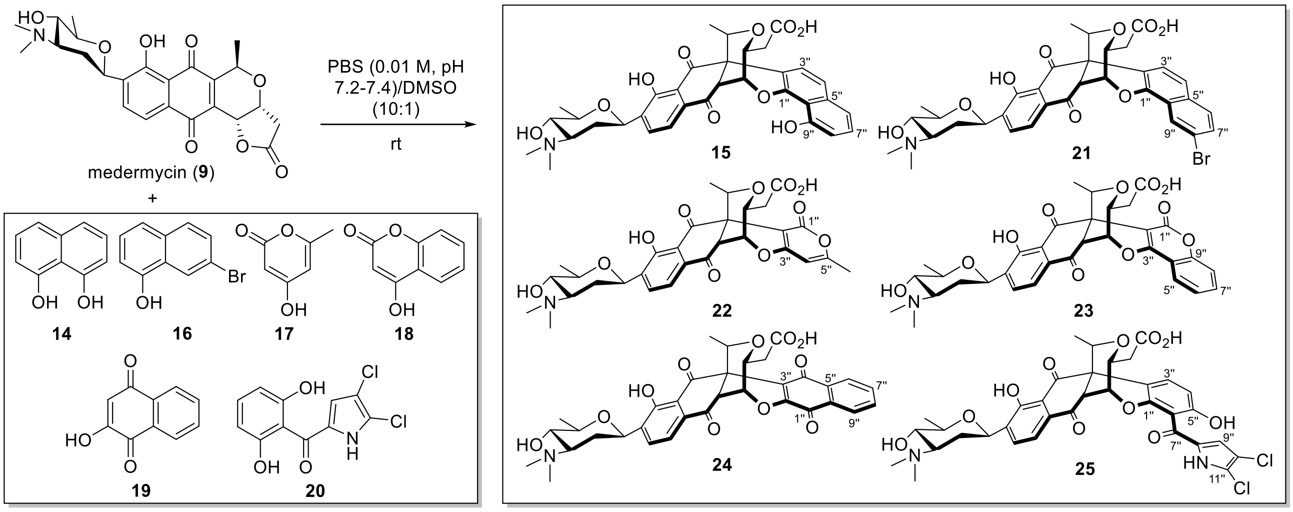

Chimedermycin I (15): Medermycin (**9**, 2.5 mg, 5.5 μmol) and naphthalene-1,8-diol (**14**, 5.0 mg, 31.3 μmol) were dissolved in DMSO (100 μL). Then, 1.0 mL of PBS (0.01 M, pH 7.2−7.4) was added. The reaction was allowed to proceed at rt for 144 h and detected using LC-MS (Supplementary Fig. 5). The mixture was dried *in vacuo* and purified by HPLC on a C18-PFP column (ACE C18-PFP, 10 × 250 mm, 5 μm, 3.0 mL/min) eluting with 40% MeCN (0.5‰ TFA) to yield compound **15** (2.6 mg, t_R = 8.0 min, 77% yield). $[\alpha]_D^{27}$ −227.0 (*c* 0.1, MeOH); UV (MeOH) λ_{max} (log ε) 228 (4.40), 342 (3.74); ECD (0.81 mM, MeOH) λ_{max} ($\Delta\varepsilon$) 226 (+61.1), 240 (−51.1), 290 (+8.3), 342 (−20.9) nm; IR (KBr) ν_{max} 3443, 2981, 1683, 1647, 1578, 1519, 1391, 1252, 1204, 1133, 830, 722, 671 cm^{-1}; ^{1}H and ^{13}C NMR, see Supplementary Table 4; HRESIMS *m/z* 618.2329 [M + H]$^+$ (calcd for $C_{34}H_{36}O_{10}N$, 618.2334).

Chimedermycin J (21): Medermycin (**9**, 7.0 mg, 15.3 μmol) and 7-bromonaphthalen-1-ol (**16**, 7.1 mg, 32.0 μmol) were dissolved in DMSO (200 μL). Then, 2.0 mL of PBS (0.01 M, pH 7.2−7.4) was added. The reaction was allowed to proceed at rt for 72 h and detected using LC-MS (Supplementary Fig. 5). The mixture was dried *in vacuo* and purified by HPLC on a Phenyl column (YMC-Pack Ph, 10 × 250 mm, 5 μm, 3.0 mL/min) using a step gradient solvent system from 35% to 90% MeCN over 22 min to afford compound **21** (6.0 mg, t_R = 20.5 min, 58% yield). $[\alpha]_D^{26}$ −481.3 (*c* 0.01, MeOH); UV (MeOH) λ_{max} (log ε) 220 (4.51), 246 (4.59), 275 (4.01), 356 (3.80); ECD (0.74 mM, MeOH) λ_{max} ($\Delta\varepsilon$) 231 (+77.1), 247 (−70.6), 289 (+3.0), 332 (−23.8) nm; IR (KBr) ν_{max} 3423, 1690, 1639, 1424, 1265, 1204, 1128, 1025, 836, 725 cm^{-1}; ^{1}H and ^{13}C NMR, see Supplementary Table 4; HRESIMS *m/z* 680.1509 [M + H]$^+$ (calcd for $C_{34}H_{35}O_9NBr$, 680.1490).

Chimedermycin K (22): Medermycin (**9**, 4.0 mg, 8.8 μmol) and 4-hydroxy-6-methyl-2-pyrone (**17**, 4.5 mg, 35.7 μmol) were dissolved in DMSO (100 μL). Then, 1.0 mL of PBS (0.01 M, pH 7.2−7.4) was added. The reaction was allowed to proceed at rt for 48 h and detected using LC-MS (Supplementary Fig. 5). The mixture was dried *in vacuo* and purified by HPLC on a C18-PFP column (ACE C18-PFP, 10 × 250 mm, 5 μm, 3.0 mL/min) eluting with 40% MeCN (0.5‰ TFA) to yield compound **22** (3.3 mg, t_R = 6.5 min, 65% yield). $[\alpha]_D^{27}$ −92.4 (*c* 0.1, MeOH); UV (MeOH) λ_{max} (log ε) 201 (4.24), 233 (4.07), 277 (3.73), 351 (3.53) nm; ECD (0.86 mM, MeOH) λ_{max} ($\Delta\varepsilon$) 207 (−37.9), 299 (+4.5), 344 (−2.3) nm; IR (KBr) ν_{max} 3446, 2930, 1697, 1563, 1418, 1263, 1206, 1139, 802, 723 cm^{-1}; ^{1}H and ^{13}C NMR, see Supplementary Table 4; HRESIMS *m/z* 584.2123 [M + H]$^+$ (calcd for $C_{30}H_{34}O_{11}N$, 584.2126).

Chimedermycin L (23): Medermycin (**9**, 5.0 mg, 10.9 μmol) and 4-hydroxycoumarin (**18**, 4.0 mg, 24.7 μmol) were dissolved in DMSO (100 μL). Then, 1.0 mL of PBS (0.01 M, pH 7.2−7.4) was added. The reaction was allowed to proceed at rt for 120 h and detected using LC-MS (Supplementary Fig. 5). The mixture was dried *in vacuo* and purified by HPLC on a C18-PFP column (ACE C18-PFP, 10 × 250 mm, 5 μm, 3.0 mL/min) eluting with 35% MeCN

(0.5‰ TFA) to yield compound **23** (4.2 mg, t_R = 8.5 min, 62% yield). $[\alpha]_D^{27}$ −60.1 (*c* 0.1, MeOH); UV (MeOH) λ_{max} (log ε) 201 (4.48), 233 (4.30), 271 (4.01), 308 (3.82), 351 (3.70) nm; ECD (0.81 mM, MeOH) λ_{max} ($\Delta\varepsilon$) 230 (−45.0), 242 (+21.5), 271 (−28.9), 308 (+9.9), 342 (−7.9) nm; IR (KBr) ν_{max} 3424, 2924, 1698, 1615, 1403, 1265, 1203, 1128, 1013, 765 cm^{-1}; ^1H and ^{13}C NMR, see Supplementary Table 5; HRESIMS *m/z* 620.2129 $[M + H]^+$ (calcd for $C_{33}H_{34}O_{11}N$, 620.2126).

Chimedermycin M (24): Medermycin (**9**, 5.0 mg, 10.9 μmol) and 2-hydroxy-1,4-naphoquinone (**19**, 4.0 mg, 23.0 μmol) were dissolved in DMSO (100 μL). Then, 1.0 mL of PBS (0.01 M, pH 7.2−7.4) was added. The reaction was allowed to proceed at rt for 120 h and detected using LC-MS (Supplementary Fig. 5). The mixture was dried *in vacuo* and purified by HPLC on a C18-PFP column (ACE C18-PFP, 10 × 250 mm, 5 μm, 3.0 mL/min) eluting with 35% MeCN (0.5‰ TFA) to yield compound **24** (4.6 mg, t_R = 8.0 min, 67% yield). $[\alpha]_D^{27}$ −307.2 (*c* 0.1, MeOH); UV (MeOH) λ_{max} (log ε) 239 (4.16), 347 (3.59) nm; ECD (0.79 mM, MeOH) λ_{max} ($\Delta\varepsilon$) 210 (+5.4), 227 (−11.8), 248 (+12.3), 286 (−15.4) nm; IR (KBr) ν_{max} 3426, 2936, 1683, 1429, 1384, 1269, 1207, 1136, 798, 729 cm^{-1}; ^1H and ^{13}C NMR, see Supplementary Table 5; HRESIMS *m/z* 632.2126 $[M + H]^+$ (calcd for $C_{34}H_{34}O_{11}N$, 632.2126).

Chimedermycin N (25): Medermycin (**9**, 3.0 mg, 6.6 μmol) and pyoluteorin (**20**, 4.2 mg, 15.6 μmol) were dissolved in DMSO (100 μL). Then, 1.0 mL of PBS (0.01 M, pH 7.2−7.4) was added. The reaction was allowed to proceed at rt for 240 h and detected using LC-MS (Supplementary Fig. 5). The mixture was dried *in vacuo* and purified by HPLC on a C18-PFP column (ACE C18-PFP, 10 × 250 mm, 5 μm, 3.0 mL/min) eluting with 35% MeCN (0.5‰ TFA) to yield compound **25** (2.8 mg, t_R = 9.0 min, 59% yield). $[\alpha]_D^{27}$ −290.4 (*c* 0.1, MeOH); UV (MeOH) λ_{max} (log ε) 201 (4.60), 235 (4.26), 306 (4.10) nm; ECD (0.69 mM, MeOH) λ_{max} ($\Delta\varepsilon$) 251 (−5.2), 314 (+0.7), 355 (−15.3) nm; IR (KBr) ν_{max} 3435, 2927, 1682, 1637, 1443, 1396, 1269, 1205, 1130, 1080, 841, 804, 723 cm^{-1}; ^1H and ^{13}C NMR, see Supplementary Table 5; HRESIMS *m/z* 729.1621 $[M + H]^+$ (calcd for $C_{35}H_{35}O_{11}N_2Cl_2$, 729.1612).

Synthetic procedure for sekgranaticin B (27)

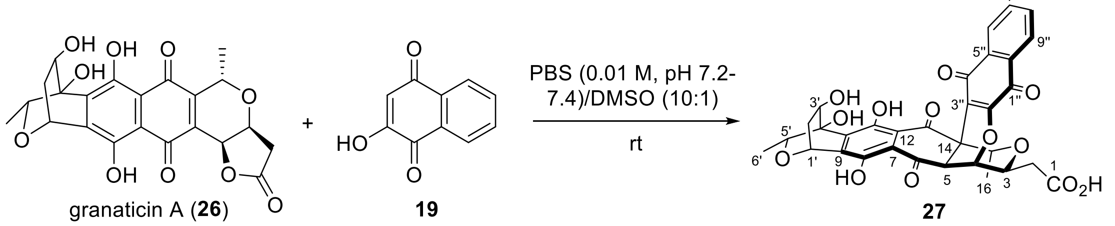

Granaticin A (**26**, 100.0 mg, 225.2 μmol), that was isolated from the fermentation broth of the *Streptomyces* sp. 166#,[22] and 2-hydroxy-1,4-naphoquinone (**19**, 80.0 mg, 459.8 μmol) were dissolved in DMSO (2 mL). Then, 20 mL of PBS (0.01 M, pH 7.2−7.4) was added. The reaction was allowed to proceed at room temperature for 168 h. The mixture was dried *in vacuo* and purified by HPLC on a Phenyl column (YMC-Pack Ph, 10 × 250 mm, 5 μm, 4.0 mL/min) eluting with 30% MeCN (0.5‰ TFA) to yield sekgranaticin B (**27**) (117.0 mg, t_R = 20.5 min, 84% yield). $[\alpha]_D^{26}$ +179.0 (*c* 0.05, MeOH); ^1H NMR (500 MHz, DMSO-d_6) δ 11.69 (s, 1H, 8-OH), 11.40 (s, 1H, 11-OH), 7.94 (dd, *J* = 5.8, 3.4 Hz, 1H, H-9″), 7.81 (dd, *J* = 5.6, 3.5 Hz, 1H, H-6″), 7.77 (m, 2H, H-7″&8″), 5.32 (s, 1H, H-4), 4.96 (s, 1H, H-1′), 4.54 (d, *J* = 1.5 Hz, 1H, H-5), 4.35 (dd, *J* = 8.9, 4.1 Hz, 1H, H-3), 4.25 (q, *J* = 6.5 Hz, 1H, H-15), 3.79 (d, *J* = 8.1 Hz, 1H, H-3′), 3.64 (q, *J* = 6.2 Hz, 1H, H-5′), 2.81 (dd, *J* = 16.2, 4.2 Hz, 1H, H-2a), 2.48 (overlapped, 1H, H-2′a), 2.40 (dd, *J* = 16.2, 8.9 Hz, 1H, H-2b), 1.60 (d, *J* = 6.5 Hz, 3H, H-16), 1.20 (d, *J* = 14.2 Hz, 1H, H-2′b), 0.78 (d, *J* = 6.2 Hz, 3H, H-6′); ^{13}C NMR (125 MHz, DMSO-d_6) δ 197.3 (C-6), 197.0 (C-13), 181.5 (C-4″), 177.3 (C-1″), 171.5 (C-1), 157.1 (C-2″), 151.9 (C-11), 147.1 (C-8), 135.3 (C-9), 134.9 (C-10), 134.7 (C-7″), 133.8 (C-8″), 131.0 (C-5″), 130.3 (C-10″), 126.1 (C-6″), 125.9 (C-9″), 119.9 (C-3″), 114.8 (C-12), 112.5 (C-7), 80.1 (C-4′), 72.0 (C-15), 71.4 (C-5′), 70.5 (C-4), 70.4 (C-3), 69.9 (C-3′), 61.3 (C-1′), 48.7 (C-14), 42.8 (C-5), 36.6 (C-2′), 36.3 (C-2), 16.7 (C-6′), 14.8 (C-16); HRESIMS *m/z* 619.1448 $[M + H]^+$ (calcd for $C_{32}H_{27}O_{13}$, 619.1446), 636.1719 $[M + NH_4]^+$ (calcd

for $C_{32}H_{30}O_{13}N$, 636.1712).

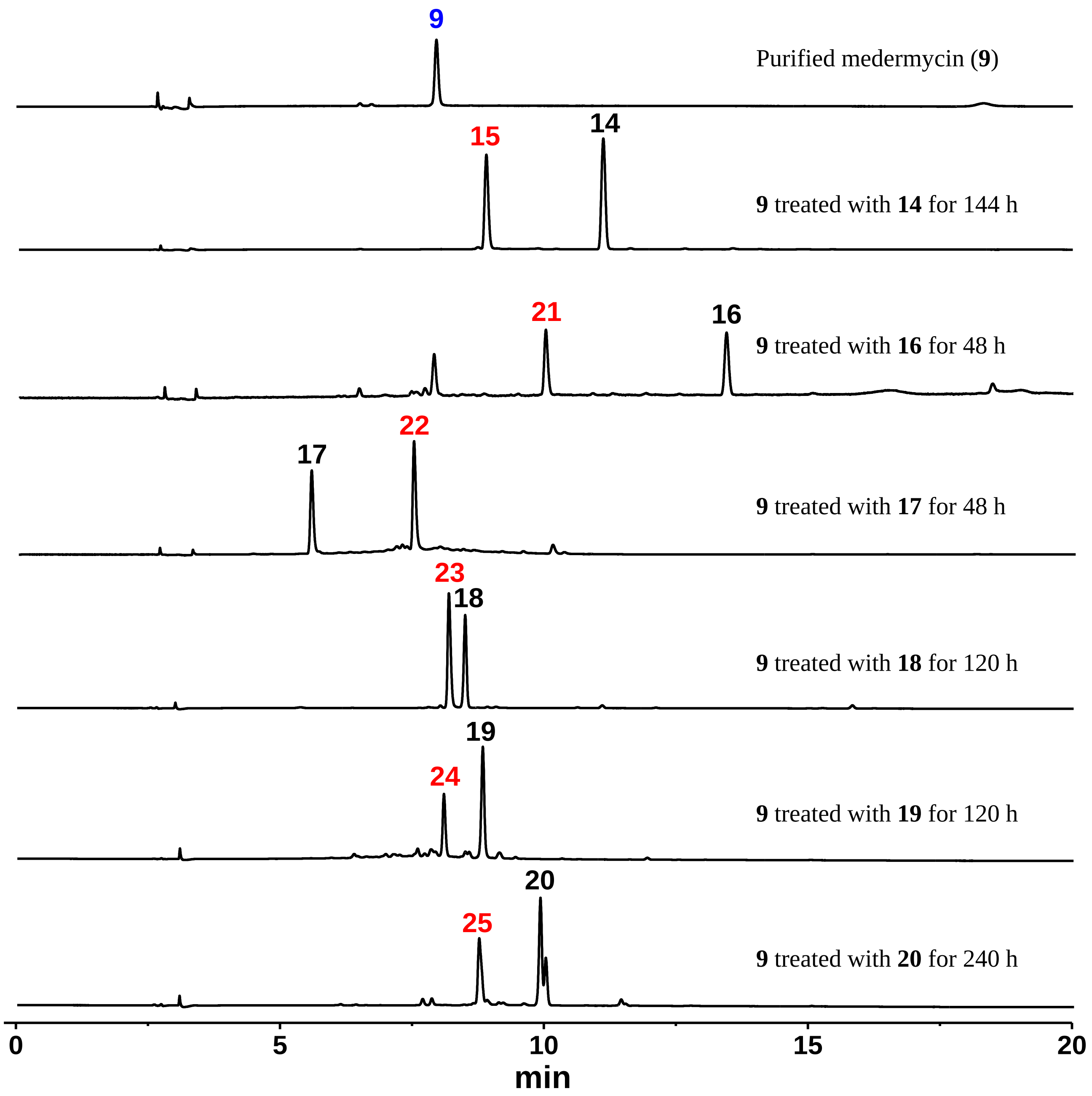

Supplementary Fig. 5. HPLC traces (280 nm) for syntheses of chimedermycins I−N (**15** and **21**−**25**).

Supplementary Table 4. ^{1}H and ^{13}C NMR data for chimedermycins I−K (**15**, **21**, and **22**) in methanol-d_4

No.	**15**[a]		**21**[b]		**22**[c]	
	δ_C	δ_H, mult. (J in Hz)	δ_C	δ_H, mult. (J in Hz)	δ_C	δ_H, mult. (J in Hz)
1	174.1, C		174.3, C		173.9, C	
2	37.9, CH2	2.88, dd (15.8, 4.9);	37.8, CH2	2.87, dd (16.1, 4.9);	37.3, CH2	2.75, dd (16.2, 5.0);
		2.62, dd (15.8, 8.4)		2.59, dd (16.1, 8.3)		2.56, dd (16.3, 8.3)
3	72.1, CH	4.51, dd (8.3, 5.0)	72.4, CH	4.50, dd (8.5, 4.9)	71.2, CH	4.41, dd (8.4, 5.5)
4	71.1, CH	5.39, s	70.5, CH	5.28, s	72.7, CH	5.22, d (1.9)
5	43.8, CH	4.25, d (1.7)	44.4, CH	4.21, d (1.9)	45.0, CH	4.11, d (1.9)
6	192.2, C		192.2, C		191.7, C	
7	133.7, C		133.8, C		133.1, C	
8	119.2, CH	7.52, d (7.9)	119.2, CH	7.47, d (7.9)	118.8, CH	7.53, d (8.0)
9	135.4, CH	7.87, d (7.9)	135.5, CH	7.85, d (7.9)	134.0, CH	7.85, d (8.0)
10	137.9, C		137.8, C		137.6, C	
11	159.2, C		159.2, C		157.4, C	
12	117.4, C		117.4, C		118.2, C	
13	204.1, C		204.3, C		202.8, C	
14	52.9, C		53.2, C		49.8, C	
15	76.2, CH	4.43, q (6.6)	76.3, CH	4.41, q (6.6)	73.7, CH	4.30, q (6.6)
16	14.5, CH3	1.61, d (6.6)	14.6, CH3	1.61, d (6.5)	14.7, CH3	1.63, d (6.6)
1′	72.3, CH	5.02, d (10.8)	72.3, CH	5.01, dd (10.7, 2.0)	72.5, CH	5.01, d (10.8)
2′	30.1, CH2	2.51, m;	30.1, CH2	2.50, m;	30.2, CH2	2.53, m;
		1.61, overlapped		1.60, overlapped		1.60, overlapped
3′	68.5, CH	3.60, m	68.5, CH	3.60, m	68.5, CH	3.60, m
4′	71.2, CH	3.42, dd (10.0, 8.9)	71.2, CH	3.41, dd (10.0, 8.9)	71.2, CH	3.44, dd (10.2, 8.7)
5′	78.2, CH	3.57, m	78.5, CH	3.56, m	78.5, CH	3.57, m
6′	18.2, CH3	1.39, d (6.1)	18.2, CH3	1.39, d (6.2)	18.2, CH3	1.41, d (6.1)
7′	42.2, CH3	2.91, s	42.2, CH3	2.91, s	42.1, CH3	2.92, s
8′	37.2, CH3	2.75, s	37.3, CH3	2.75, s	37.4, CH3	2.79, s
1″	152.0, C		150.2, C		164.6, C	
2″	115.6, C		117.3, C		100.5, C	
3″	123.2, CH	6.92, d (8.8)	123.9, CH	7.01, d (8.7)	169.5, C	
4″	122.6, CH	7.22, d (8.8)	121.2, CH	7.24, d (8.8)	101.1, CH	6.07, s
5″	137.8, C		133.8, C		164.0, C	
6″	119.9, CH	7.18, d (7.9)	130.5, CH	7.63, d (8.8)	19.5, CH3	2.13, s
7″	129.4, CH	7.31, t (7.9)	131.4, CH	7.56, dd (8.8, 1.9)		
8″	112.2, CH	6.83, d (7.7)	121.1, C			
9″	155.7, C		125.1, CH	8.34, d (1.9)		
10″	115.2, C		126.7, C			

[a] Recorded at 600 MHz for ^{1}H and 150 MHz for ^{13}C. [b] Recorded at 500 MHz for ^{1}H and 150 MHz for ^{13}C. [c] Recorded at 500 MHz for ^{1}H and 125 MHz for ^{13}C.

Supplementary Table 5. ^{1}H (500 MHz) and ^{13}C (125 MHz) NMR data for chimedermycins L−N (**23**−**25**) in methanol-d_4

No.	**23**		**24**		**25**	
	δ_C	δ_H, mult. (J in Hz)	δ_C	δ_H, mult. (J in Hz)	δ_C	δ_H, mult. (J in Hz)
1	173.8, C		173.9, C		174.3, C	
2	37.5, CH_2	2.85, dd (16.2, 4.9);	37.3, CH_2	2.82, dd (16.3, 4.7);	37.2, CH_2	2.59, dd (16.4, 4.9);
		2.60, dd (16.2, 8.4)		2.60, dd (16.3, 8.5)		2.48, dd (16.5, 8.4)
3	71.4, CH	4.52, dd (8.3, 4.9)	71.9, CH	4.49, dd (8.4, 4.8)	72.0, CH	4.33, m
4	73.3, CH	5.47, s	73.0, CH	5.37, s	70.1, CH	4.91, s
5	45.1, CH	4.24, d (1.8)	45.1, CH	4.19, d (1.7)	44.5, CH	4.06, d (1.8)
6	191.5, C		191.3, C		192.4, C	
7	133.0, C		133.1, C		134.0, C	
8	118.9, CH	7.52, d (8.0)	119.1, CH	7.54, d (8.0)	119.1, CH	7.55, d (7.9)
9	134.0, CH	7.83, d (8.0)	134.2, CH	7.82, d (8.0)	135.4, CH	7.91, d (7.9)
10	137.7, C		137.5, C		137.7, C	
11	157.3, C		157.5, C		159.3, C	
12	118.4, C		118.9, C		117.2, C	
13	202.4, C		202.1, C		204.1, C	
14	50.4, C		50.7, C		52.6, C	
15	73.7, CH	4.39, q (6.6)	74.0, CH	4.38, q (6.6)	77.0, CH	4.35, q (6.6)
16	14.8, CH_3	1.70, d (6.6)	15.1, CH_3	1.70, d (6.5)	14.5, CH_3	1.54, d (6.6)
1′	72.4, CH	5.02, d (10.3)	72.4, CH	5.03, d (10.6)	72.3, CH	5.03, d (10.8)
2′	30.2, CH_2	2.53, m; 1.62, m	30.2, CH_2	2.49, m; 1.65, m	30.2, CH_2	2.53, m; 1.66, m
3′	68.5, CH	3.61, m	68.6, CH	3.62, m	68.6, CH	3.61, m
4′	71.2, CH	3.43, dd (9.9, 8.9)	71.2, CH	3.42, dd (10.1, 8.8)	71.2, CH	3.45, dd (10.1, 9.0)
5′	78.5, CH	3.57, m	78.5, CH	3.57, m	78.6, CH	3.57, m
6′	18.2, CH_3	1.40, d (6.1)	18.2, CH_3	1.39, d (6.2)	18.2, CH_3	1.41, d (6.1)
7′	42.1, CH_3	2.93, s	42.1, CH_3	2.93, s	42.2, CH_3	2.92, s
8′	37.5, CH_3	2.78, s	37.6, CH_3	2.77, s	37.3, CH_3	2.78, s
1″	162.2, C		179.0, C		153.8, C	
2″	103.2, C		158.8, C		114.3, C	
3″	164.5, C		121.7, C		128.2, CH	6.80, d (8.7)
4″	115.5, C		183.6, C		109.4, CH	6.31, d (8.7)
5″	124.3, CH	7.92, dd (8.0, 1.4)	131.7, C		157.5, C	
6″	126.0, CH	7.40, ddd (8.0, 8.0, 0.7)	127.4, CH	7.83, dd (5.9, 3.2)	115.9, C	
7″	134.6, CH	7.64, ddd (8.5, 8.5, 1.5)	135.7, CH	7.71, ddd (5.8, 5.8, 3.3)	183.8, C	
8″	117.4, CH	7.24, d (8.4)	135.0, CH	7.71, ddd (5.8, 5.8, 3.3)	131.7, C	
9″	153.7, C		127.3, CH	7.99, dd (5.9, 3.1)	119.5, CH	6.34, s
10″			133.1, C		112.1, C	
11″					122.4, C	

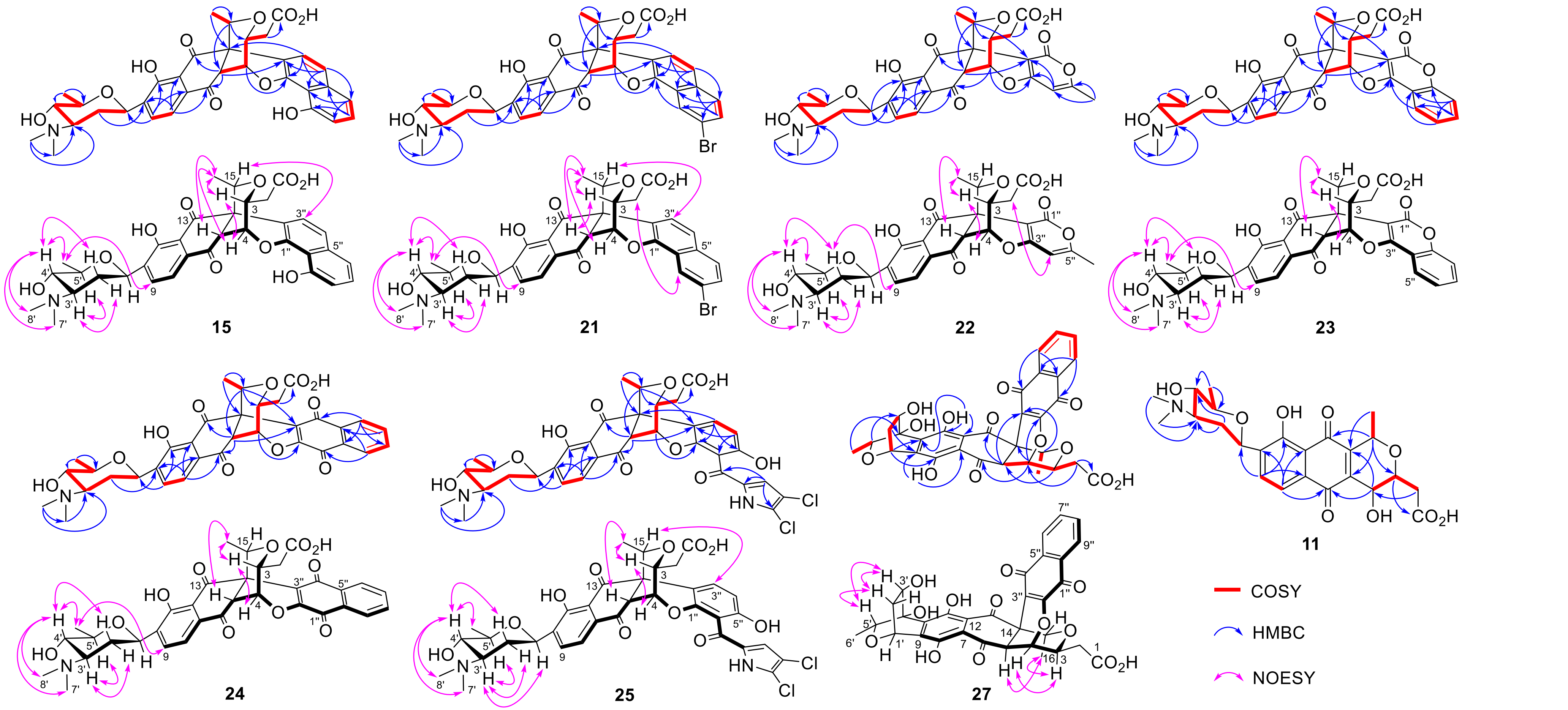

Supplementary Fig. 6. Key 2D NMR correlations of chimedermycins I−N (**15**, **21**−**25**), sekgranaticin B (**27**), and demethylmenoxymycin B (**11**).

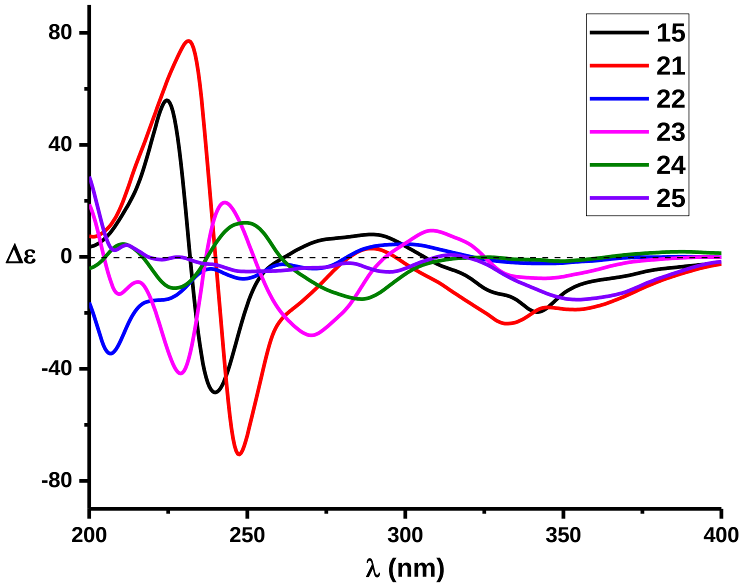

Supplementary Fig. 7. ECD curves of chimedermycins I−N (**15**, **21**−**25**).

Theory and calculation details. The calculations were performed by using the density functional theory (DFT) as carried out in the Gaussian 09.[23] Conformational searches were run by employing the "systematic" procedure implemented in Spartan'14[24] using MMFF. All MMFF minima were reoptimized with DFT calculations at the B3LYP/6-31G(d) level. Solvent effects of methanol solution were evaluated at the same DFT level by using the SCRF/PCM method.[25–27] TDDFT[28–31] at B3LYP/6-31G(d) was employed to calculate the electronic excitation energies and rotational strengths in methanol. The stable conformations obtained at the B3LYP/6-31G(d) level were further used in magnetic shielding constants at the B3LYP/6-311++G(2d,p) level. The overall calculated ECD curves were weighted by Boltzmann distribution (with a half-bandwidth of 0.30 eV). The calculated ECD spectrum were produced by SpecDis 1.70.1 software.[32]

Supplementary Fig. 8. HRESIMS spectrum of chimedermycin A (**1**)

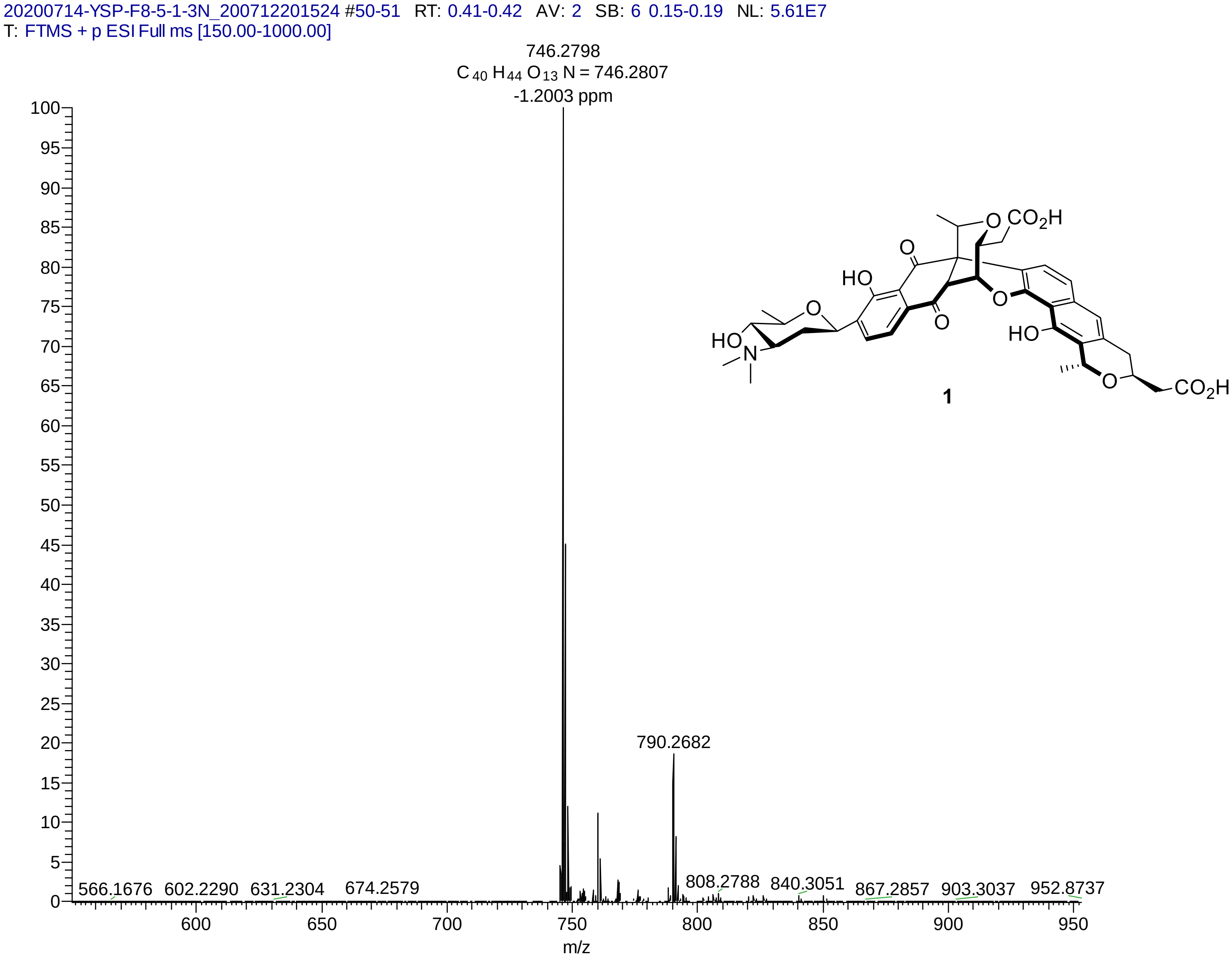

1

Supplementary Fig. 9. ^{1}H-NMR spectrum of chimedermycin A (**1**) in methanol-d_4

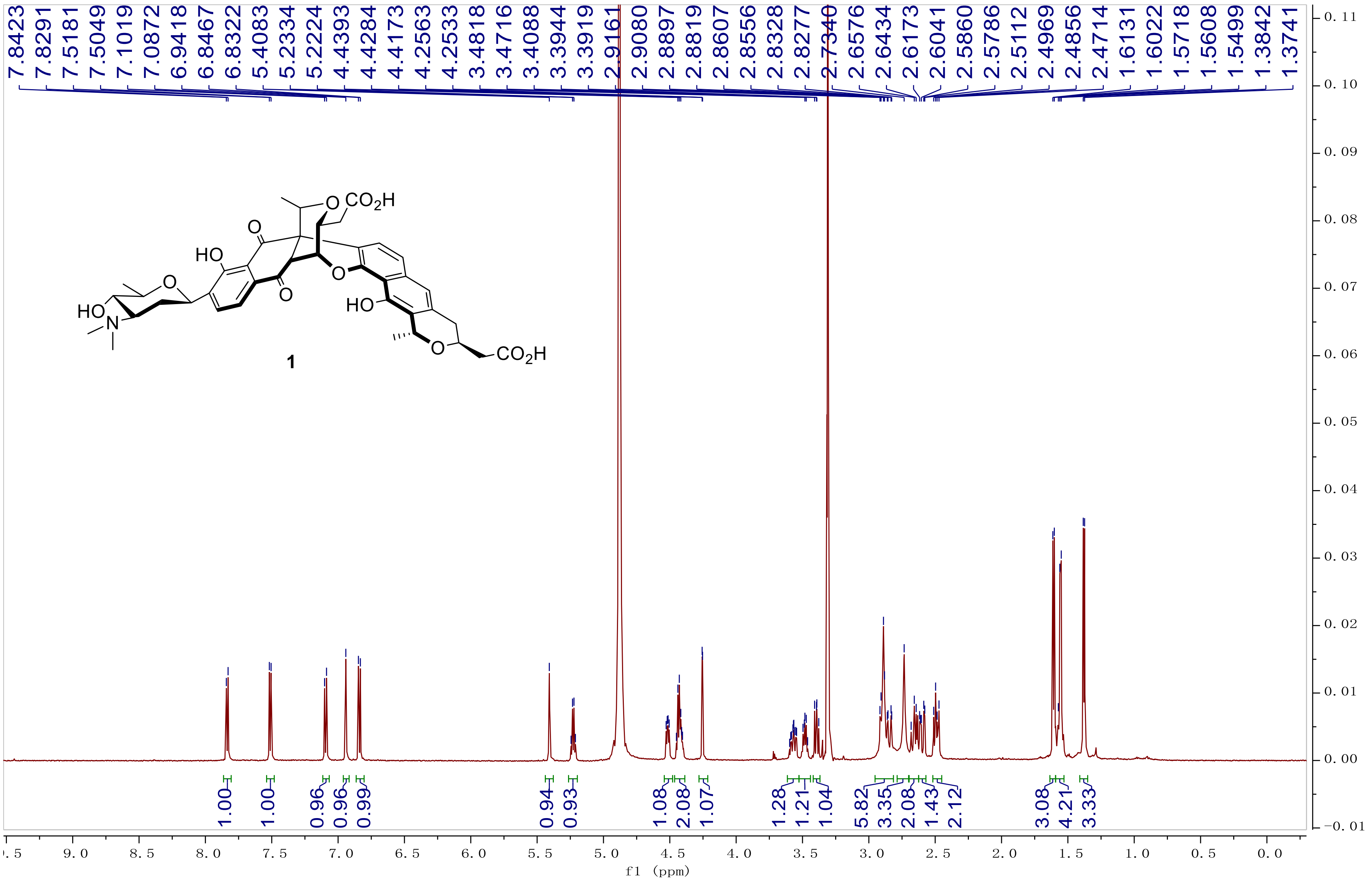

Supplementary Fig. 10. ^{13}C-NMR spectrum of chimedermycin A (**1**) in methanol-d_4

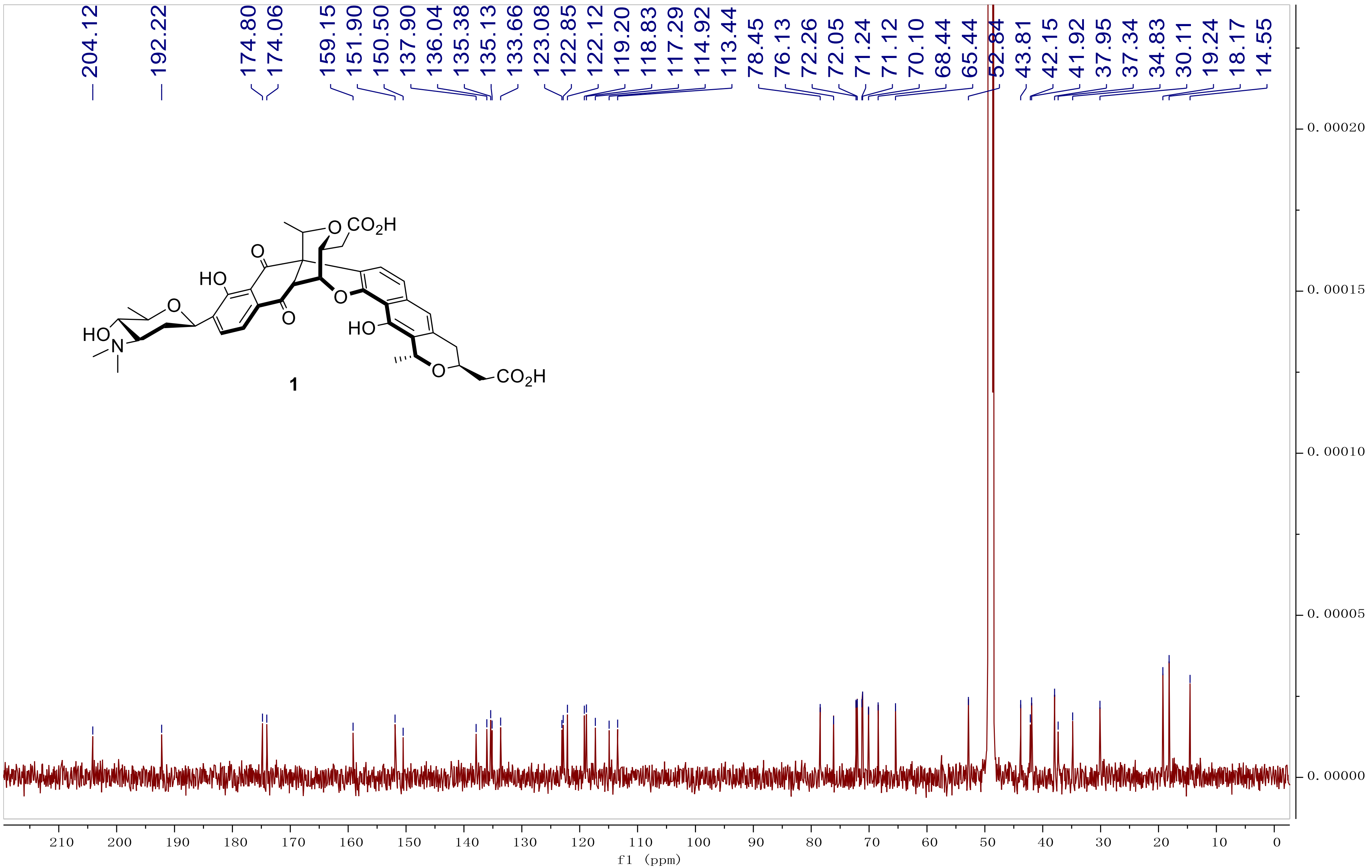

Supplementary Fig. 11. HSQC spectrum of chimedermycin A (**1**) in methanol-d_4

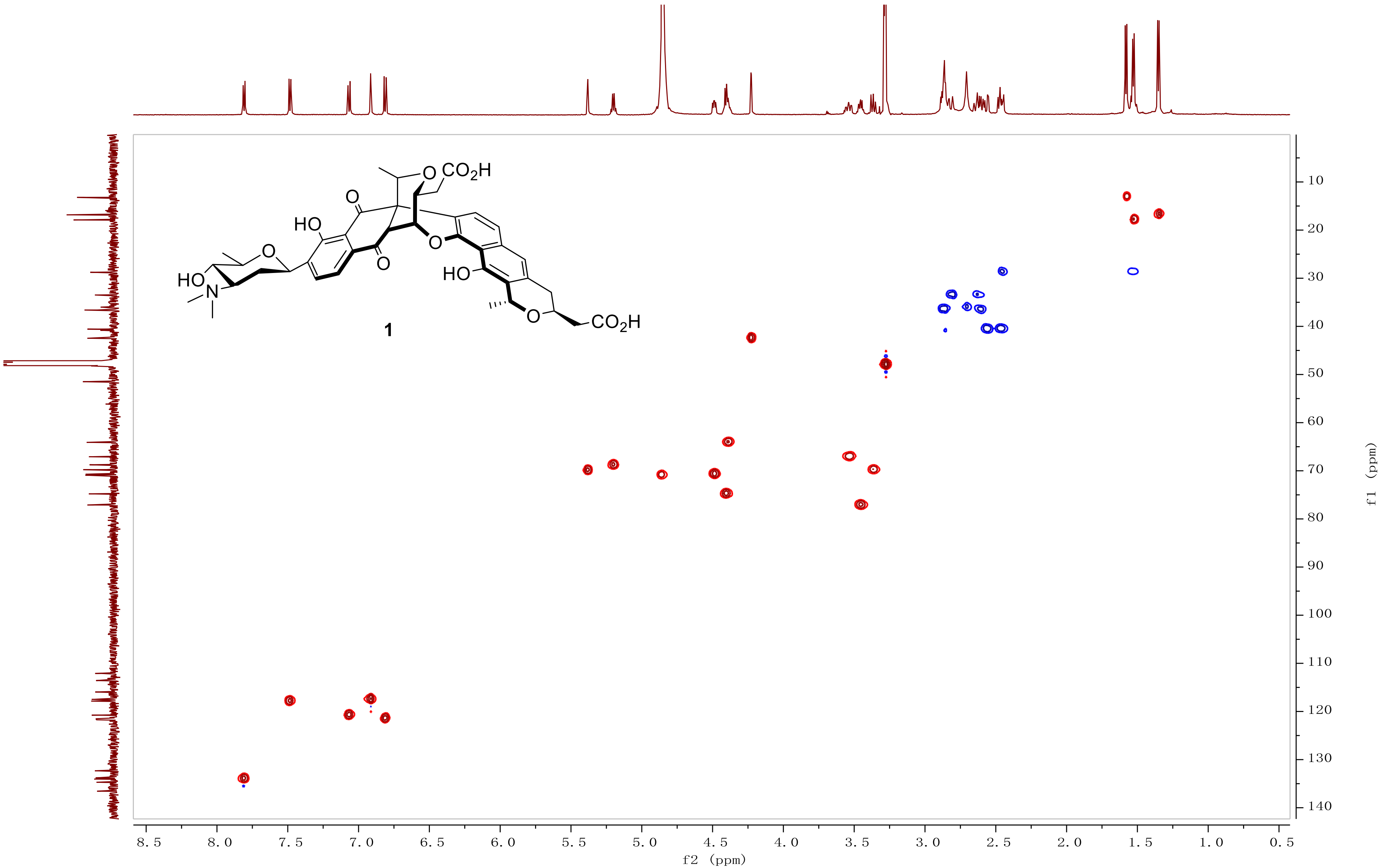

Supplementary Fig. 12. ^{1}H-^{1}H COSY spectrum of chimedermycin A (**1**) in methanol-d_4

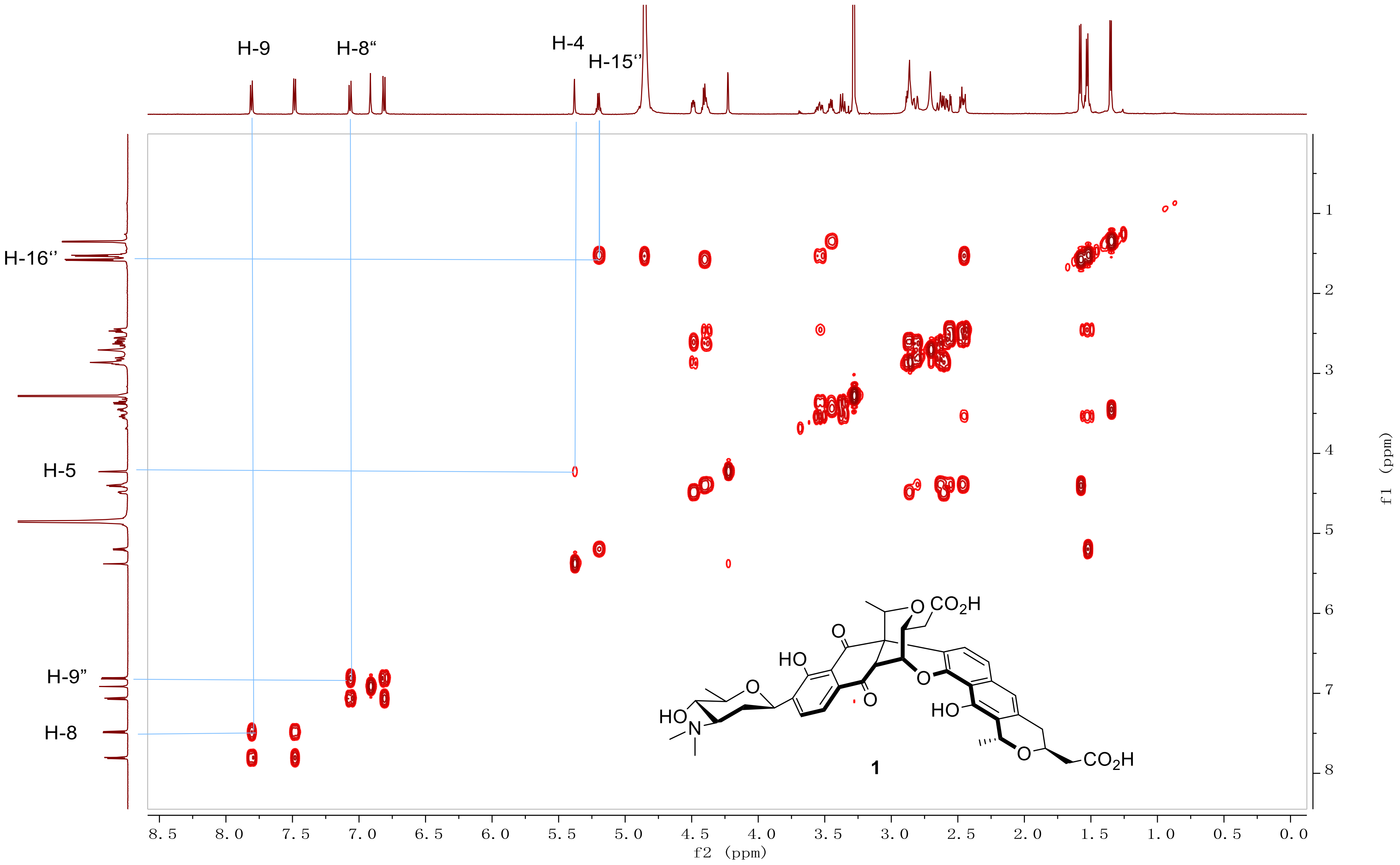

Supplementary Fig. 13. HMBC spectrum of chimedermycin A (**1**) in methanol-d_4

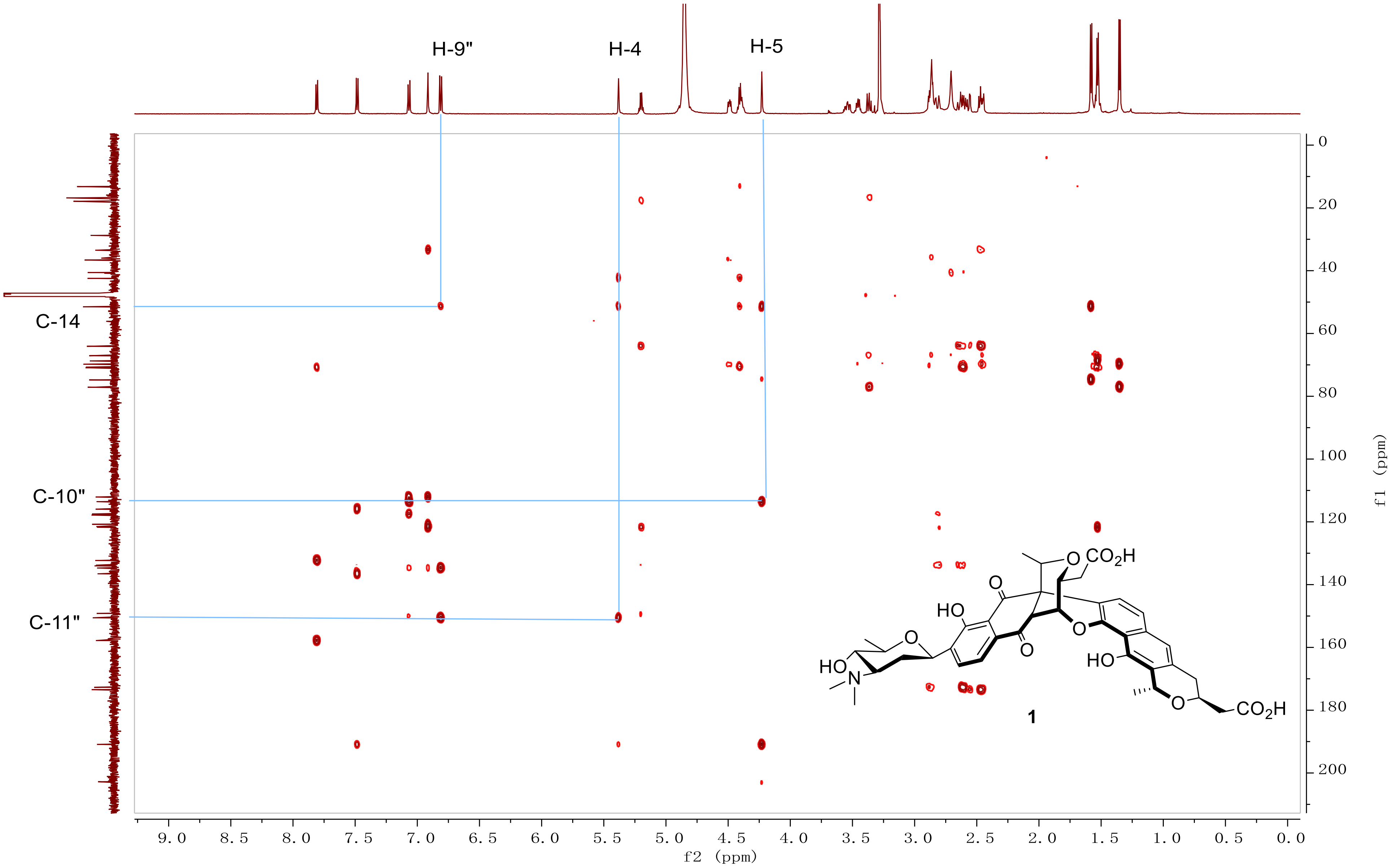

Supplementary Fig. 14. NOESY spectrum of chimedermycin A (**1**) in methanol-d_4

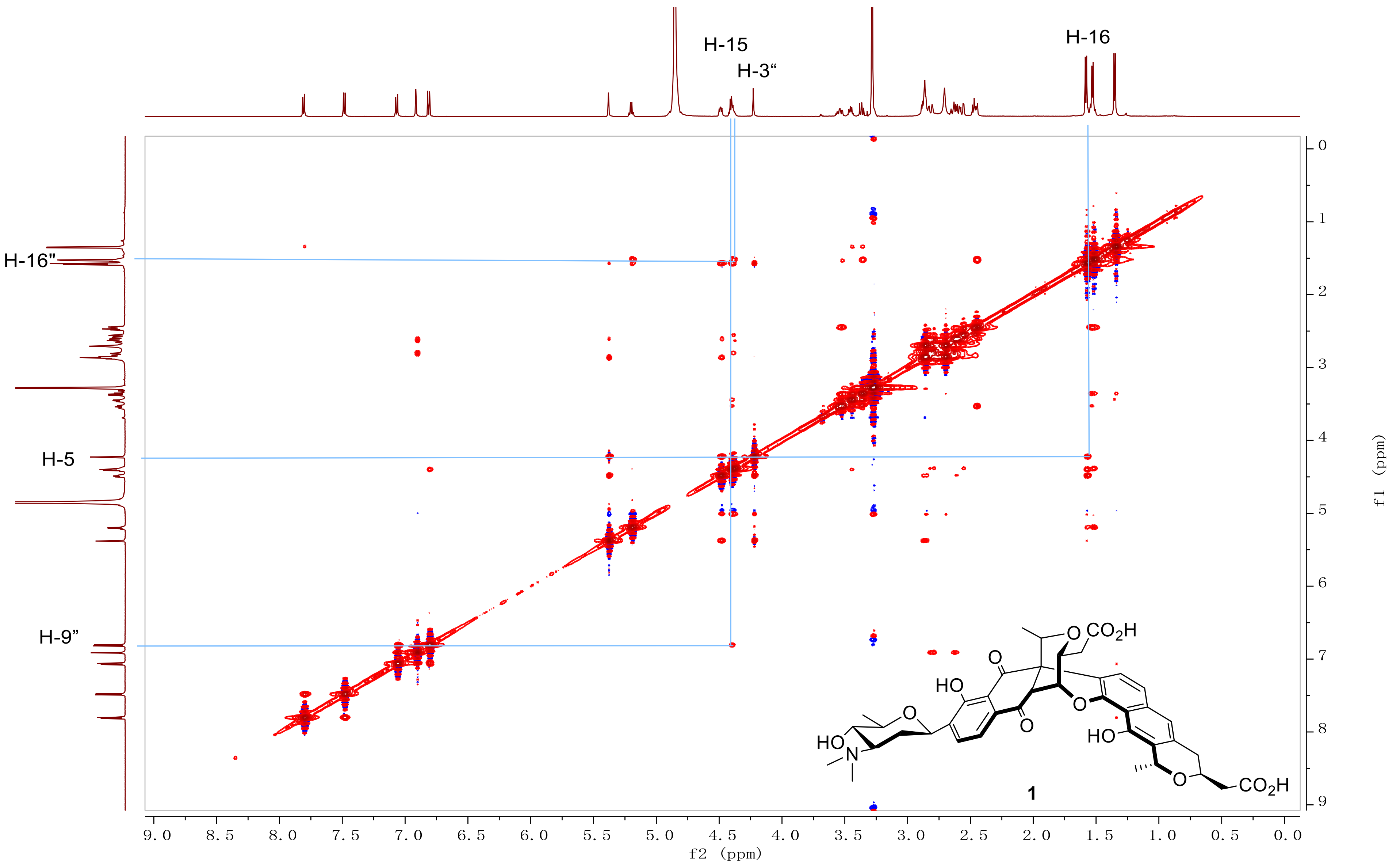

Supplementary Fig. 15. HRESIMS spectrum of chimedermycin B (**2**)

20200820-YSP-F8-6-5-2-2_200820132336 #24-25 RT: 0.19-0.20 AV: 2 NL: 4.18E7
T: FTMS + p ESI sid=35.00 Full ms [150.00-1000.00]

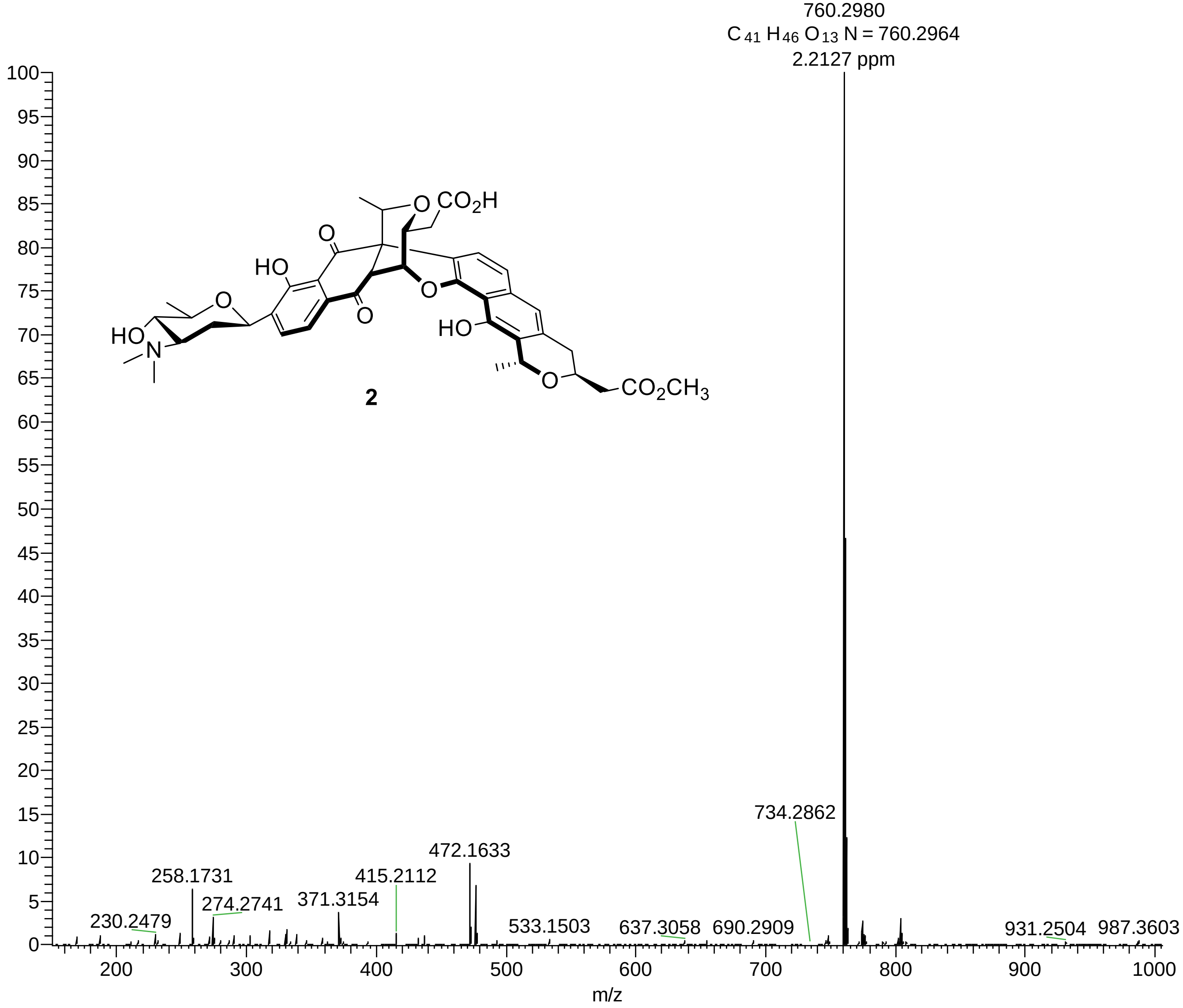

Supplementary Fig. 16. ^{1}H-NMR spectrum of chimedermycin B (**2**) in methanol-d_4

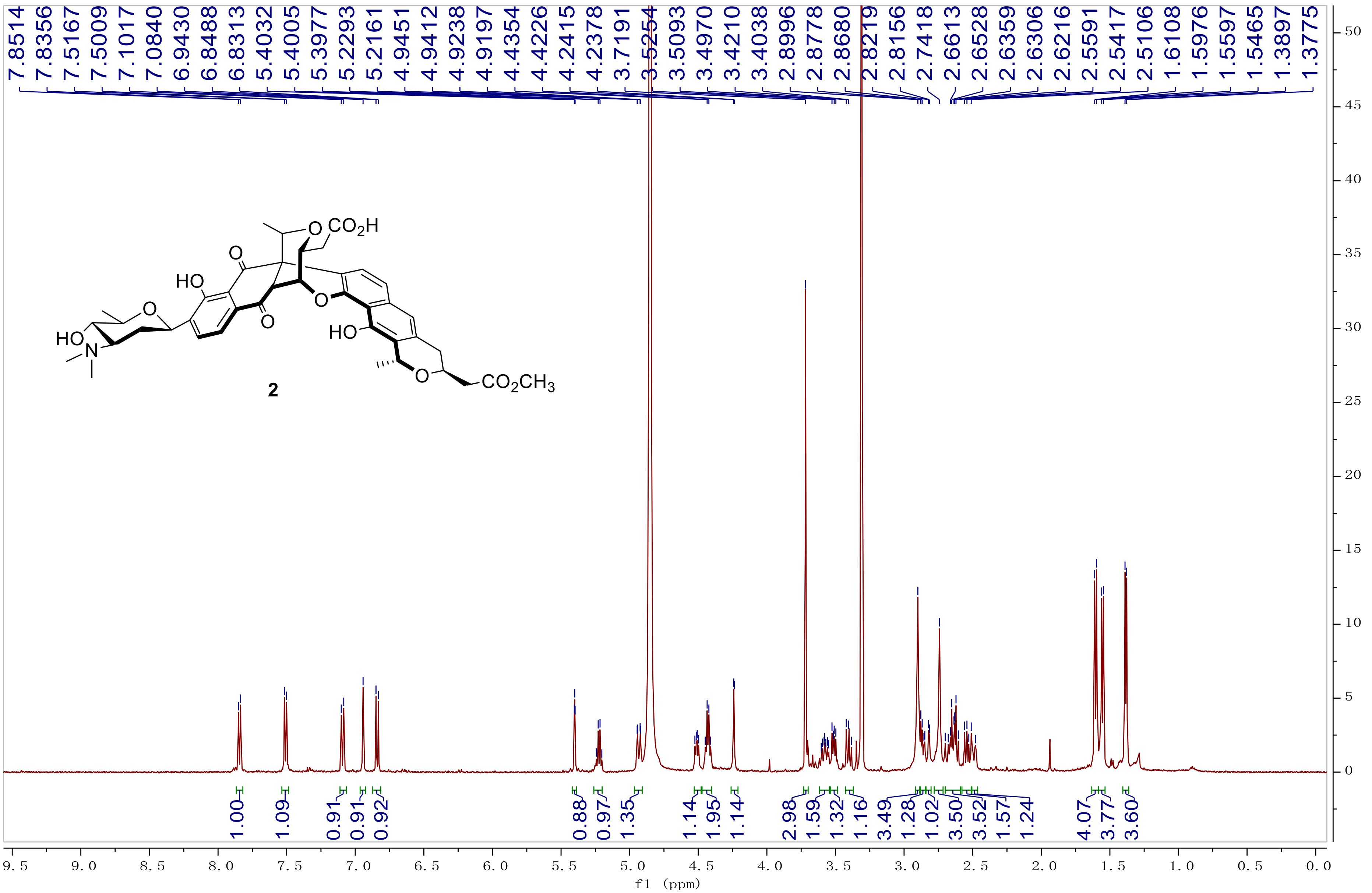

Supplementary Fig. 17. ^{13}C-NMR spectrum of chimedermycin B (**2**) in methanol-d_4

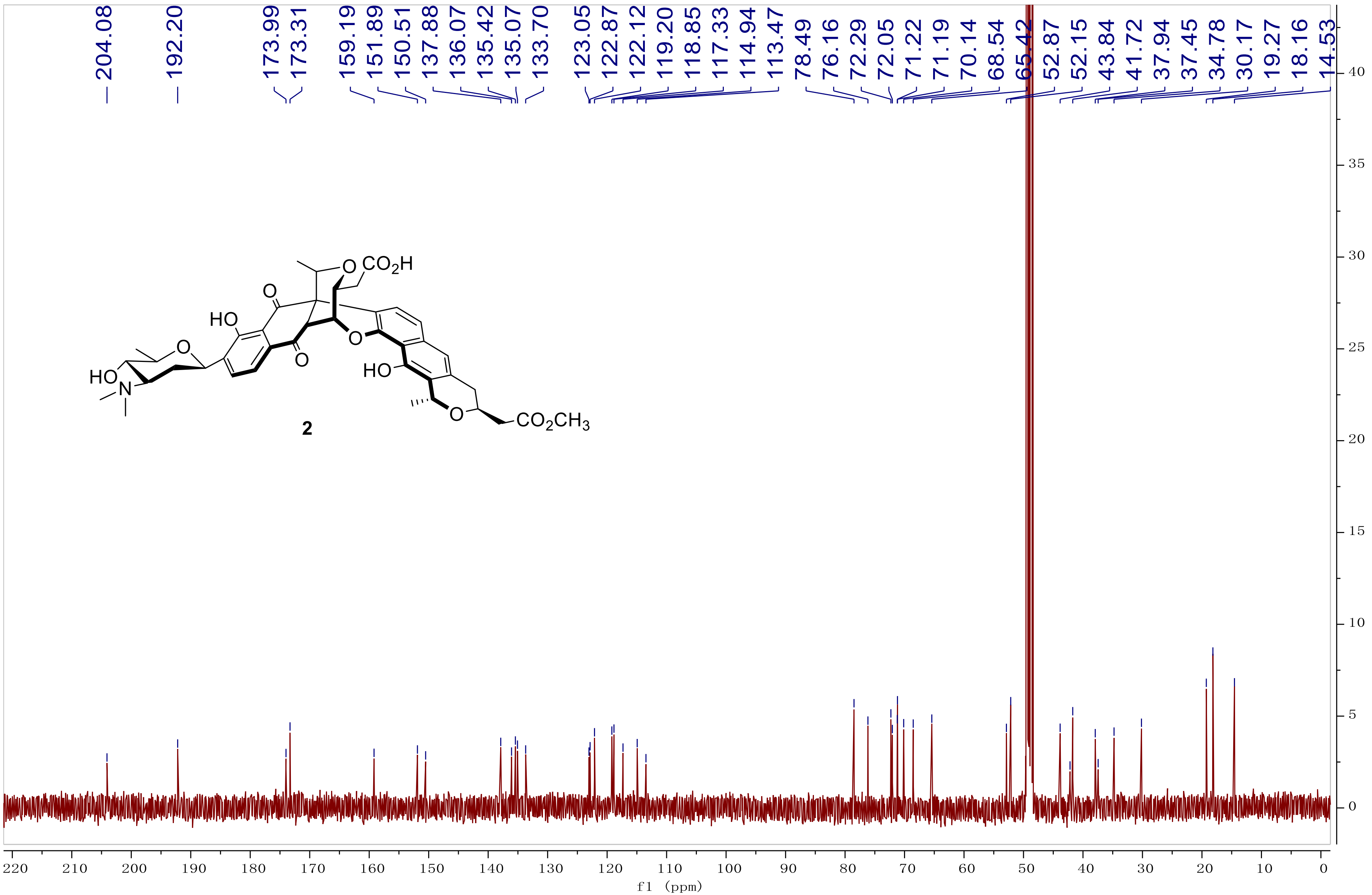

Supplementary Fig. 18. HSQC spectrum of chimedermycin B (**2**) in methanol-d_4

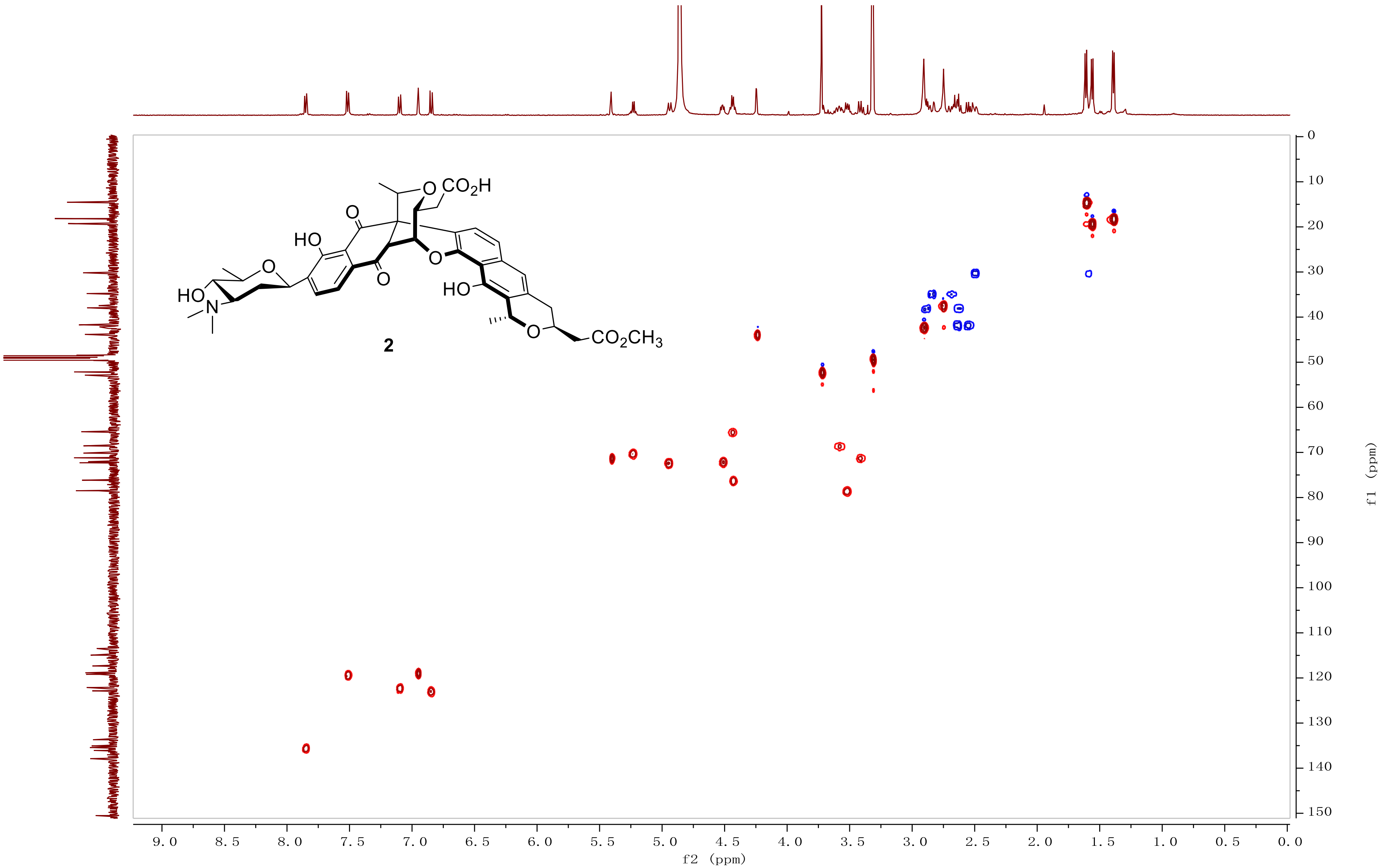

Supplementary Fig. 19. ^{1}H-^{1}H COSY spectrum of chimedermycin B (**2**) in methanol-d_4

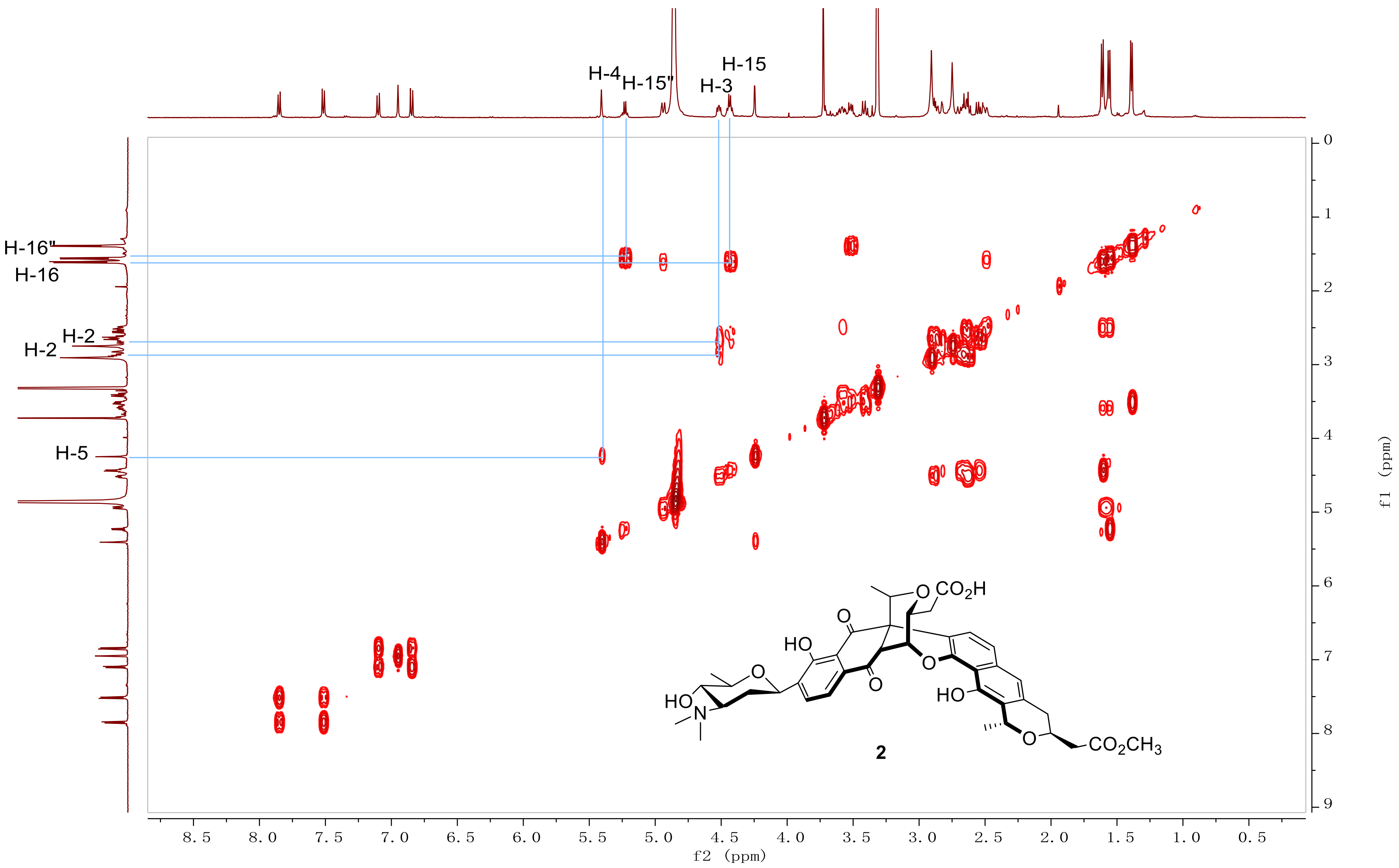

Supplementary Fig. 20. HMBC spectrum of chimedermycin B (**2**) in methanol-d_4

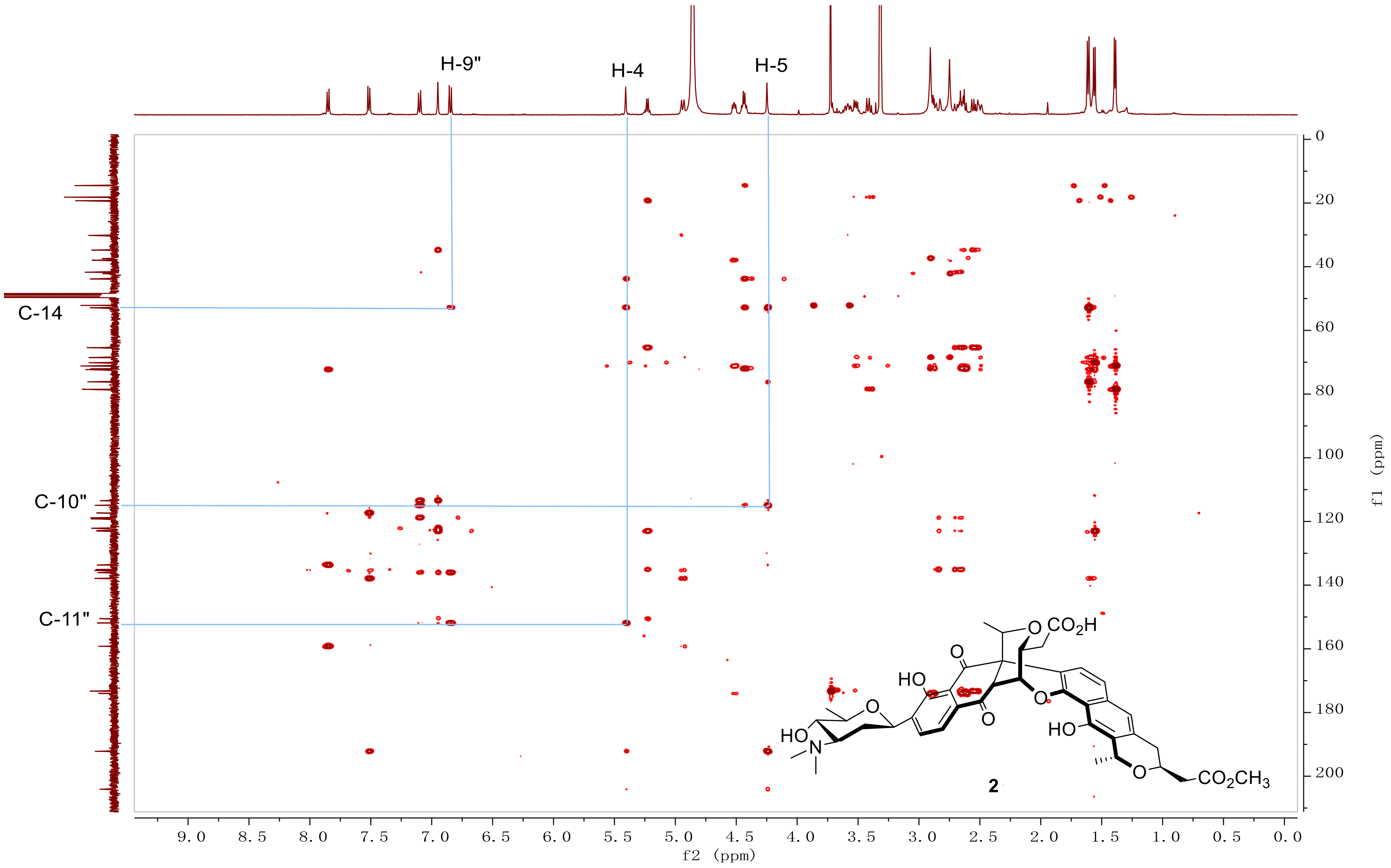

Supplementary Fig. 21. NOESY spectrum of chimedermycin B (**2**) in methanol-d_4

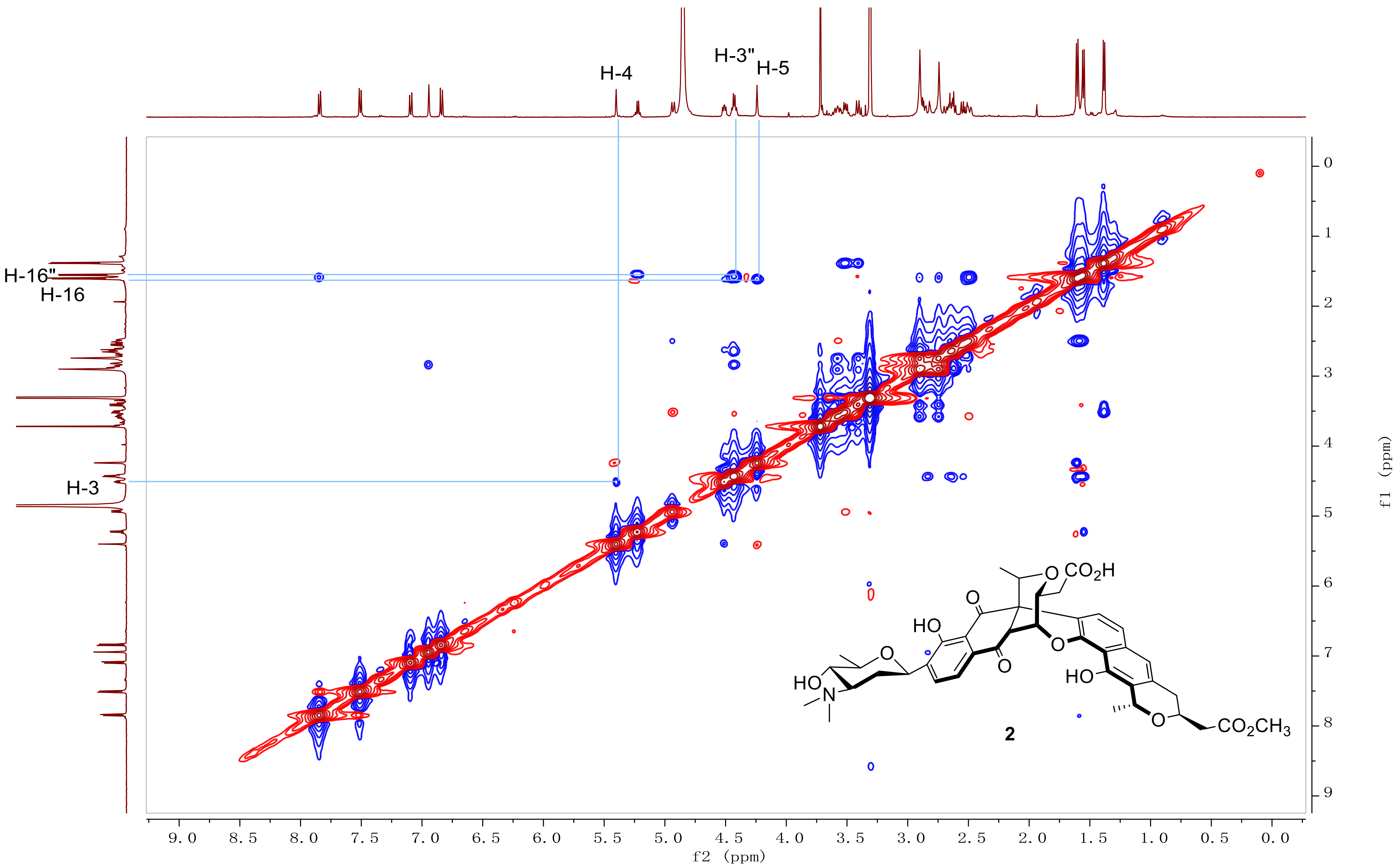

Supplementary Fig. 22. HRESIMS spectrum of chimedermycin C (**3**)

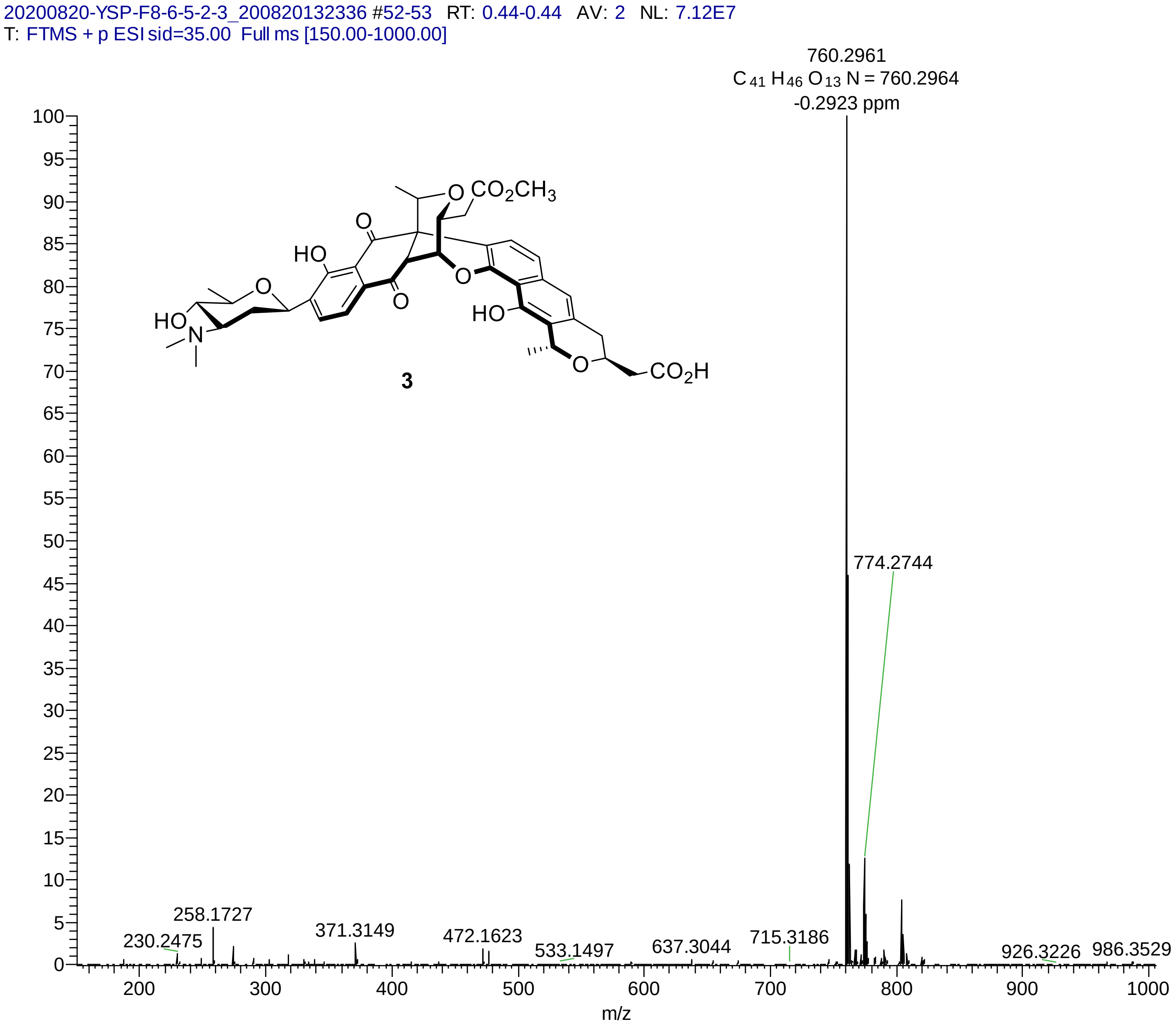

Supplementary Fig. 23. ^{1}H-NMR spectrum of chimedermycin C (**3**) in methanol-d_4

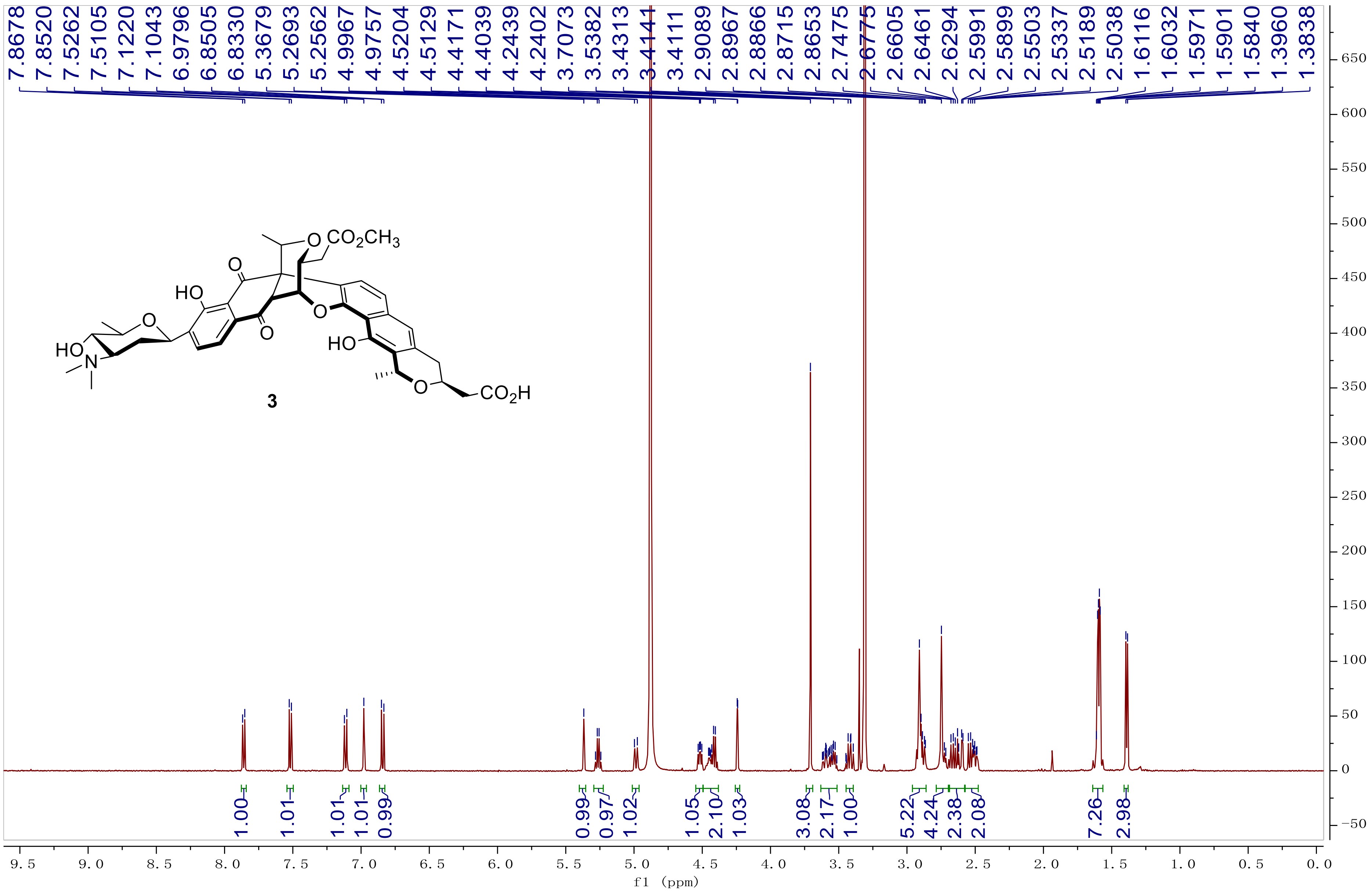

Supplementary Fig. 24. ^{13}C-NMR spectrum of chimedermycin C (**3**) in methanol-d_4

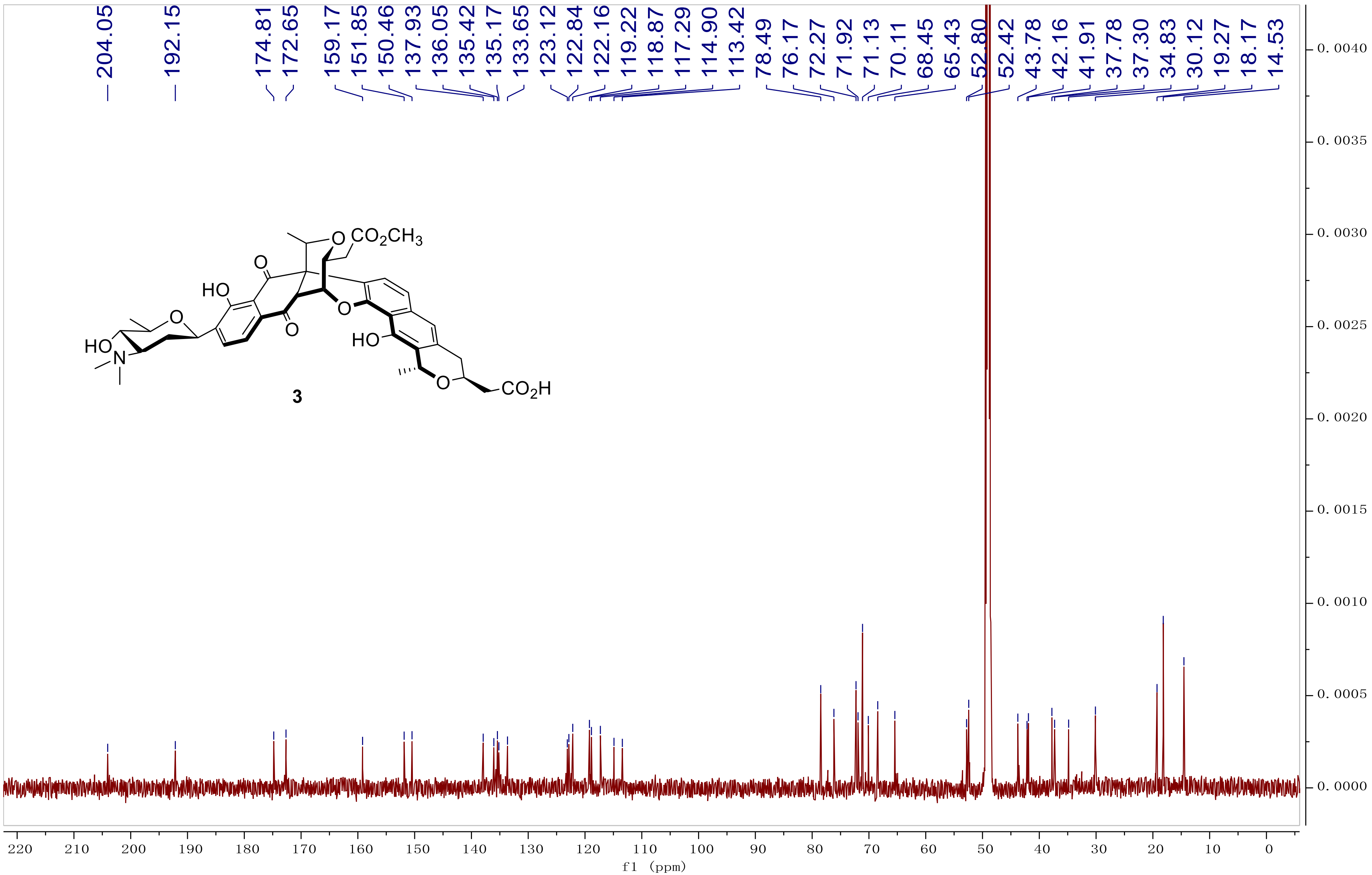

Supplementary Fig. 25. HSQC spectrum of chimedermycin C (**3**) in methanol-d_4

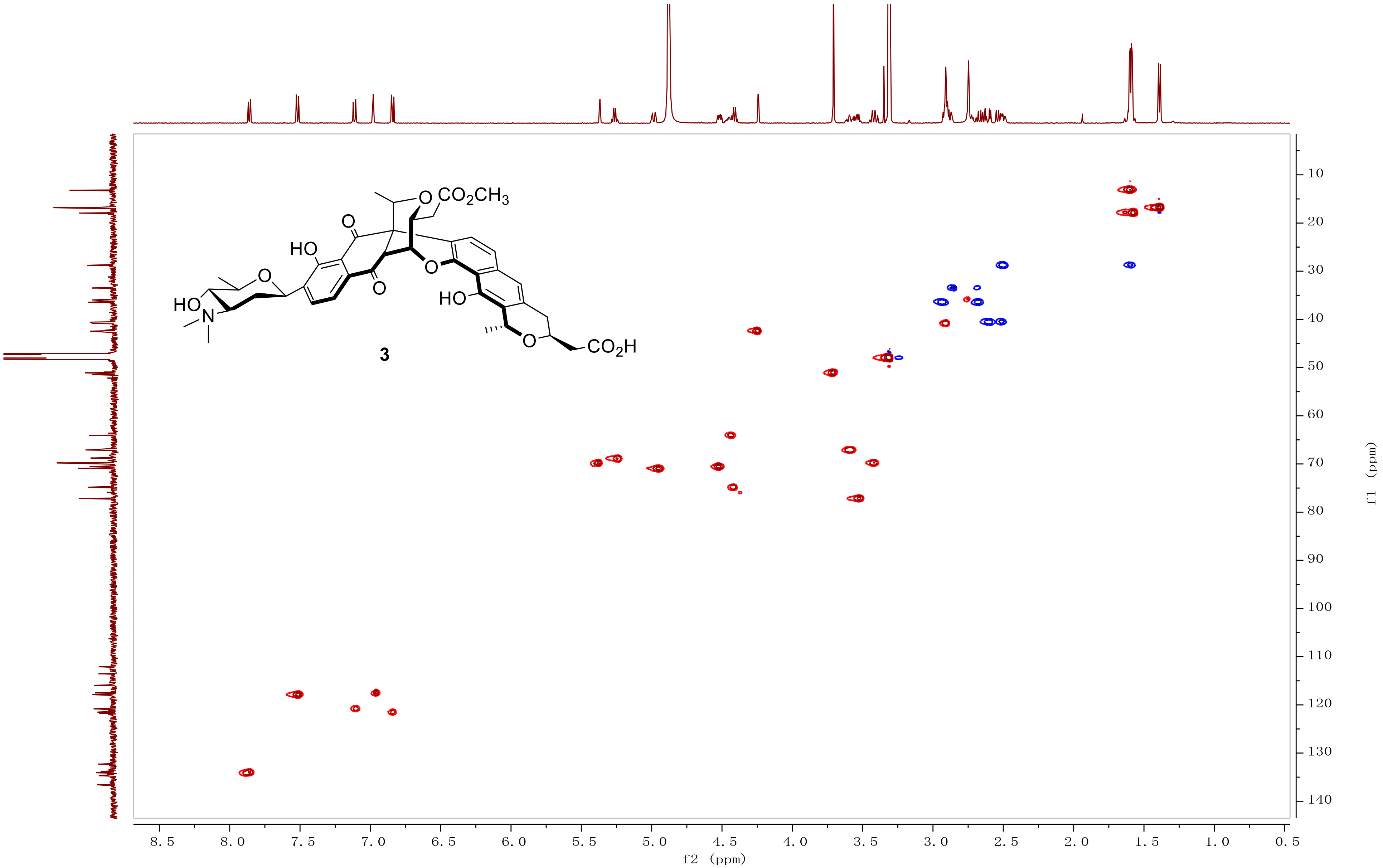

Supplementary Fig. 26. ^{1}H-^{1}H COSY spectrum of chimedermycin C (**3**) in methanol-d_4

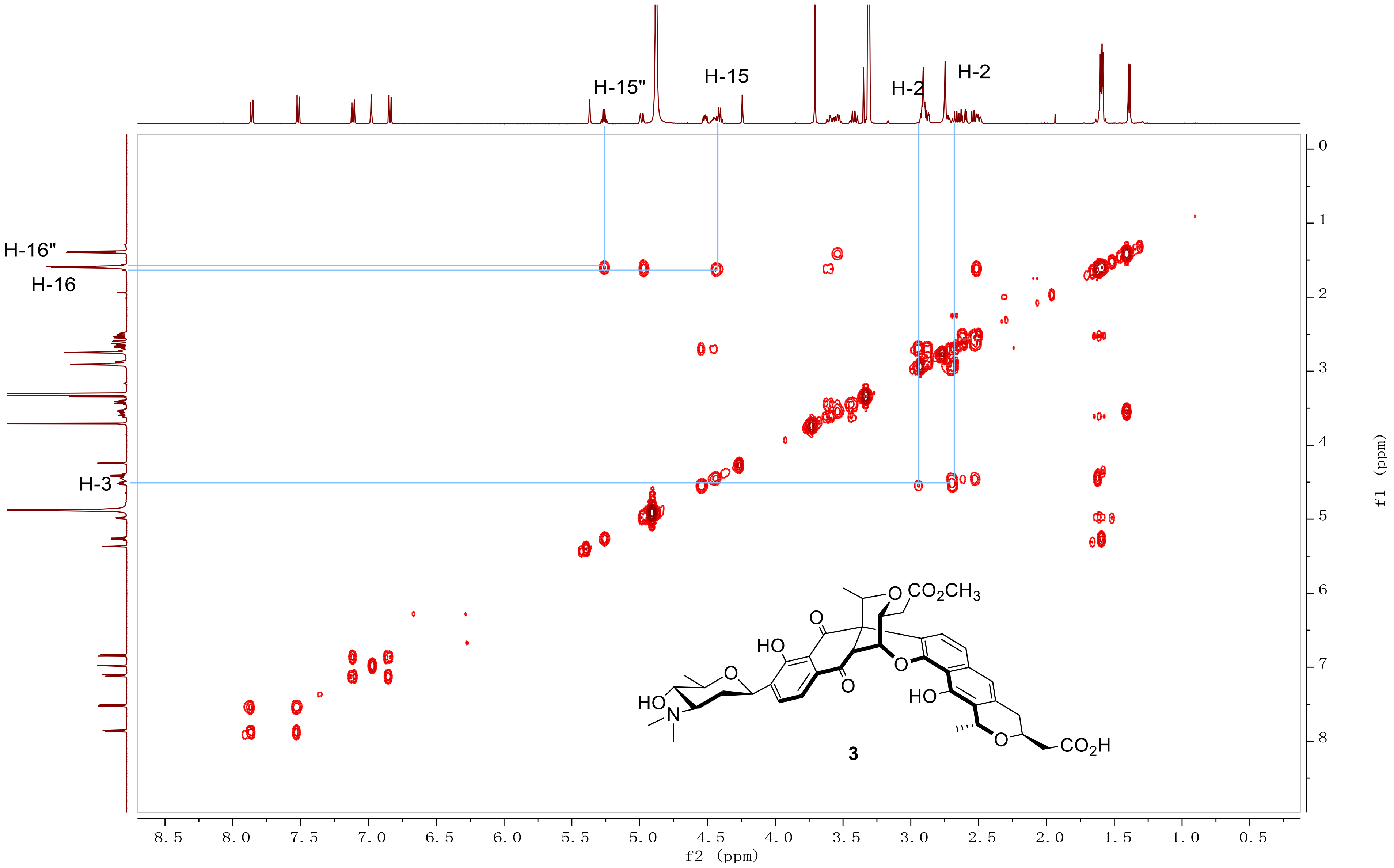

Supplementary Fig. 27. HMBC spectrum of chimedermycin C (**3**) in methanol-d_4

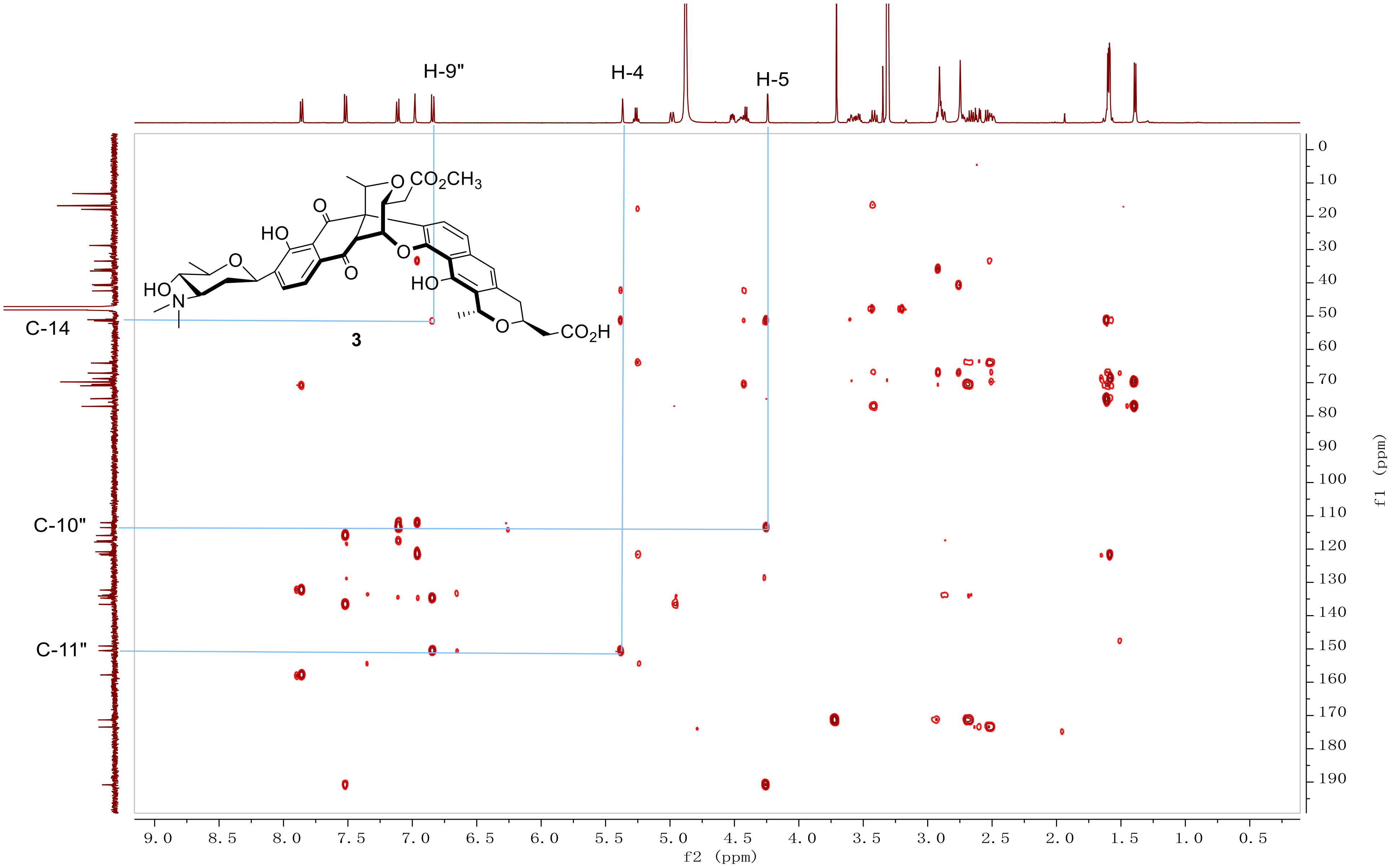

Supplementary Fig. 28. NOESY spectrum of chimedermycin C (**3**) in methanol-d_4

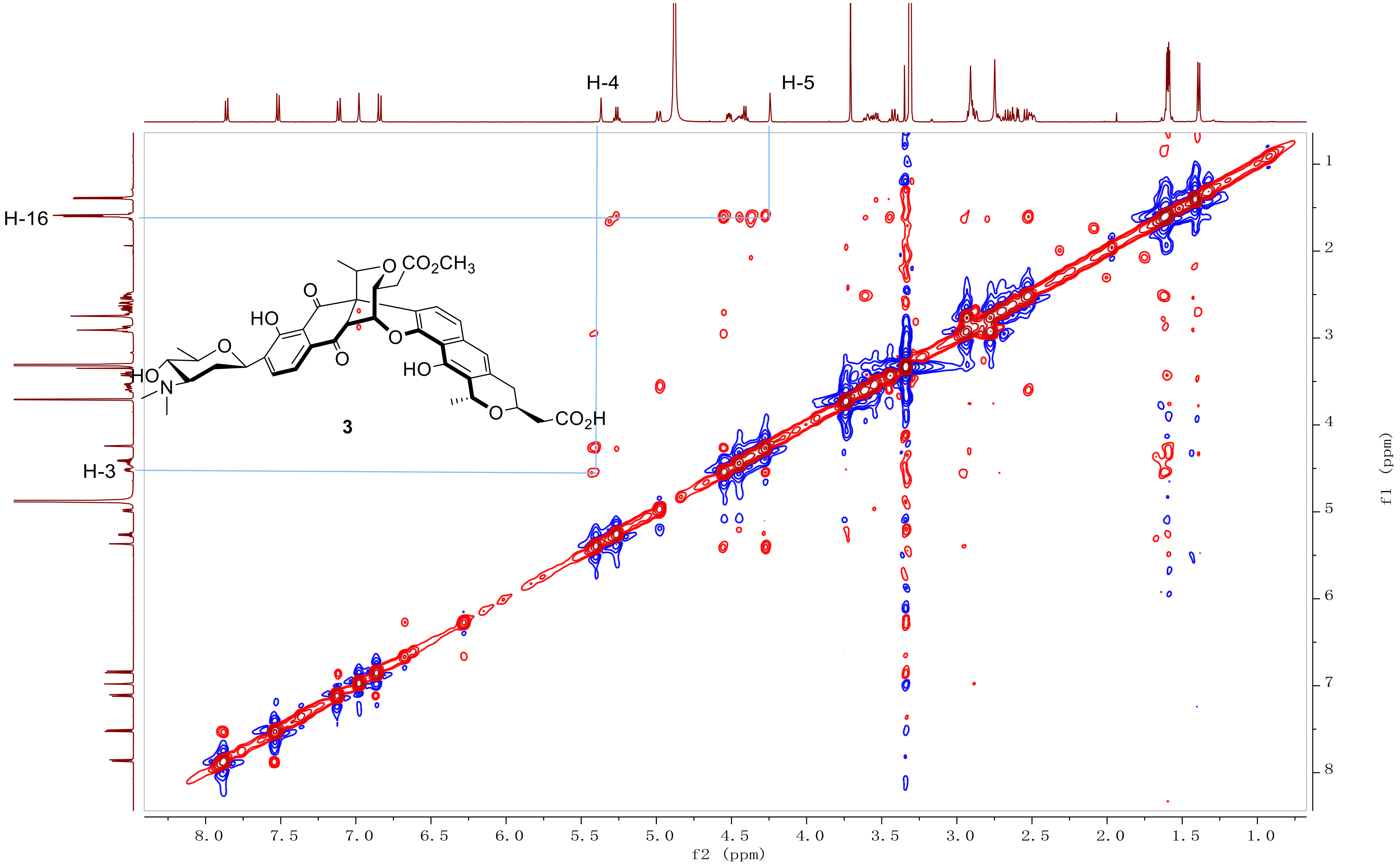

Supplementary Fig. 29. HRESIMS spectrum of chimedermycin D (**4**)

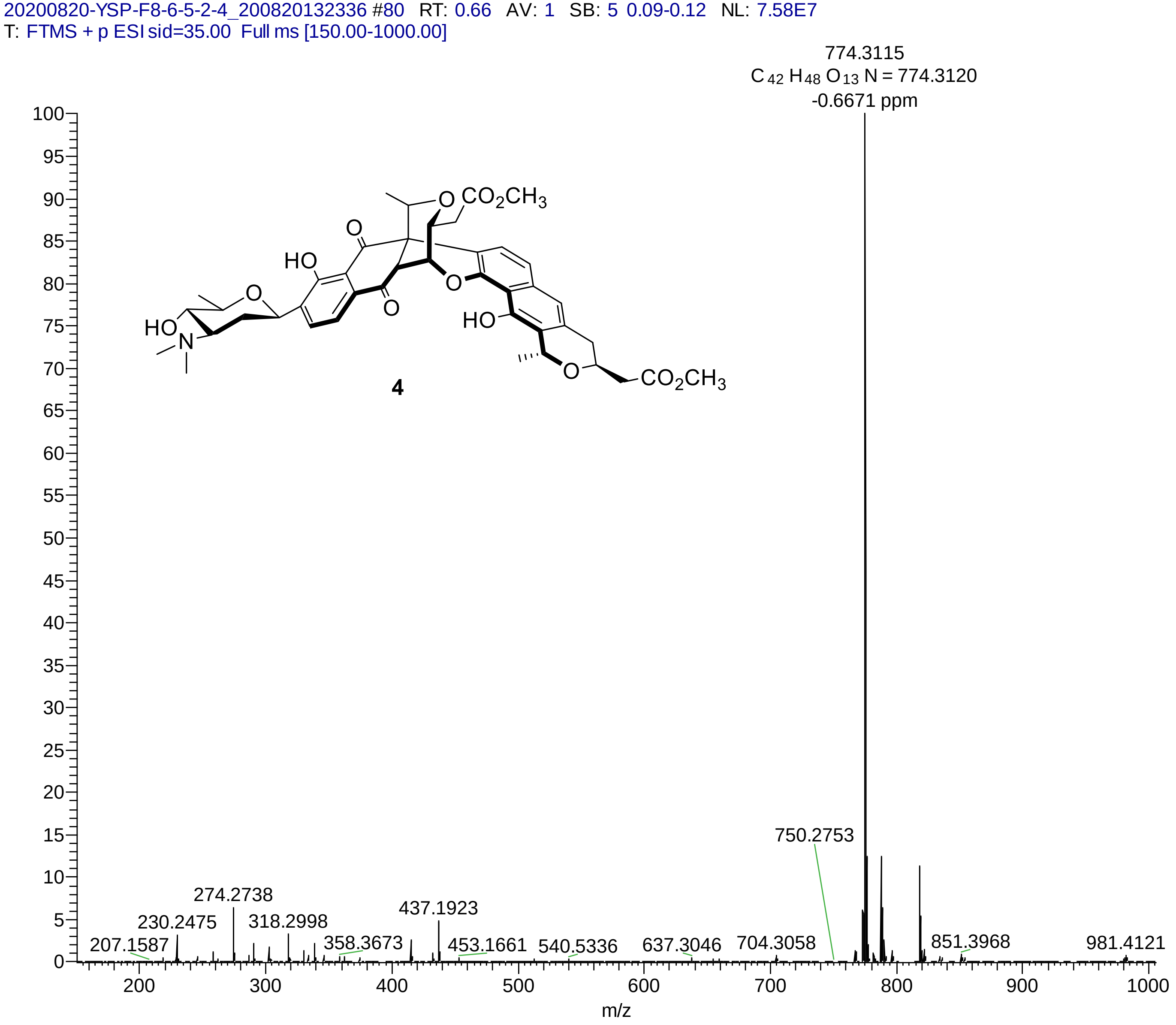

Supplementary Fig. 30. ^{1}H-NMR spectrum of chimedermycin D (**4**) in methanol-d_4

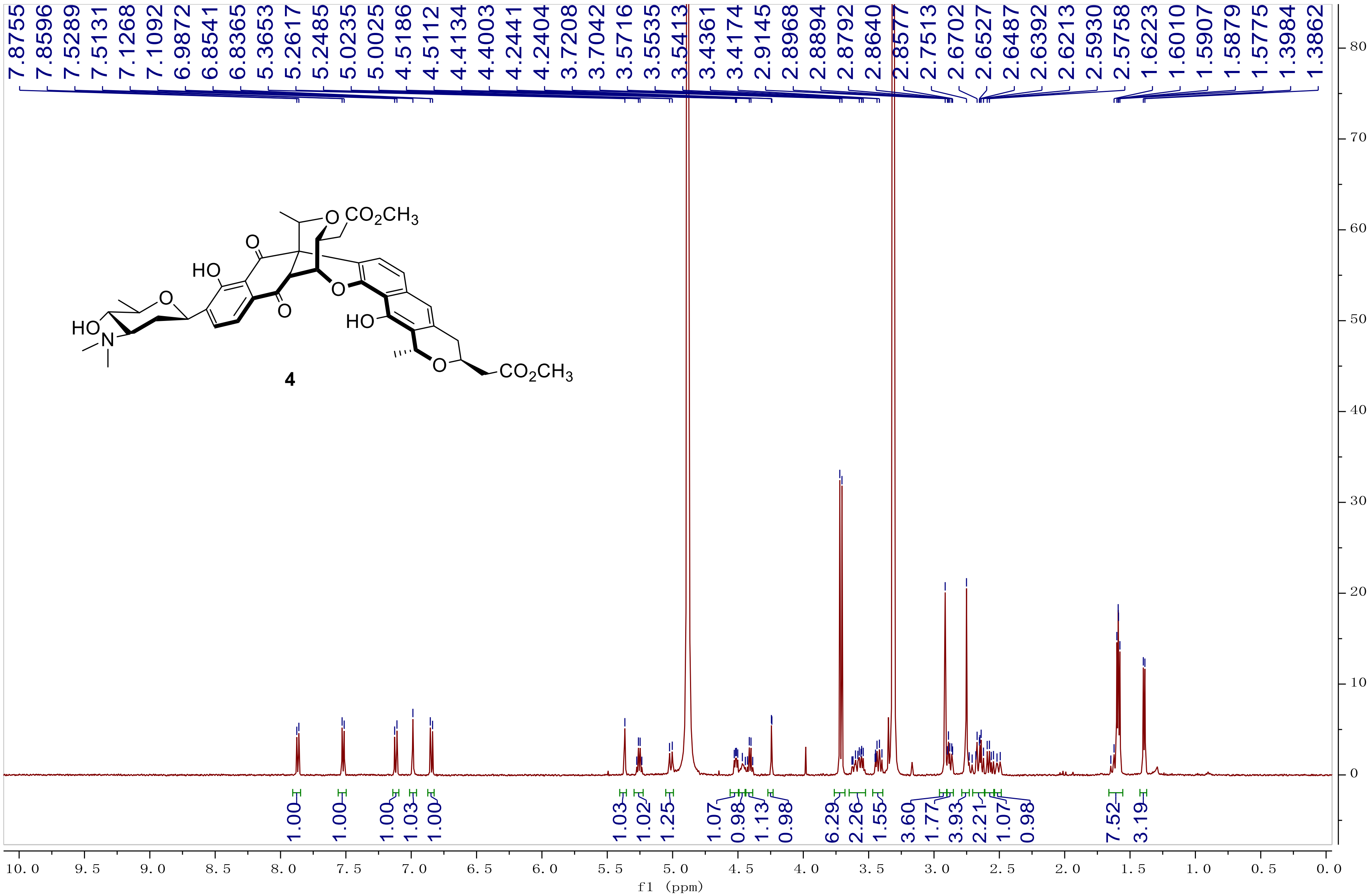

Supplementary Fig. 31. ^{13}C-NMR spectrum of chimedermycin D (**4**) in methanol-d_4

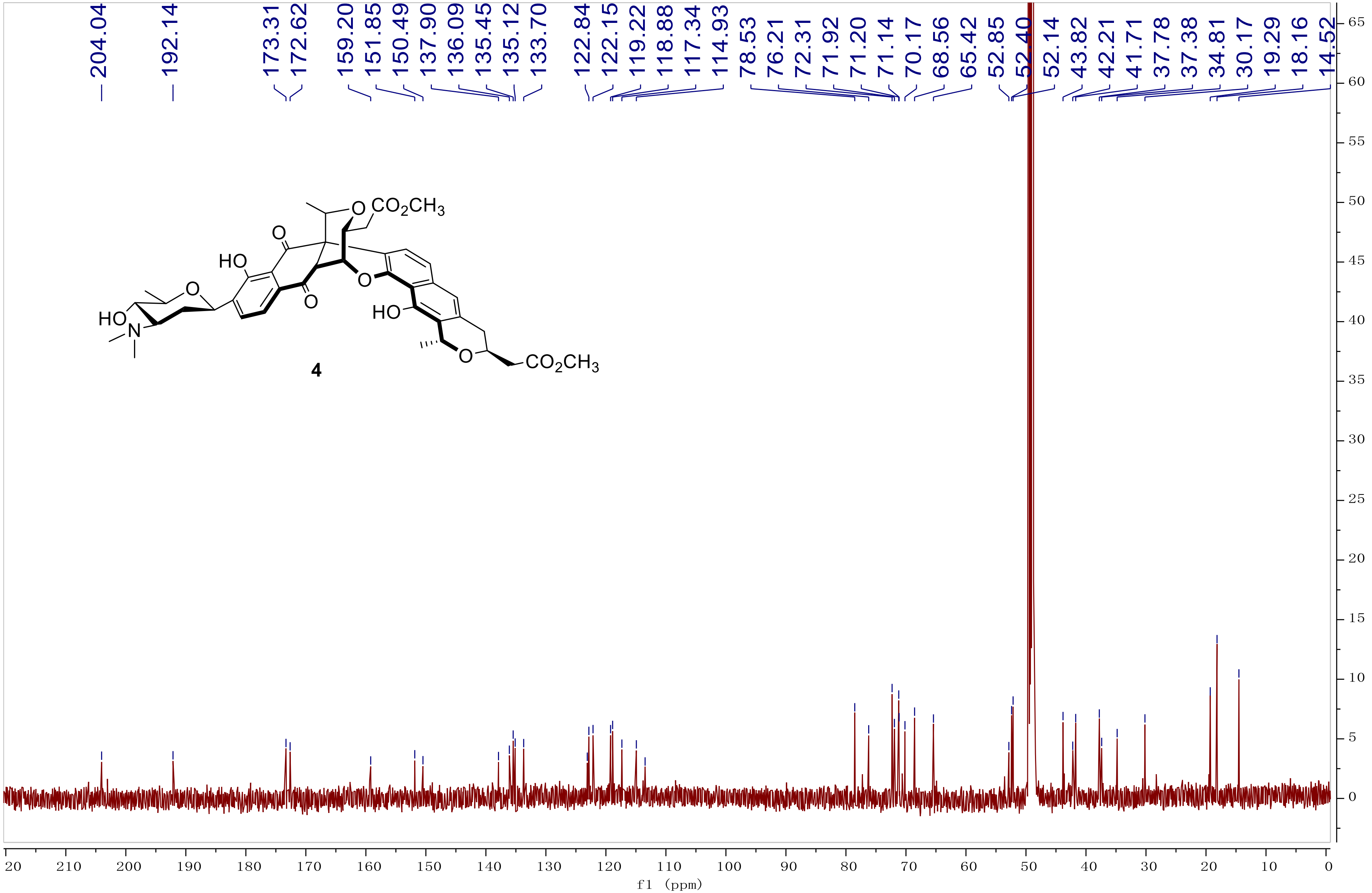

Supplementary Fig. 32. HSQC spectrum of chimedermycin D (**4**) in methanol-d_4

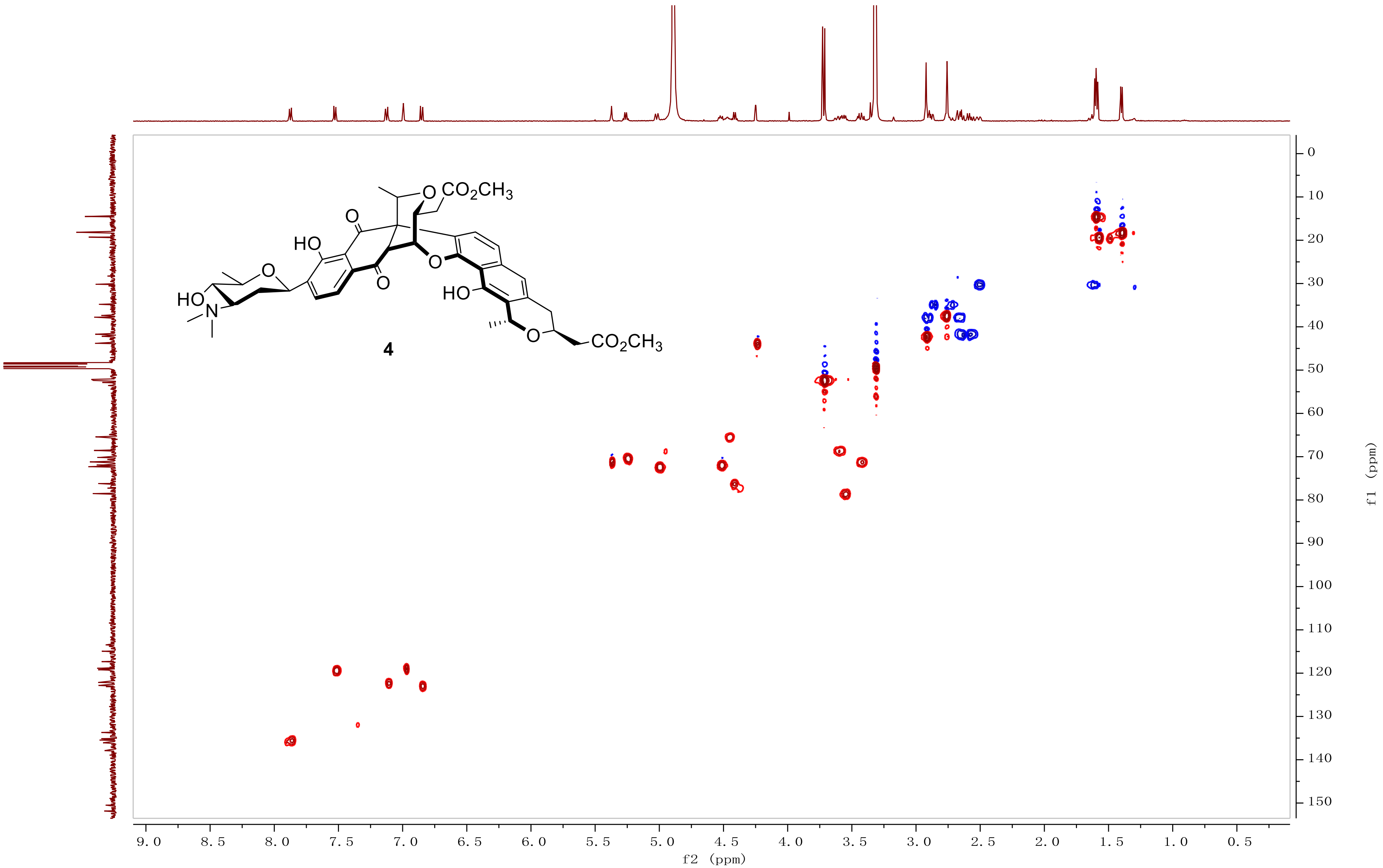

Supplementary Fig. 33. ^{1}H-^{1}H COSY spectrum of chimedermycin D (**4**) in methanol-*d*4

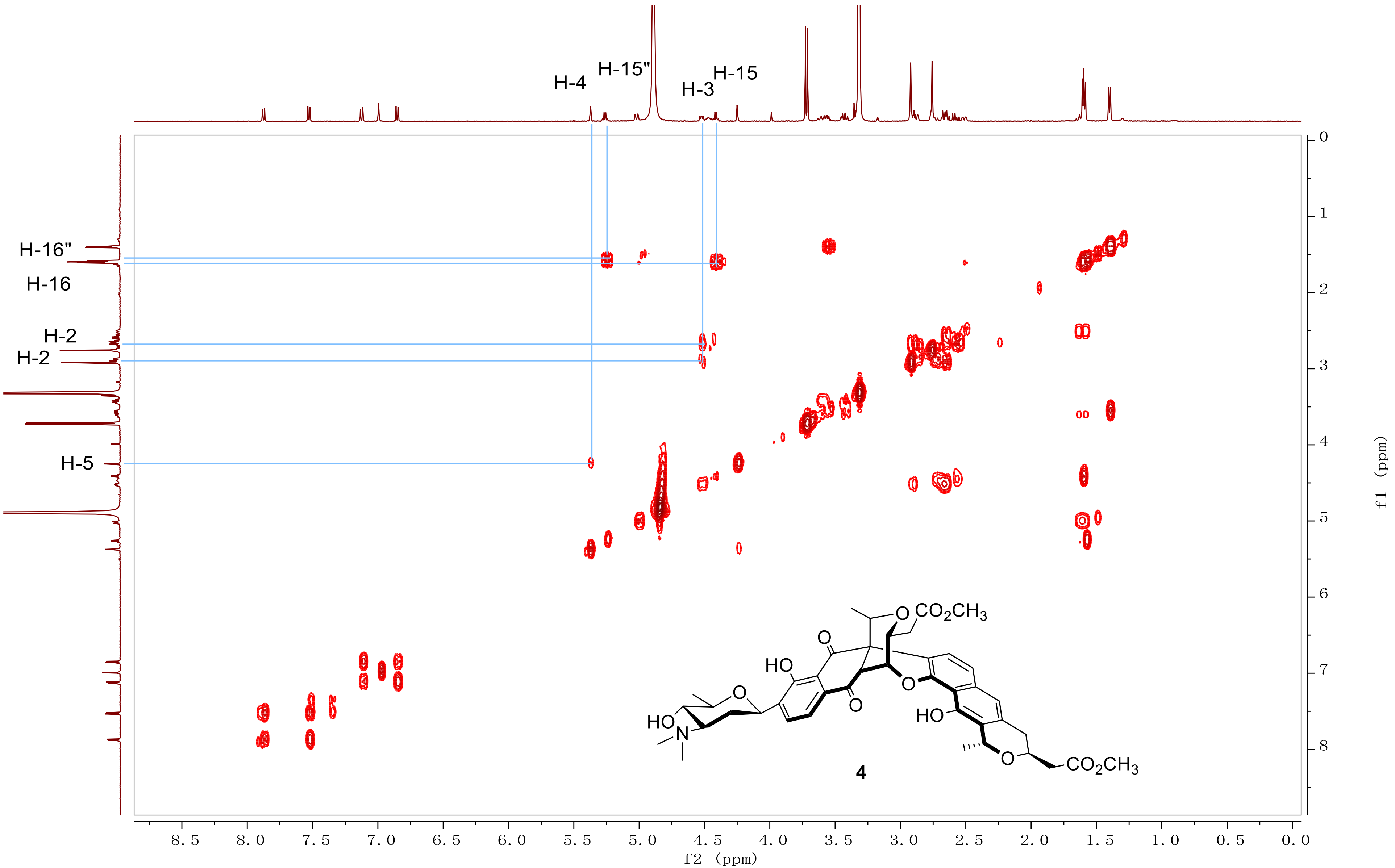

Supplementary Fig. 34. HMBC spectrum of chimedermycin D (**4**) in methanol-d_4

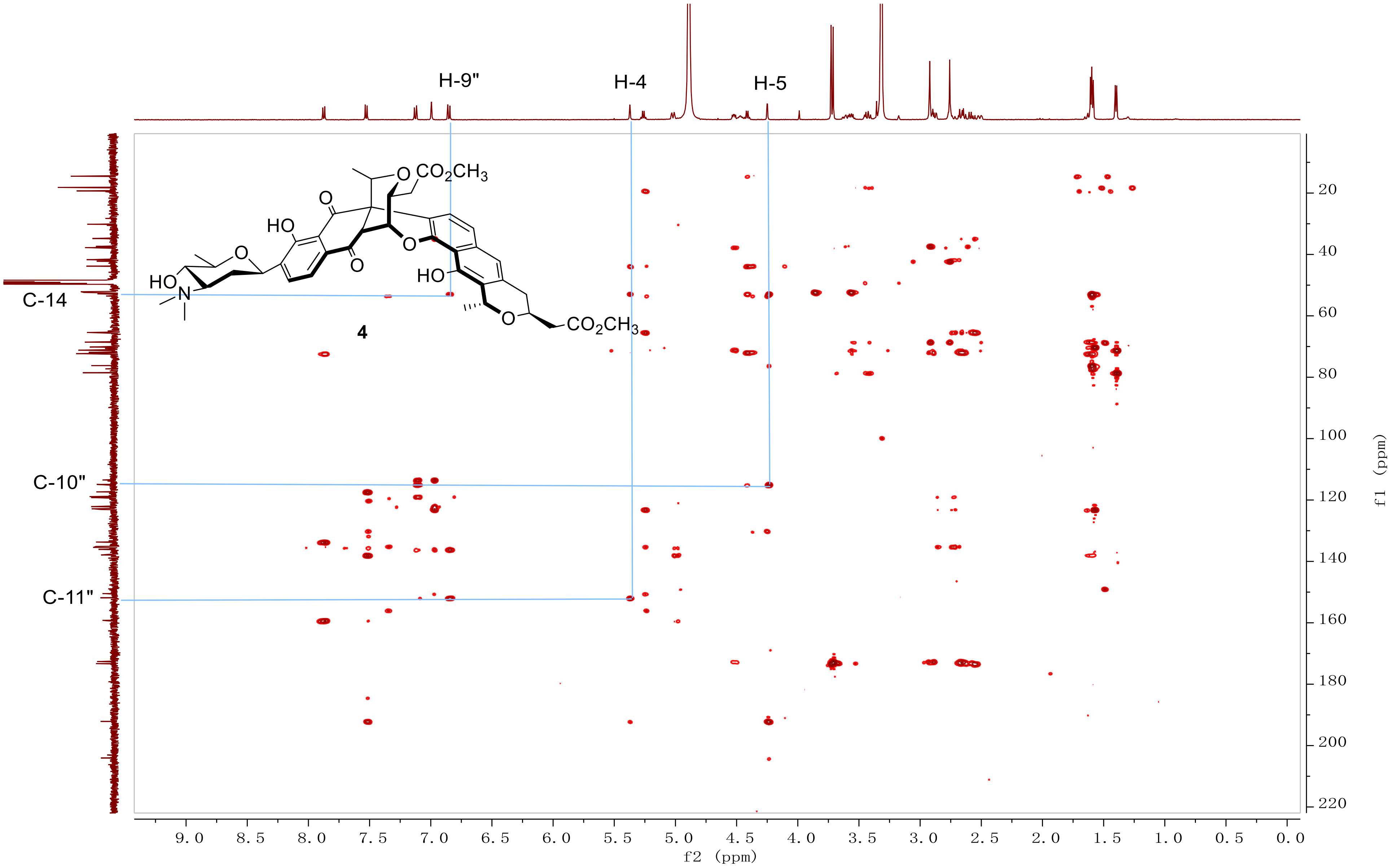

Supplementary Fig. 35. NOESY spectrum of chimedermycin D (**4**) in methanol-d_4

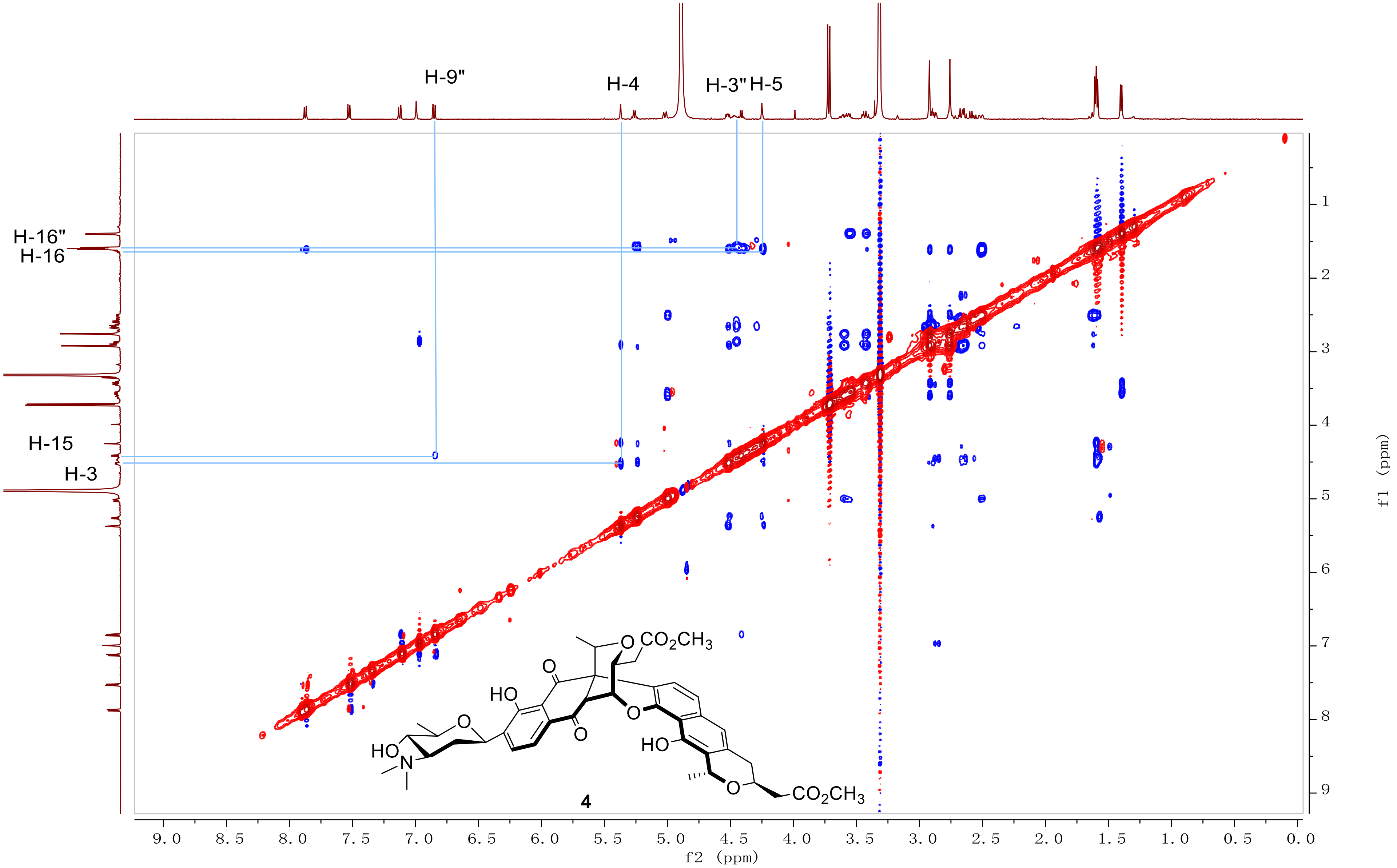

Supplementary Fig. 36. HRESIMS spectrum of chimedermycin E (**5**)

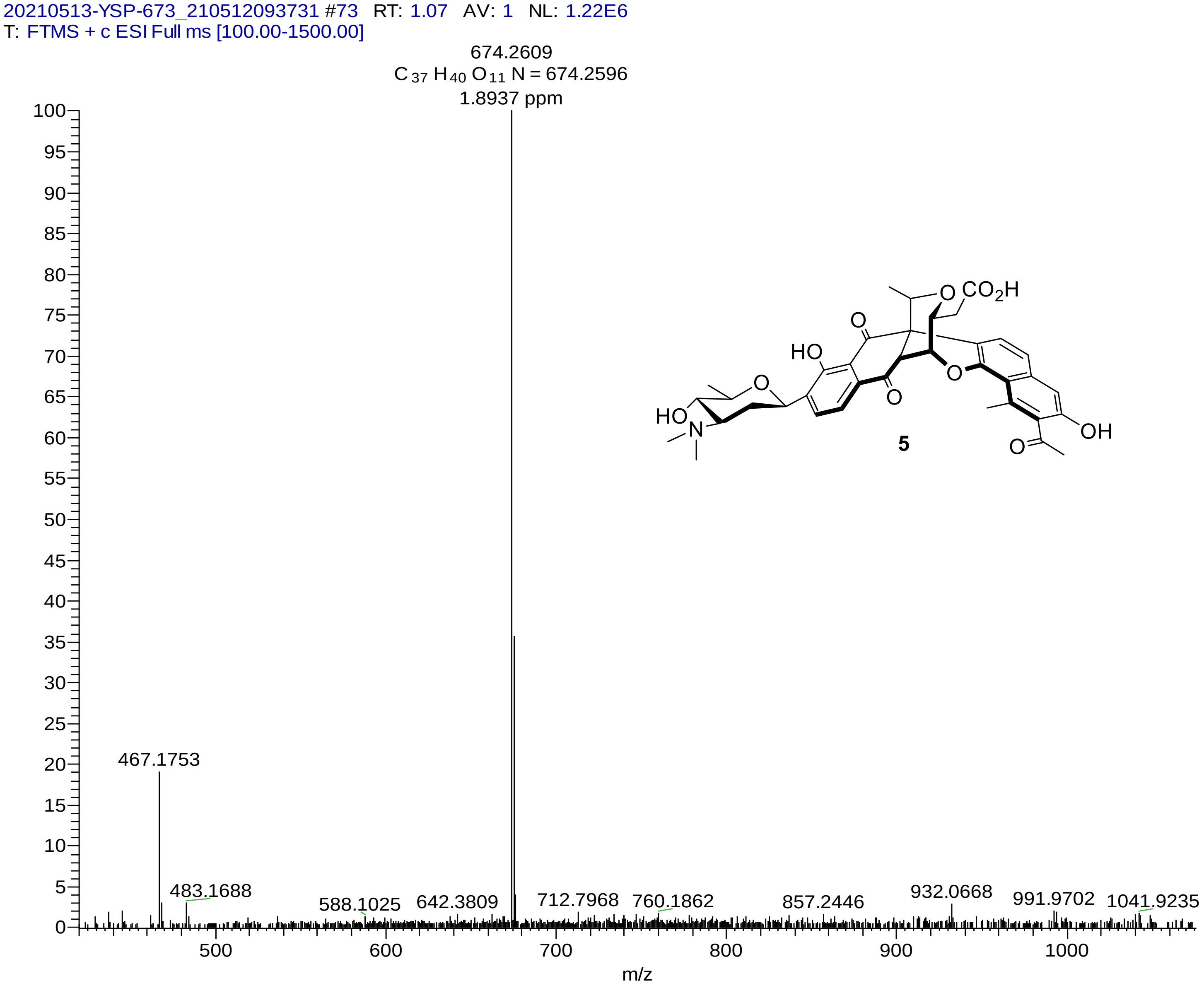

Supplementary Fig. 37. ^{1}H-NMR spectrum of chimedermycin E (**5**) in methanol-d_4

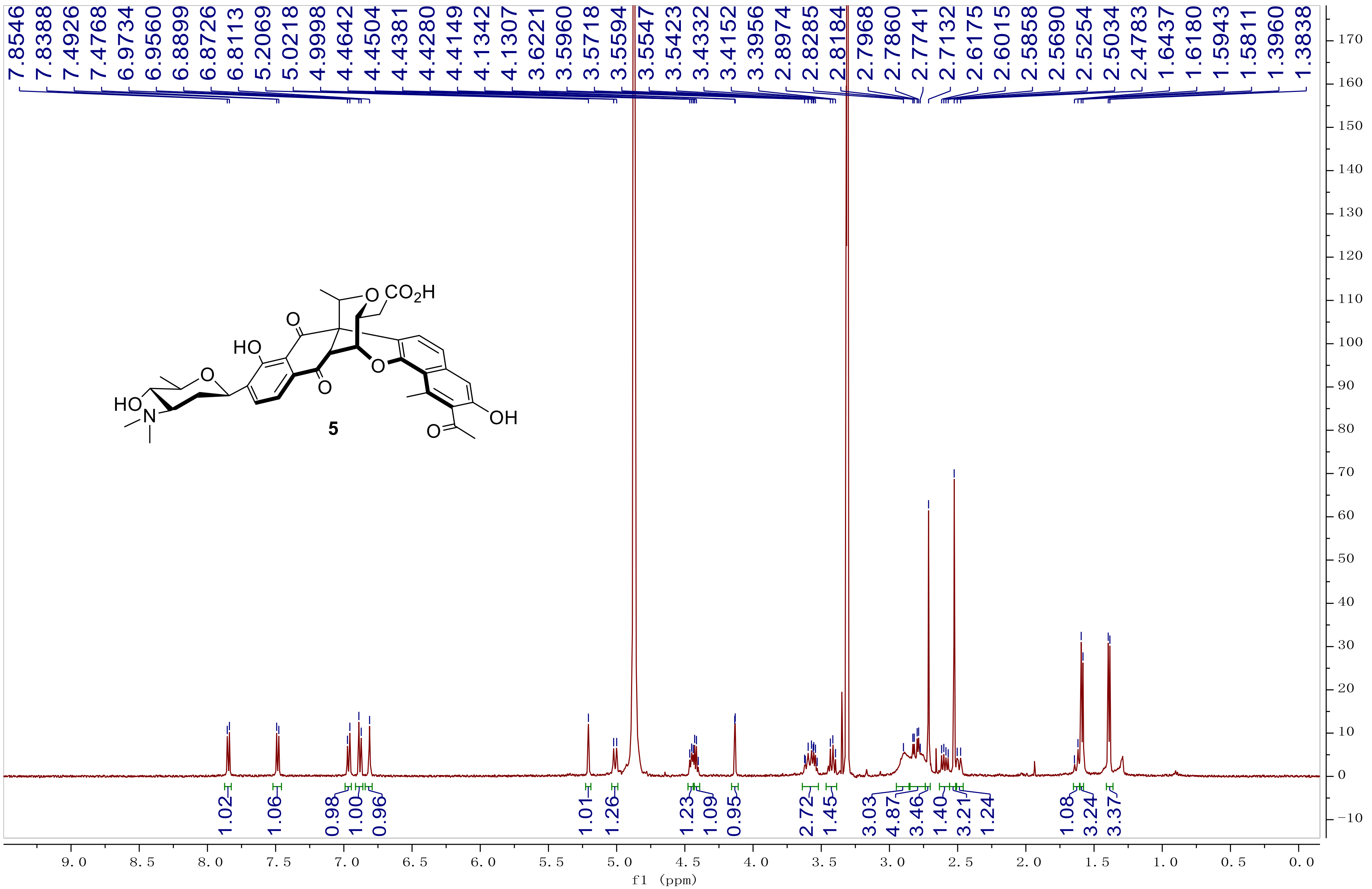

Supplementary Fig. 38. ^{13}C-NMR spectrum of chimedermycin E (**5**) in methanol-d_4

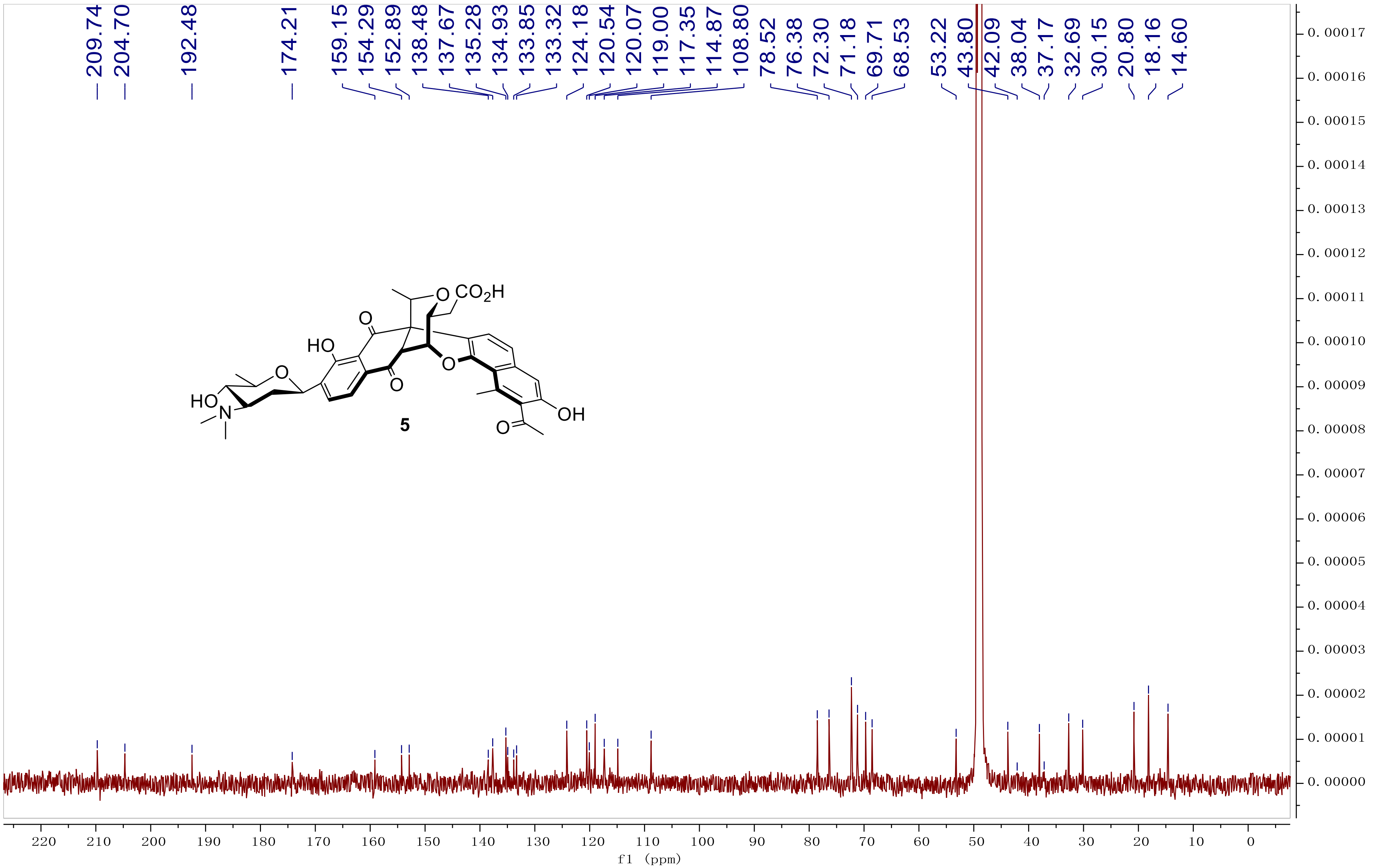

Supplementary Fig. 39. HSQC spectrum of chimedermycin E (**5**) in methanol-d_4

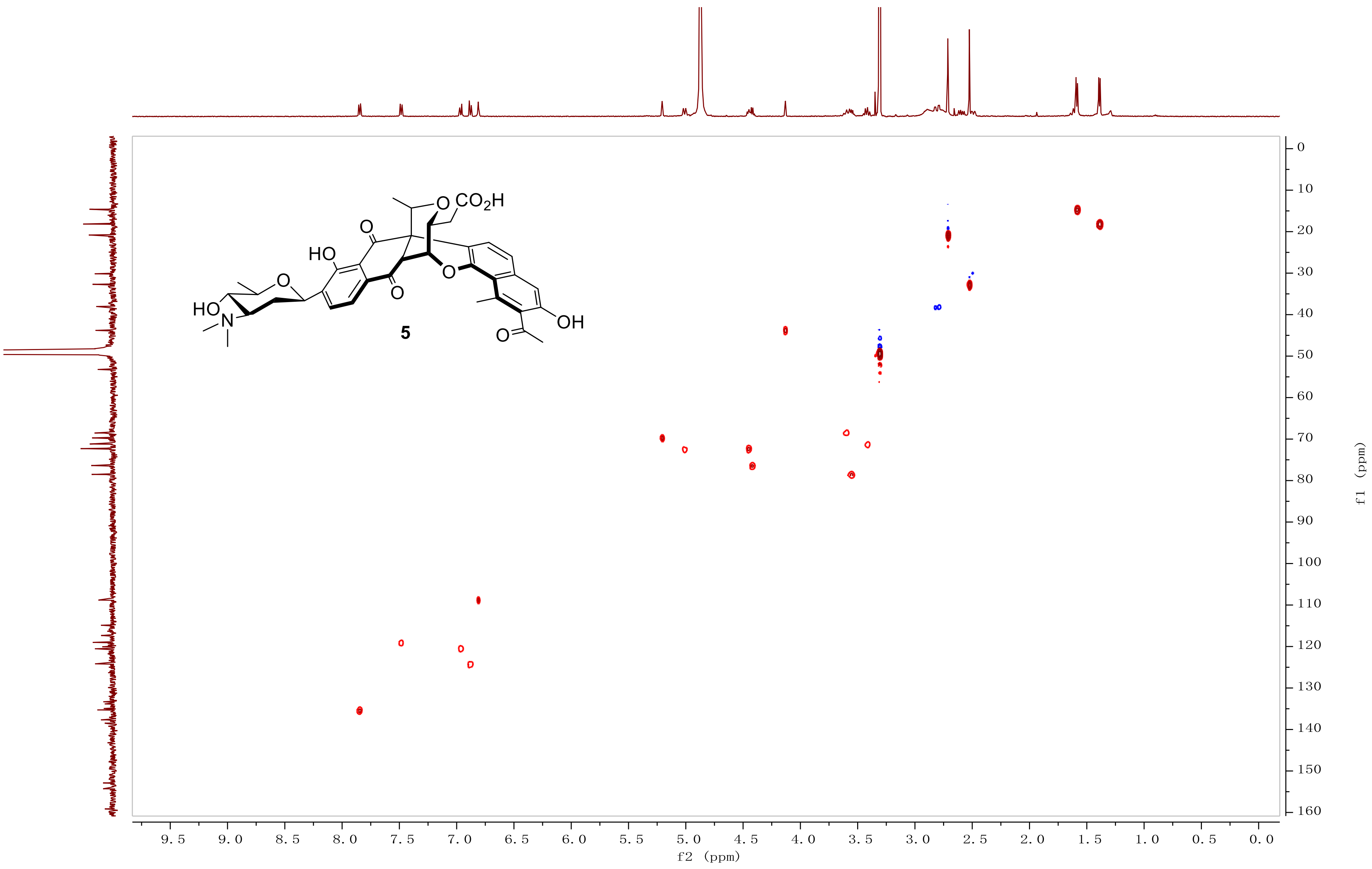

Supplementary Fig. 40. ^{1}H-^{1}H COSY spectrum of chimedermycin E (**5**) in methanol-d_4

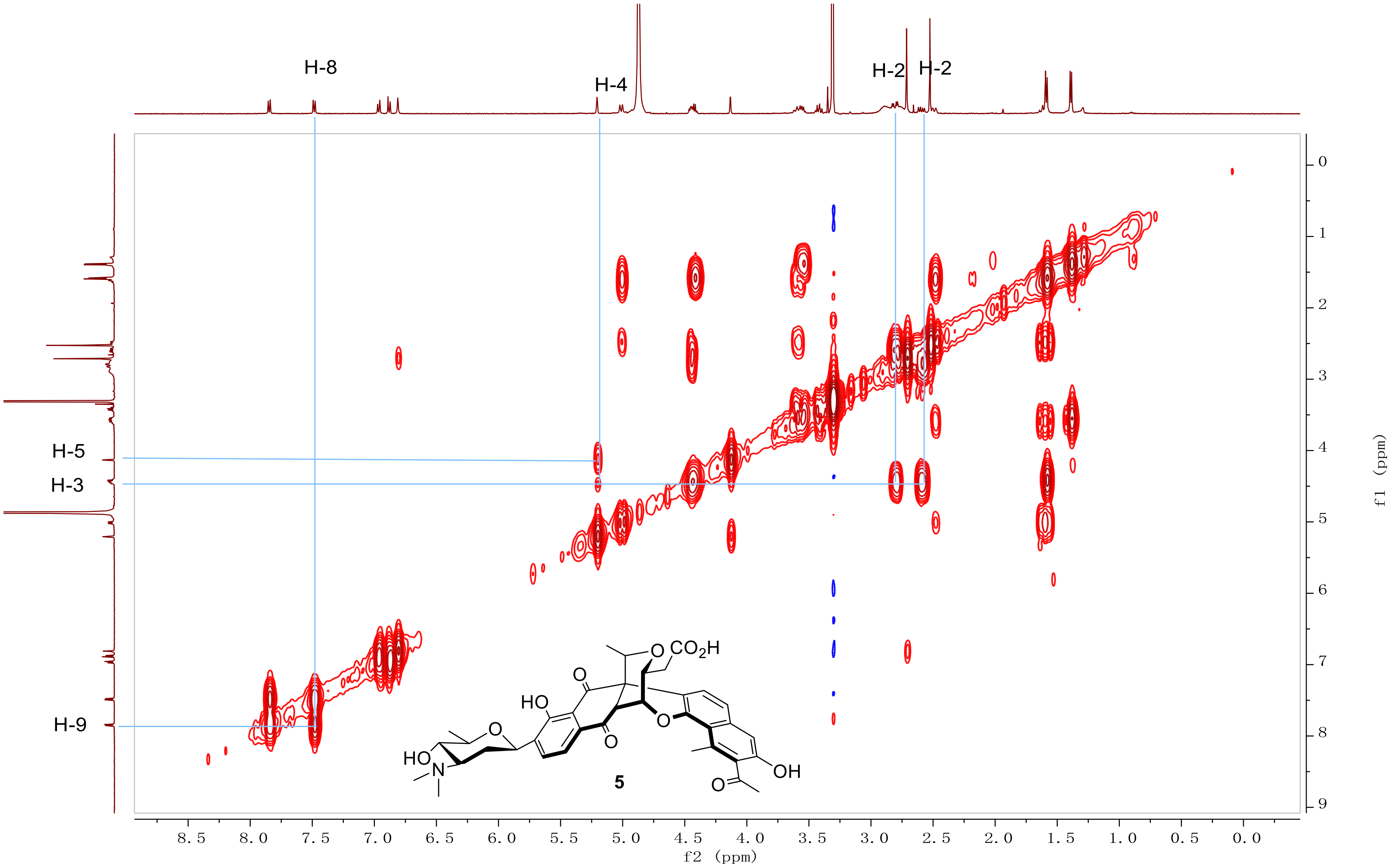

Supplementary Fig. 41. HMBC spectrum of chimedermycin E (**5**) in methanol-d_4

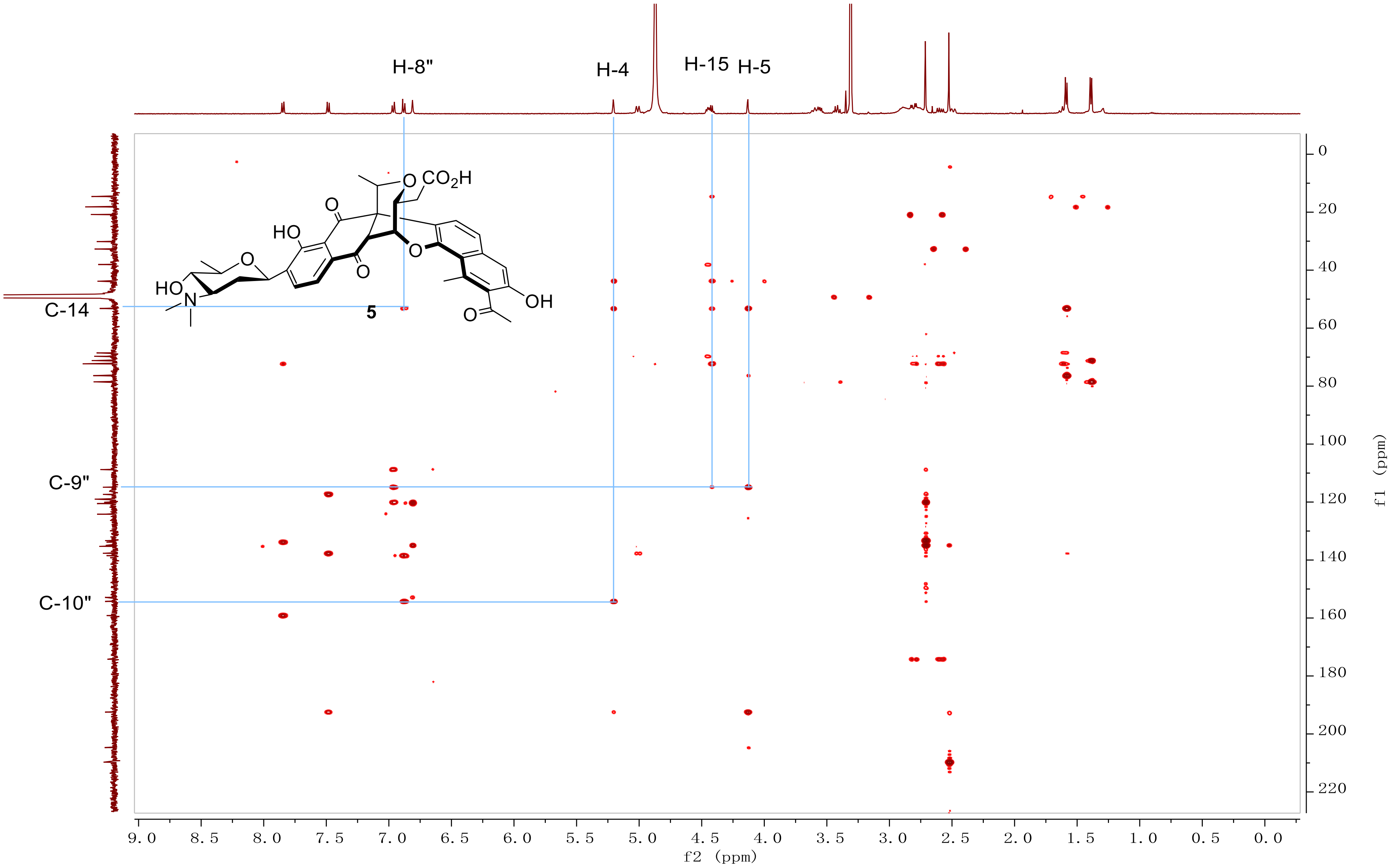

Supplementary Fig. 42. NOESY spectrum of chimedermycin E (**5**) in methanol-d_4

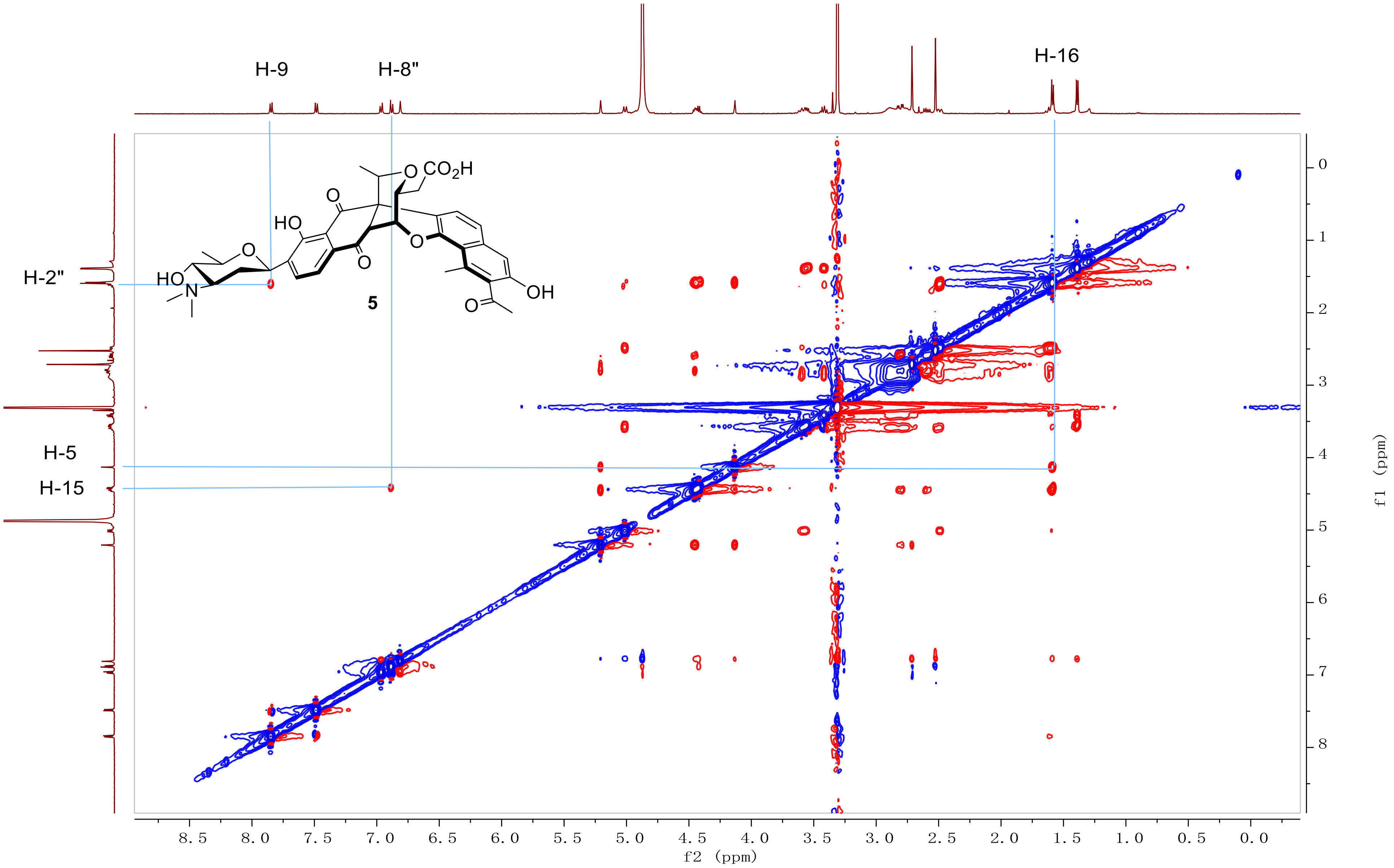

Supplementary Fig. 43. HRESIMS spectrum of chimedermycin F (**6**)

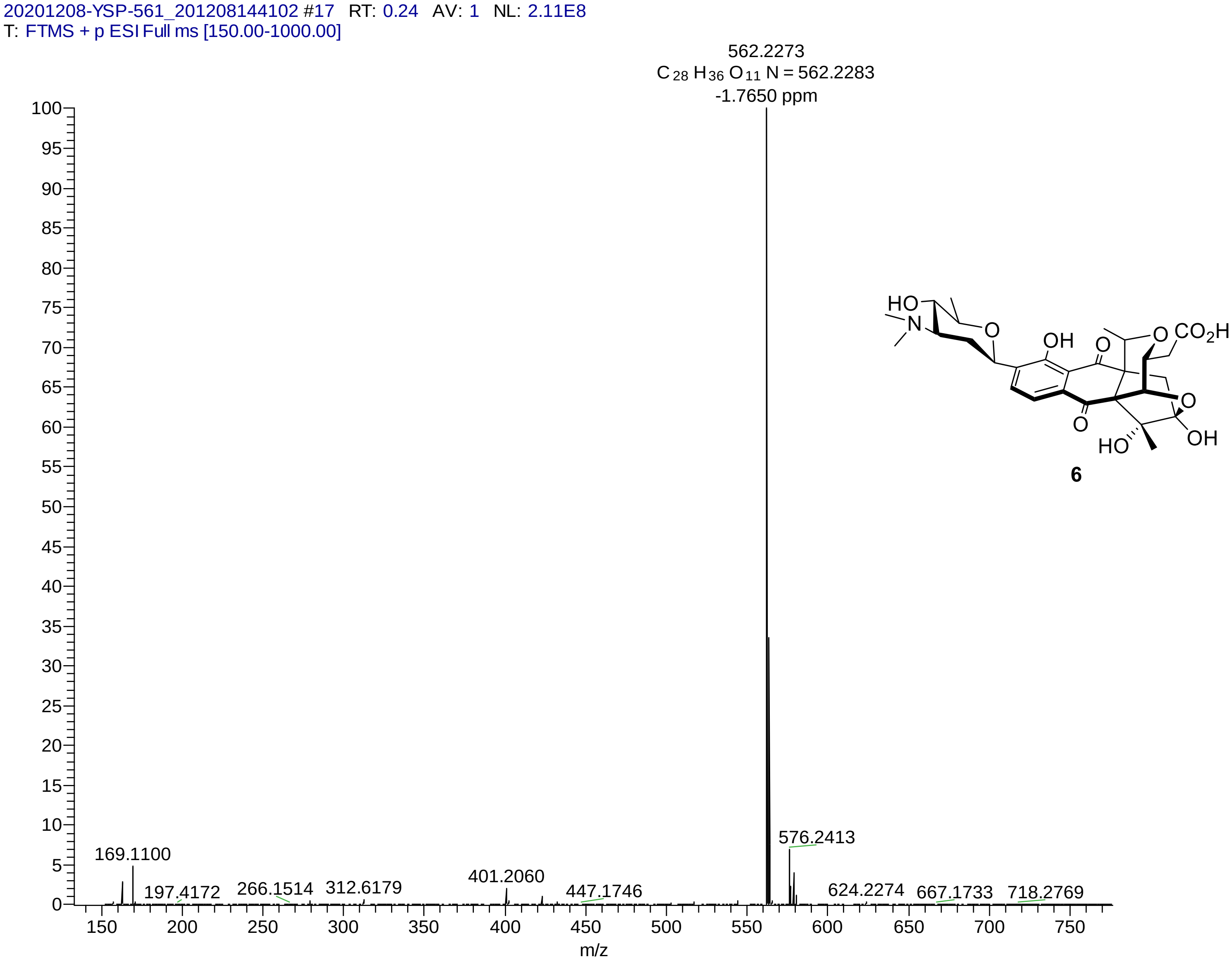

6

Supplementary Fig. 44. ^{1}H-NMR spectrum of chimedermycin F (**6**) in methanol-d_4

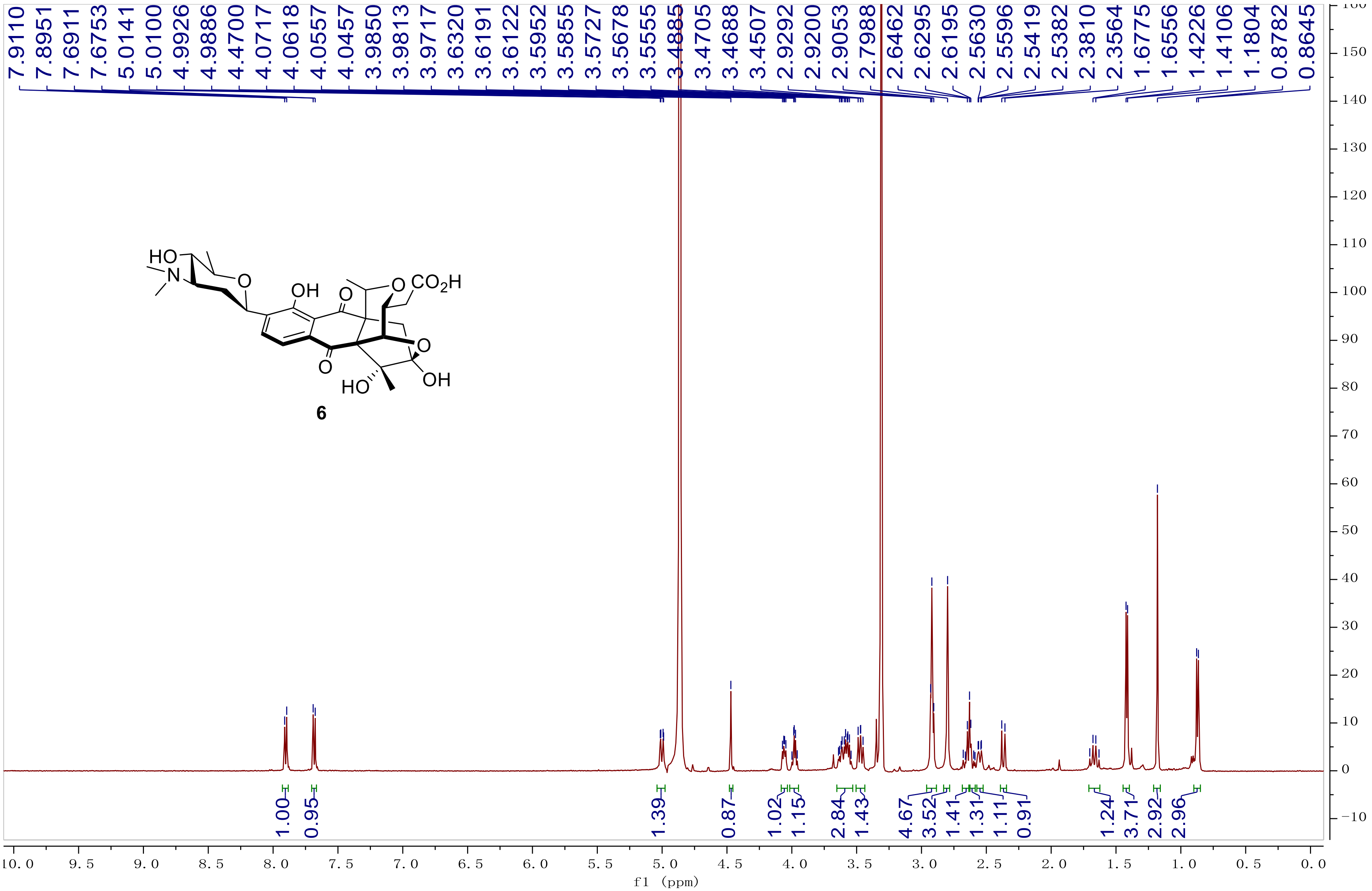

Supplementary Fig. 45. ^{13}C-NMR spectrum of chimedermycin F (**6**) in methanol-d_4

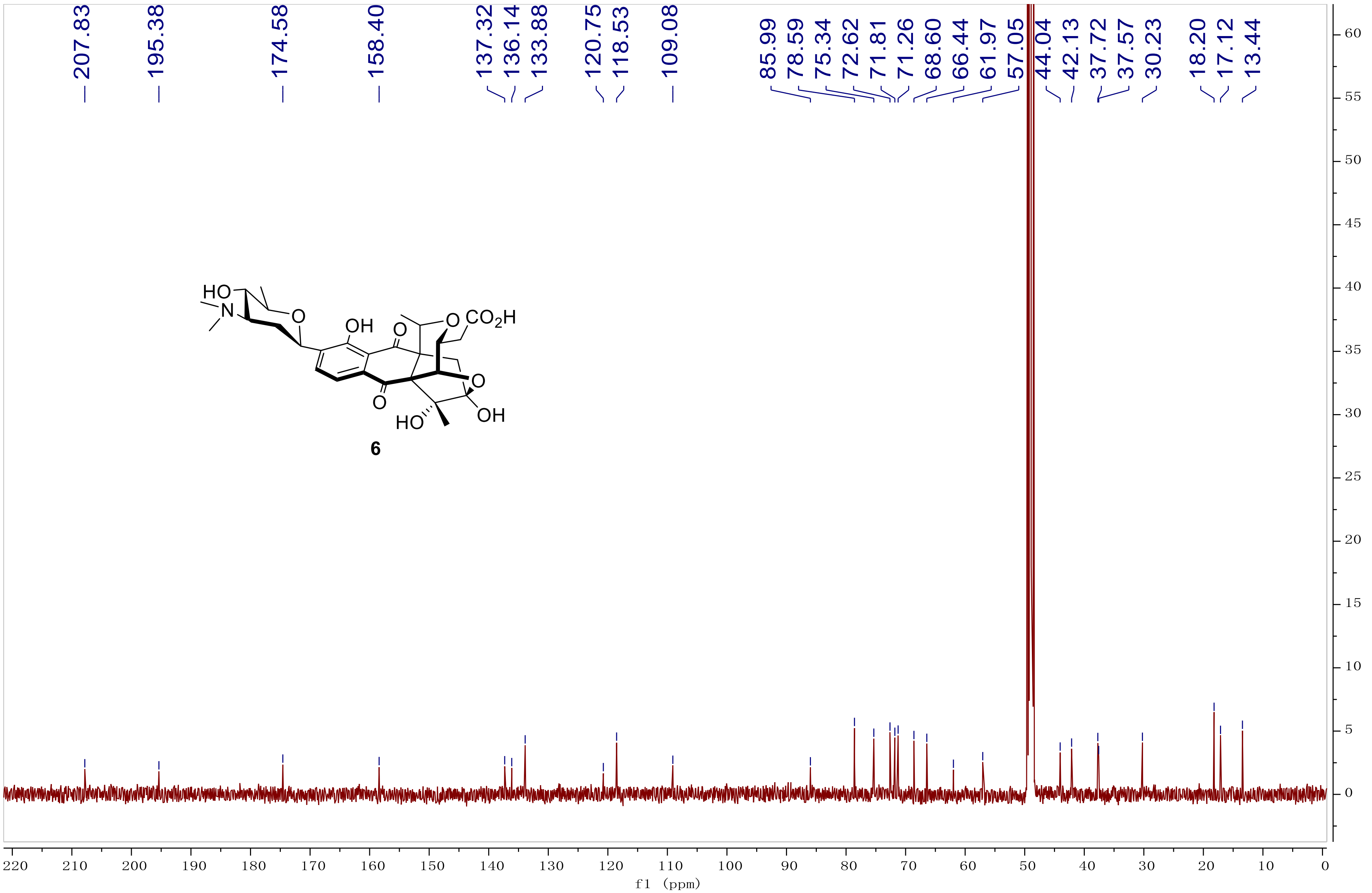

Supplementary Fig. 46. HSQC spectrum of chimedermycin F (**6**) in methanol-d_4

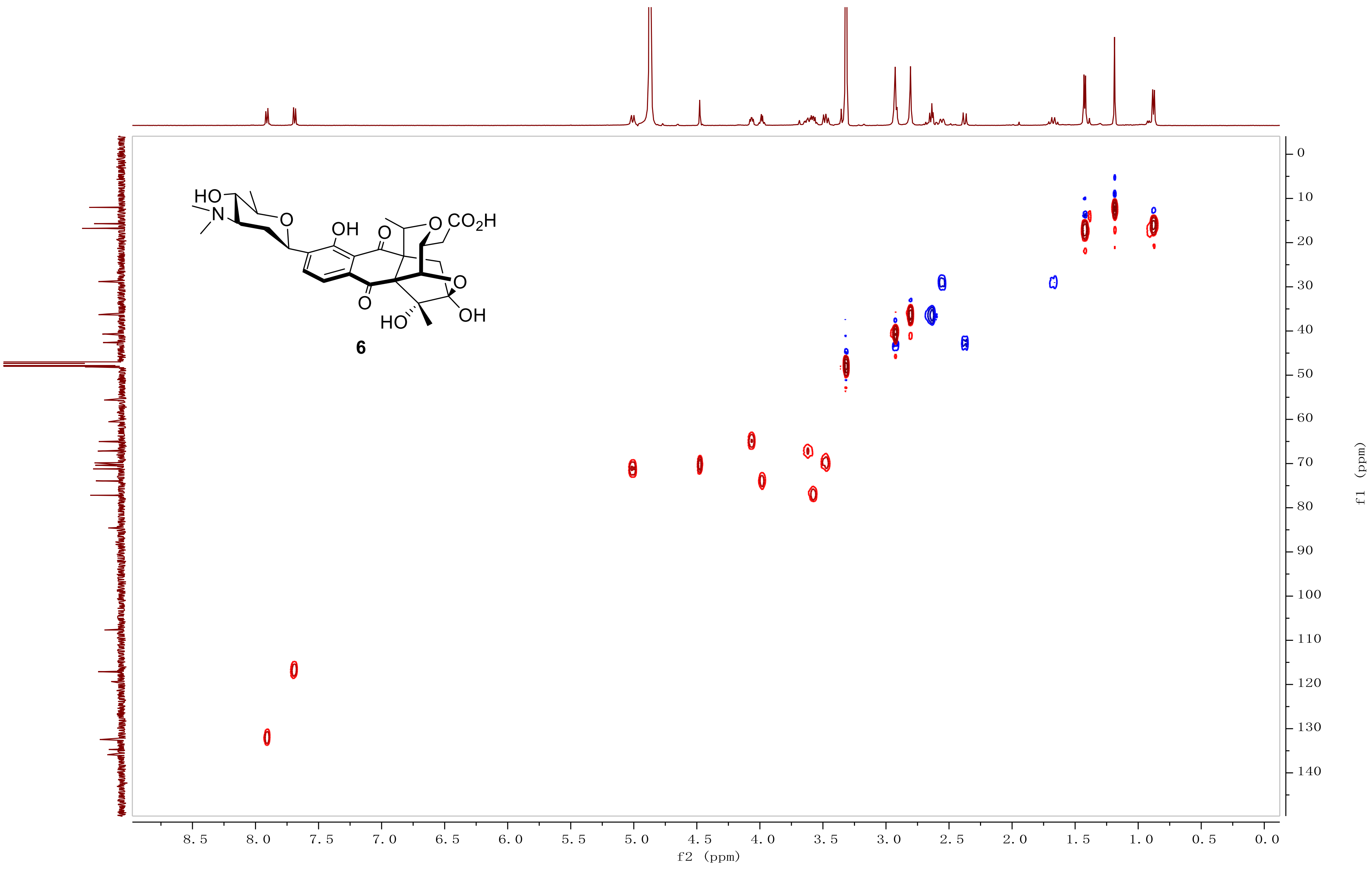

Supplementary Fig. 47. ¹H-¹H COSY spectrum of chimedermycin F (**6**) in methanol-d_4

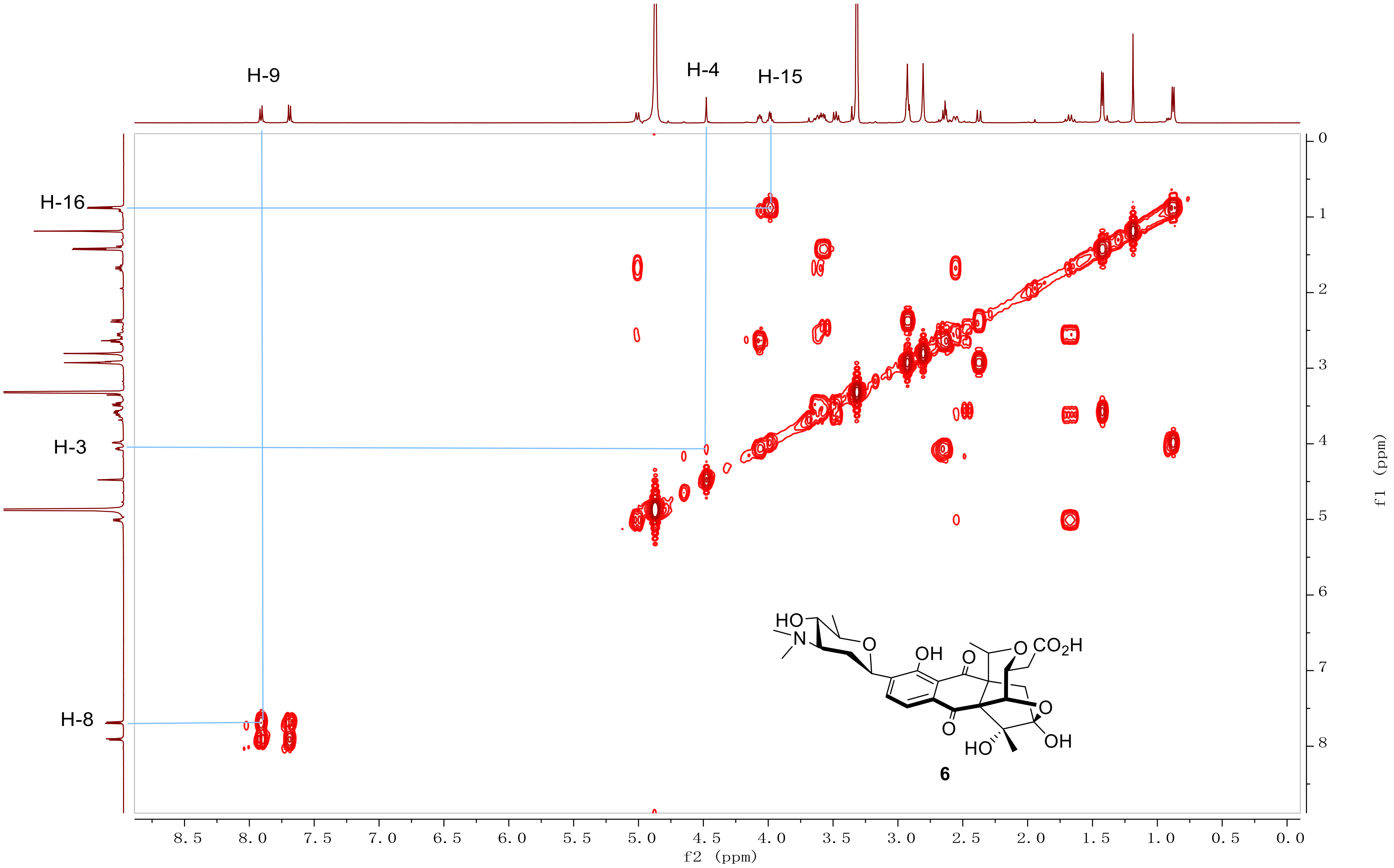

Supplementary Fig. 48. HMBC spectrum of chimedermycin F (**6**) in methanol-d_4

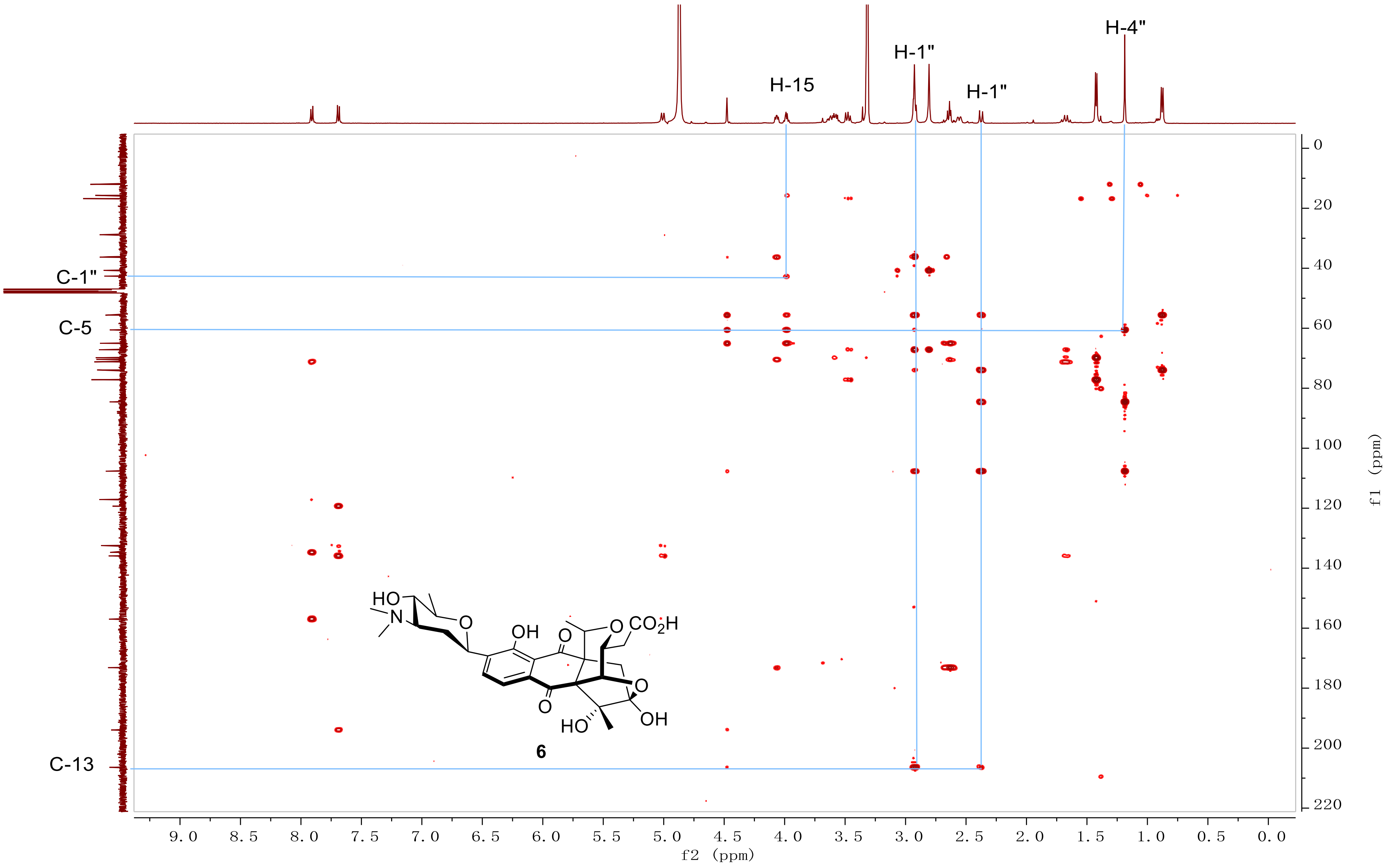

Supplementary Fig. 49. NOESY spectrum of chimedermycin F (**6**) in methanol-d_4

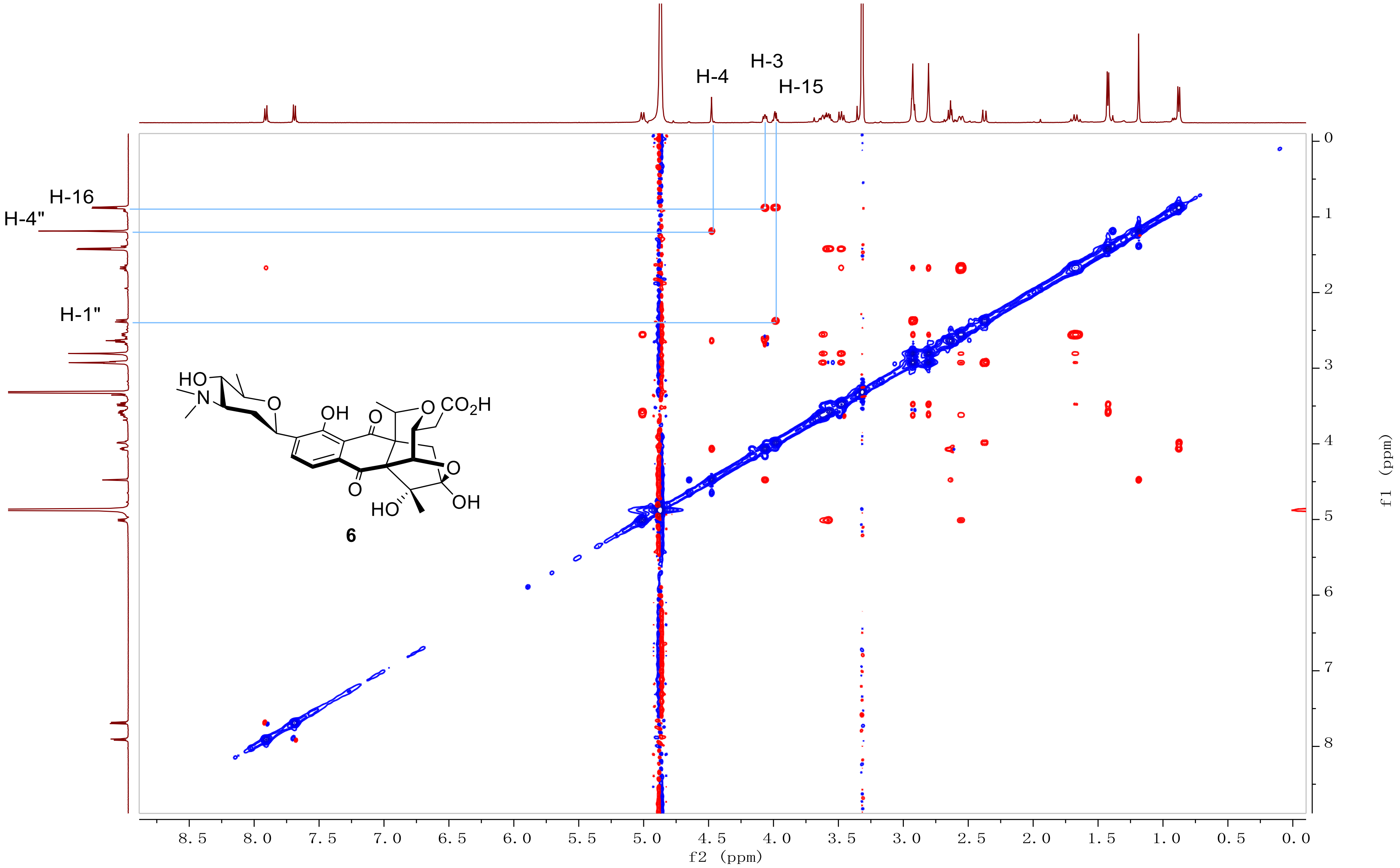

Supplementary Fig. 50. HRESIMS spectrum of chimedermycin G (**7**)

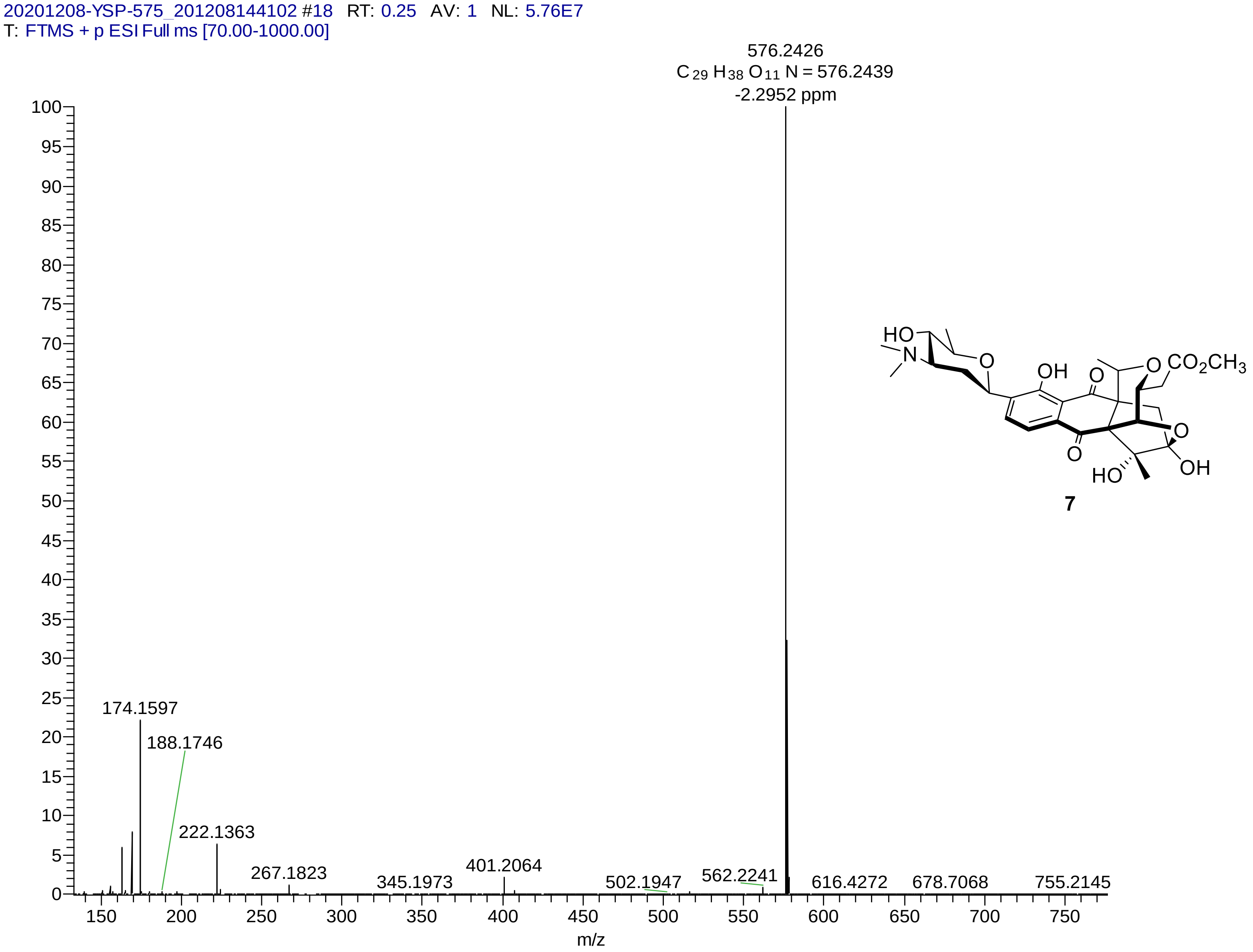

7

Supplementary Fig. 51. ^{1}H-NMR spectrum of chimedermycin G (**7**) in methanol-d_4

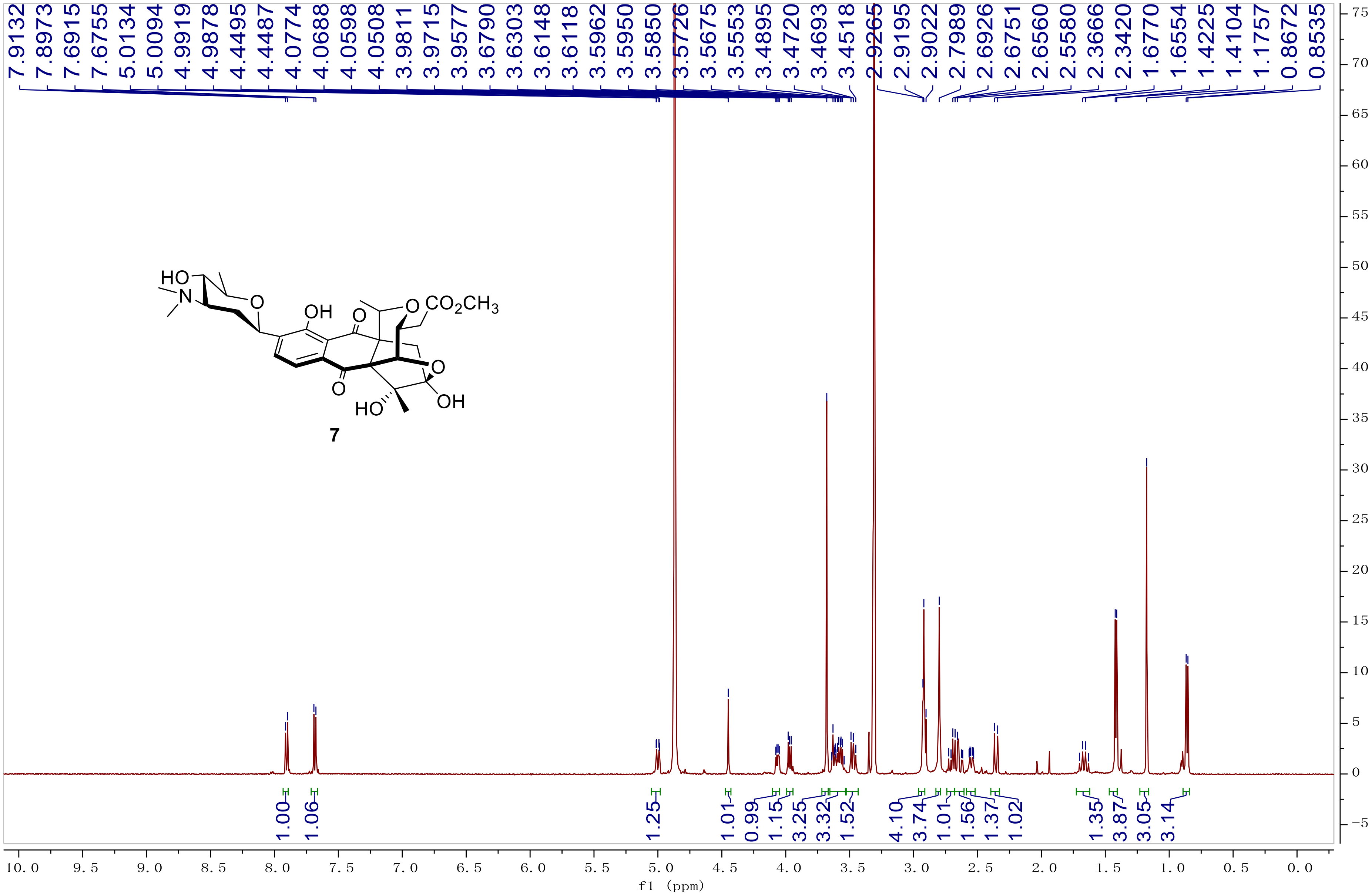

Supplementary Fig. 52. ^{13}C-NMR spectrum of chimedermycin G (**7**) in methanol-d_4

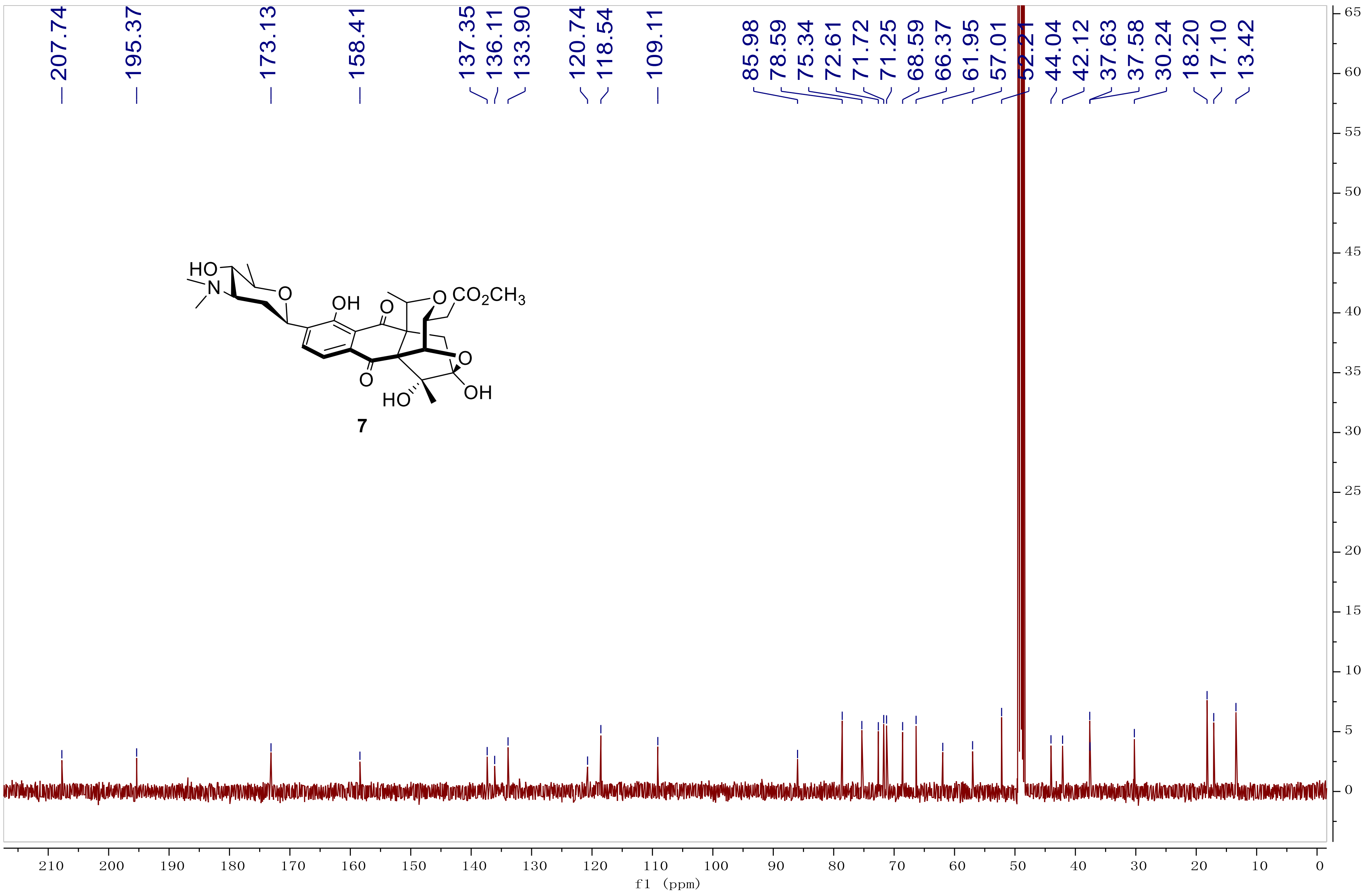

Supplementary Fig. 53. HSQC spectrum of chimedermycin G (**7**) in methanol-d_4

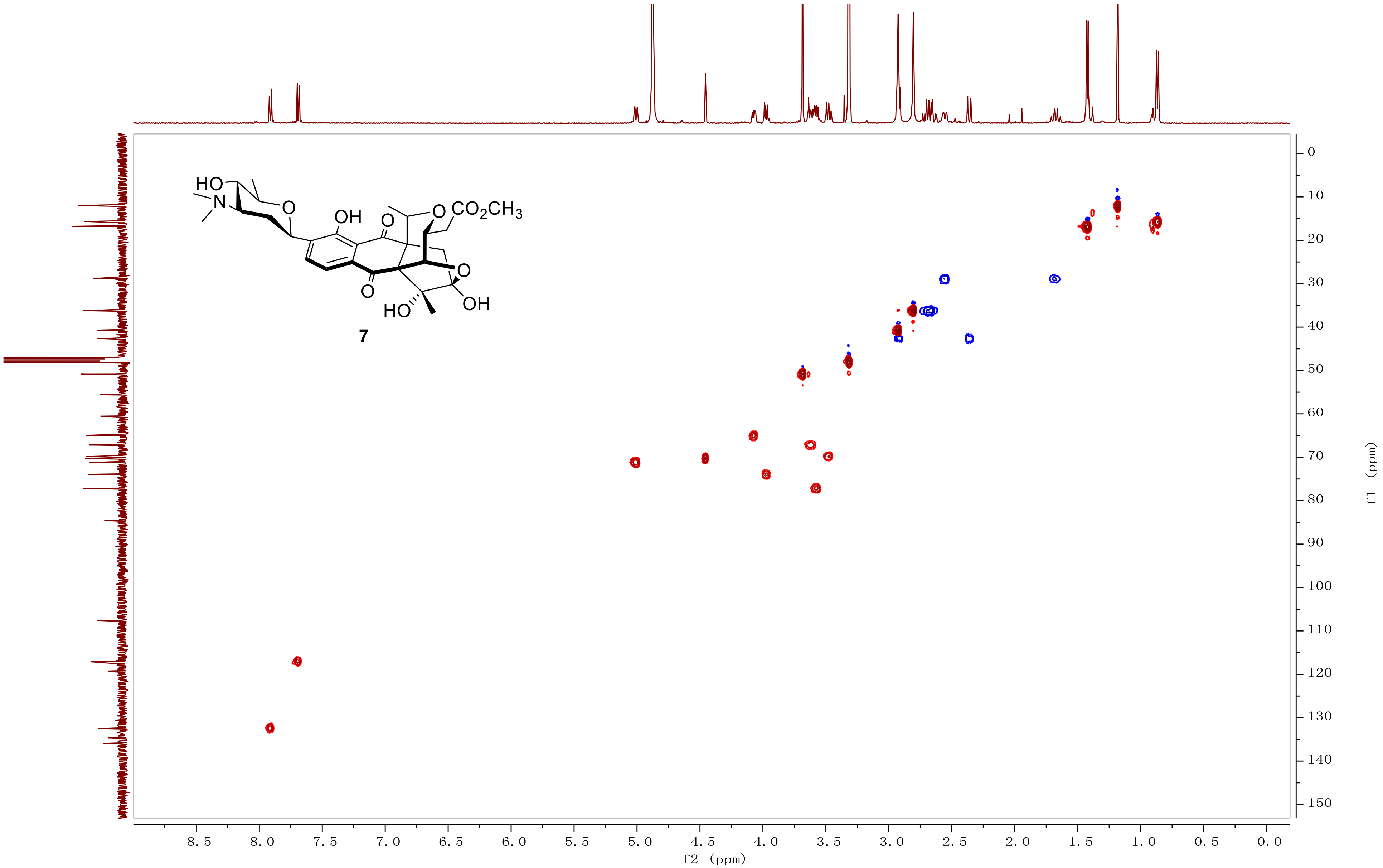

Supplementary Fig. 54. ^1H-^1H COSY spectrum of chimedermycin G (**7**) in methanol-d_4

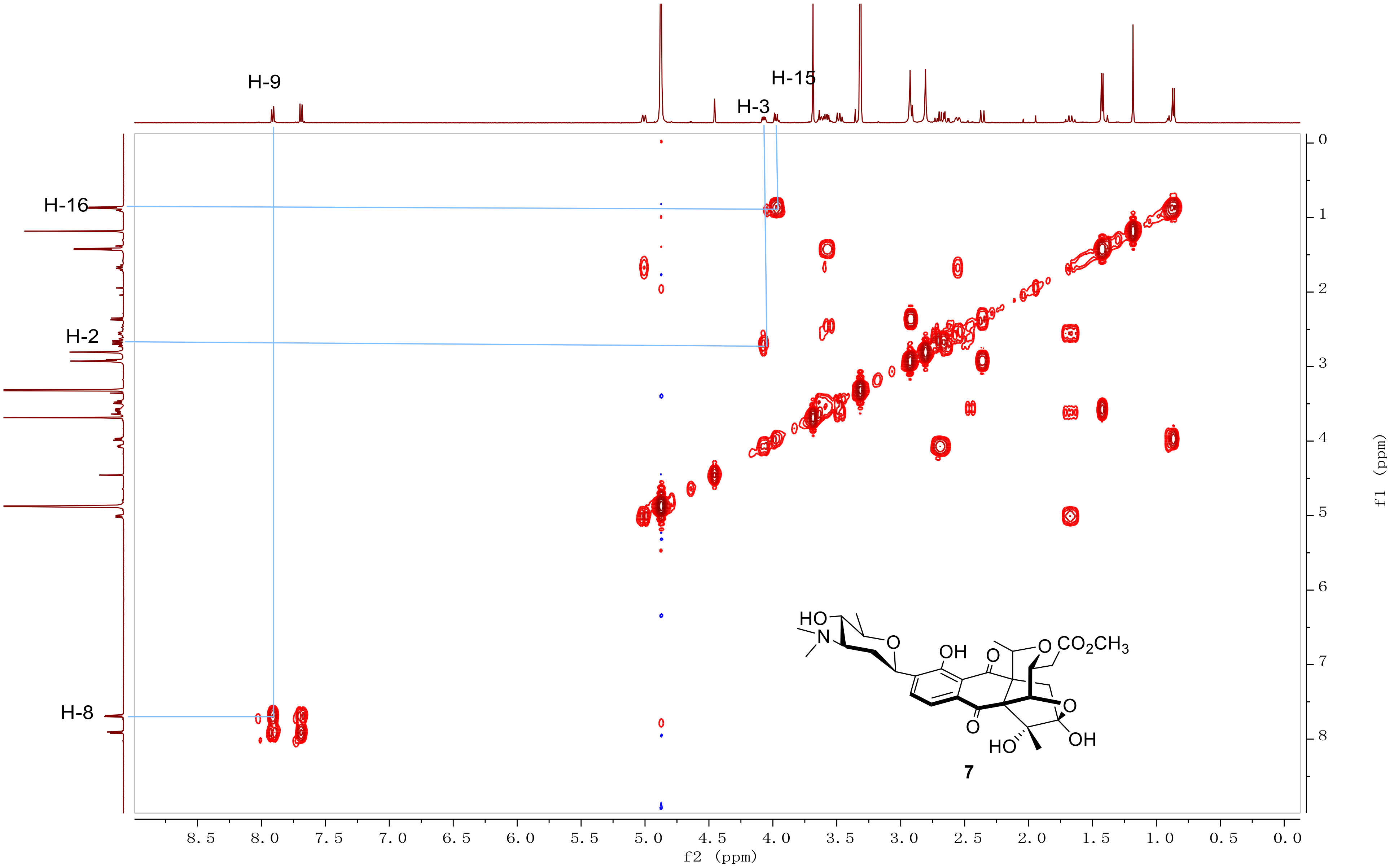

Supplementary Fig. 55. HMBC spectrum of chimedermycin G (**7**) in methanol-d_4

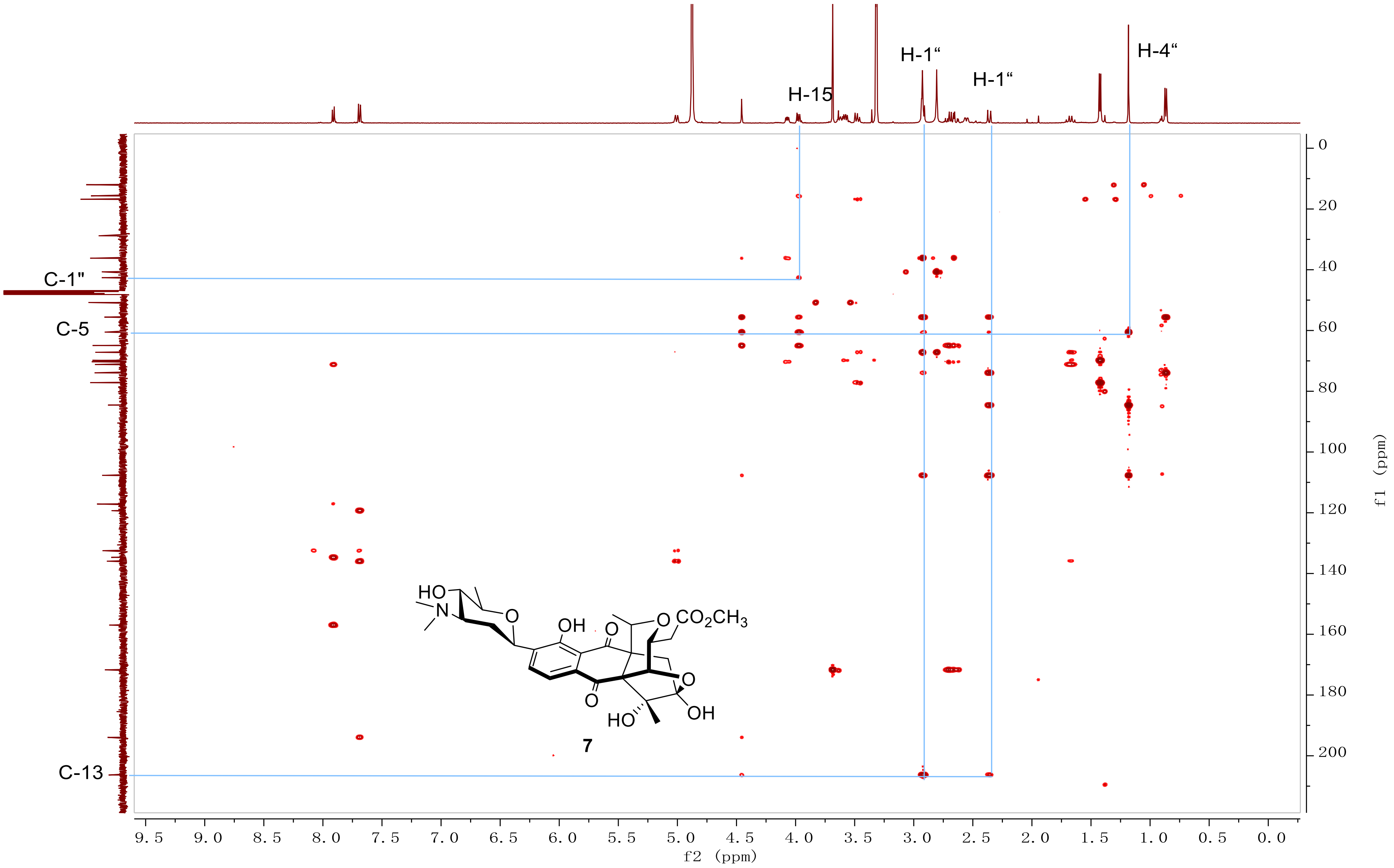

Supplementary Fig. 56. NOESY spectrum of chimedermycin G (**7**) in methanol-d_4

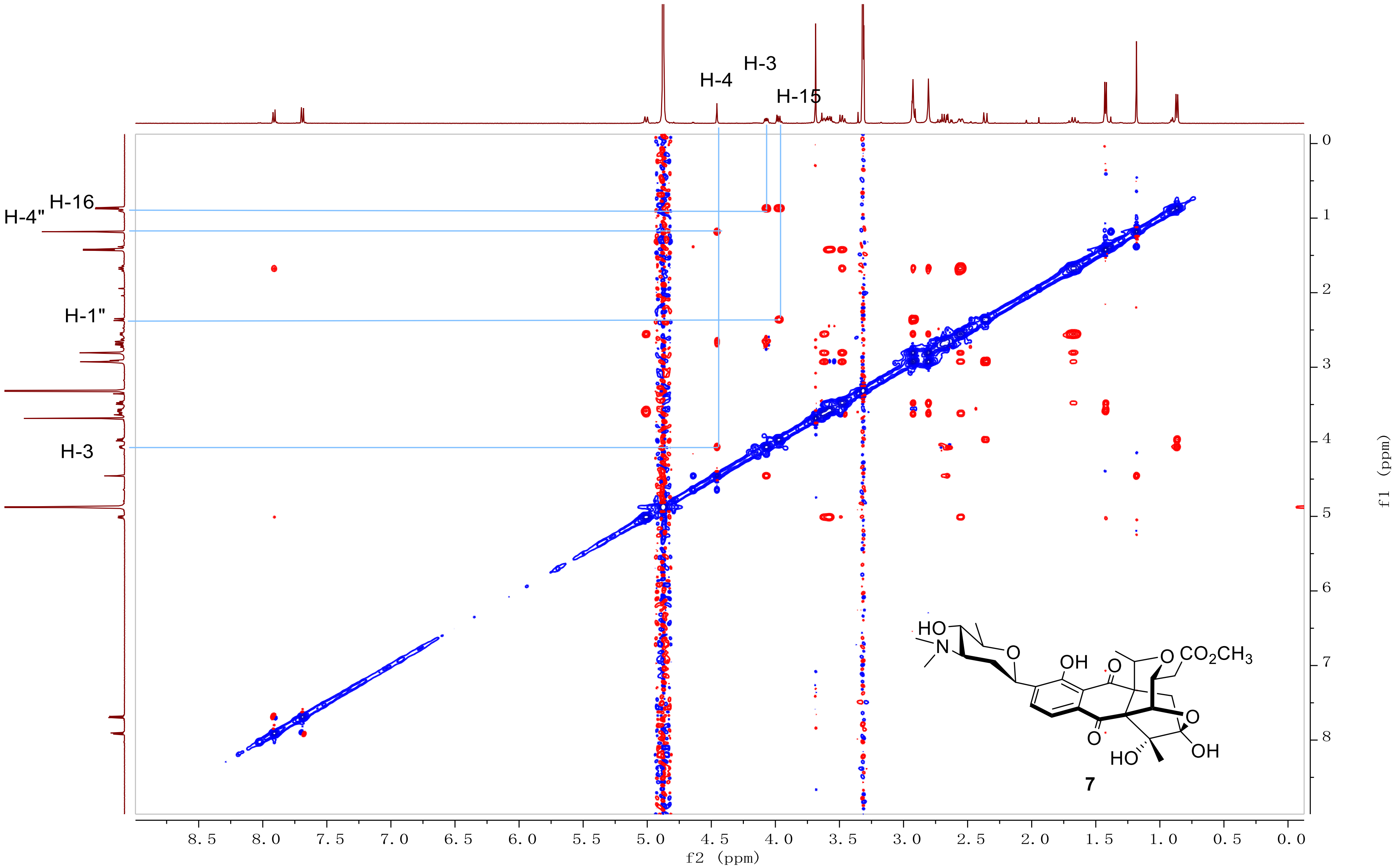

Supplementary Fig. 57. HRESIMS spectrum of chimedermycin H (**8**)

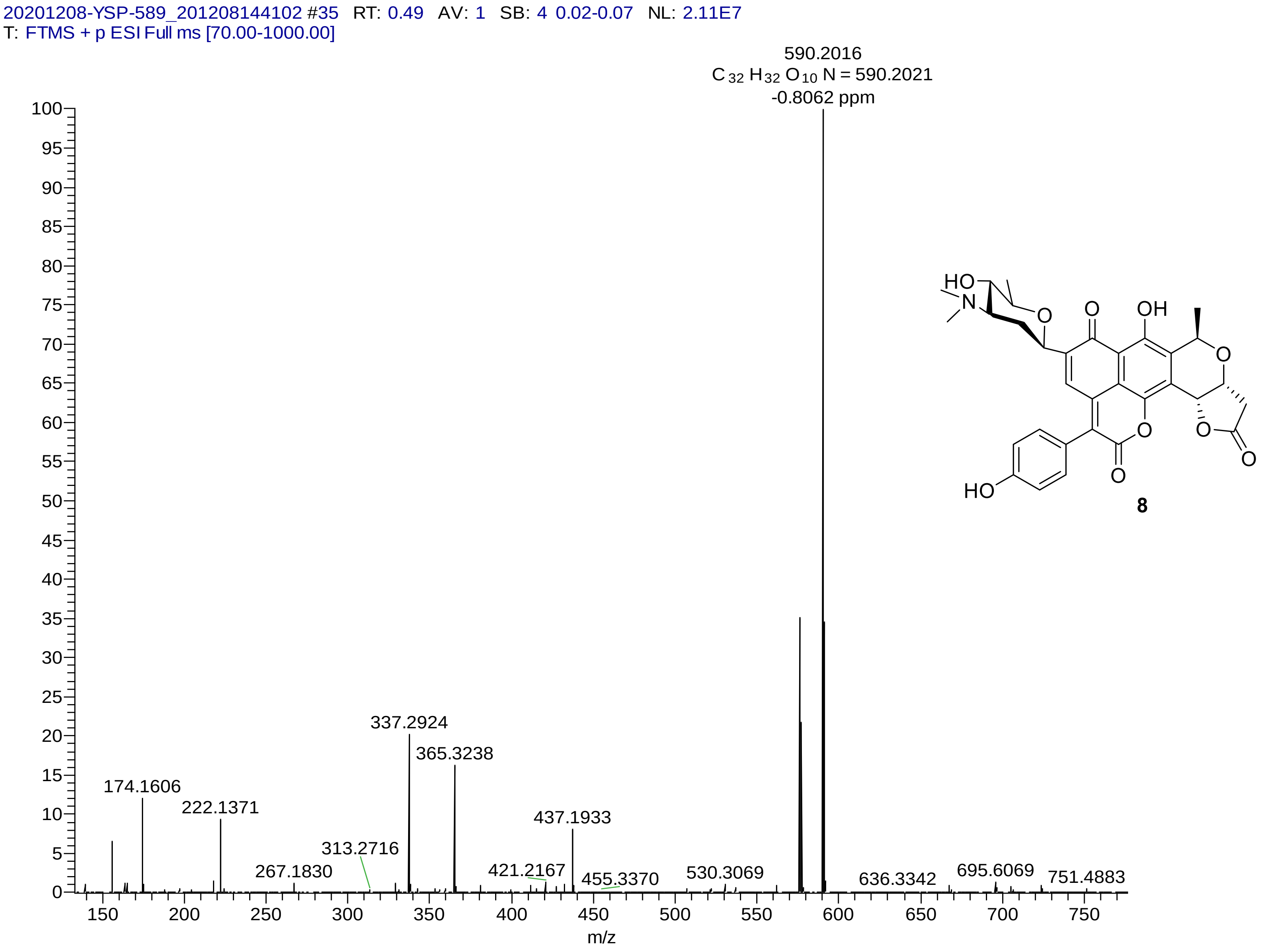

8

Supplementary Fig. 58. ^{1}H-NMR spectrum of chimedermycin H (**8**) in methanol-d_4

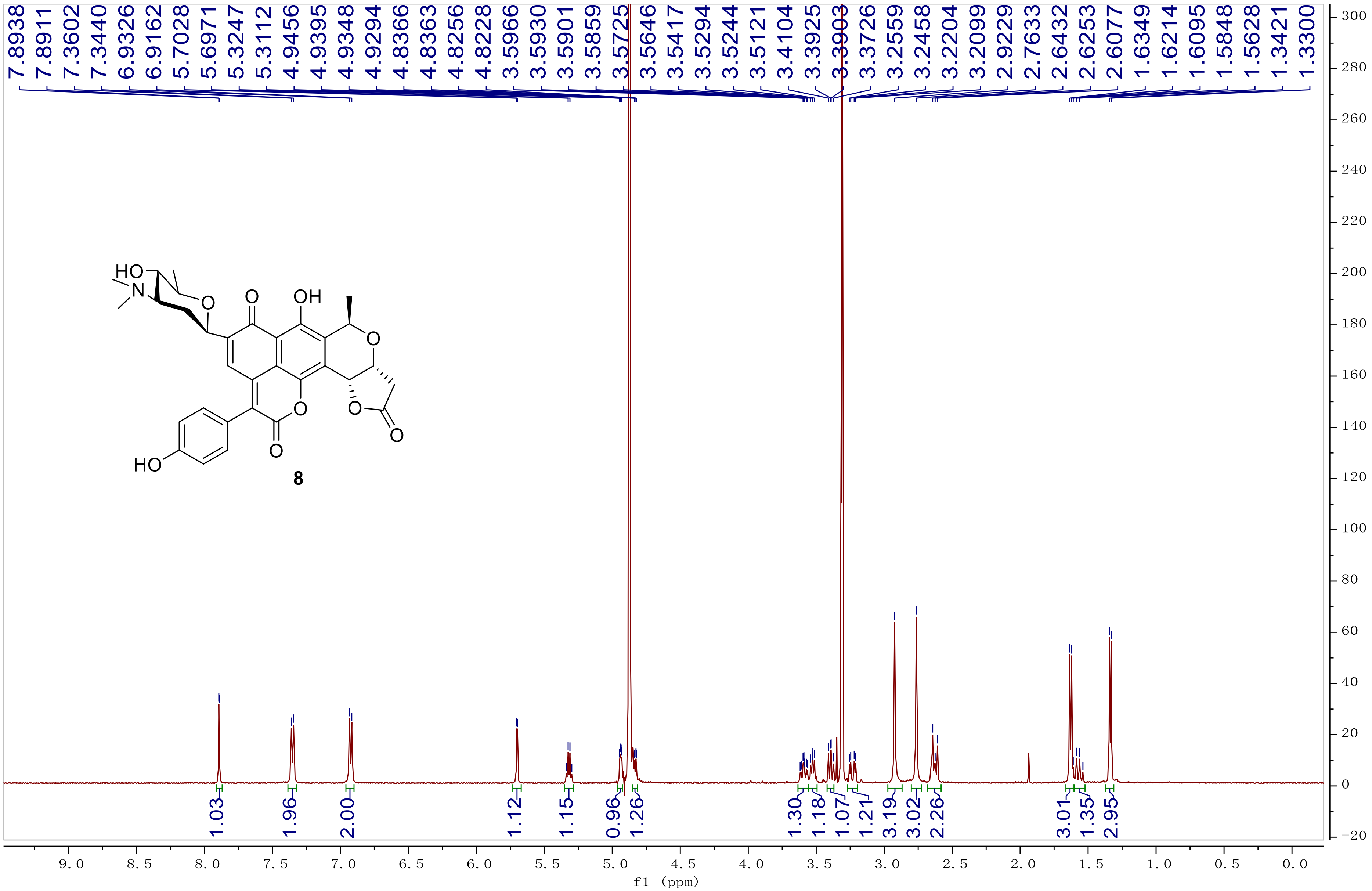

Supplementary Fig. 59. ^{13}C-NMR spectrum of chimedermycin H (**8**) in methanol-d_4

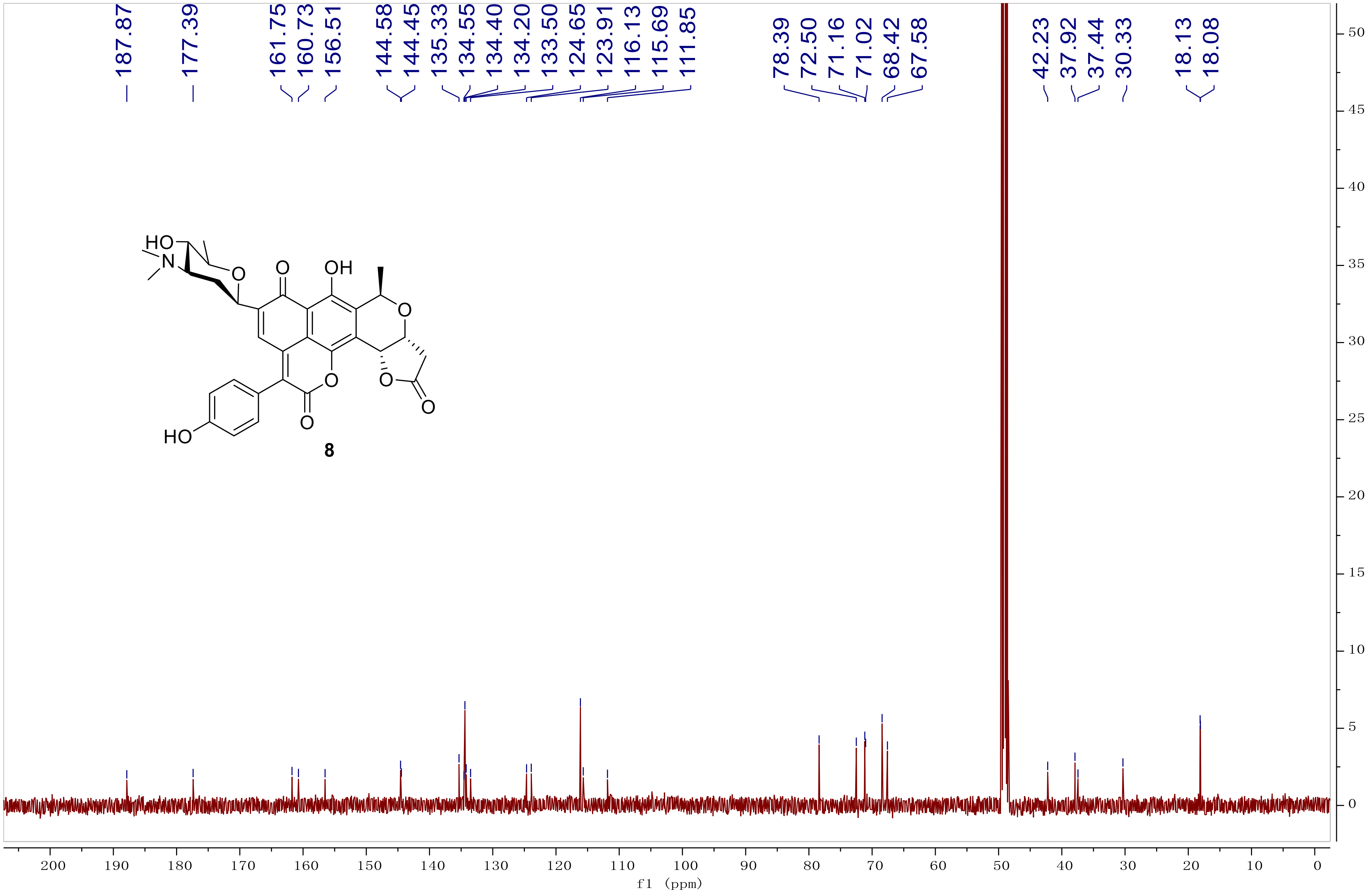

Supplementary Fig. 60. HSQC spectrum of chimedermycin H (**8**) in methanol-d_4

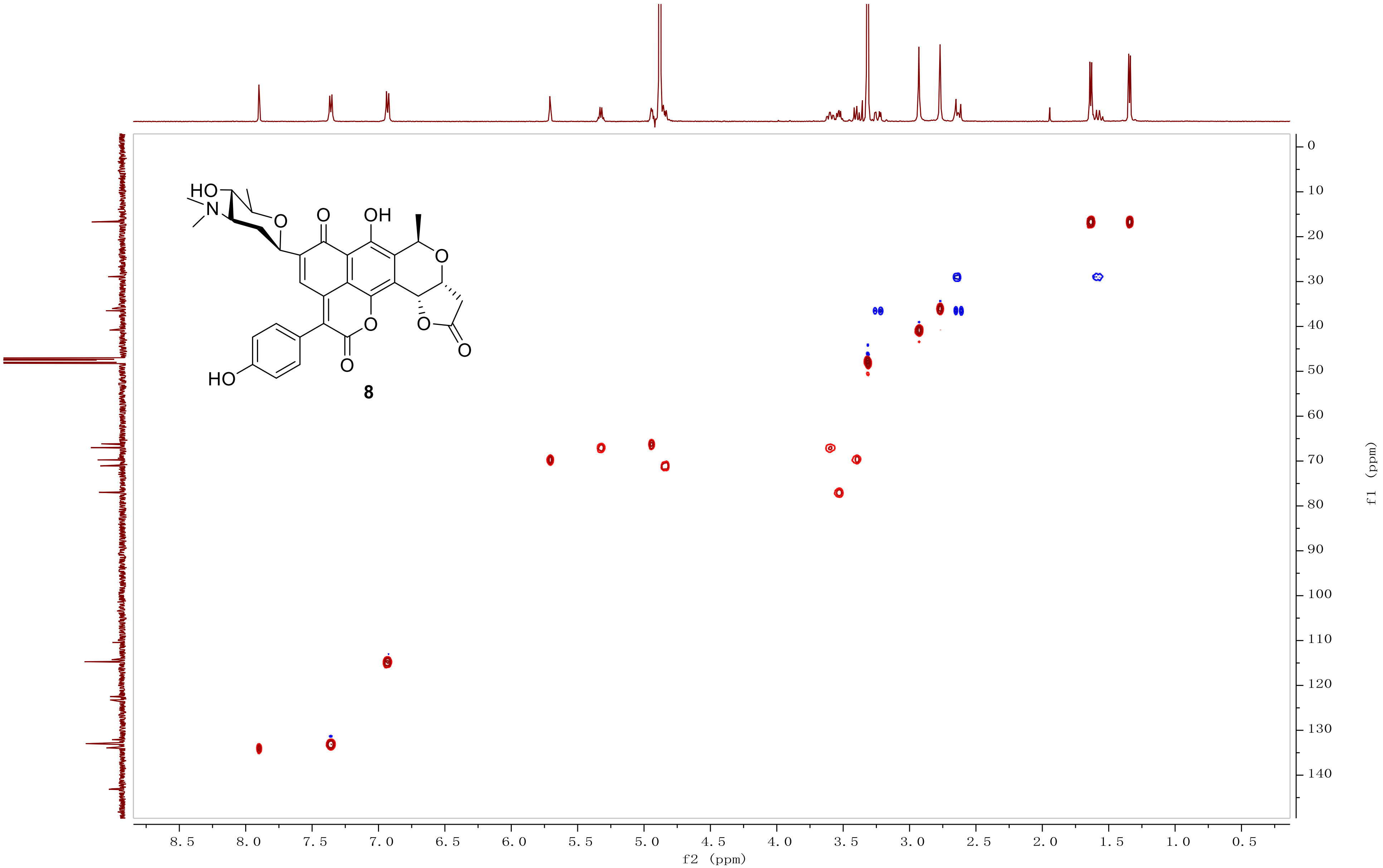

Supplementary Fig. 61. ^{1}H-^{1}H COSY spectrum of chimedermycin H (**8**) in methanol-d_4

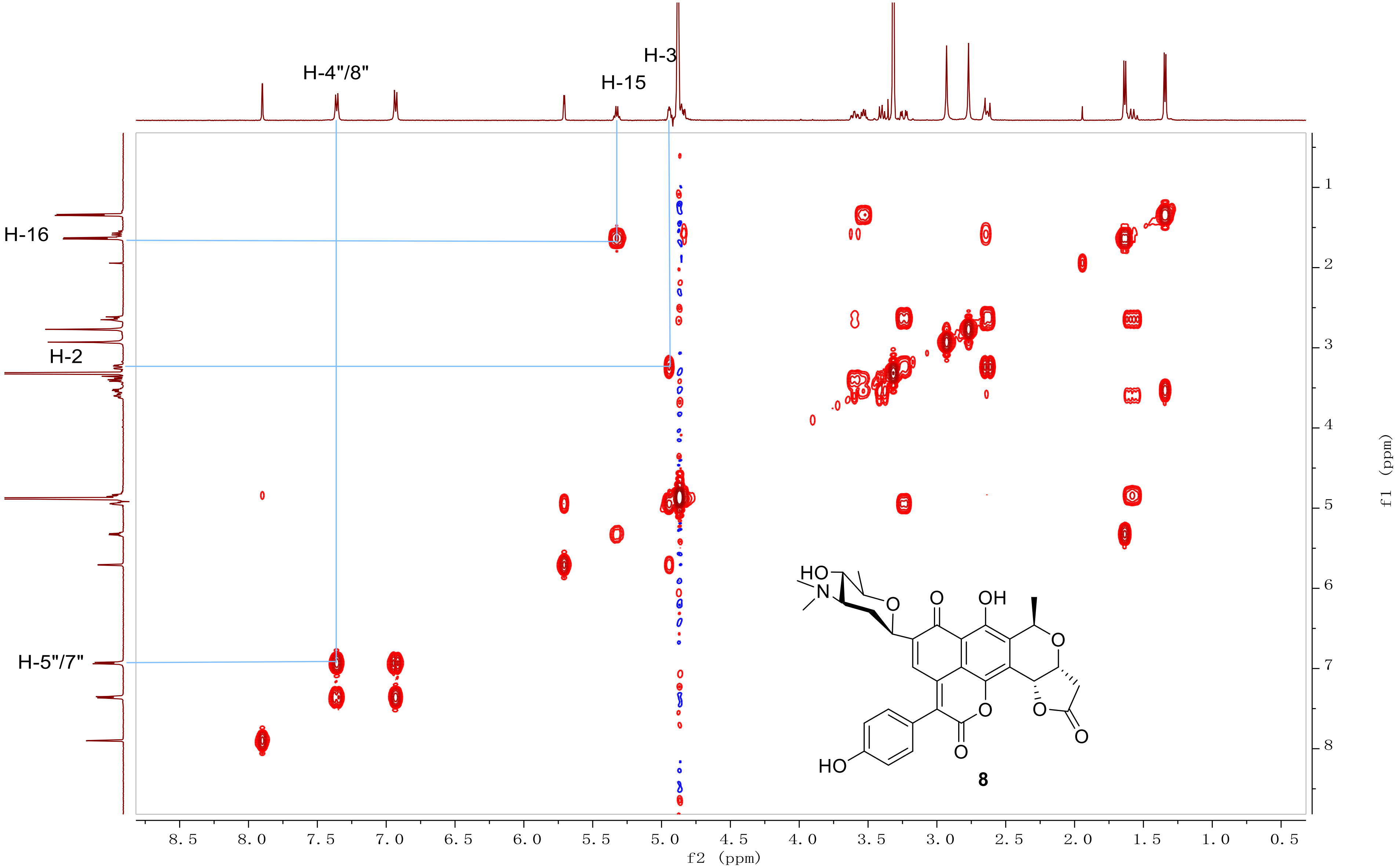

Supplementary Fig. 62. HMBC spectrum of chimedermycin H (**8**) in methanol-d_4

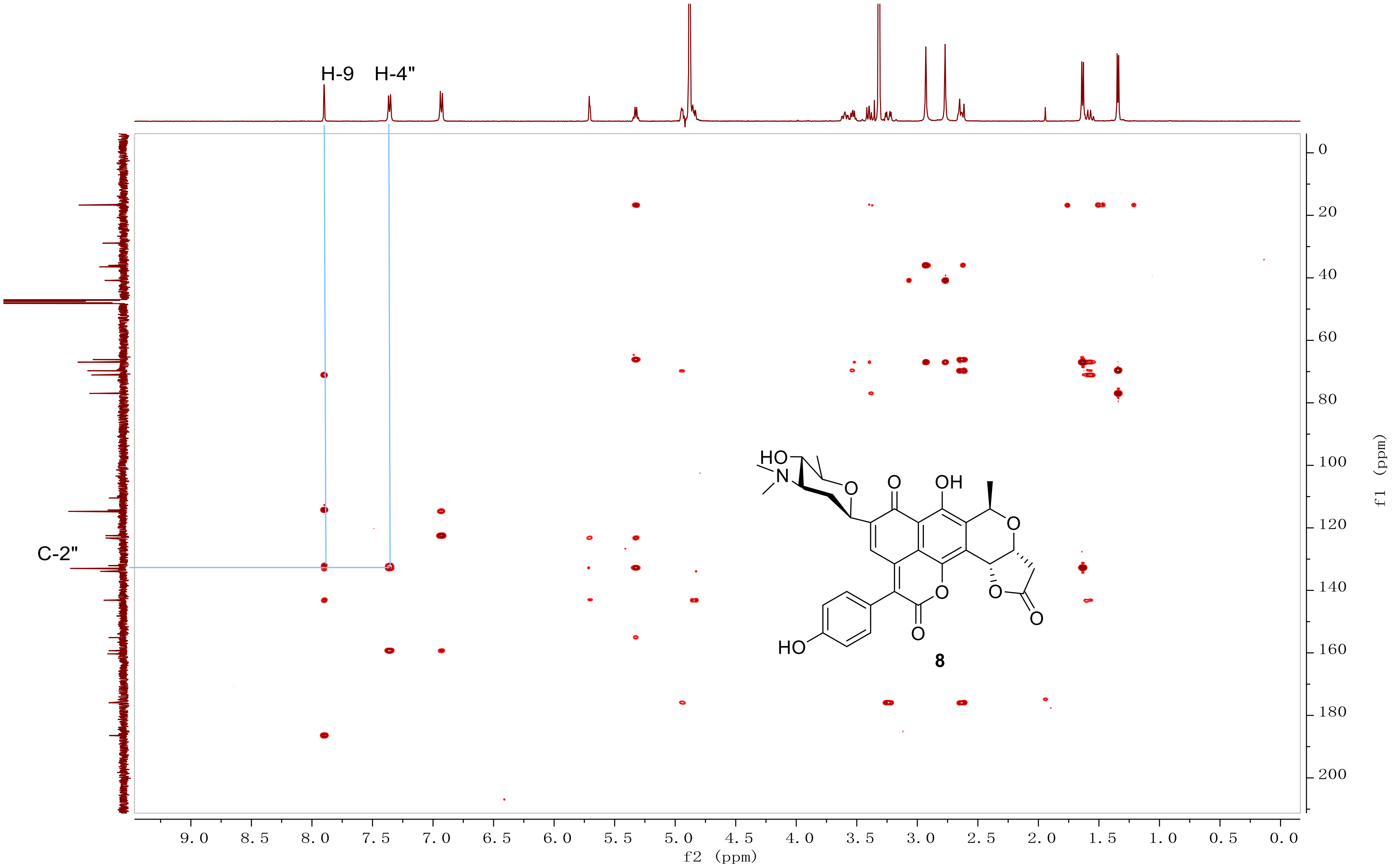

Supplementary Fig. 63. NOESY spectrum of chimedermycin H (**8**) in methanol-d_4

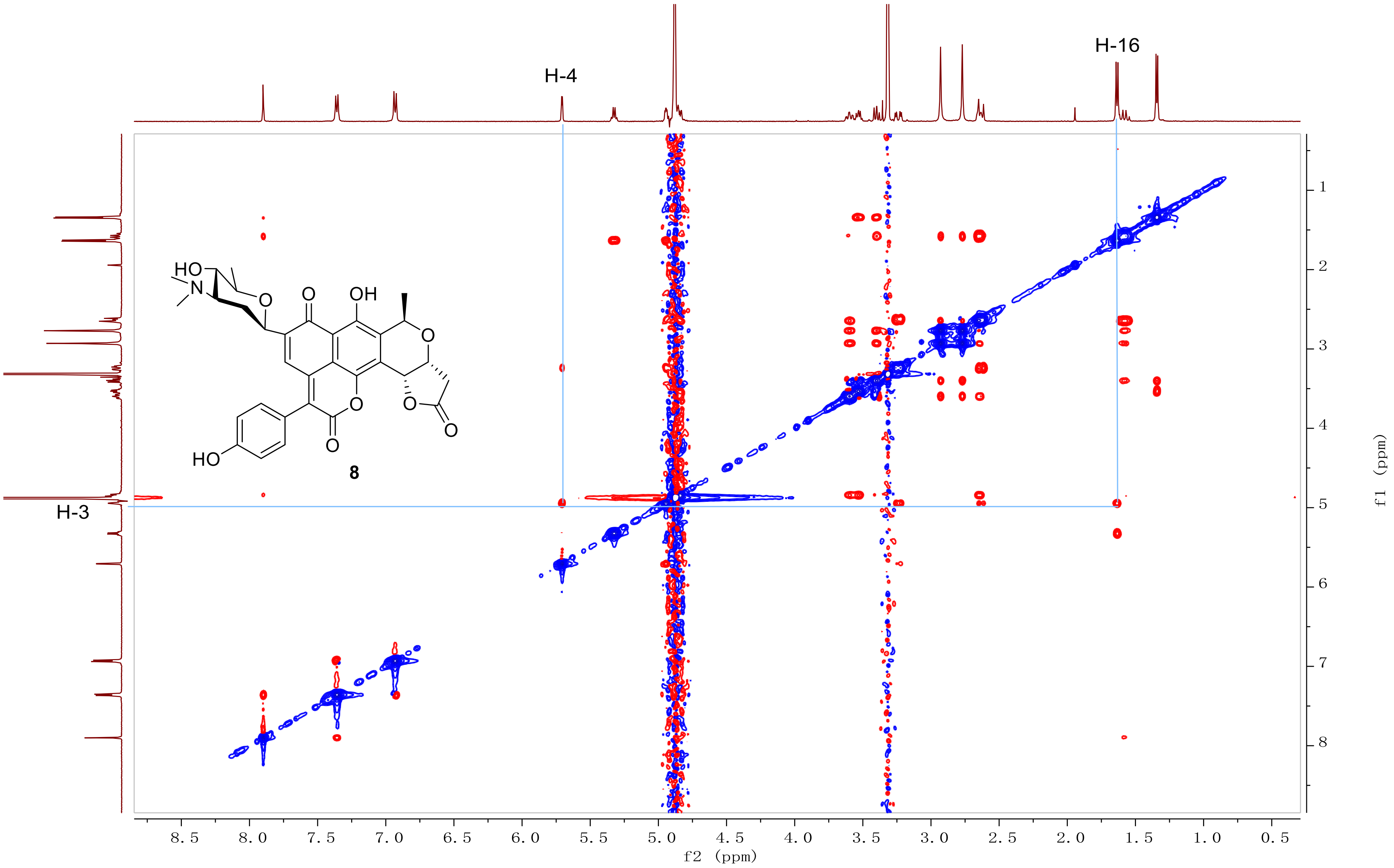

Supplementary Fig. 64. HRESIMS spectrum of demethylmenoxymycin B (**11**)

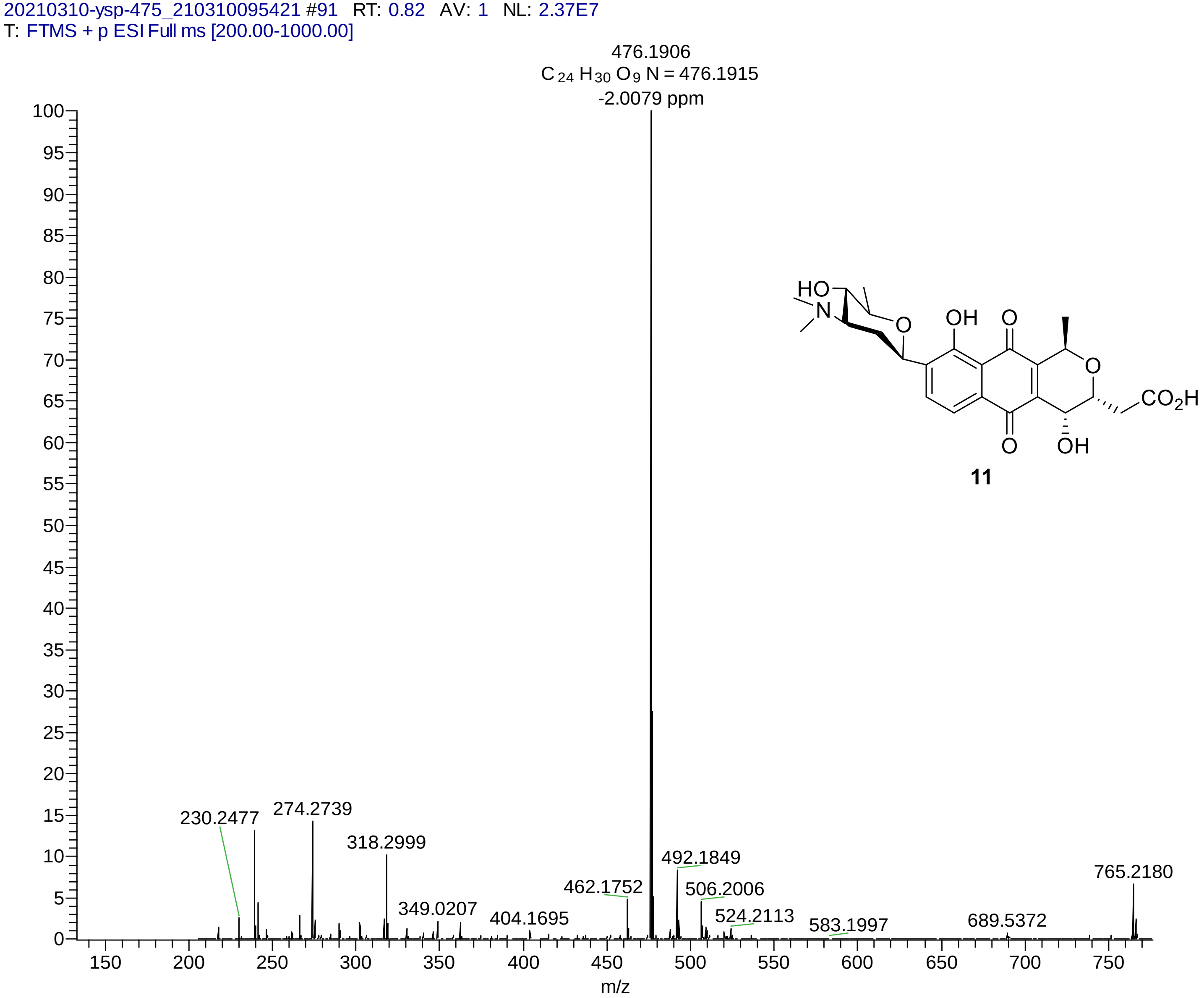

11

Supplementary Fig. 65. ^{1}H-NMR spectrum of demethylmenoxymycin B (**11**) in methanol-d_4

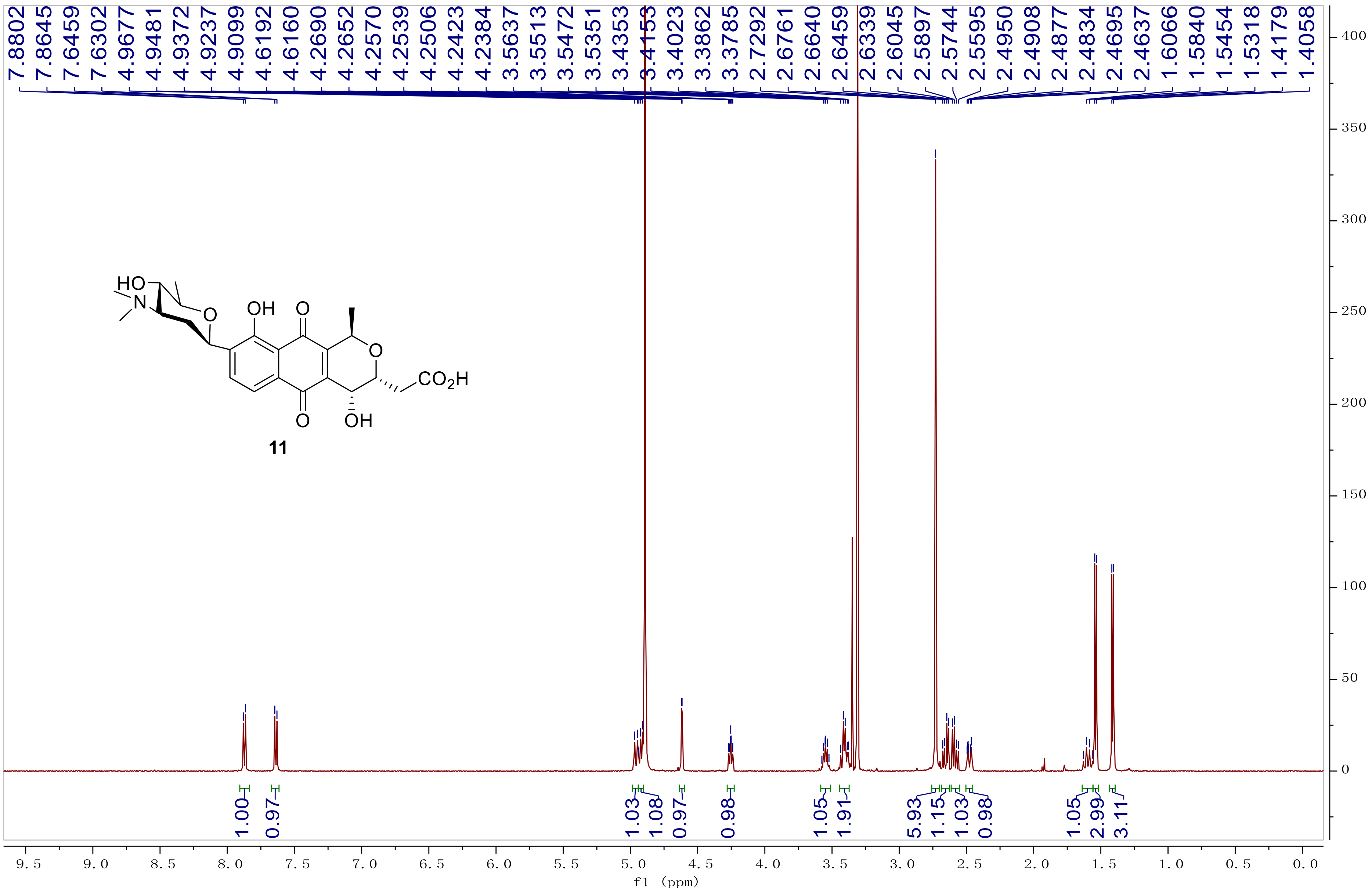

Supplementary Fig. 66. ^{13}C-NMR spectrum of demethylmenoxymycin B (**11**) in methanol-d_4

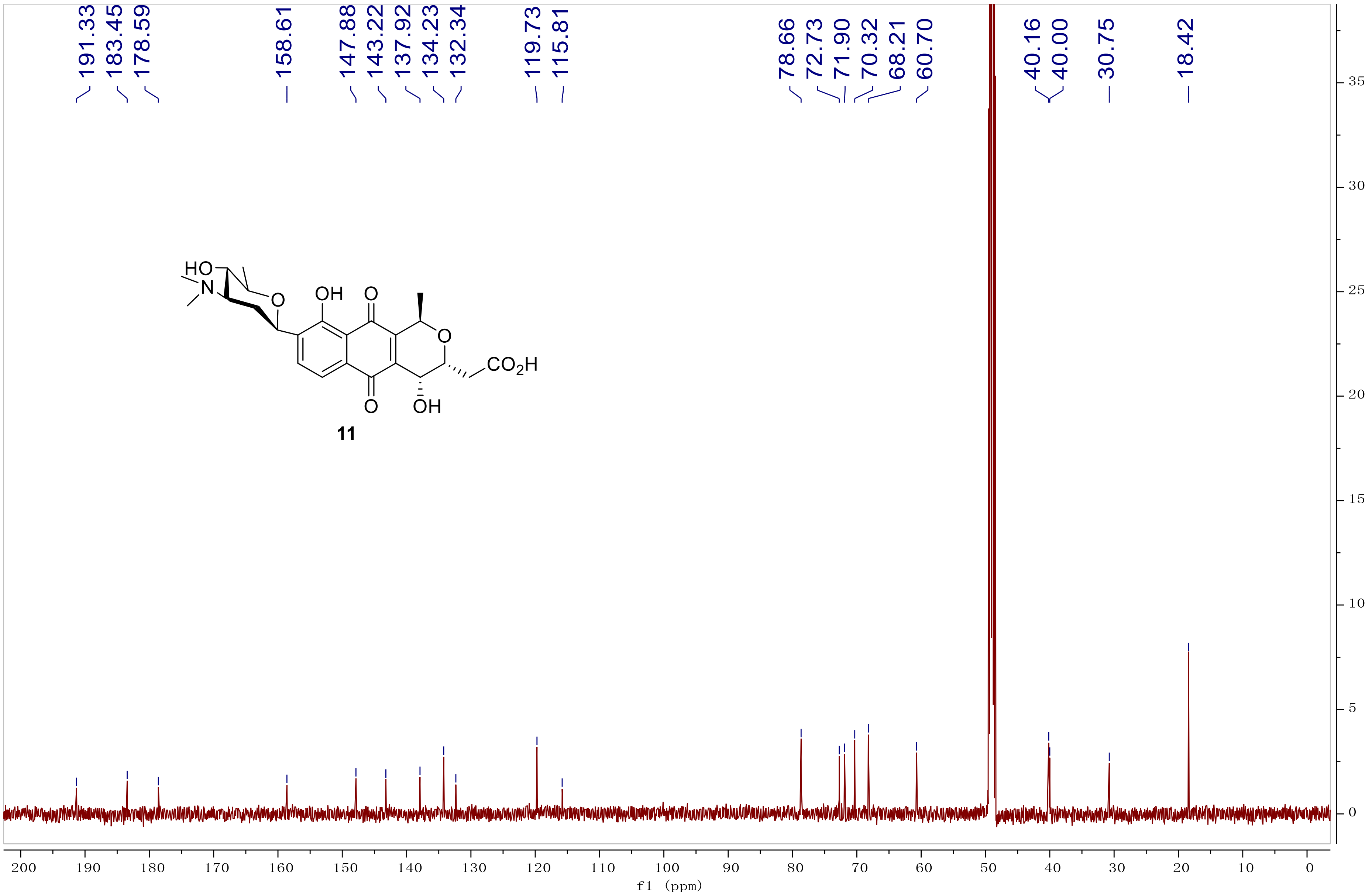

Supplementary Fig. 67. HSQC spectrum of demethylmenoxymycin B (**11**) in methanol-d_4

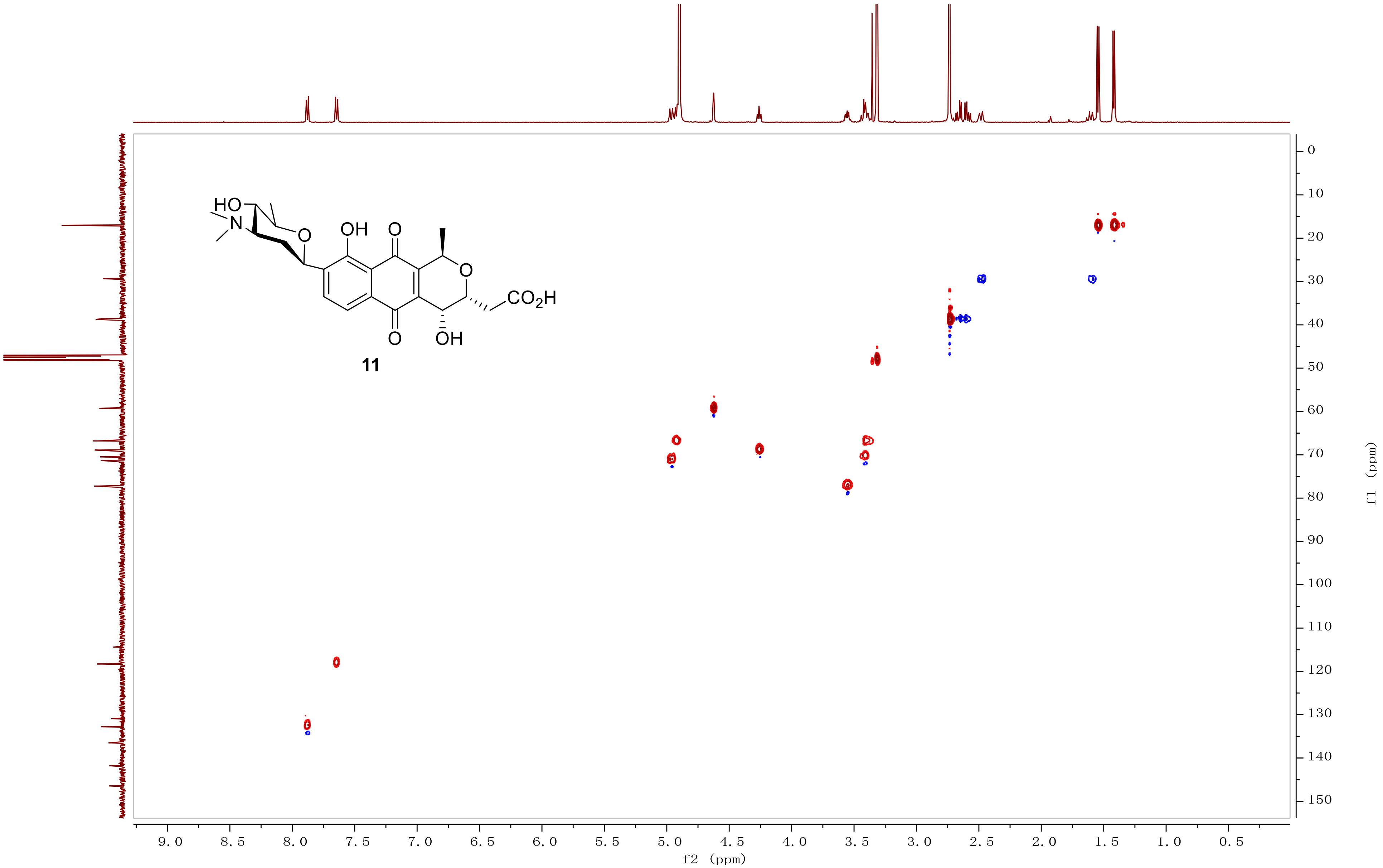

Supplementary Fig. 68. ^{1}H-^{1}H COSY spectrum of demethylmenoxymycin B (**11**) in methanol-*d*4

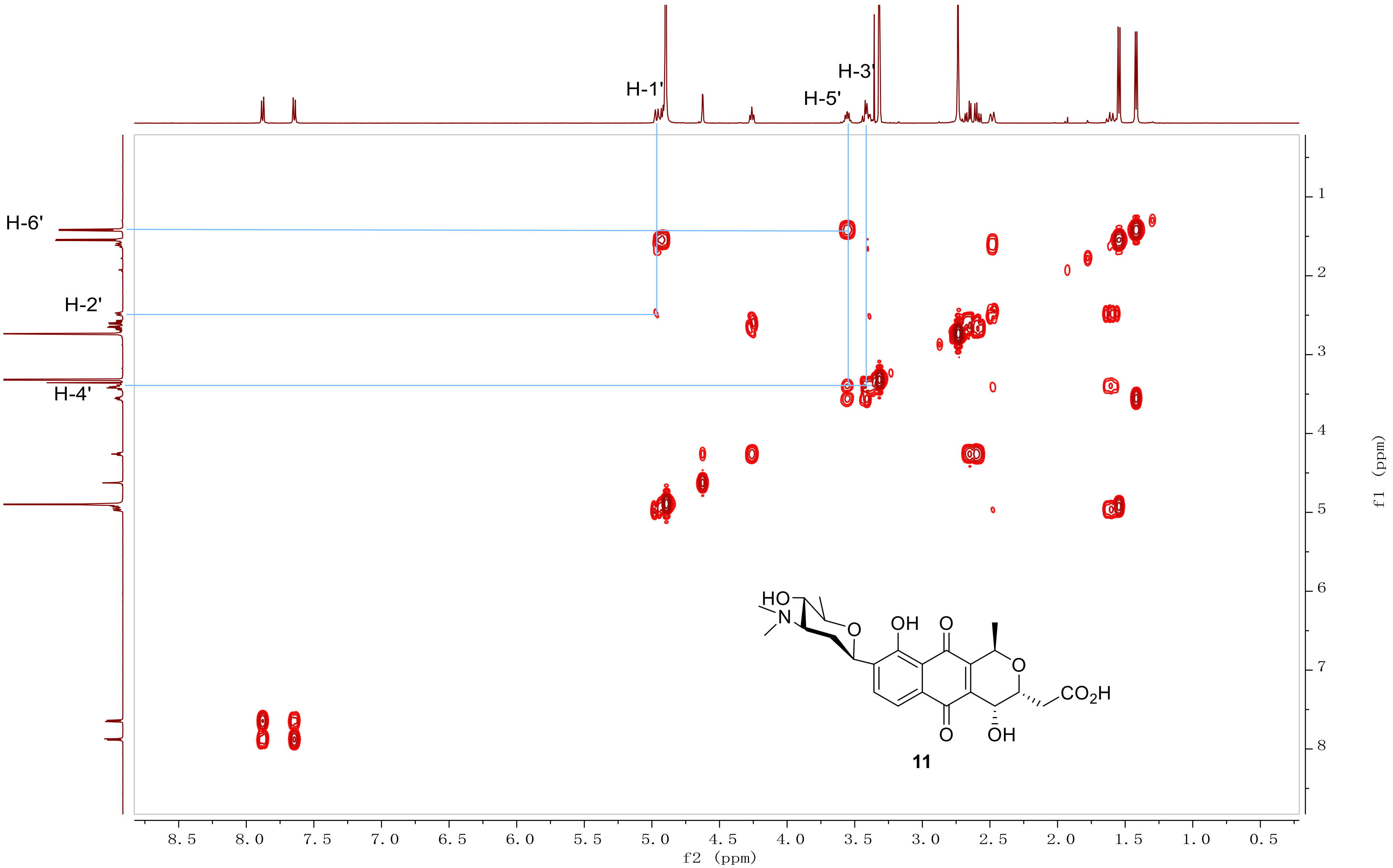

Supplementary Fig. 69. HMBC spectrum of demethylmenoxymycin B (**11**) in methanol-d_4

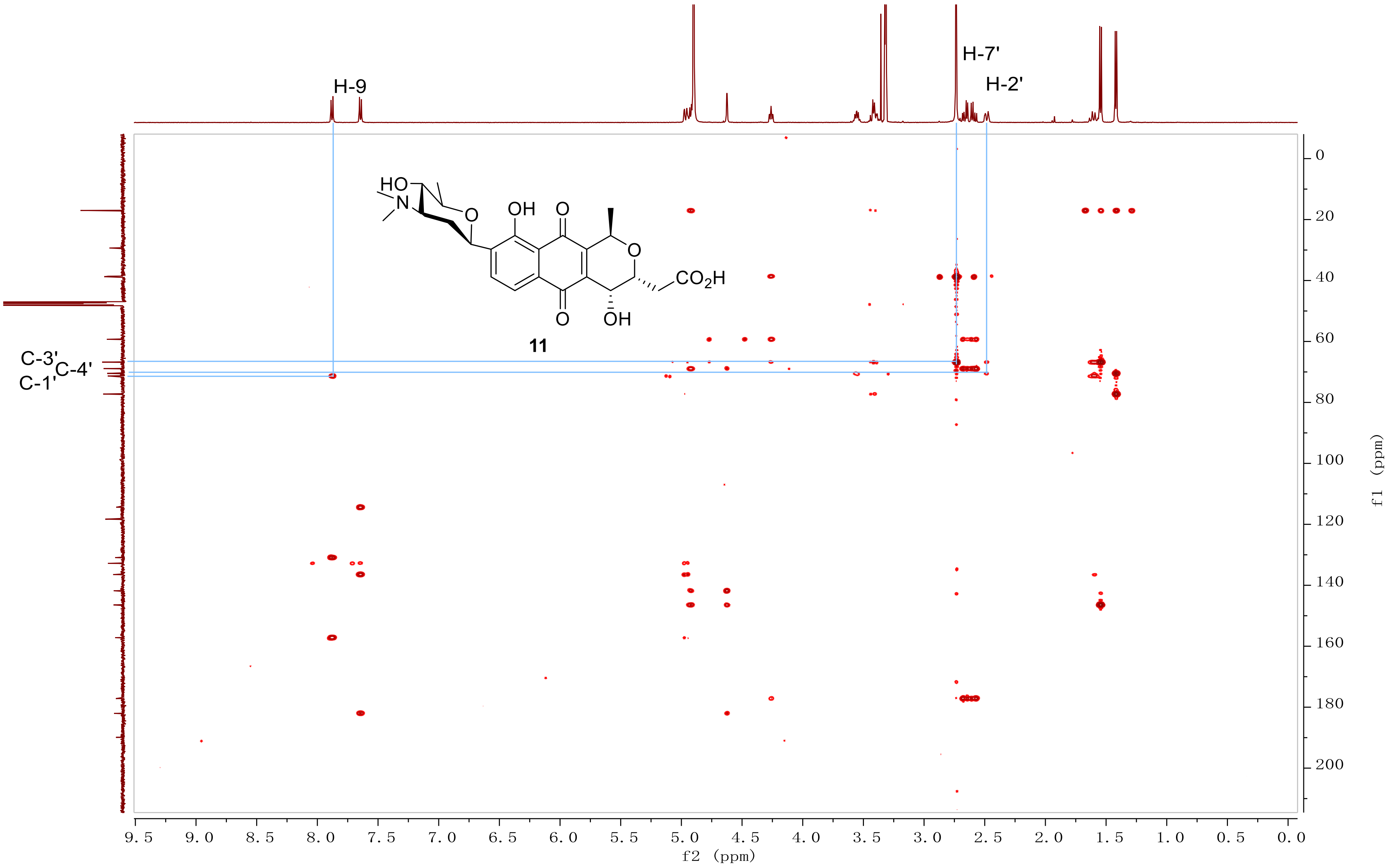

Supplementary Fig. 70. HRESIMS spectrum of chimedermycin I (**15**)

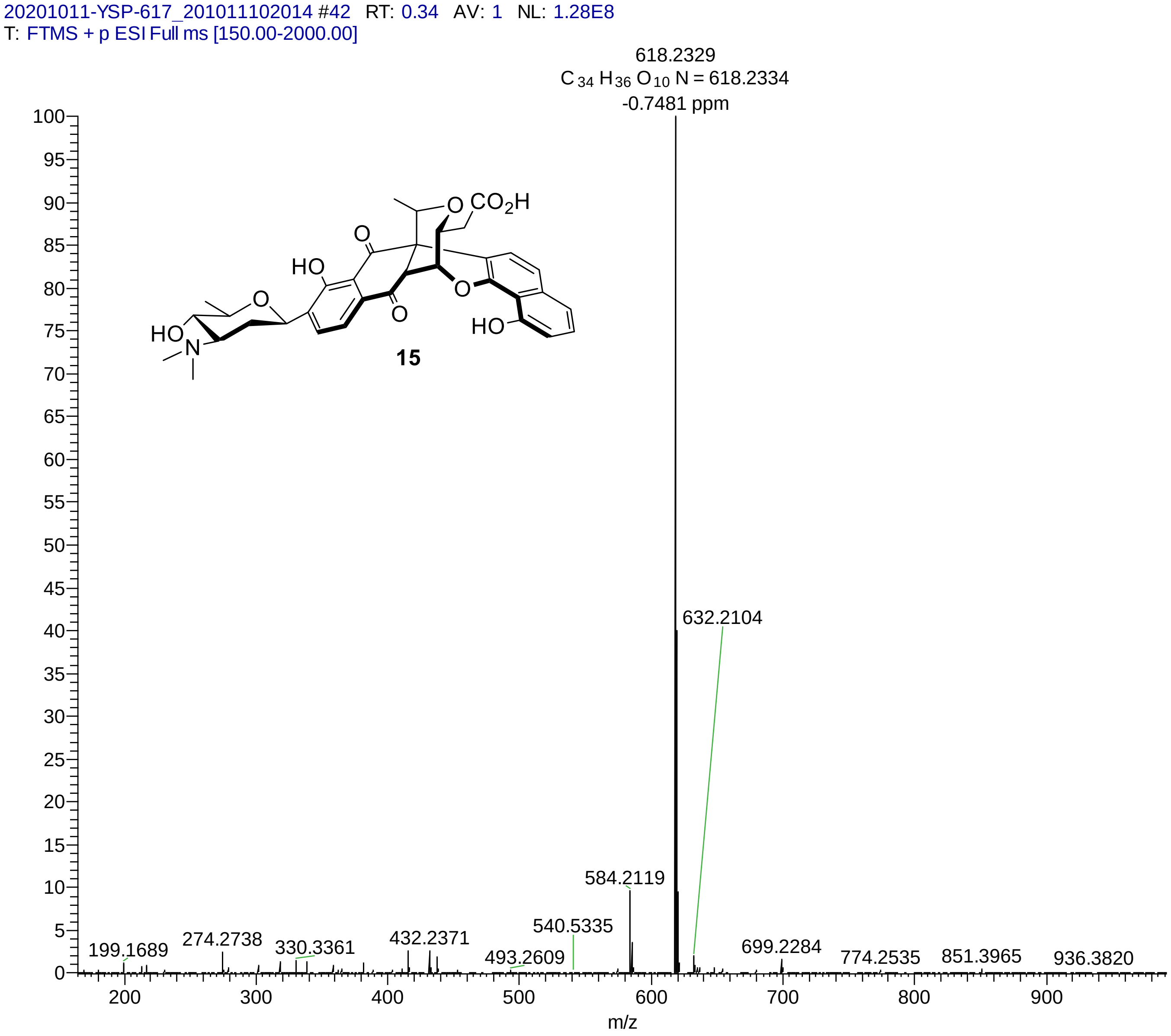

Supplementary Fig. 71. ^{1}H-NMR spectrum of chimedermycin I (**15**) in methanol-d_4

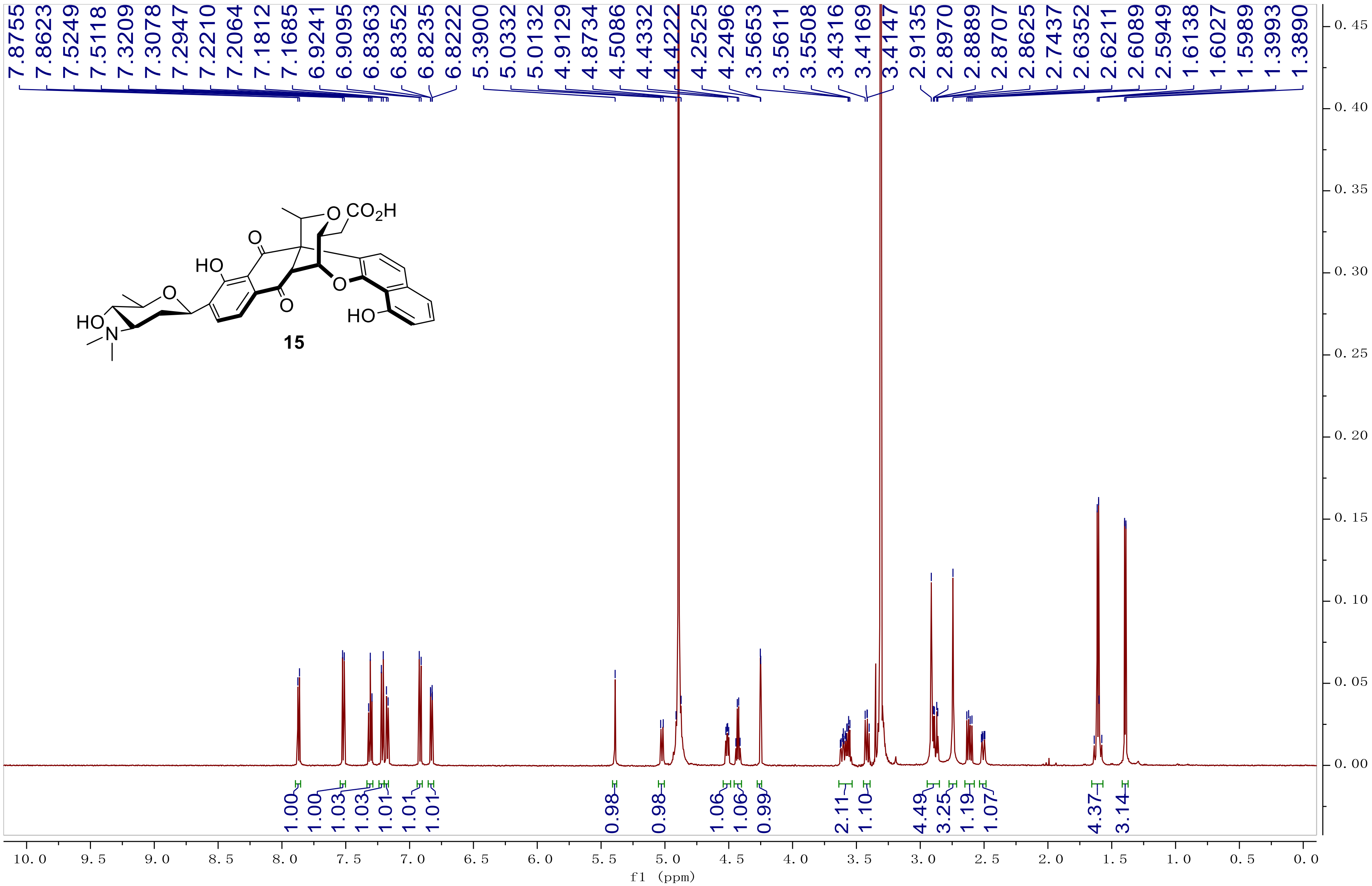

Supplementary Fig. 72. ^{13}C-NMR spectrum of chimedermycin I (**15**) in methanol-d_4

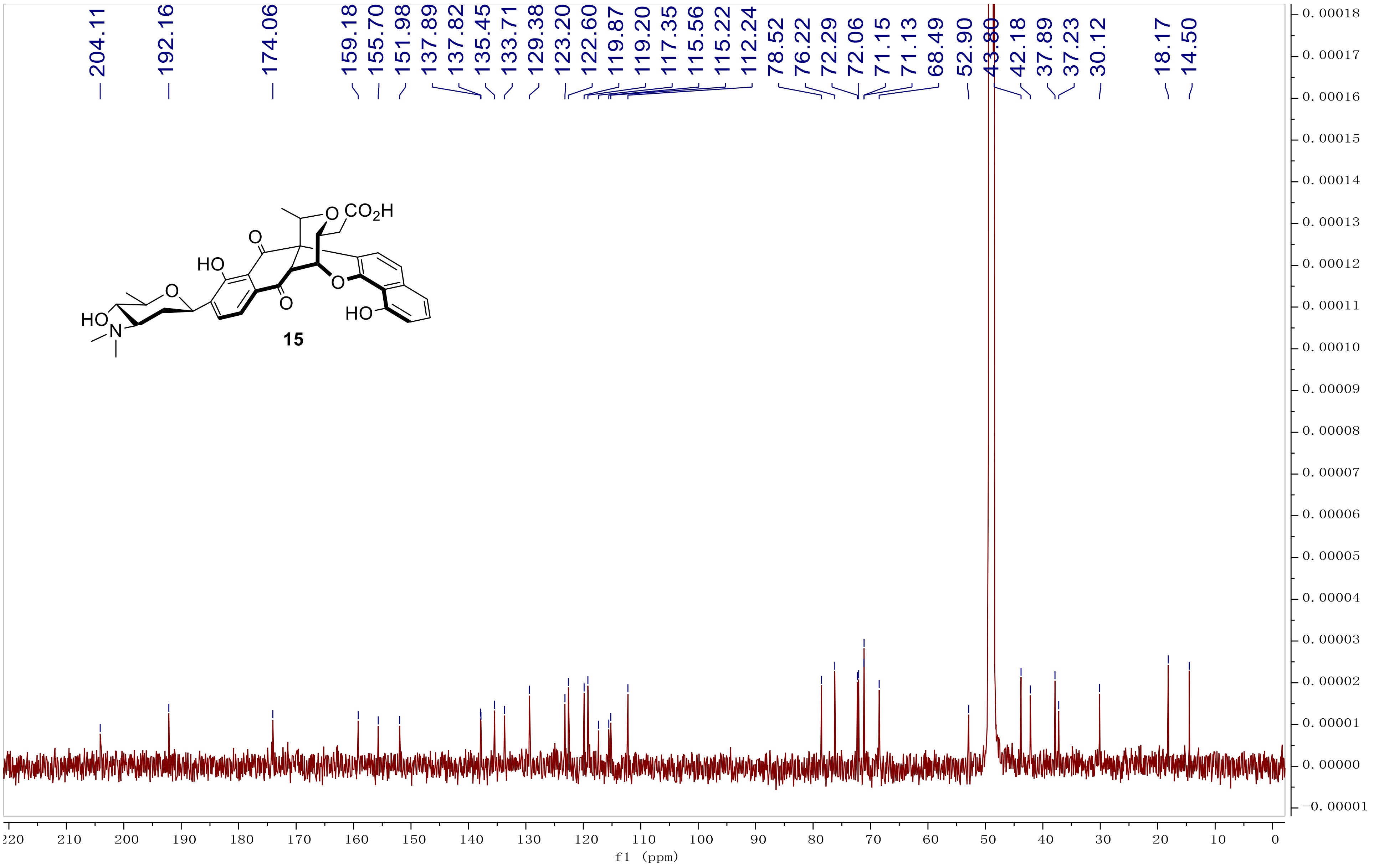

Supplementary Fig. 73. HSQC spectrum of chimedermycin I (**15**) in methanol-d_4

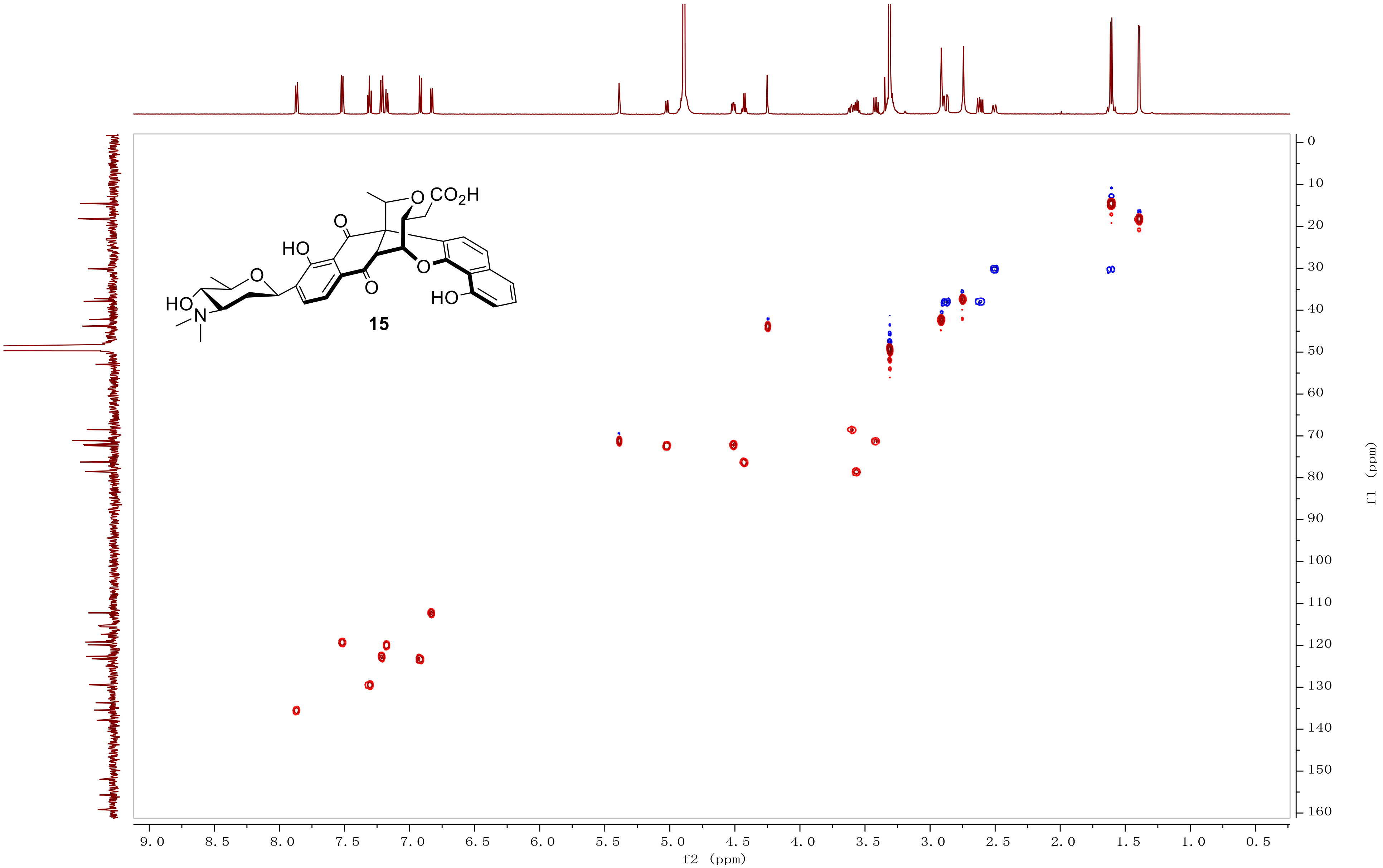

Supplementary Fig. 74. ^{1}H-^{1}H COSY spectrum of chimedermycin I (**15**) in methanol-d_4

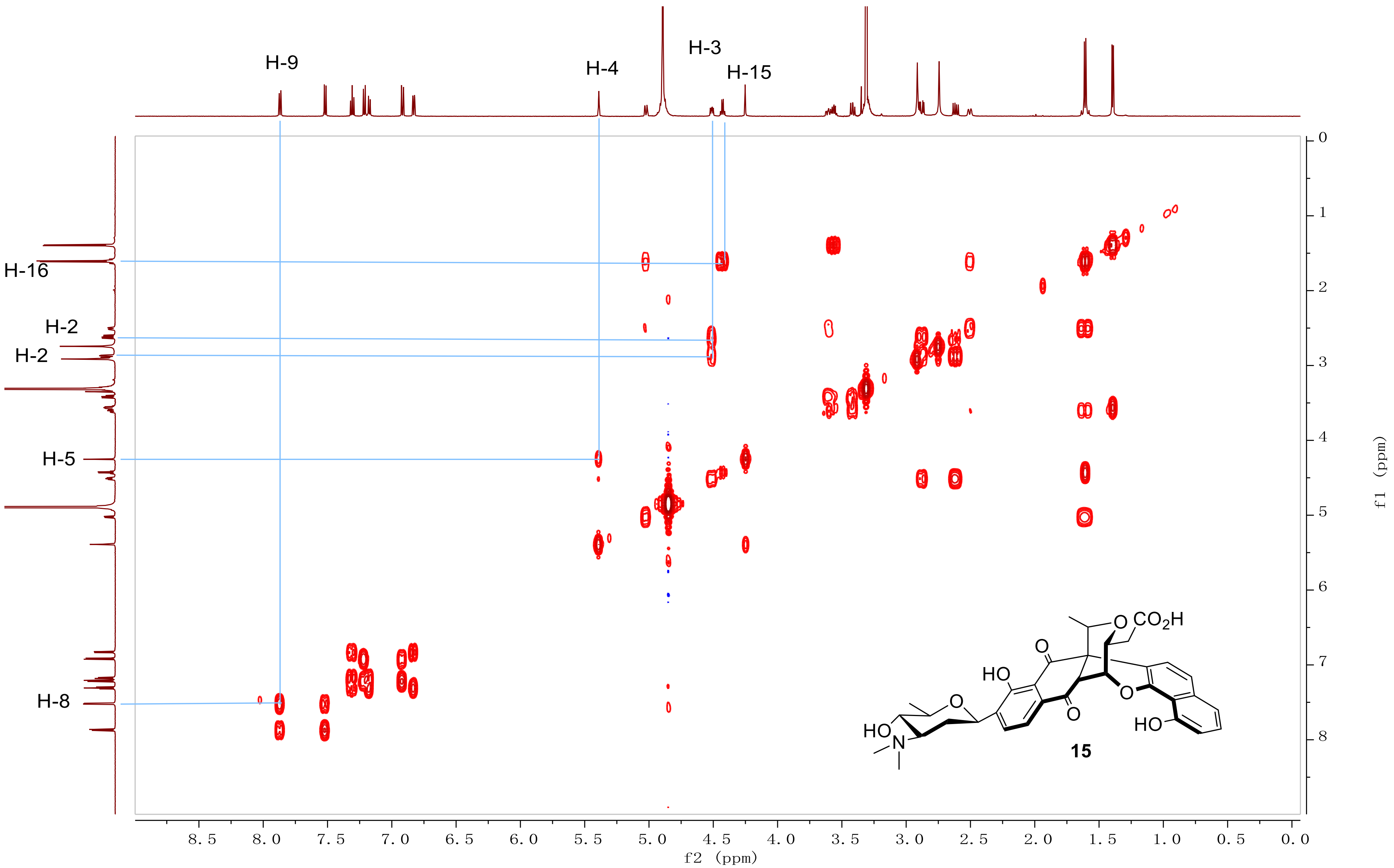

Supplementary Fig. 75. HMBC spectrum of chimedermycin I (**15**) in methanol-d_4

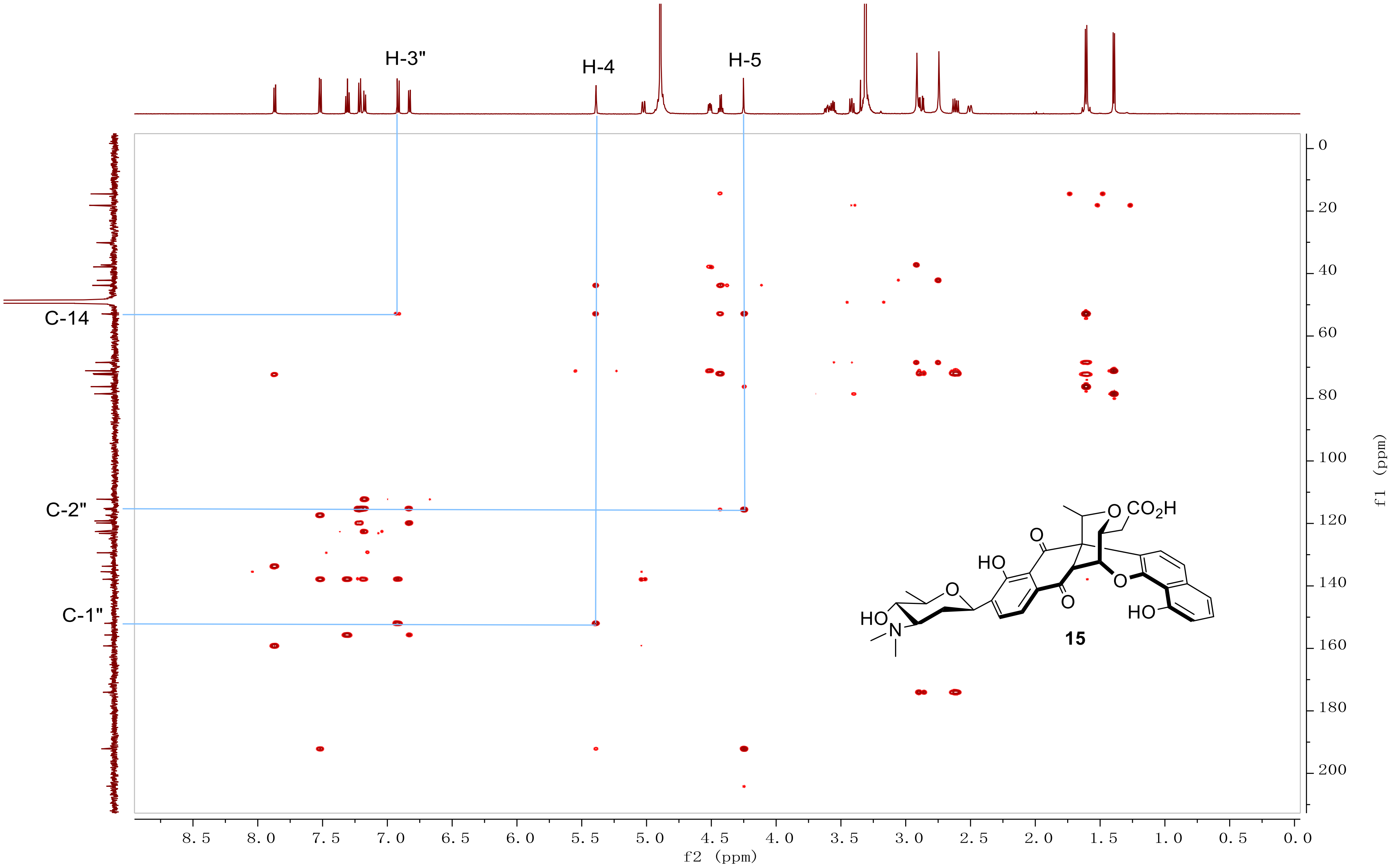

Supplementary Fig. 76. NOESY spectrum of chimedermycin I (**15**) in methanol-d_4

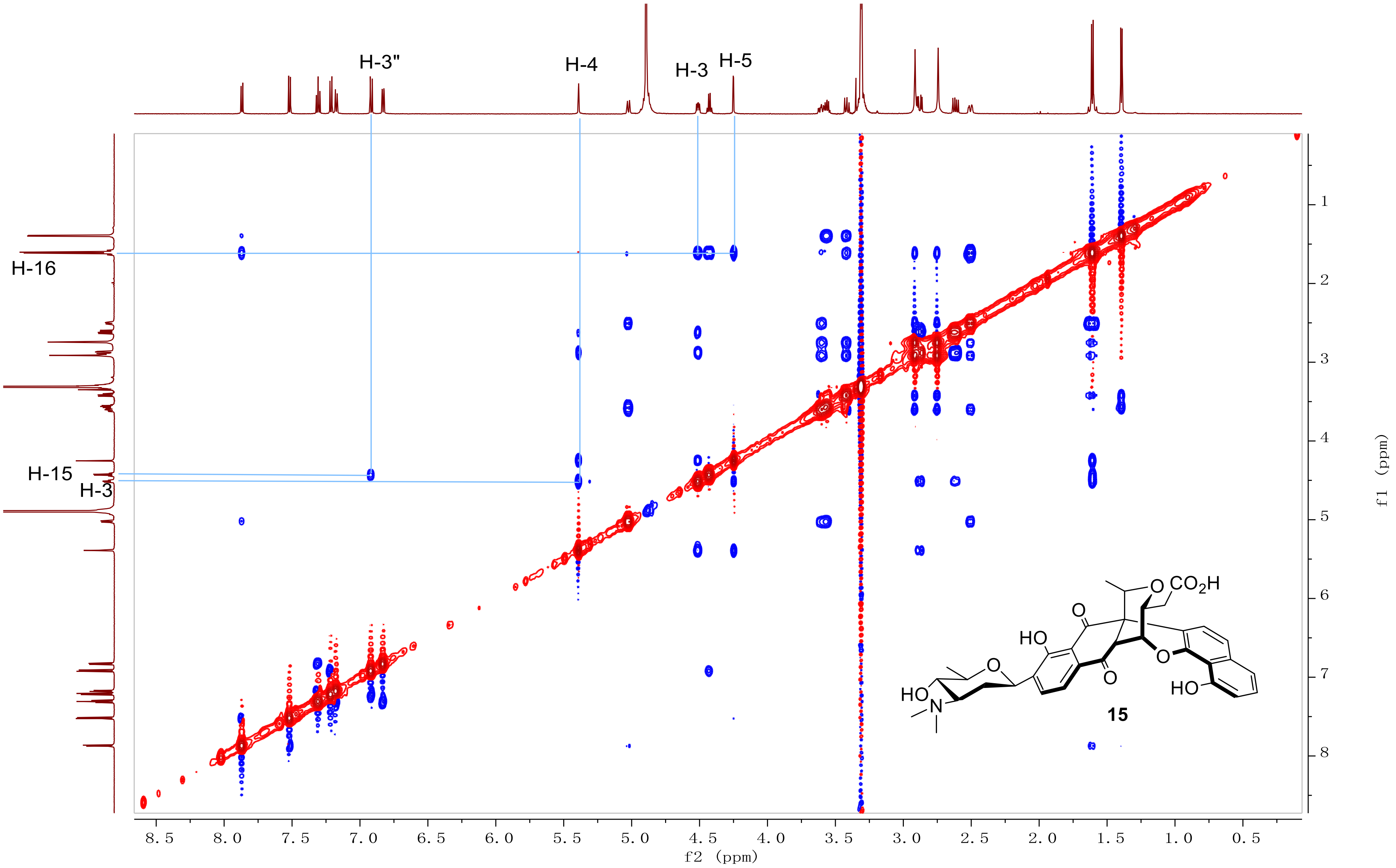

Supplementary Fig. 77. HRESIMS spectrum of chimedermycin J (**21**)

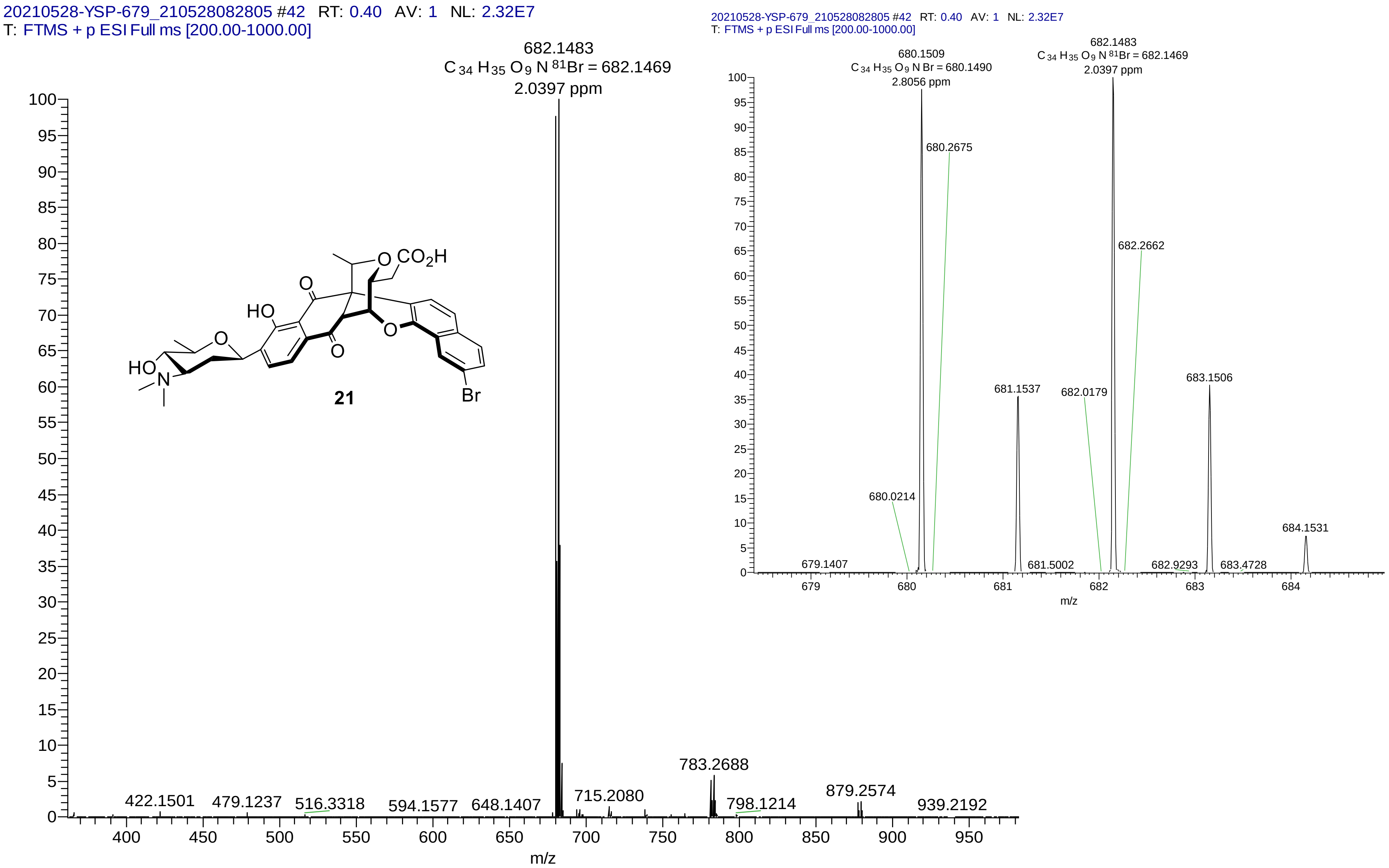

Supplementary Fig. 78. ^{1}H-NMR spectrum of chimedermycin J (**21**) in methanol-d_4

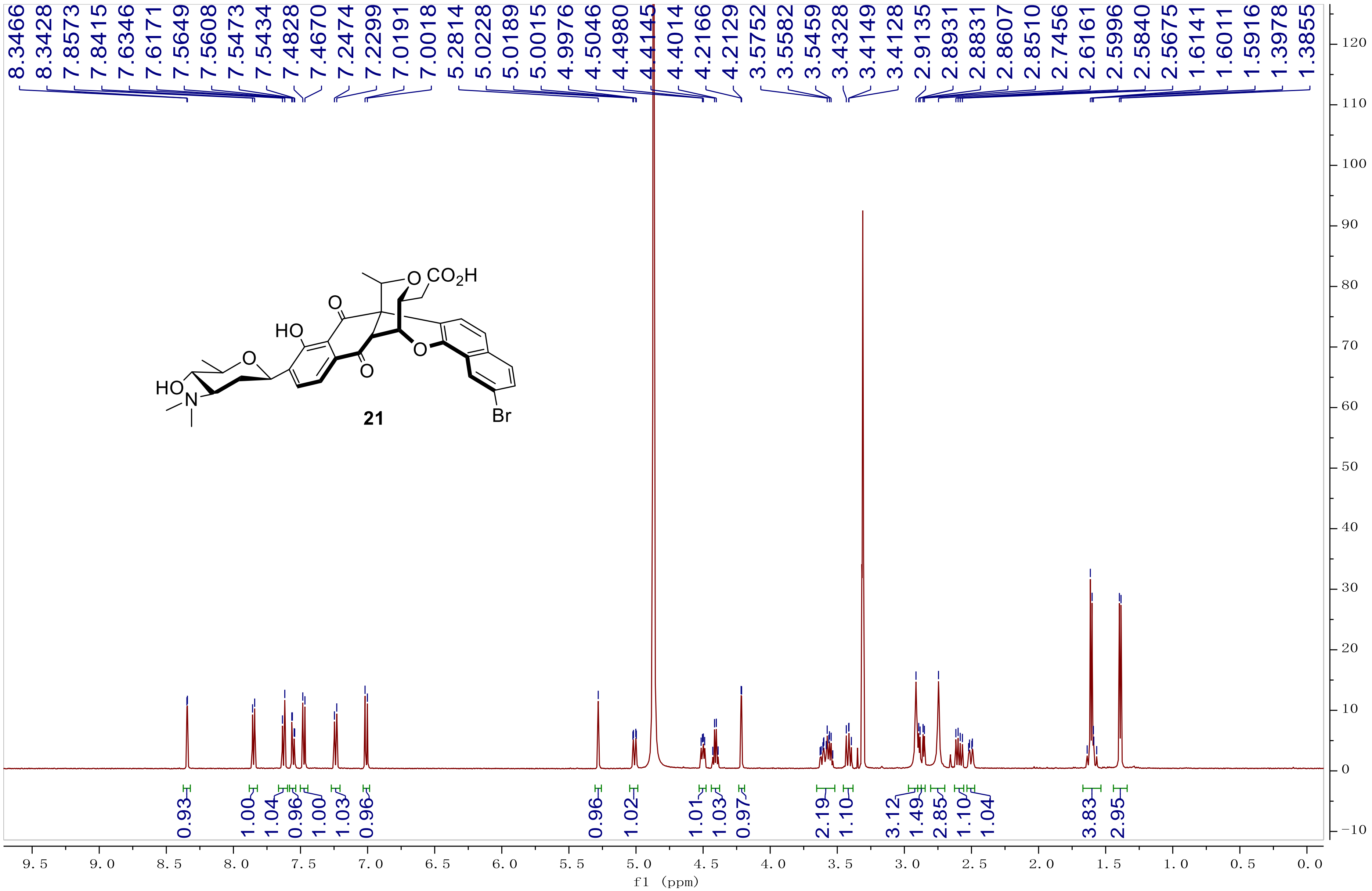

Supplementary Fig. 79. ^{13}C-NMR spectrum of chimedermycin J (**21**) in methanol-d_4

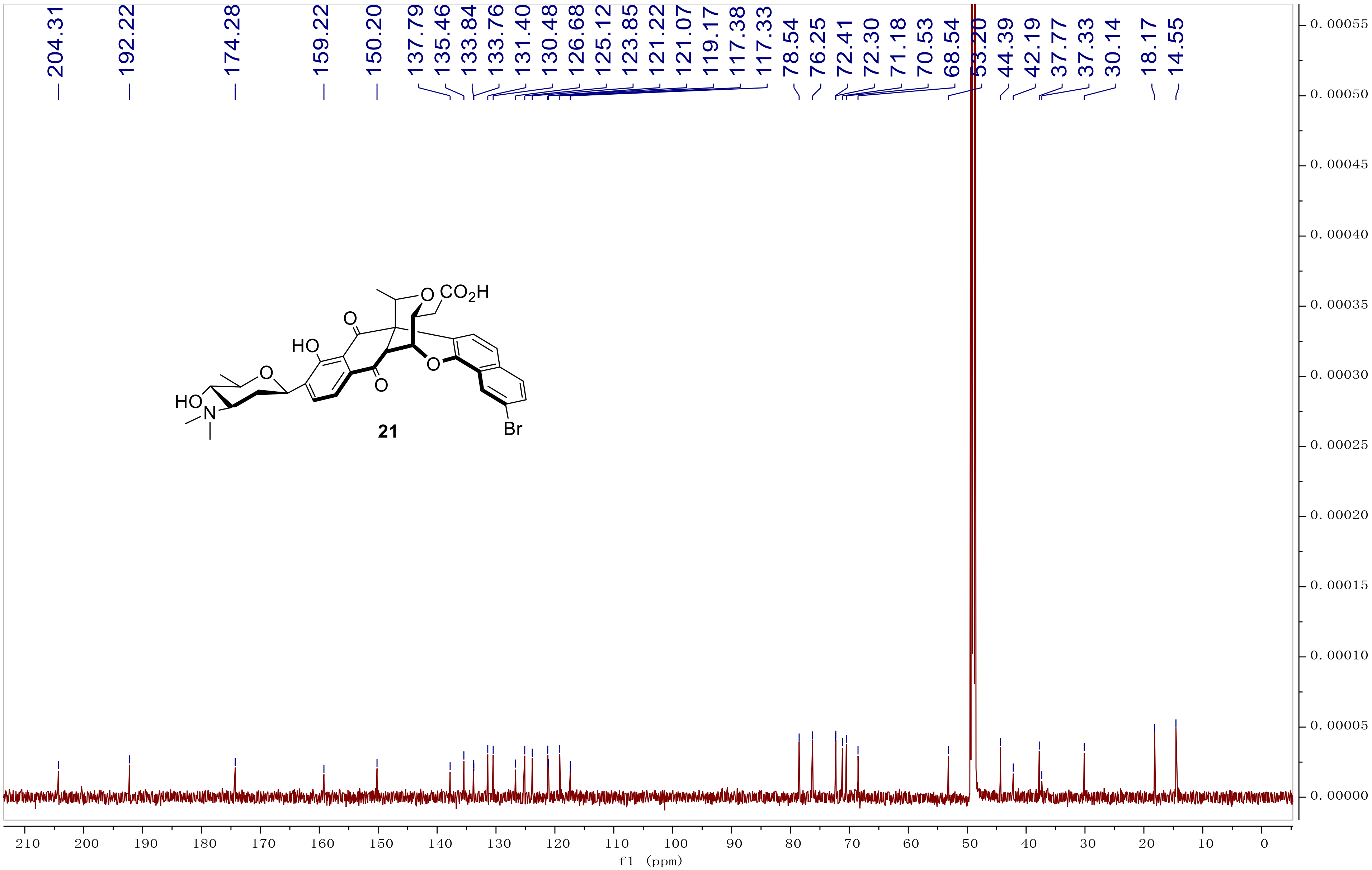

Supplementary Fig. 80. HSQC spectrum of chimedermycin J (**21**) in methanol-d_4

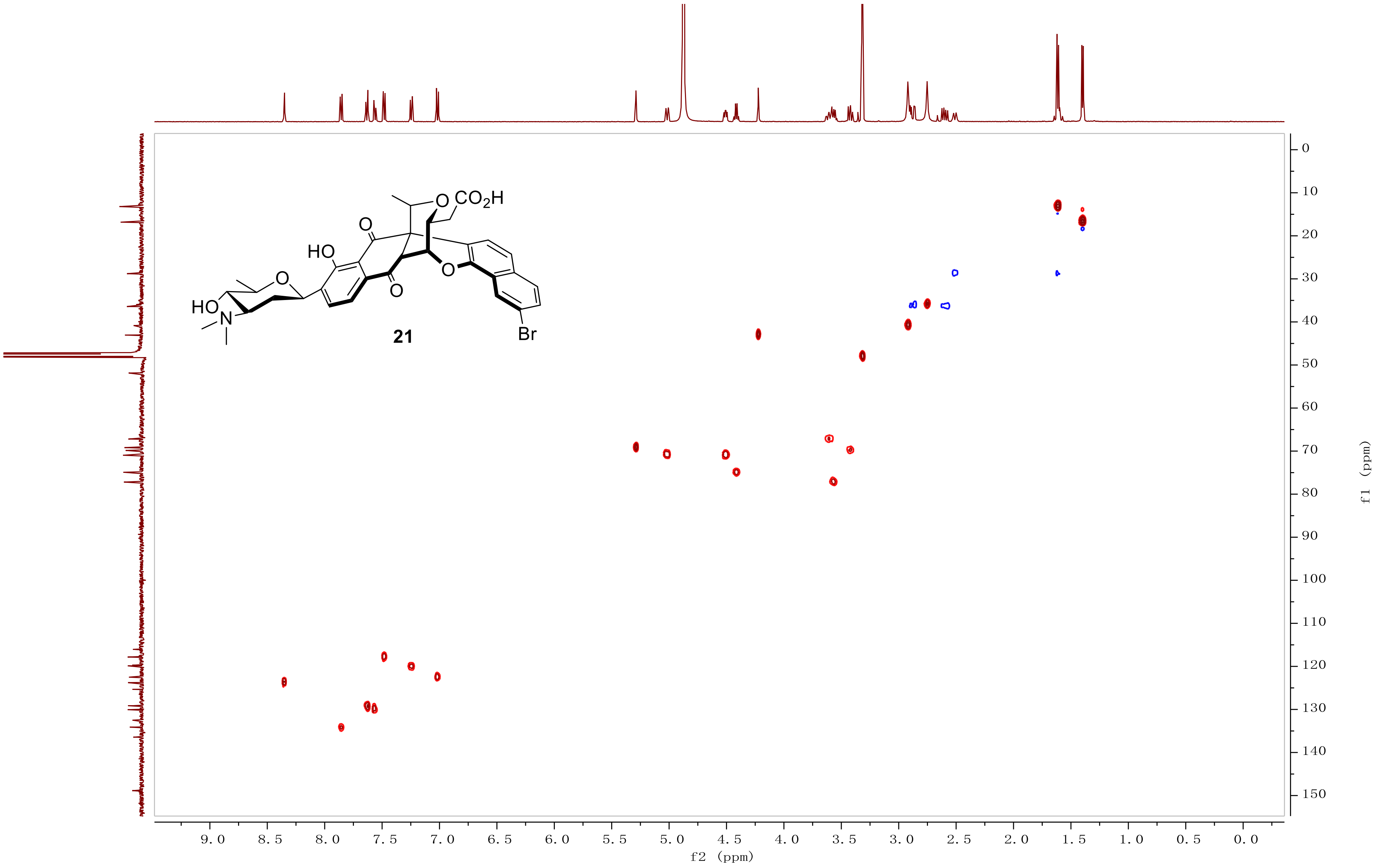

Supplementary Fig. 81. ^{1}H-^{1}H COSY spectrum of chimedermycin J (**21**) in methanol-d_4

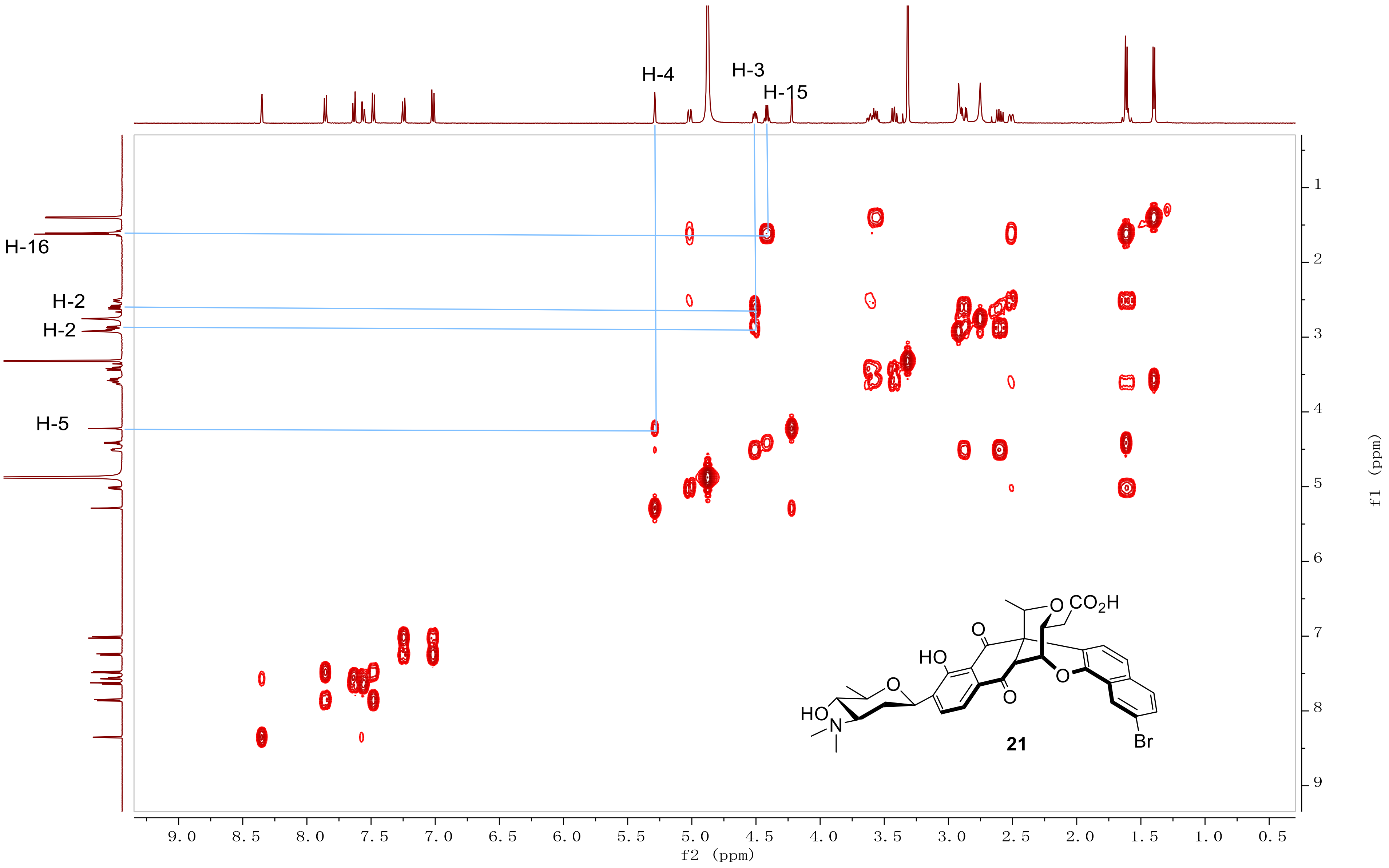

Supplementary Fig. 82. HMBC spectrum of chimedermycin J (**21**) in methanol-d_4

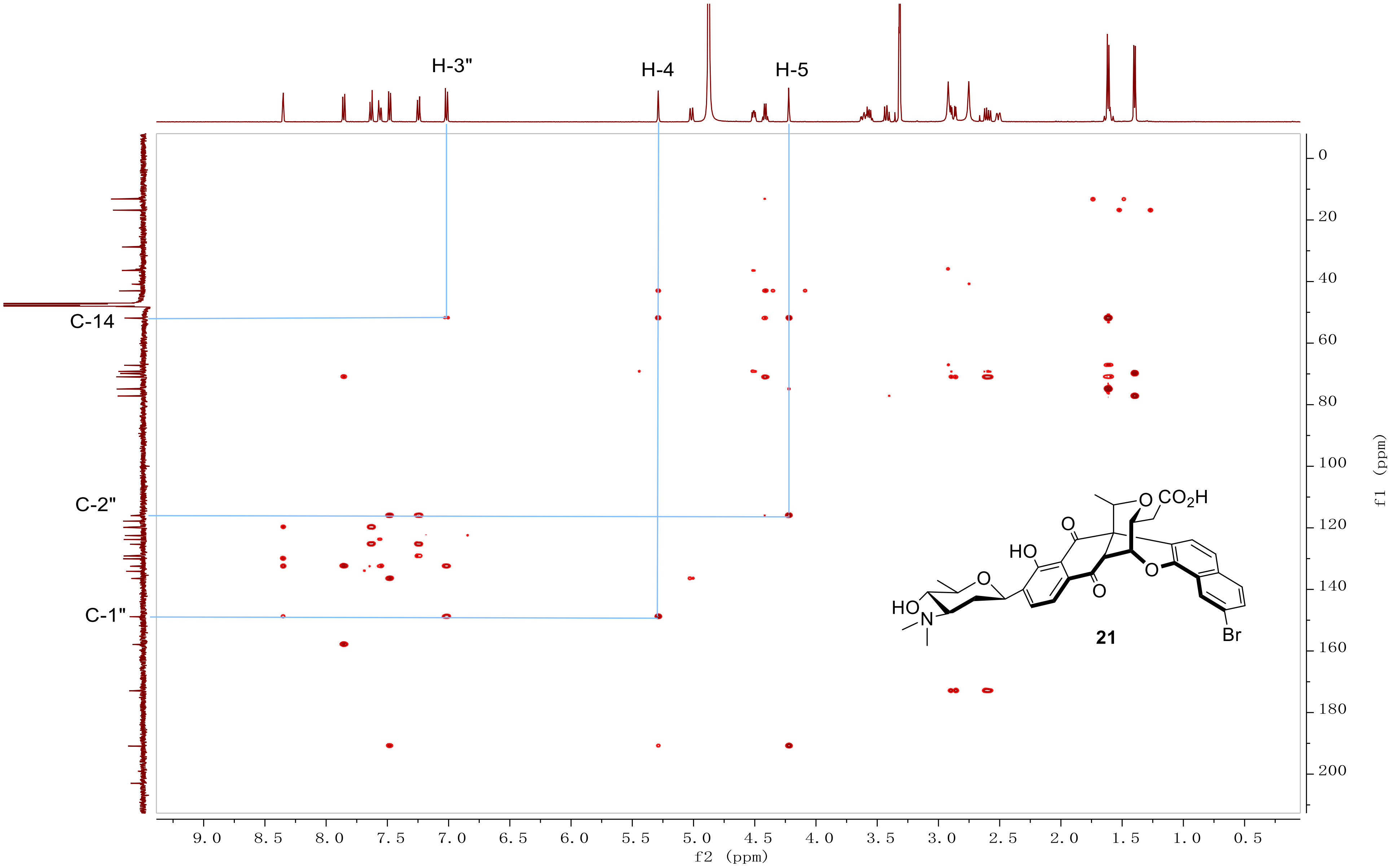

Supplementary Fig. 83. NOESY spectrum of chimedermycin J (**21**) in methanol-d_4

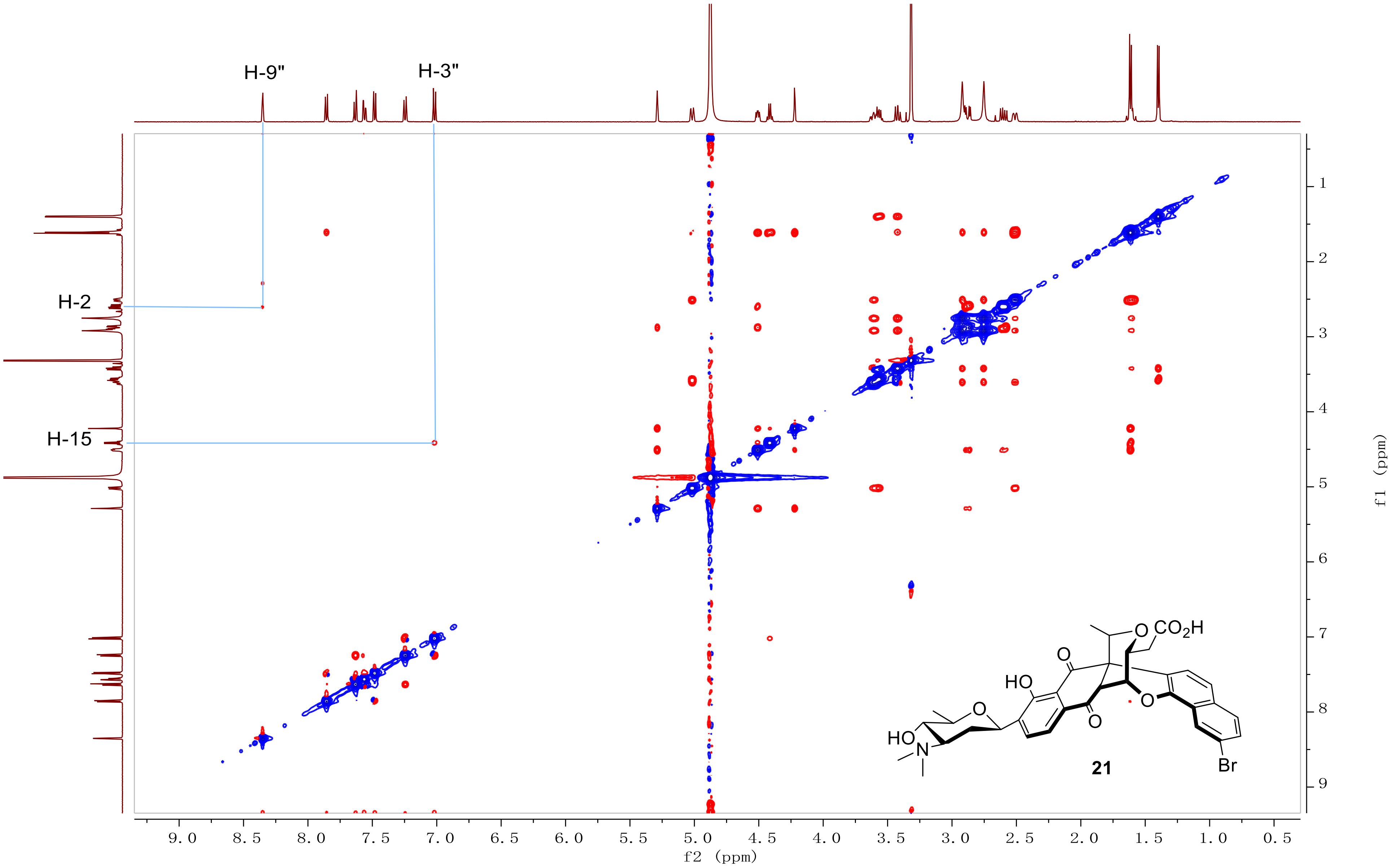

Supplementary Fig. 84. HRESIMS spectrum of chimedermycin K (**22**)

20201011-YSP-583_201011102014 #54 RT: 0.44 AV: 1 NL: 2.77E8
T: FTMS + p ESI Full ms [150.00-2000.00]

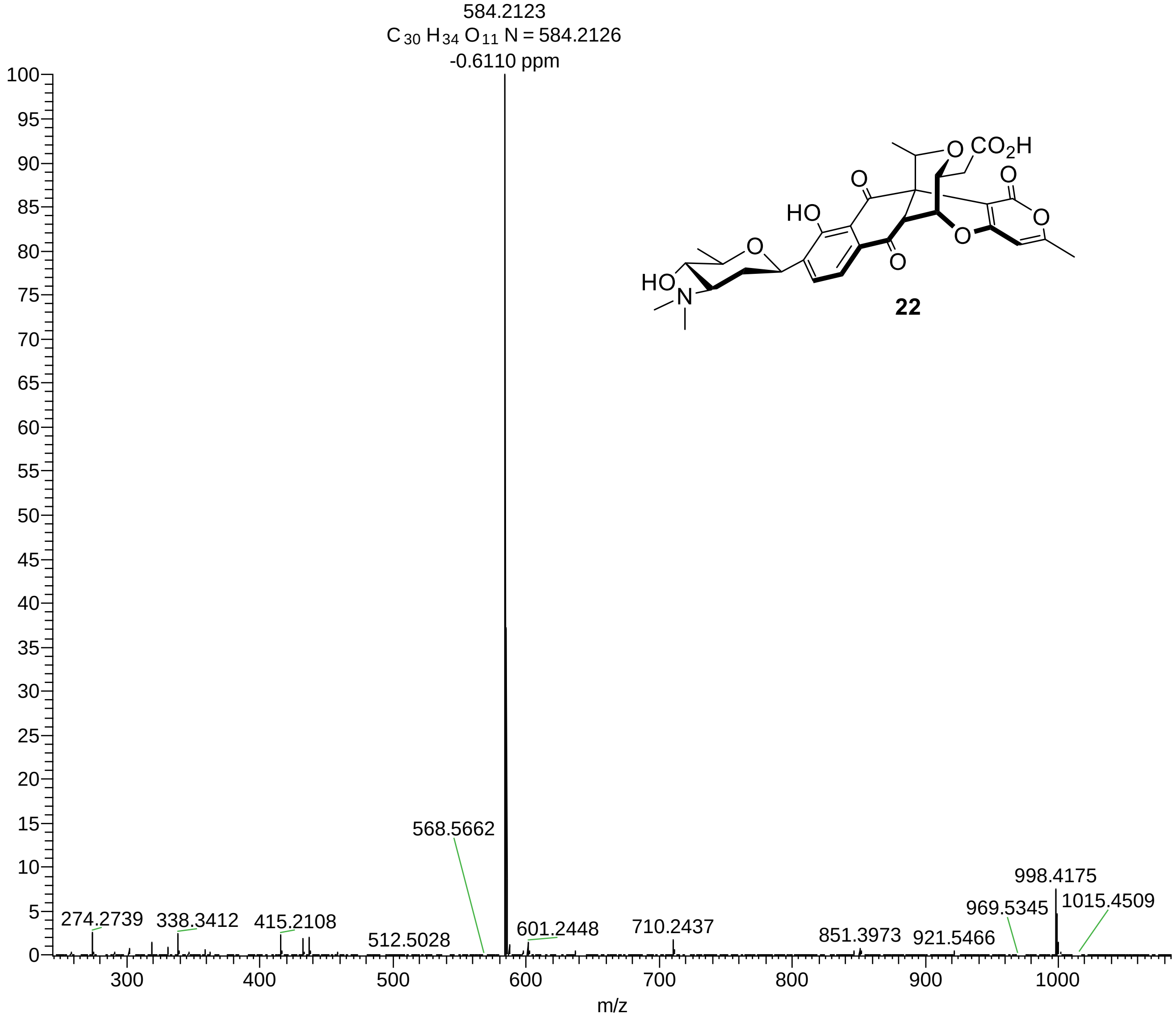

22

Supplementary Fig. 85. ^{1}H-NMR spectrum of chimedermycin K (**22**) in methanol-d_4

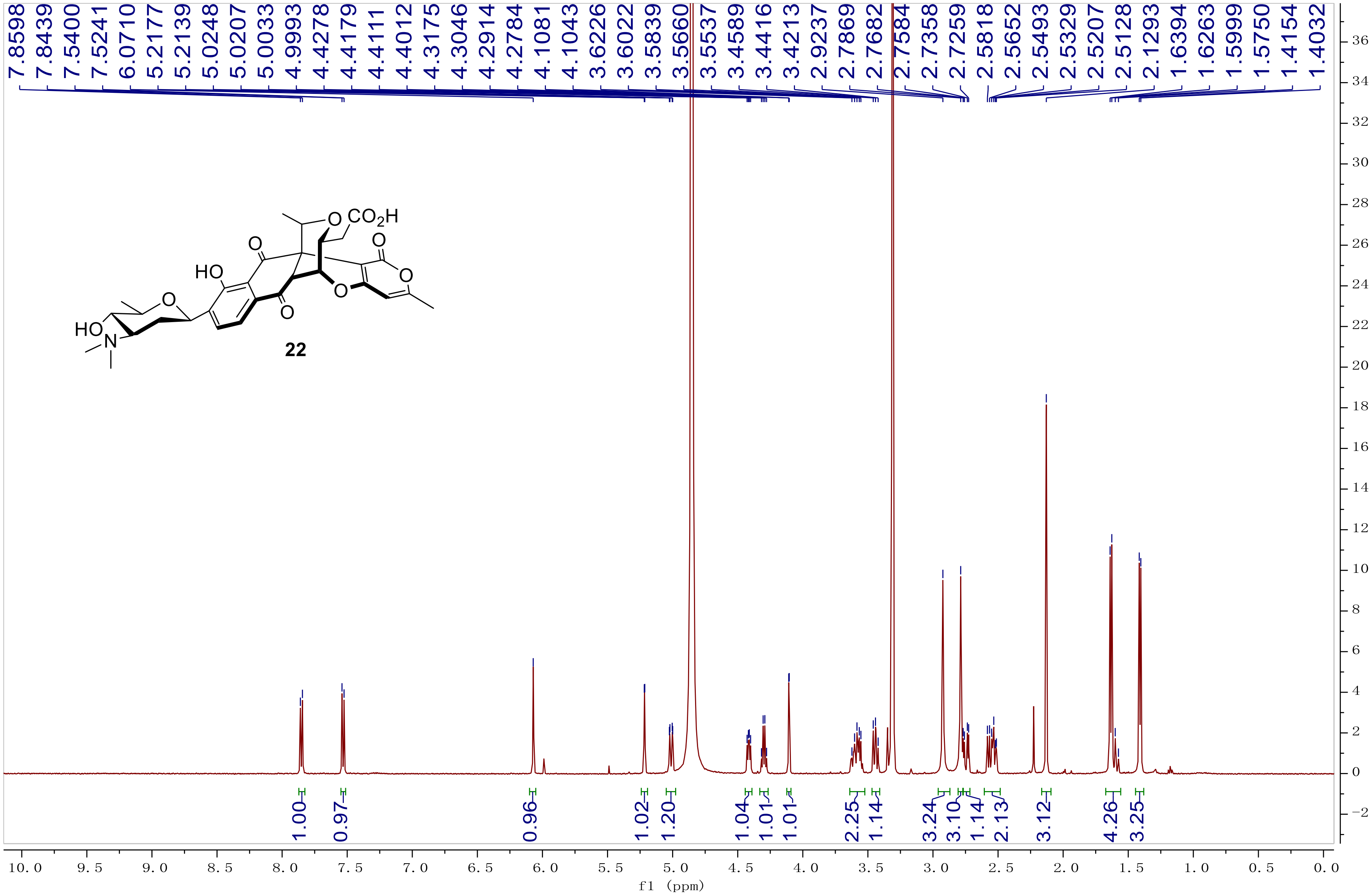

Supplementary Fig. 86. ^{13}C-NMR spectrum of chimedermycin K (**22**) in methanol-d_4

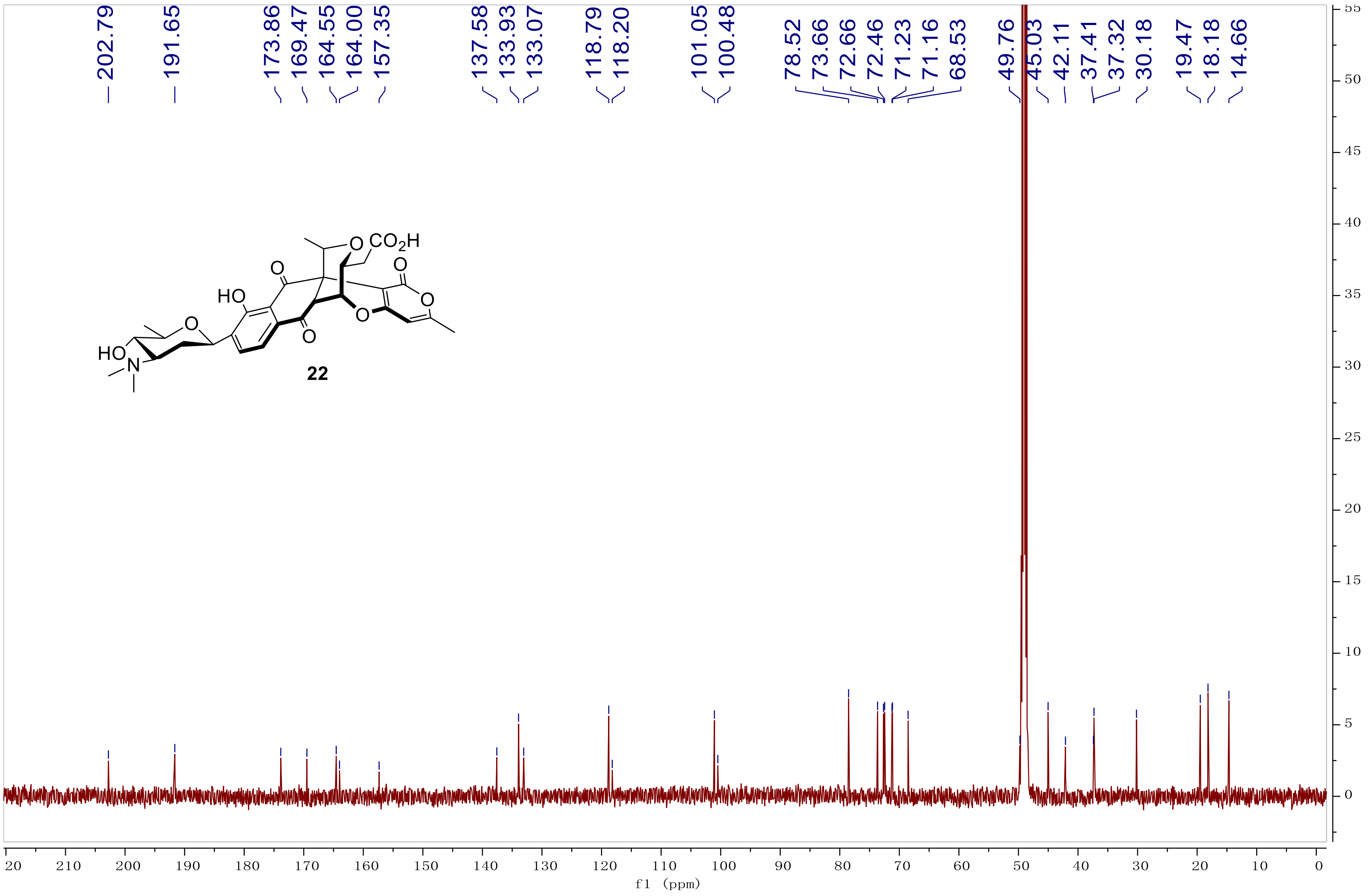

Supplementary Fig. 87. HSQC spectrum of chimedermycin K (**22**) in methanol-d_4

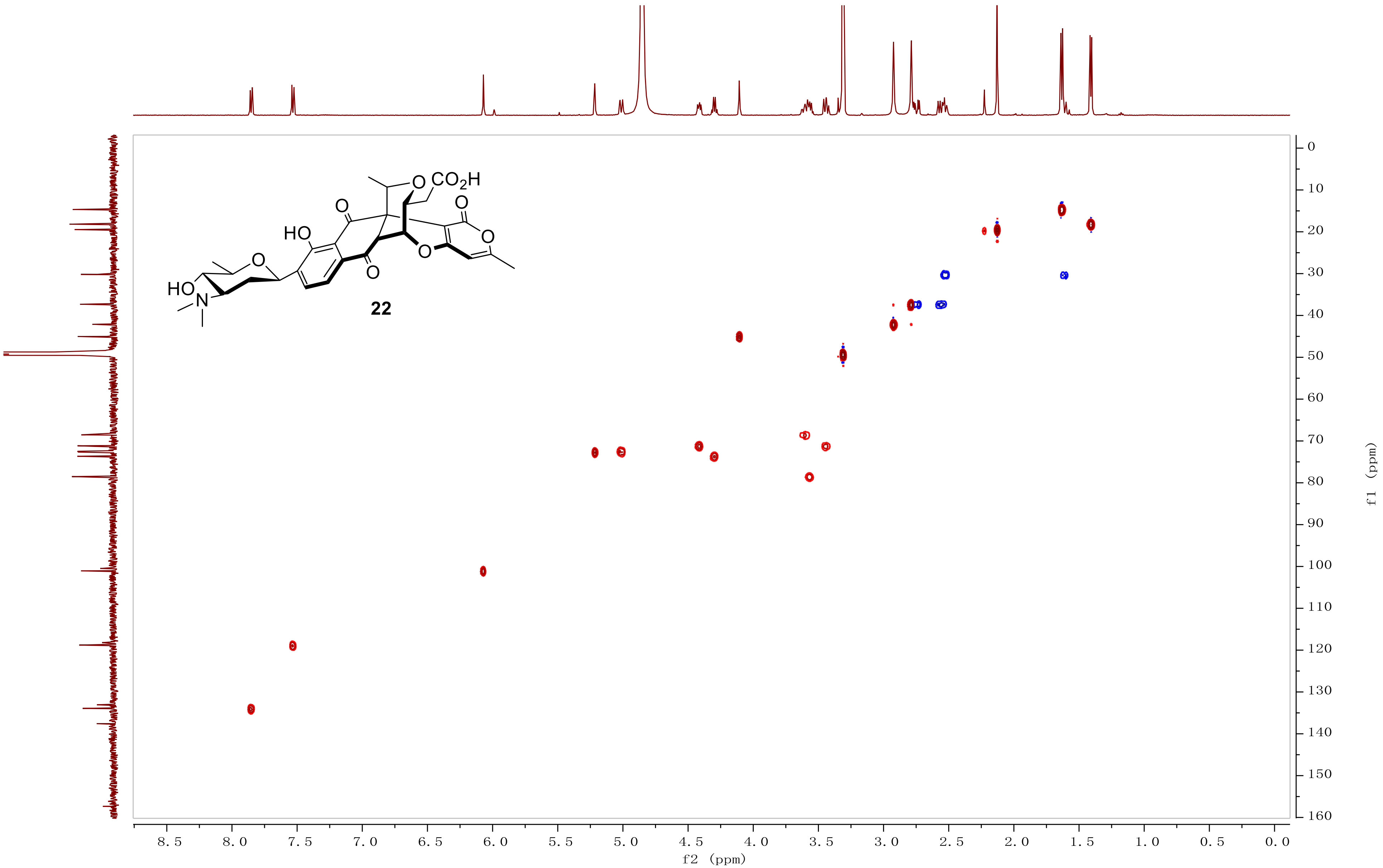

Supplementary Fig. 88. ^1H-^1H COSY spectrum of chimedermycin K (**22**) in methanol-d_4

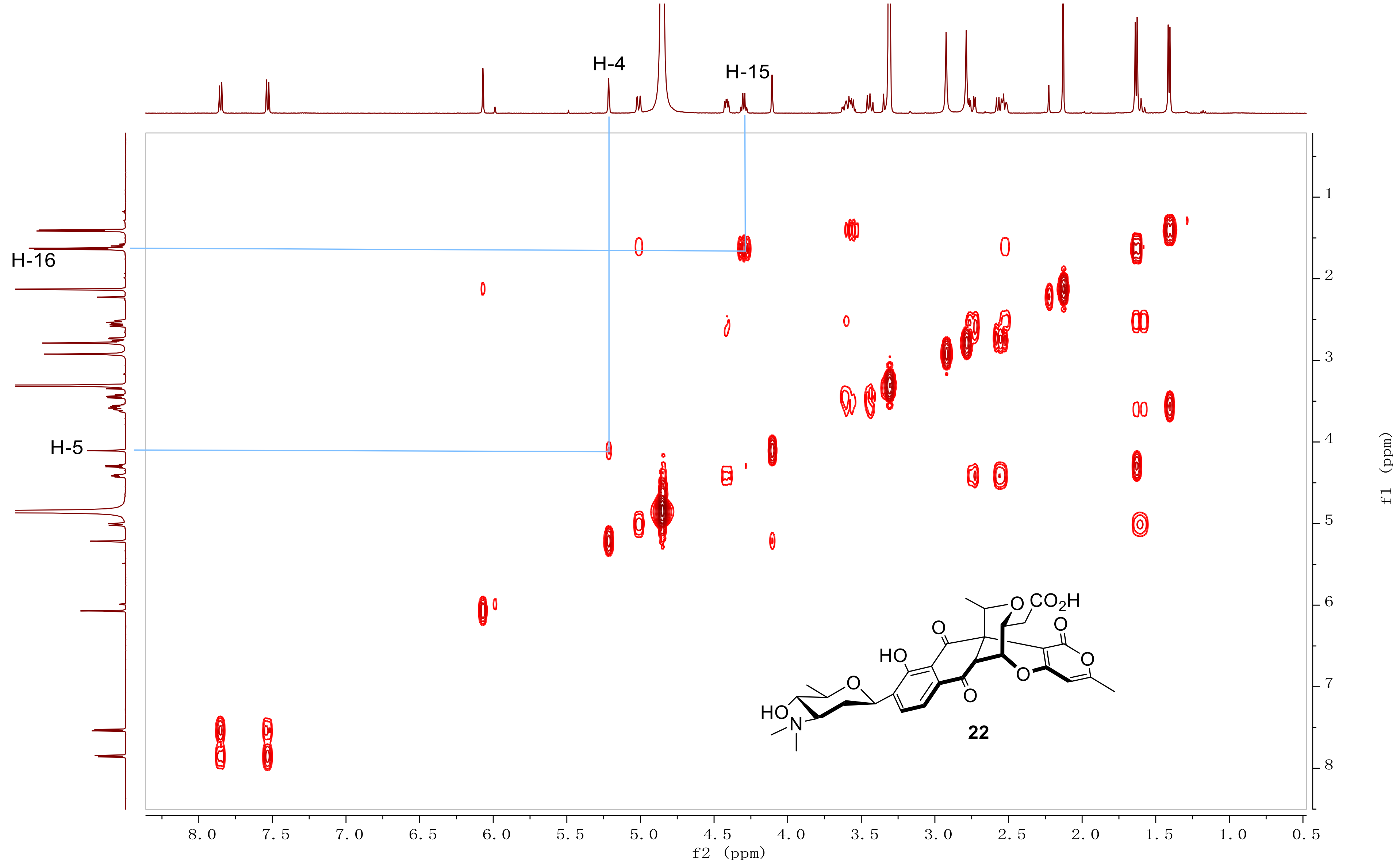

Supplementary Fig. 89. HMBC spectrum of chimedermycin K (**22**) in methanol-d_4

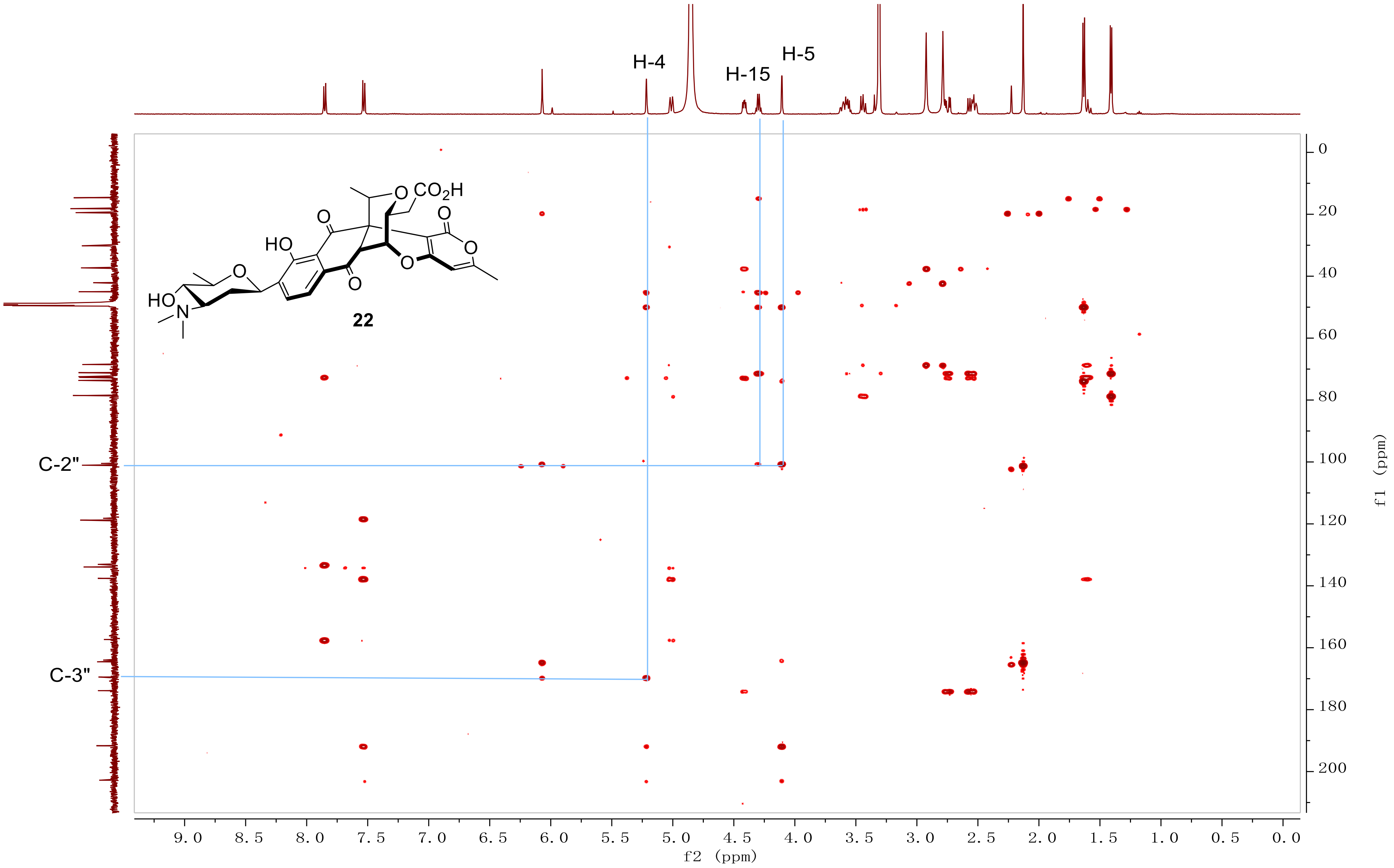

Supplementary Fig. 90. NOESY spectrum of chimedermycin K (**22**) in methanol-d_4

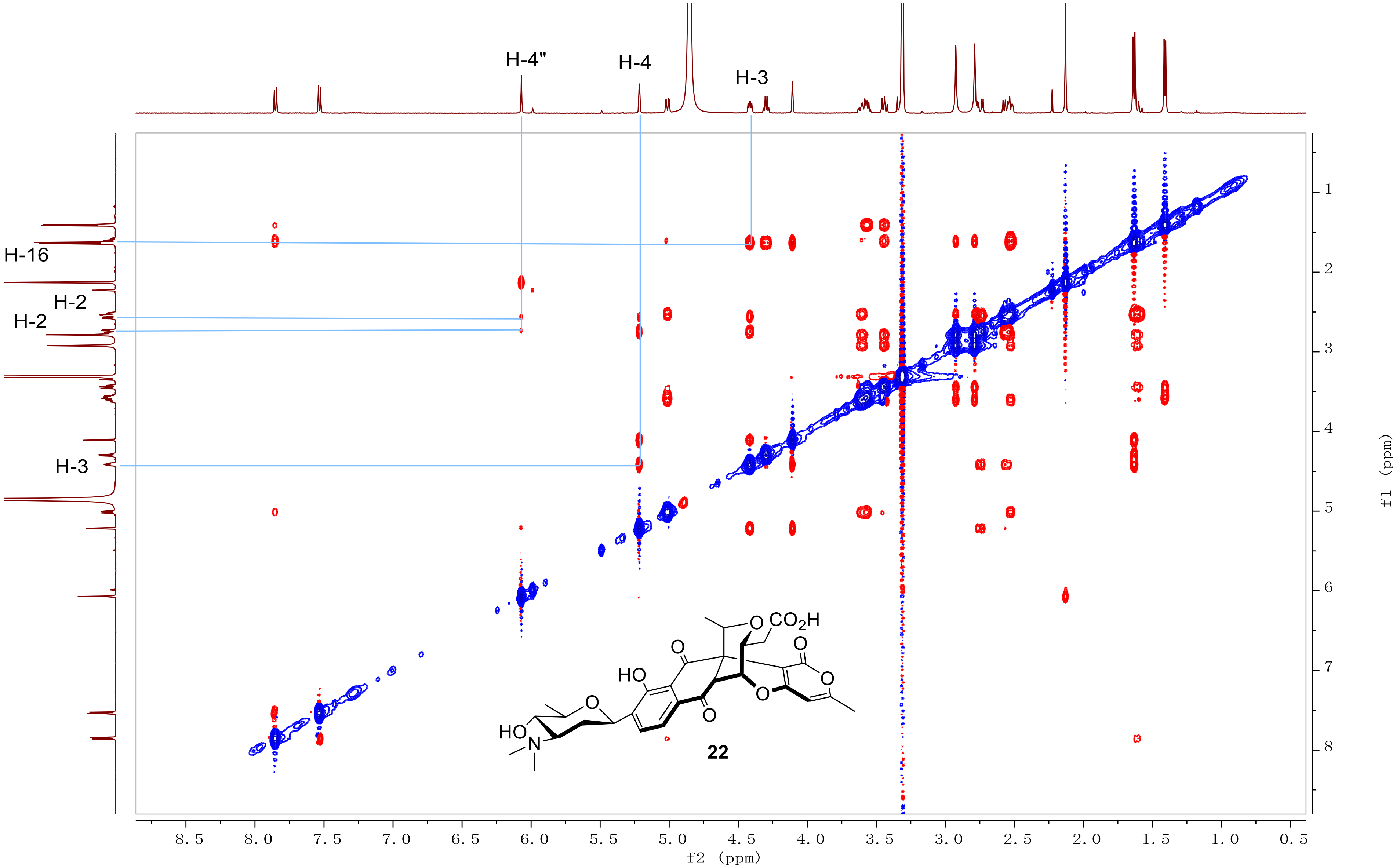

Supplementary Fig. 91. HRESIMS spectrum of chimedermycin L (**23**)

20201011-YSP-619_201011102014 #41 RT: 0.35 AV: 1 SB: 3 0.01-0.02 NL: 6.20E6
T: FTMS + p ESI Full ms [150.00-2000.00]

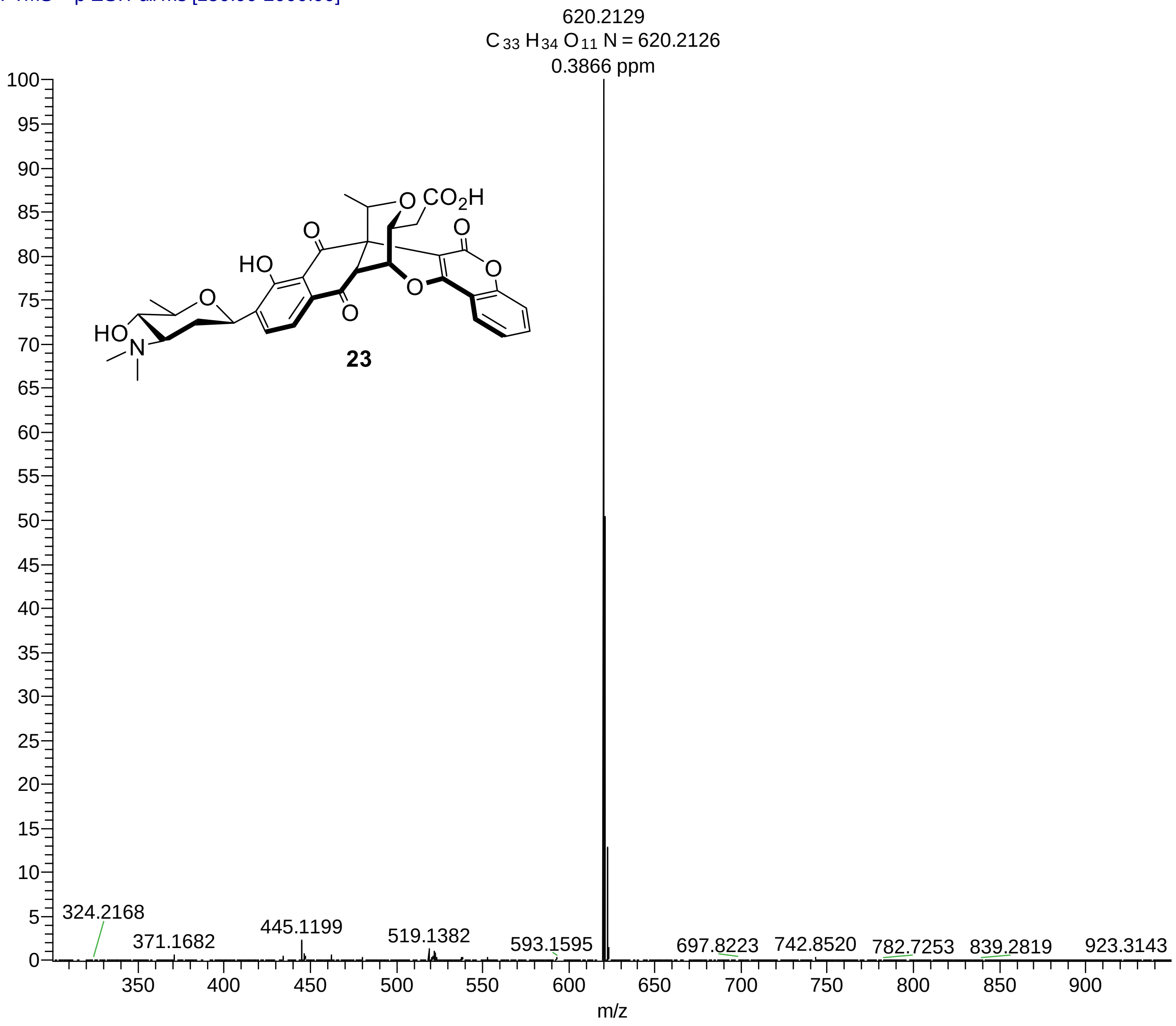

Supplementary Fig. 92. ^{1}H-NMR spectrum of chimedermycin L (**23**) in methanol-d_4

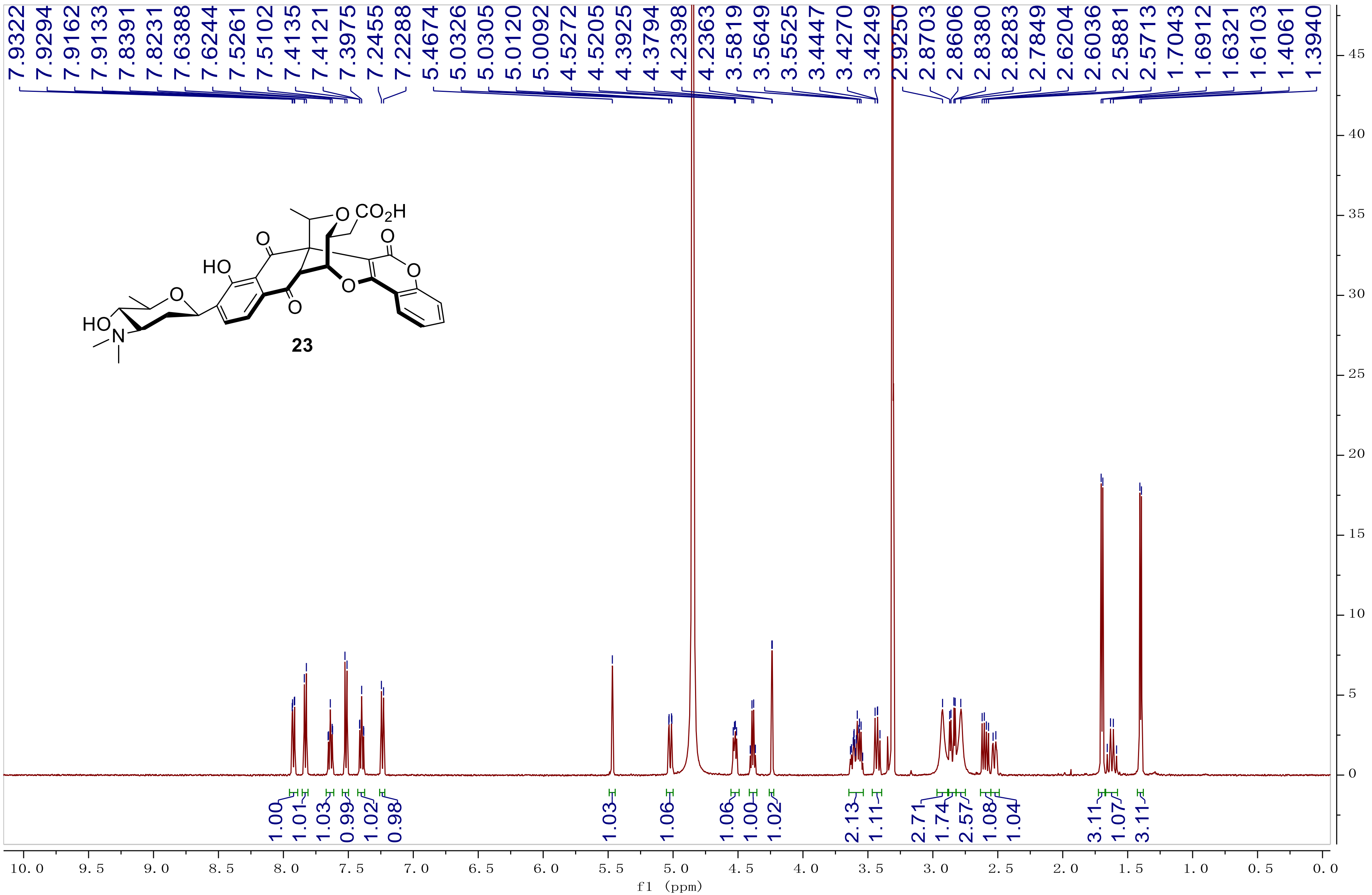

Supplementary Fig. 93. ^{13}C-NMR spectrum of chimedermycin L (**23**) in methanol-d_4

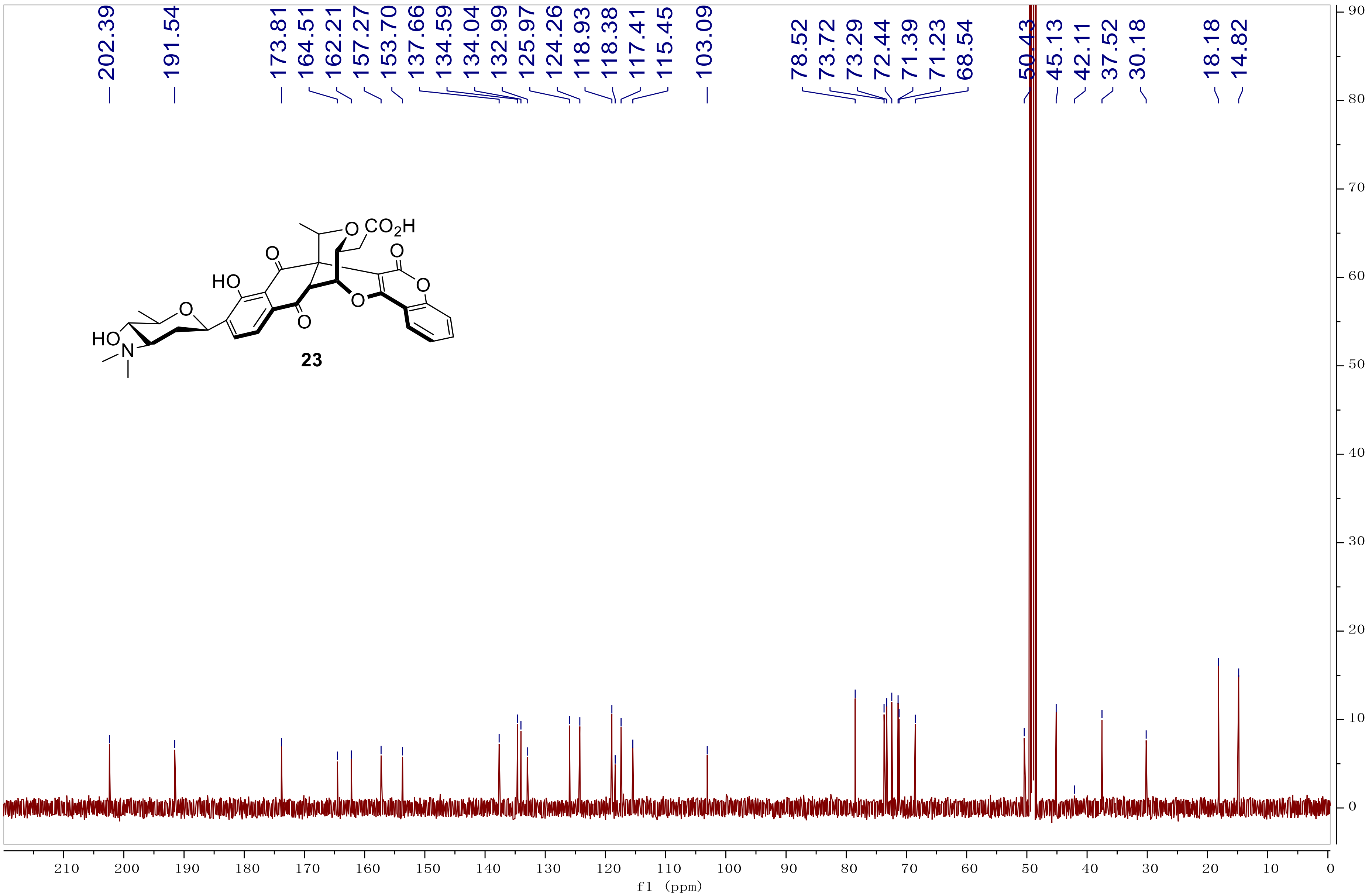

Supplementary Fig. 94. HSQC spectrum of chimedermycin L (**23**) in methanol-d_4

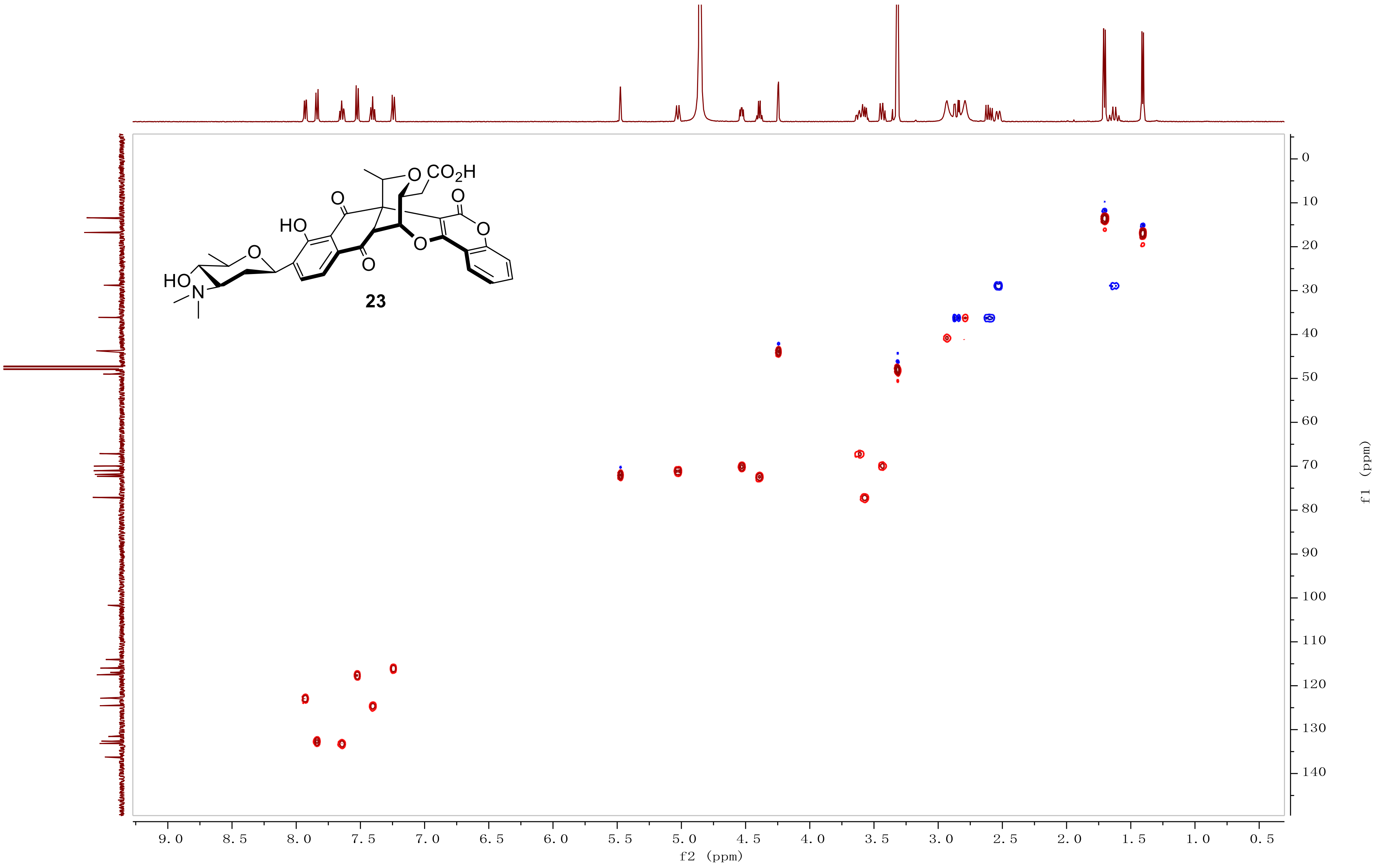

Supplementary Fig. 95. ^{1}H-^{1}H COSY spectrum of chimedermycin L (**23**) in methanol-d_4

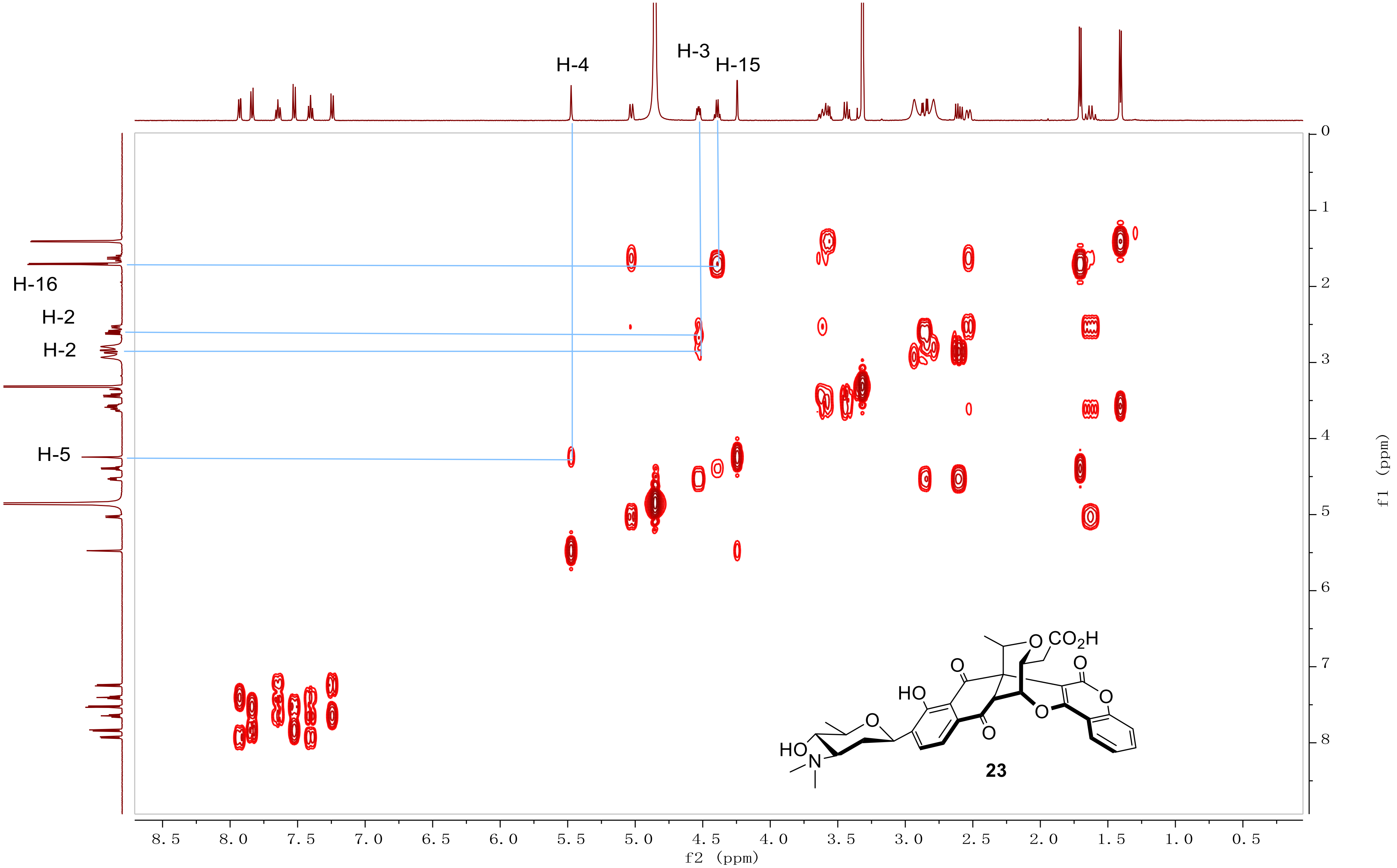

Supplementary Fig. 96. HMBC spectrum of chimedermycin L (**23**) in methanol-d_4

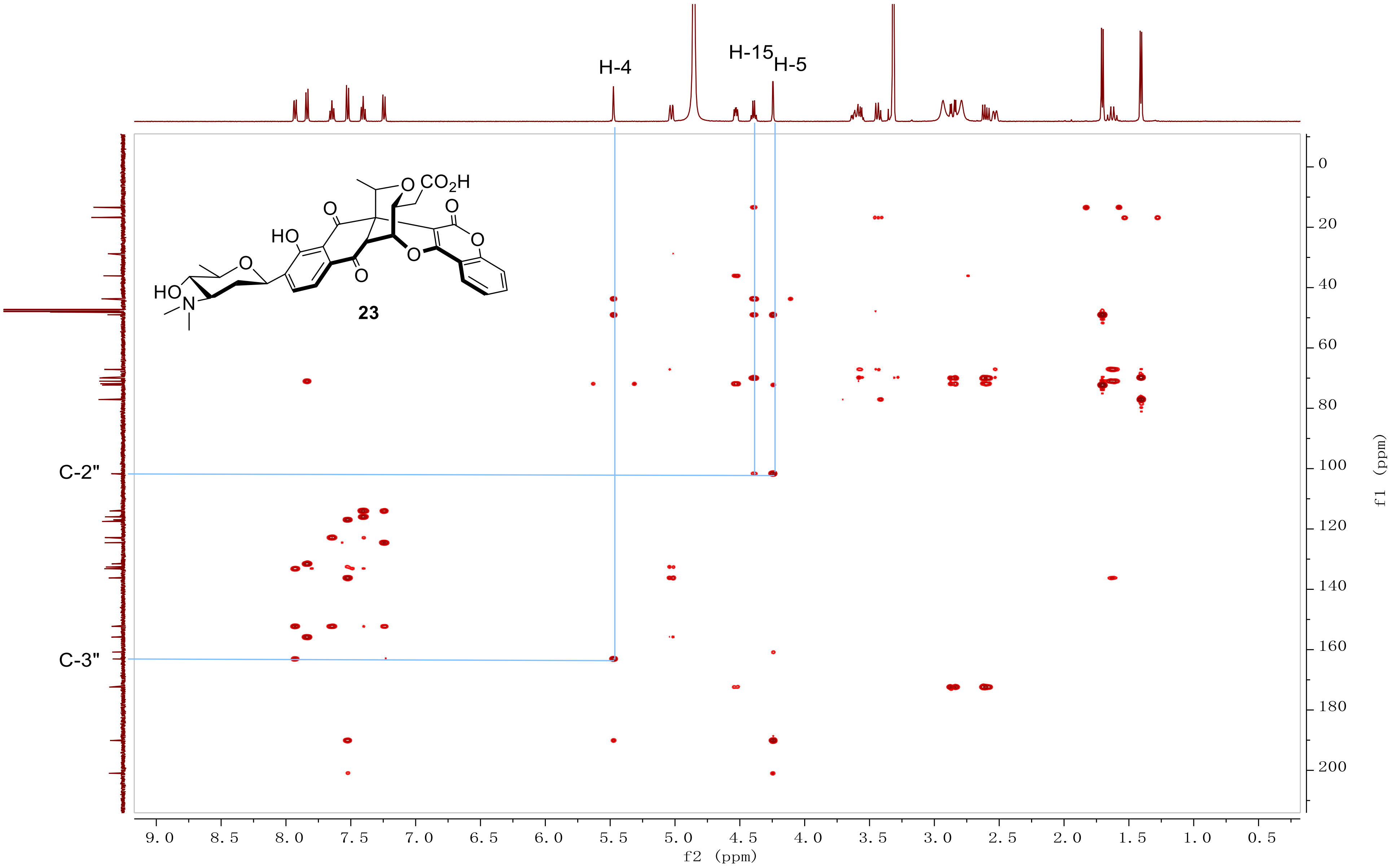

Supplementary Fig. 97. NOESY spectrum of chimedermycin L (**23**) in methanol-d_4

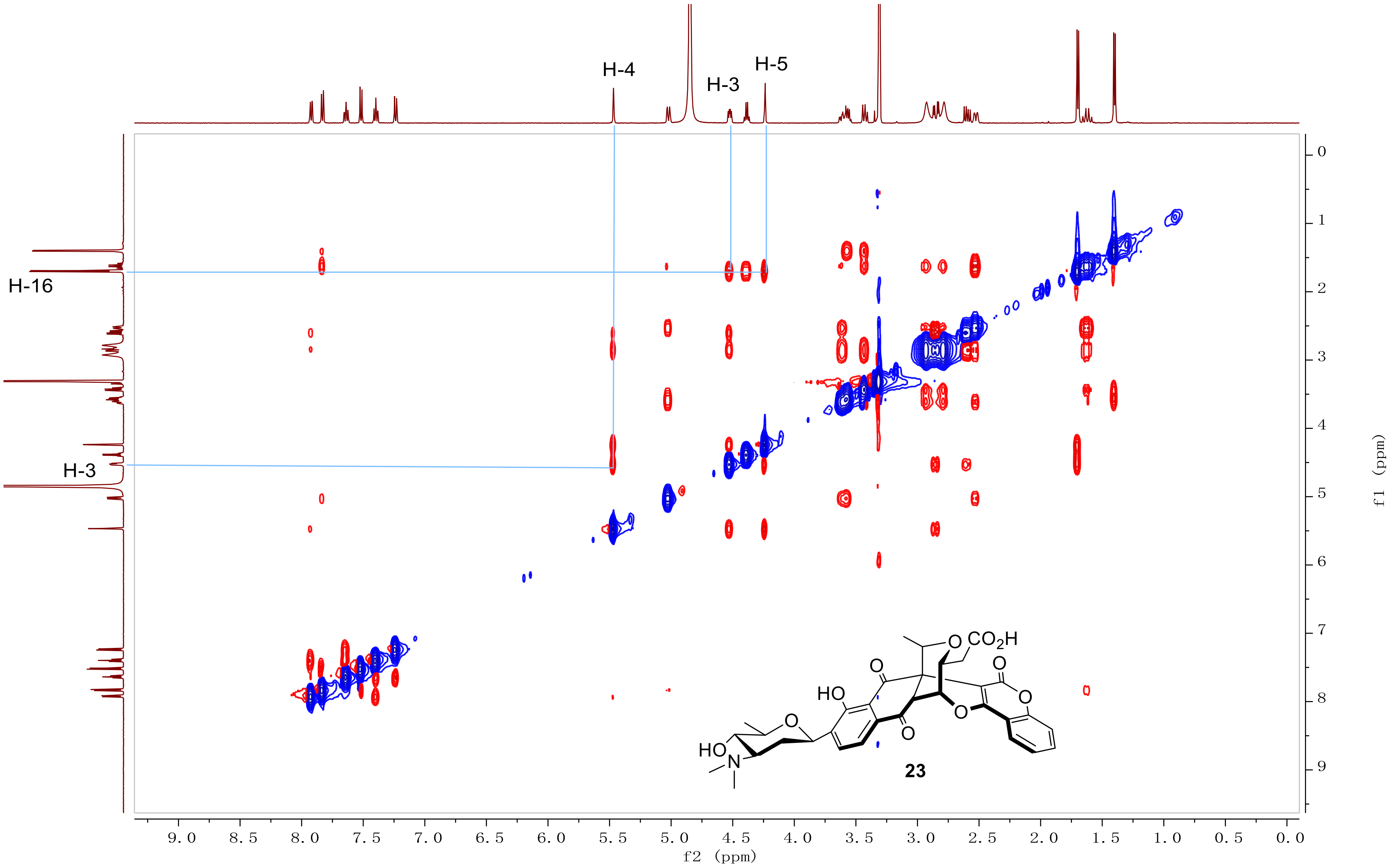

Supplementary Fig. 98. HRESIMS spectrum of chimedermycin M (**24**)

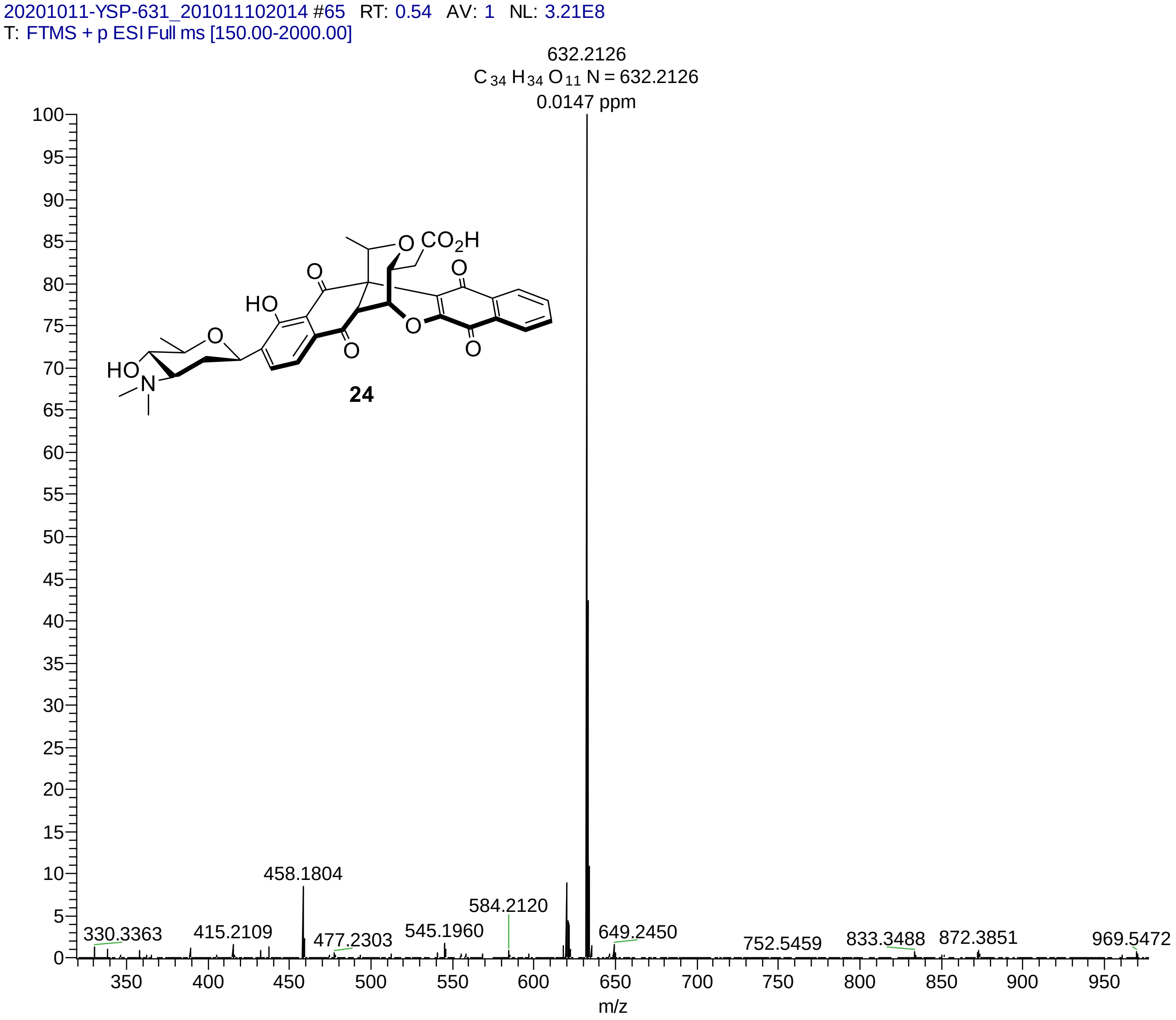

Supplementary Fig. 99. ^{1}H-NMR spectrum of chimedermycin M (**24**) in methanol-d_4

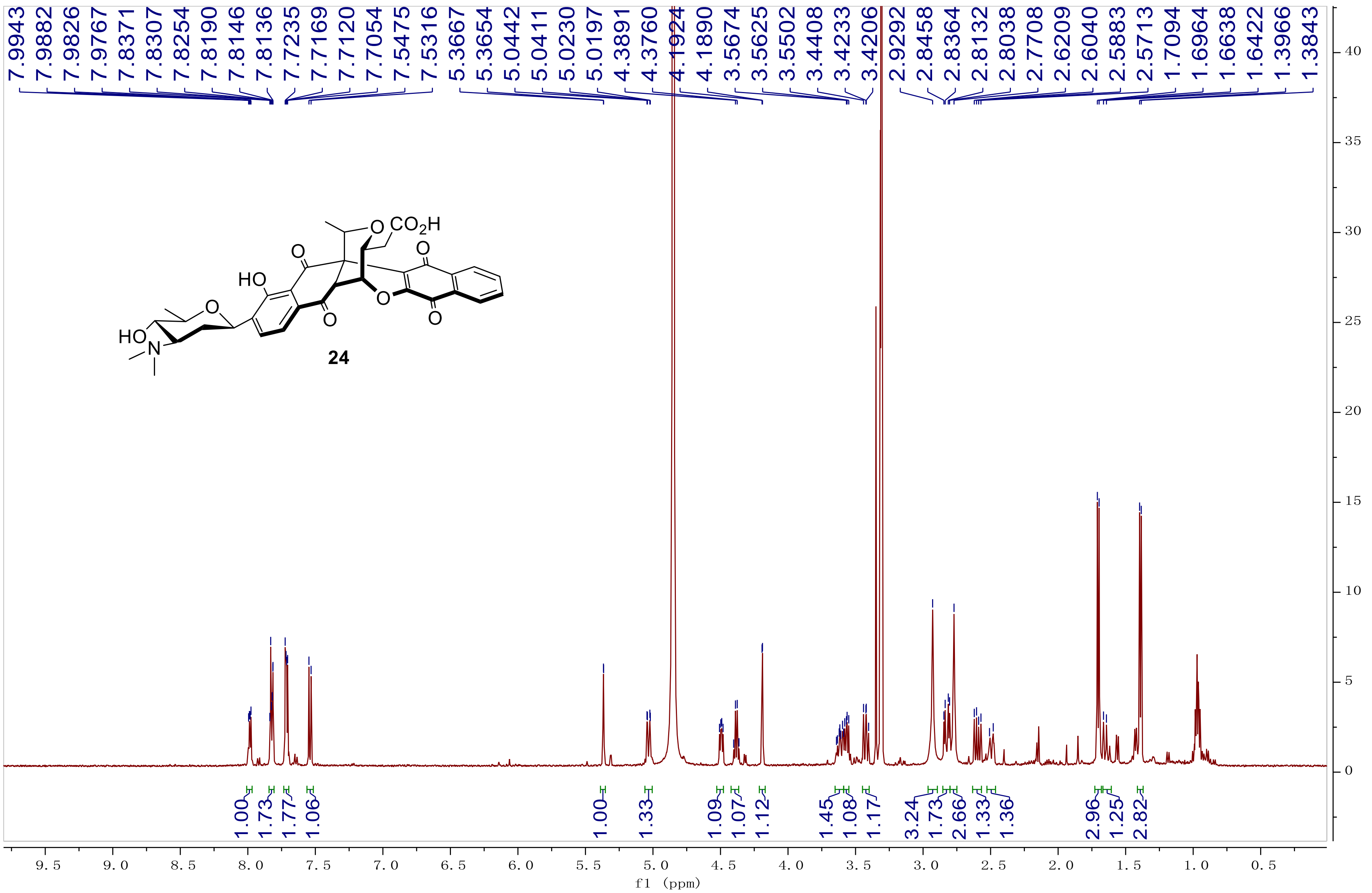

Supplementary Fig. 100. ^{13}C-NMR spectrum of chimedermycin M (**24**) in methanol-d_4

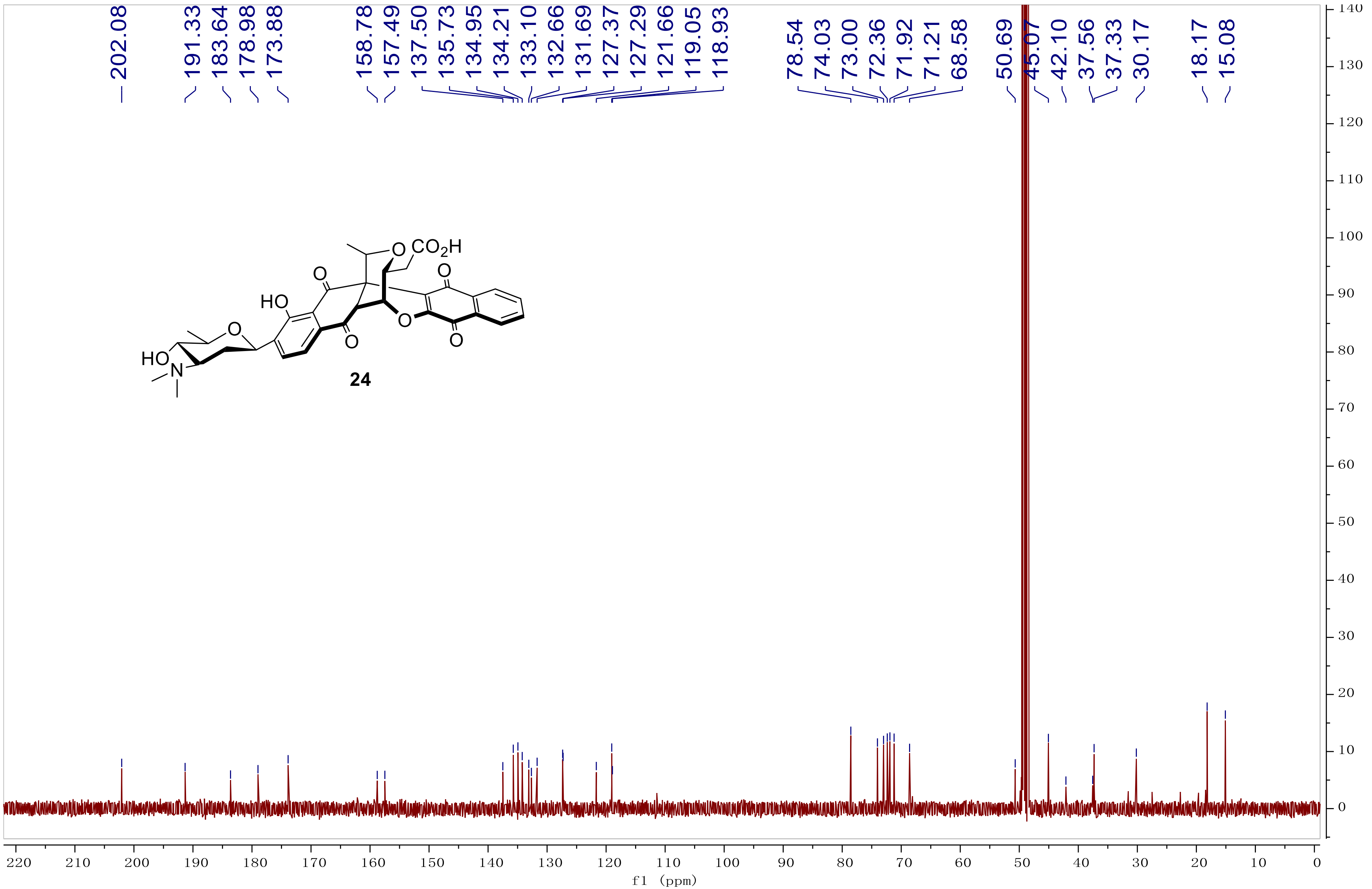

Supplementary Fig. 101. HSQC spectrum of chimedermycin M (**24**) in methanol-d_4

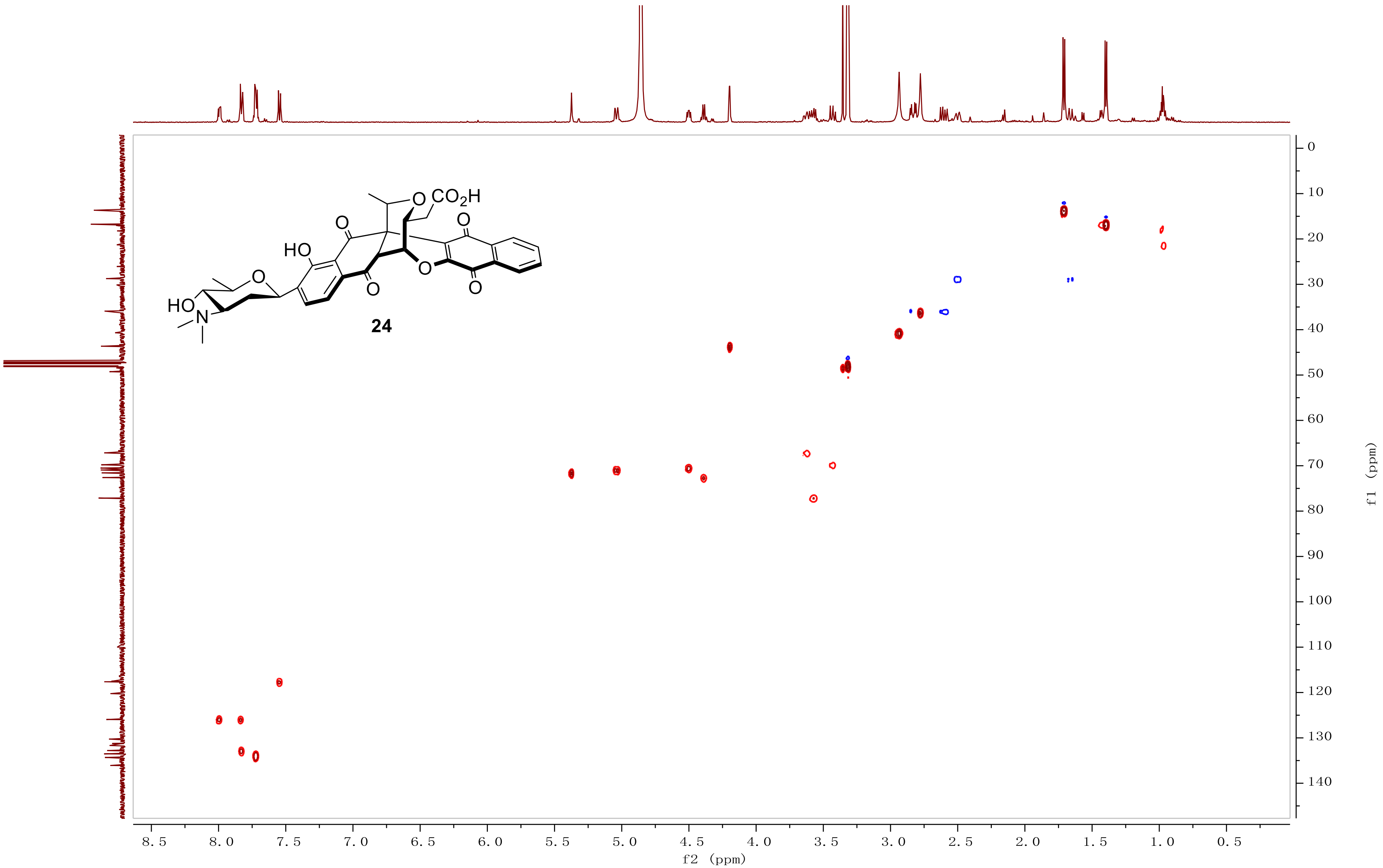

Supplementary Fig. 102. ^1H-^1H COSY spectrum of chimedermycin M (**24**) in methanol-*d*₄

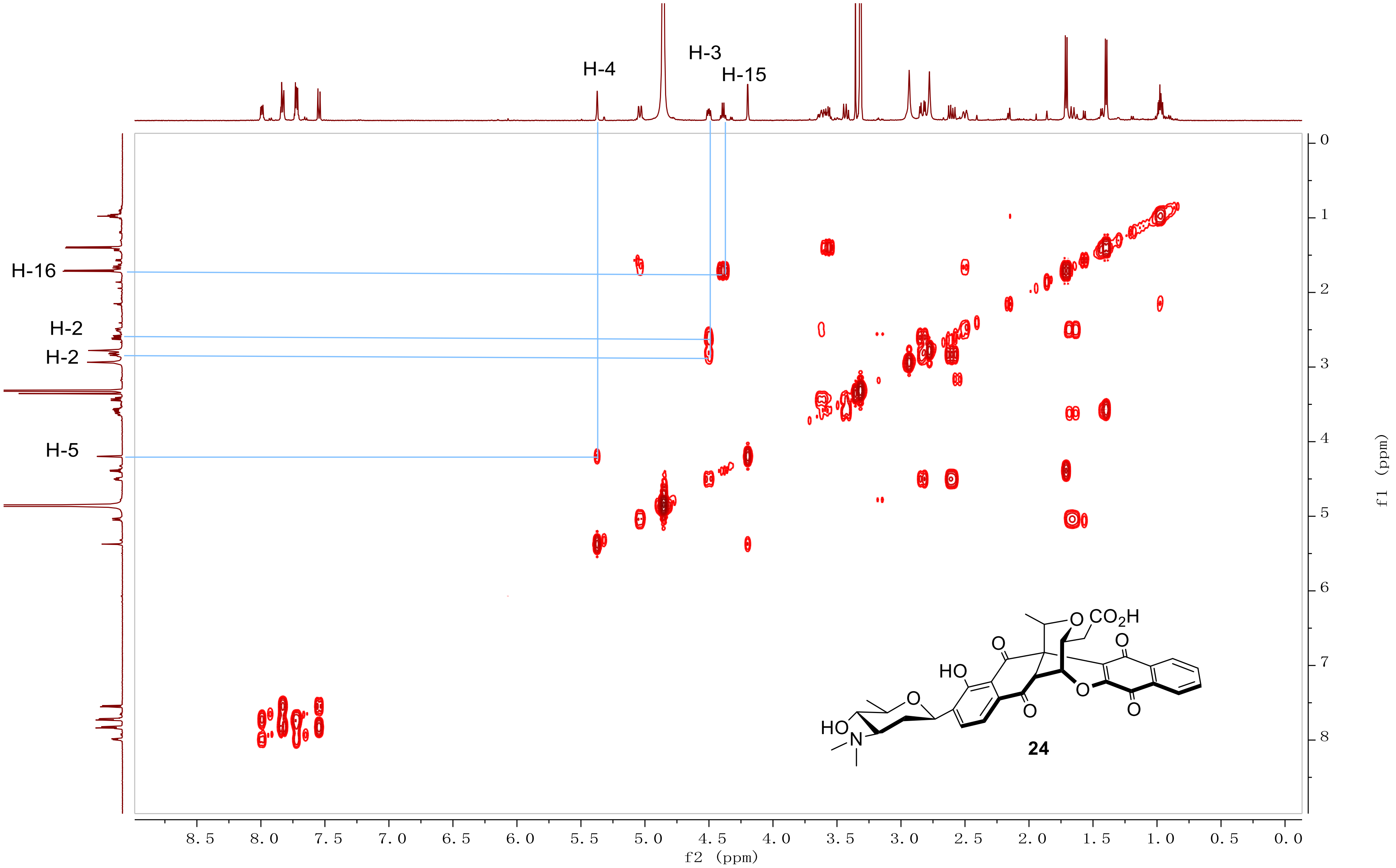

Supplementary Fig. 103. HMBC spectrum of chimedermycin M (**24**) in methanol-d_4

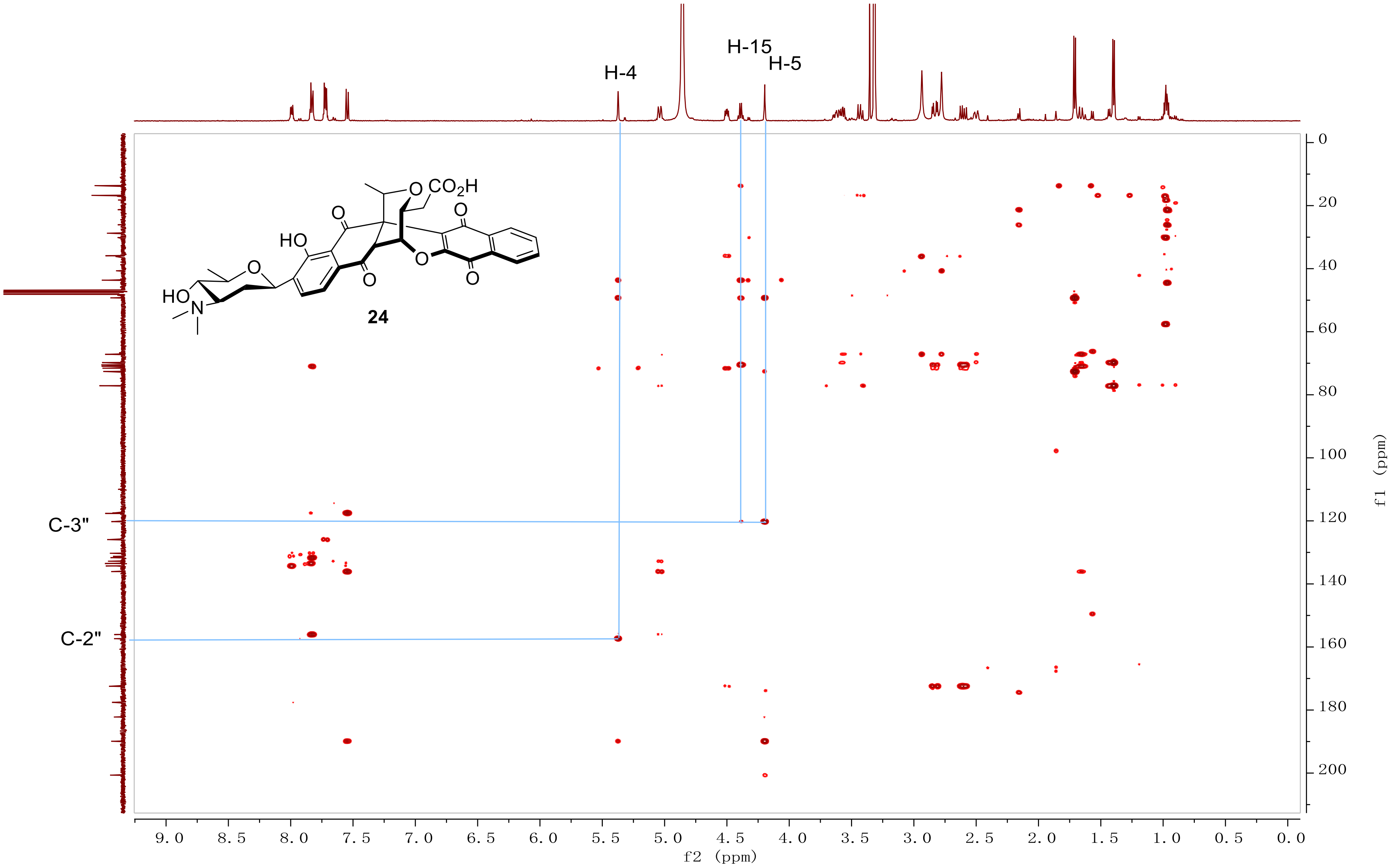

Supplementary Fig. 104. NOESY spectrum of chimedermycin M (**24**) in methanol-d_4

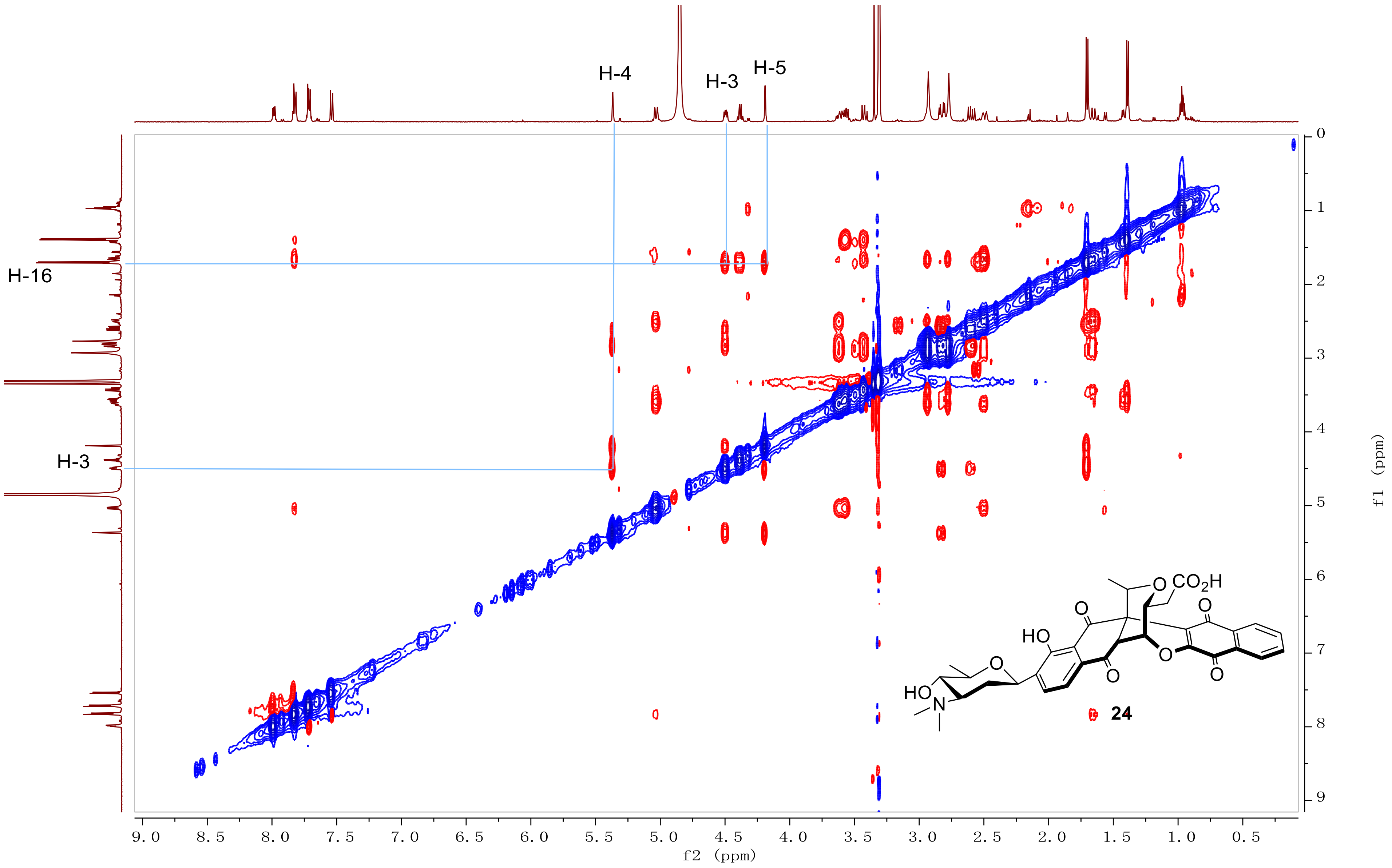

Supplementary Fig. 105. HRESIMS spectrum of chimedermycin N (**25**)

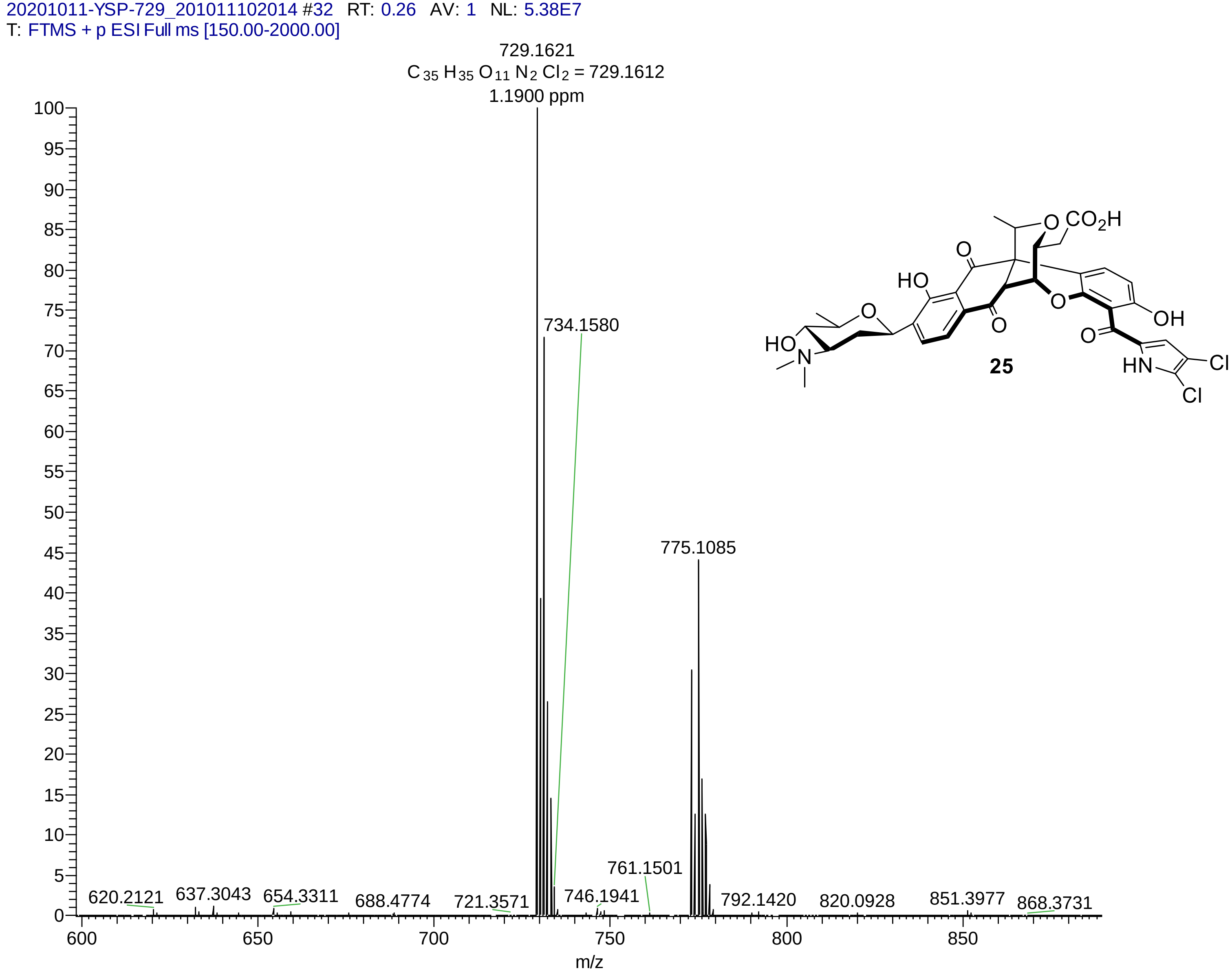

Supplementary Fig. 106. ^{1}H-NMR spectrum of chimedermycin N (**25**) in methanol-d_4

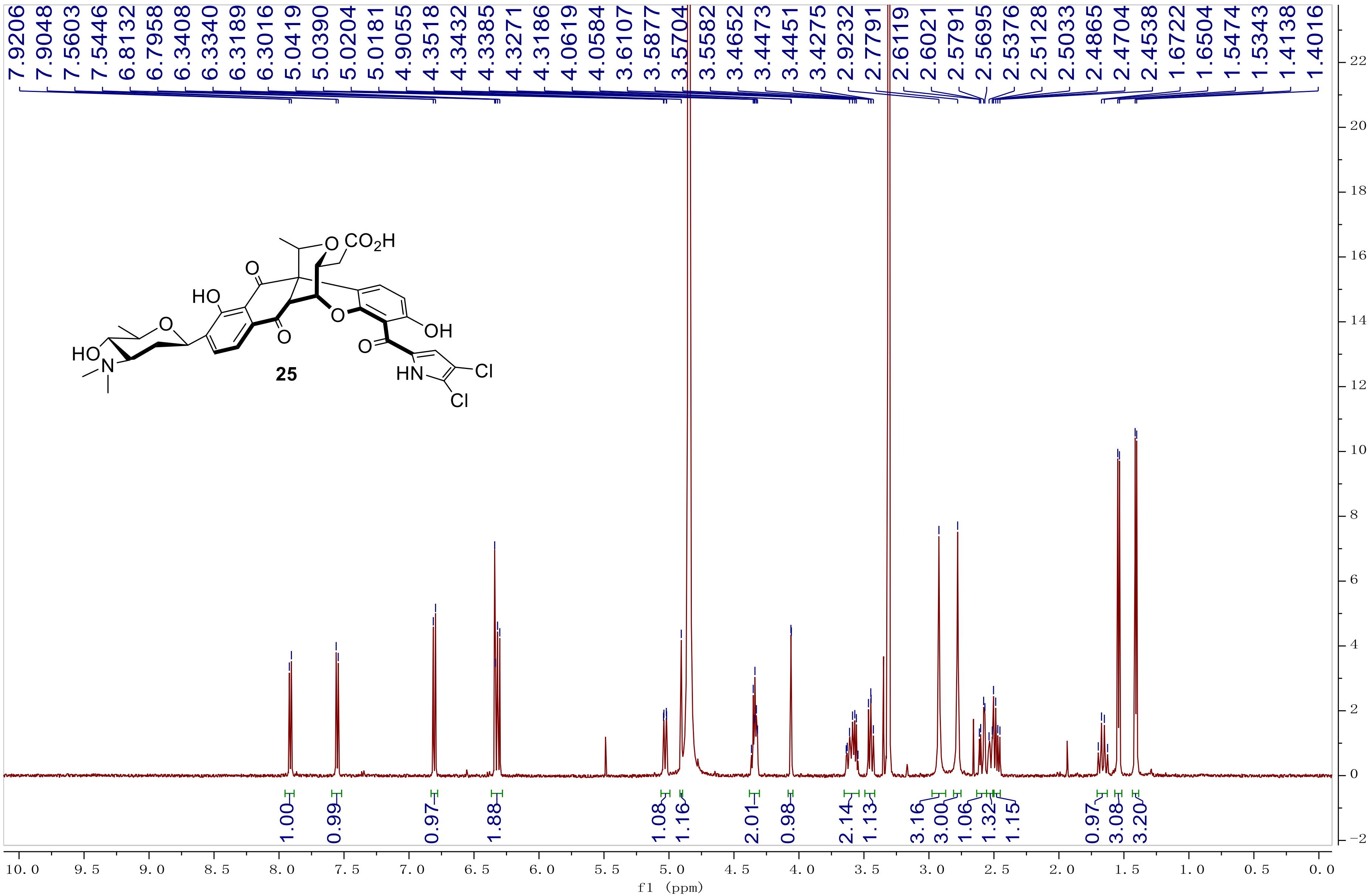

Supplementary Fig. 107. ^{13}C-NMR spectrum of chimedermycin N (**25**) in methanol-d_4

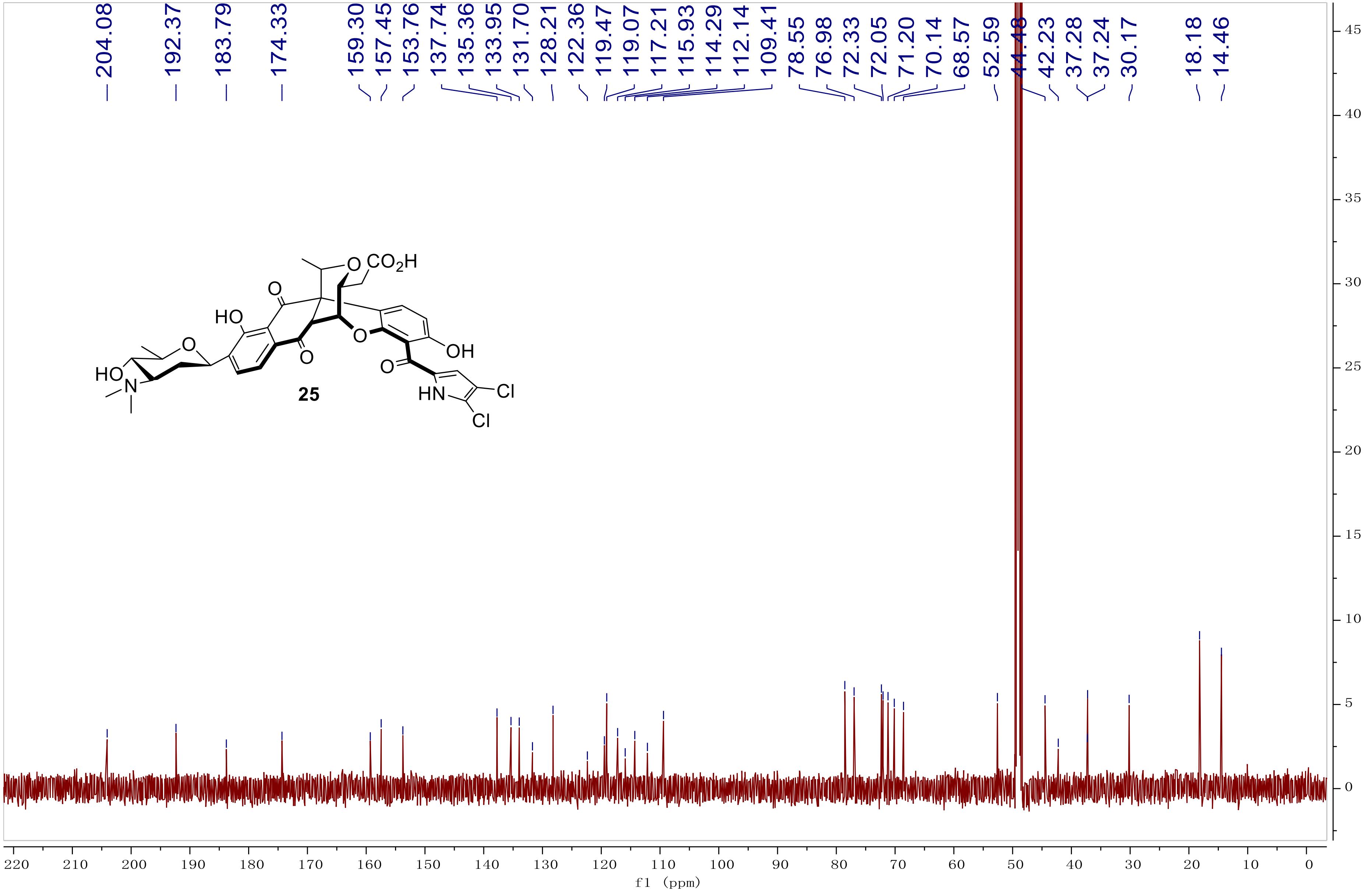

Supplementary Fig. 108. HSQC spectrum of chimedermycin N (**25**) in methanol-d_4

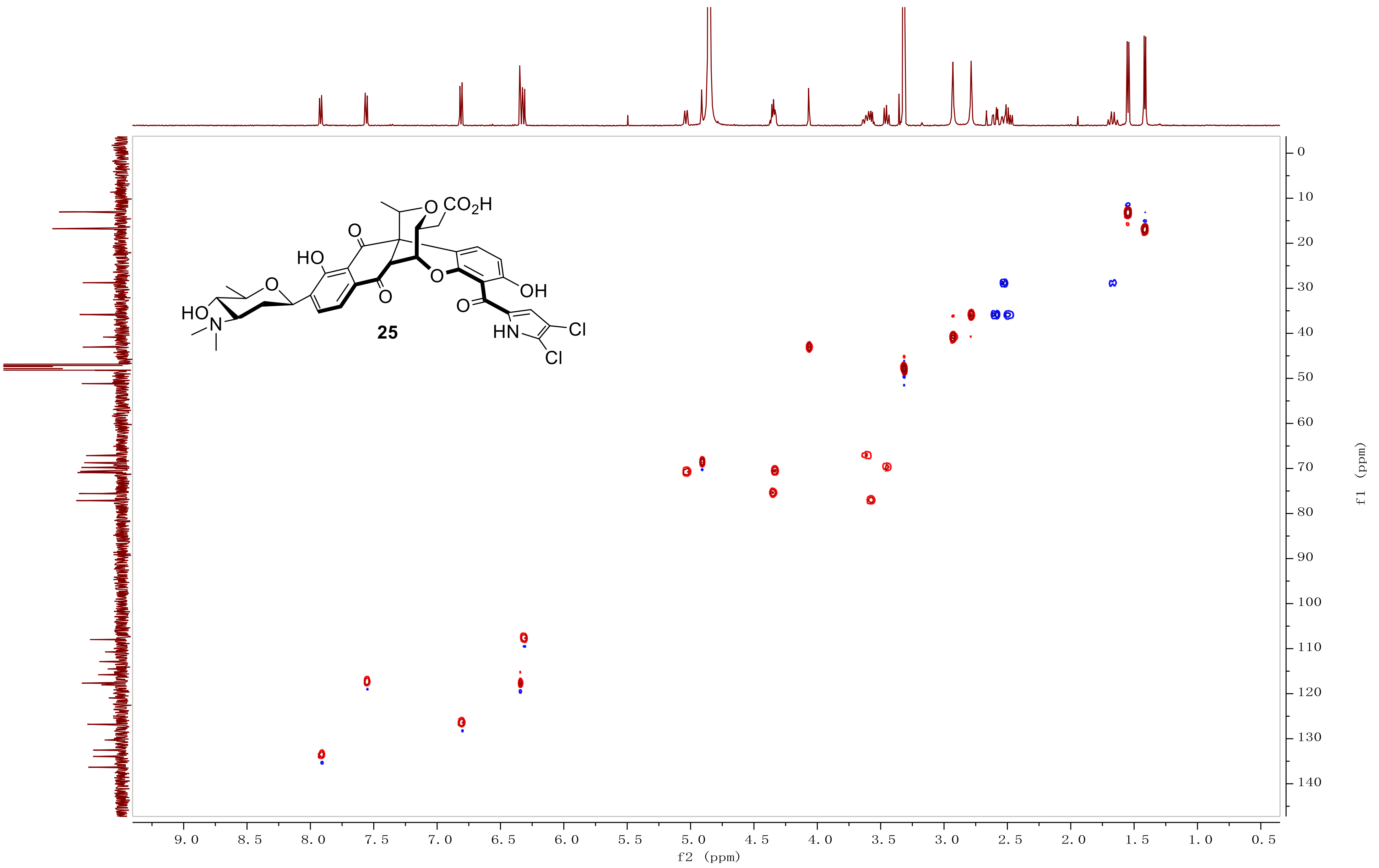

Supplementary Fig. 109. ^{1}H-^{1}H COSY spectrum of chimedermycin N (**25**) in methanol-d_4

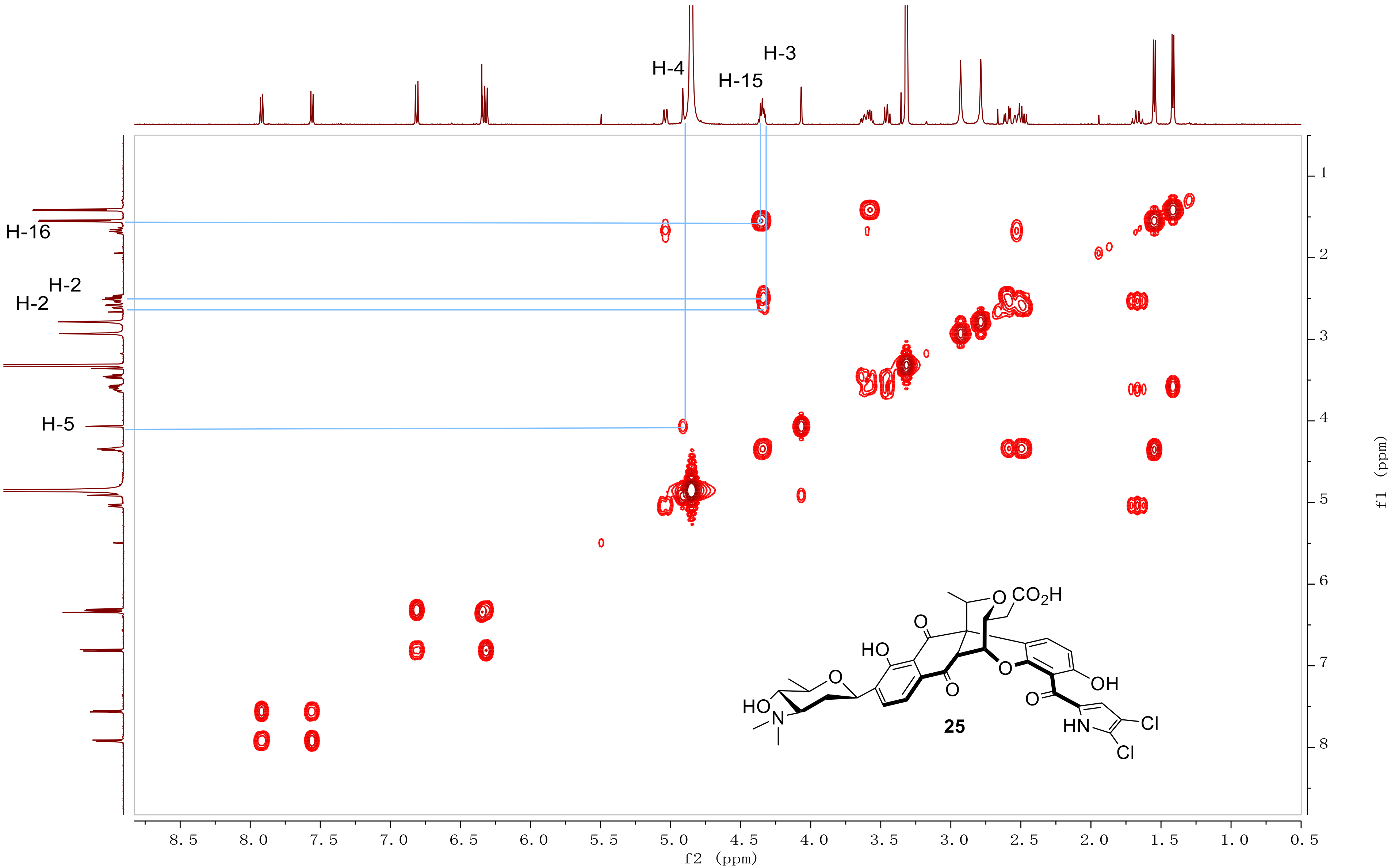

Supplementary Fig. 110. HMBC spectrum of chimedermycin N (**25**) in methanol-d_4

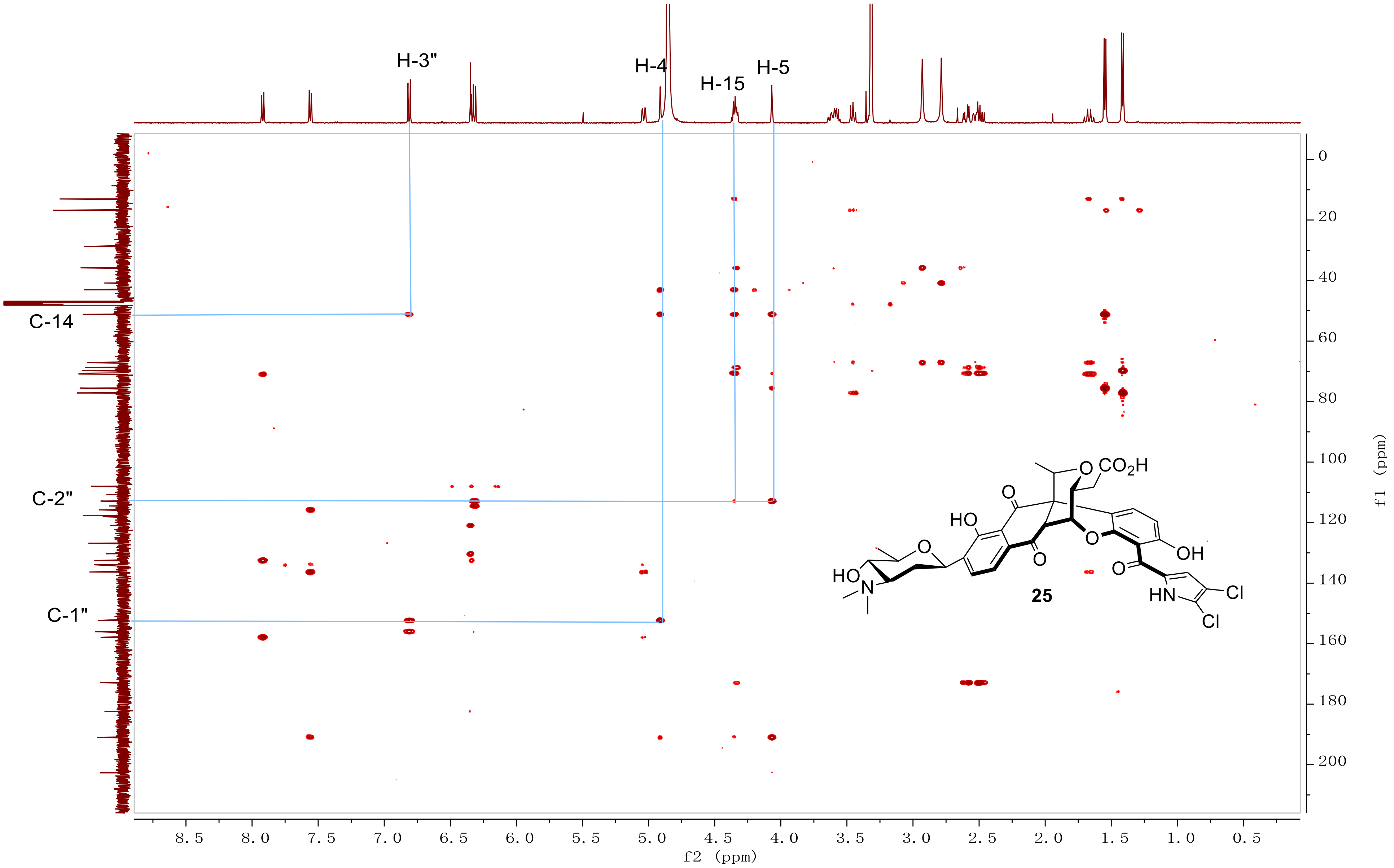

Supplementary Fig. 111. NOESY spectrum of chimedermycin N (**25**) in methanol-d_4

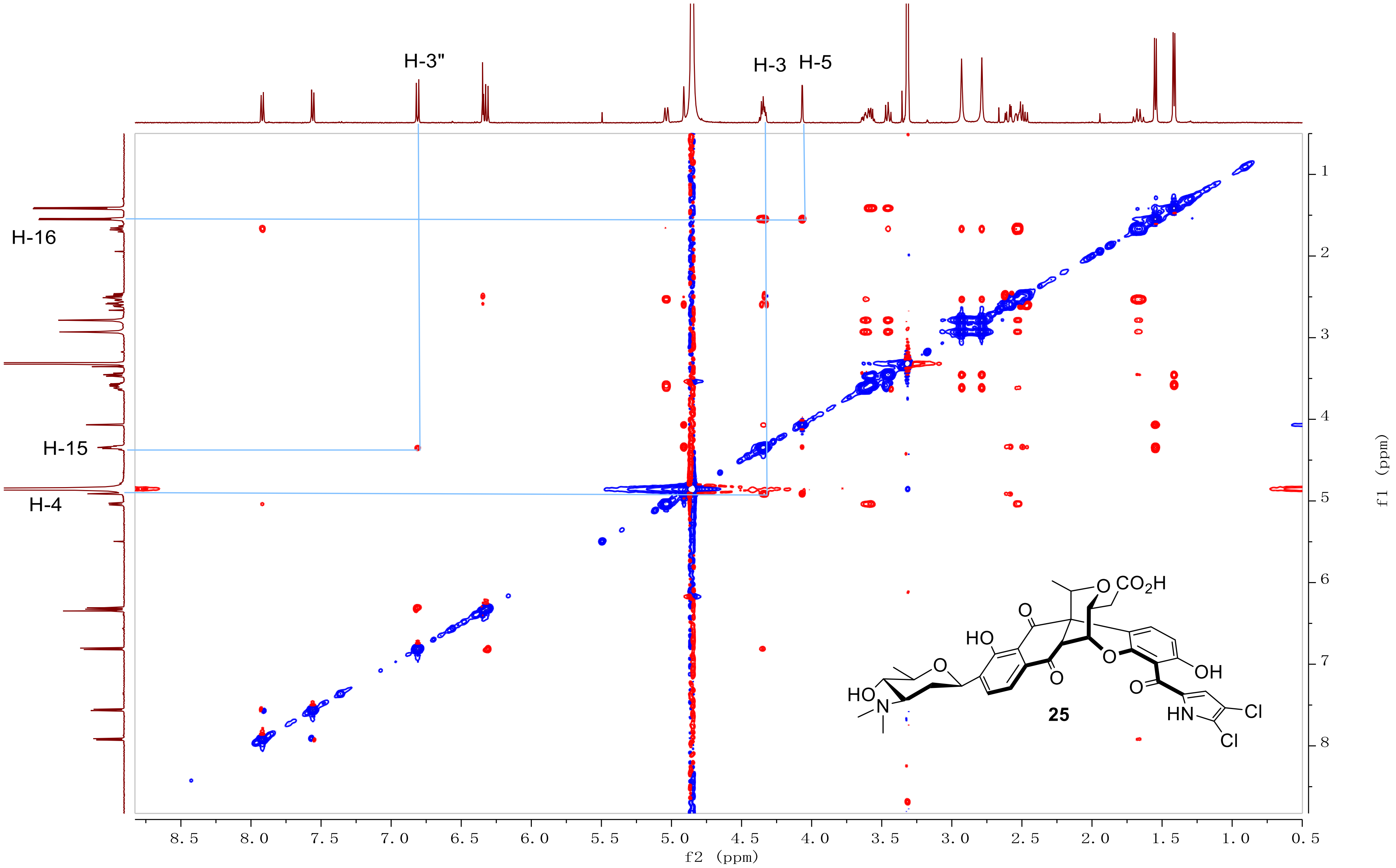

Supplementary Fig. 112. HRESIMS spectrum of sekgranaticin B (**27**)

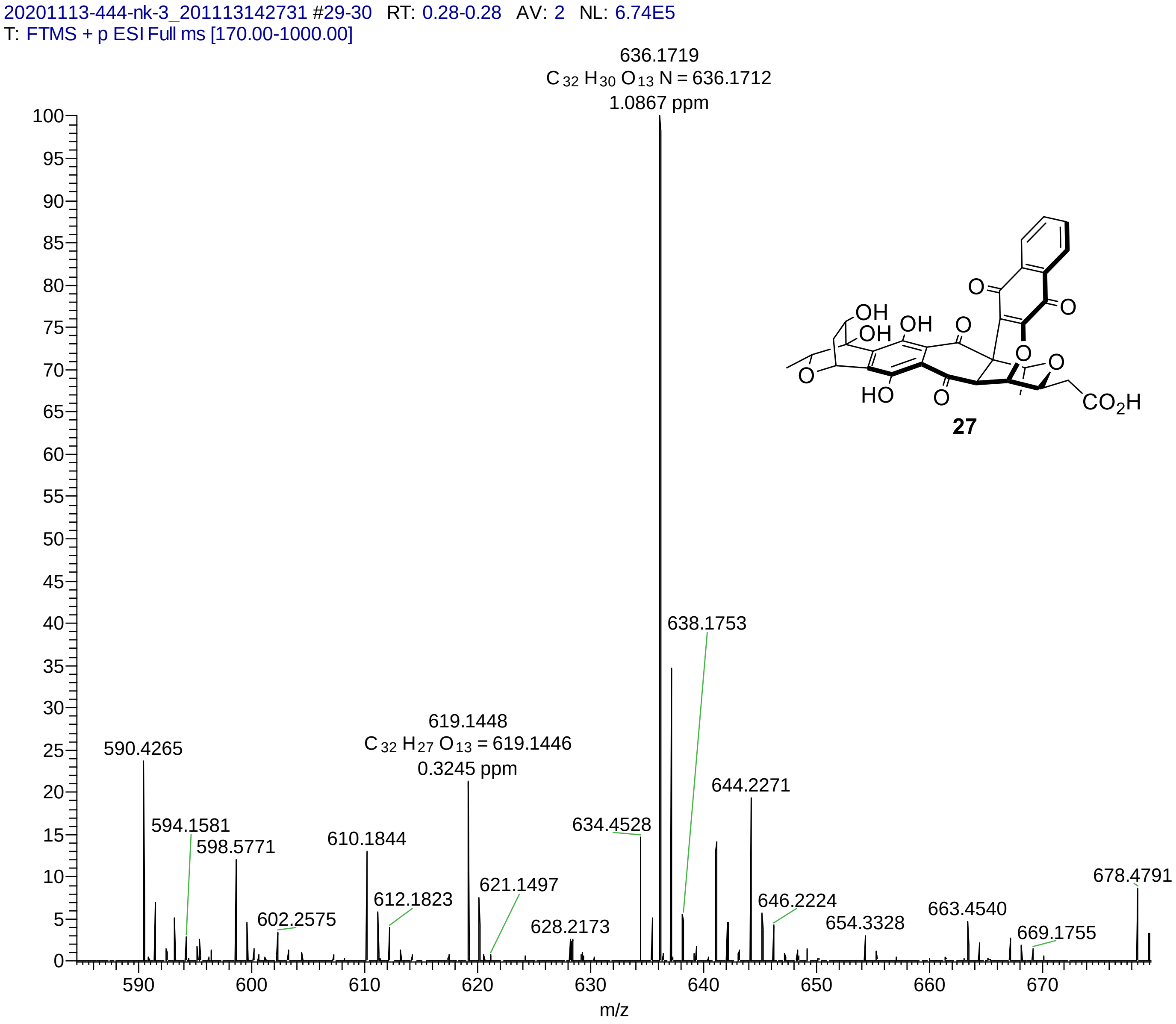

Supplementary Fig. 113. ^{1}H-NMR spectrum of sekgranaticin B (**27**) in DMSO-d_6

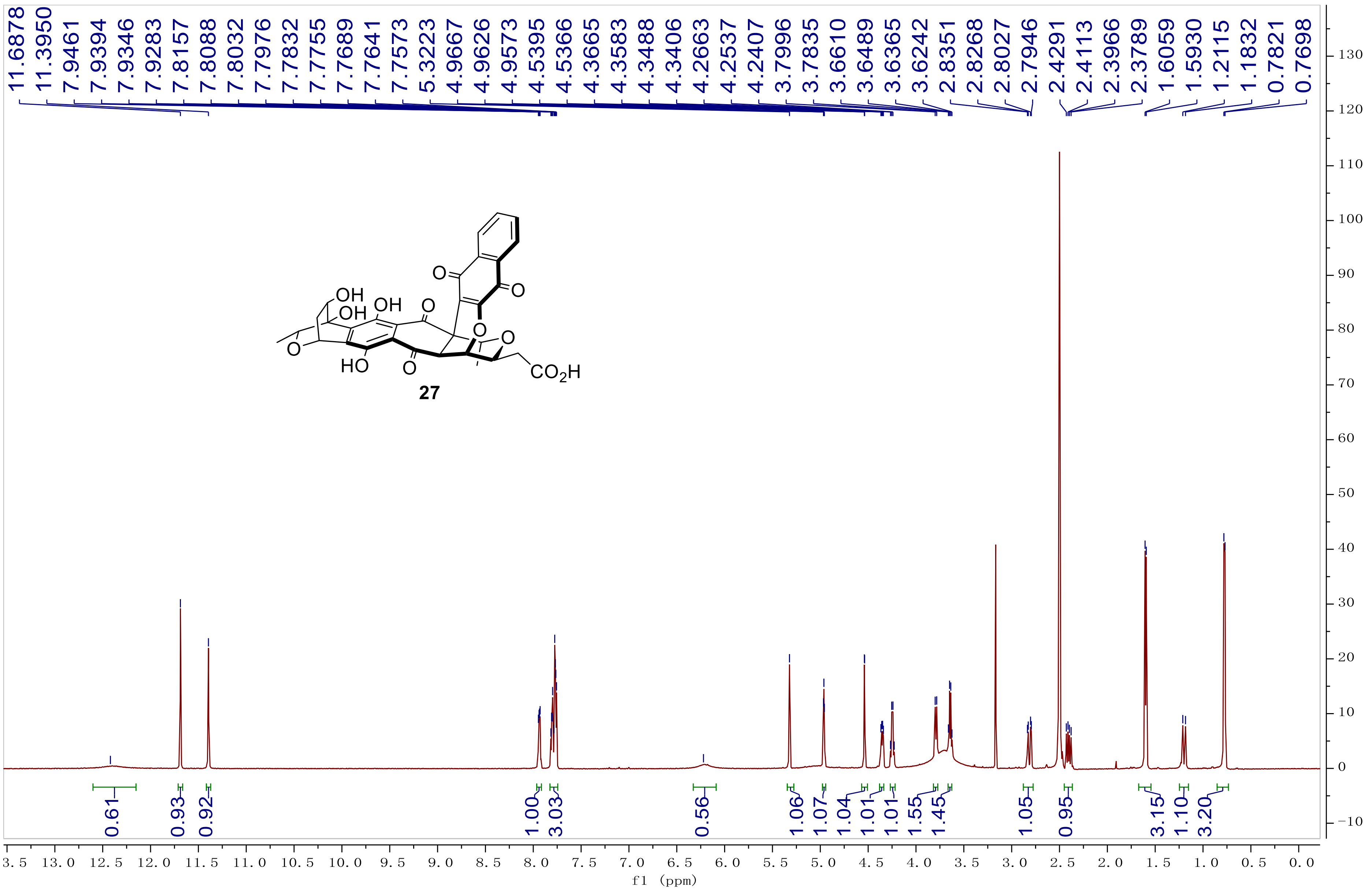

Supplementary Fig. 114. ^{13}C-NMR spectrum of sekgranaticin B (**27**) in DMSO-d_6

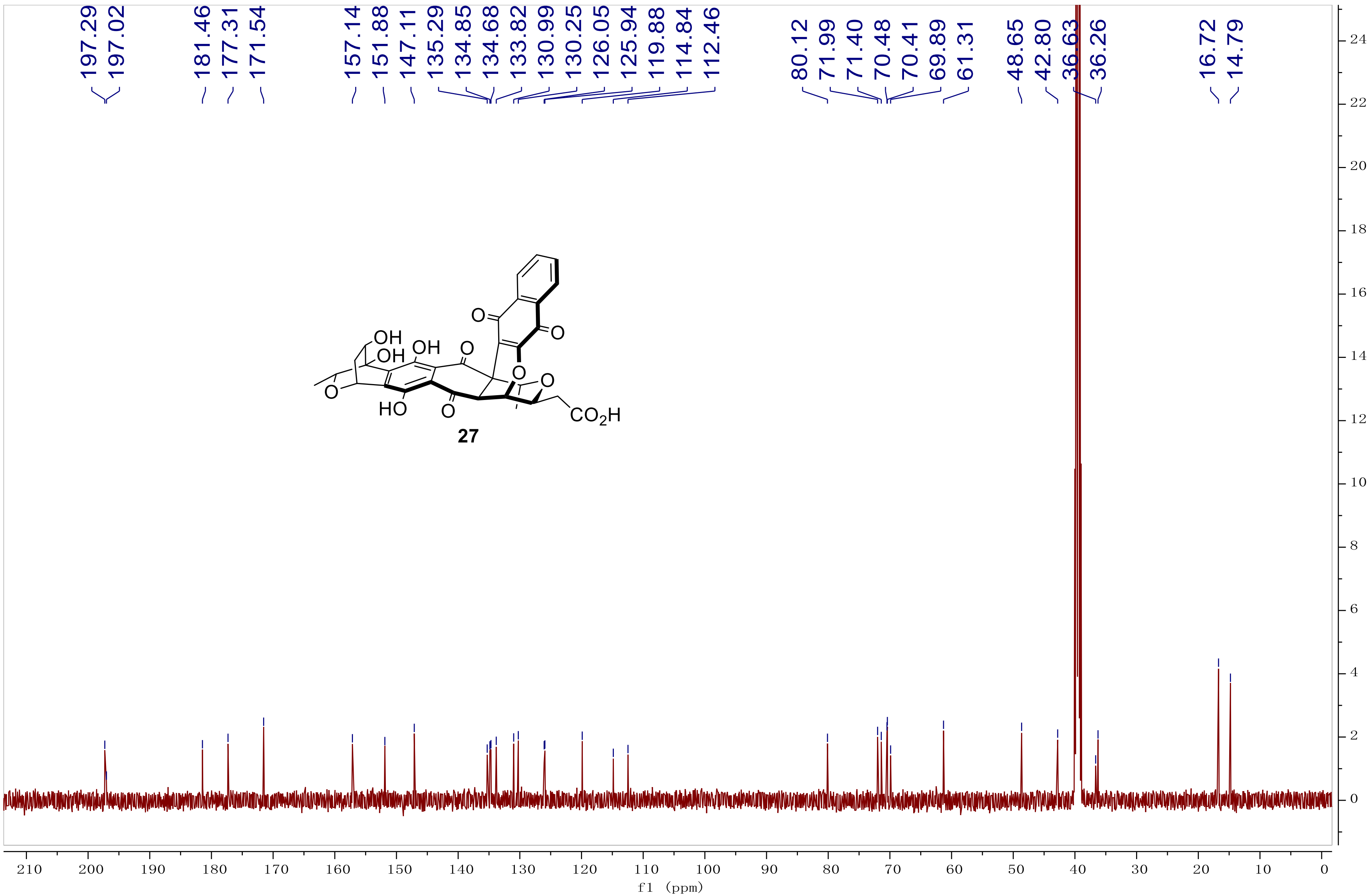

Supplementary Fig. 115. HSQC spectrum of sekgranaticin B (**27**) in DMSO-d_6

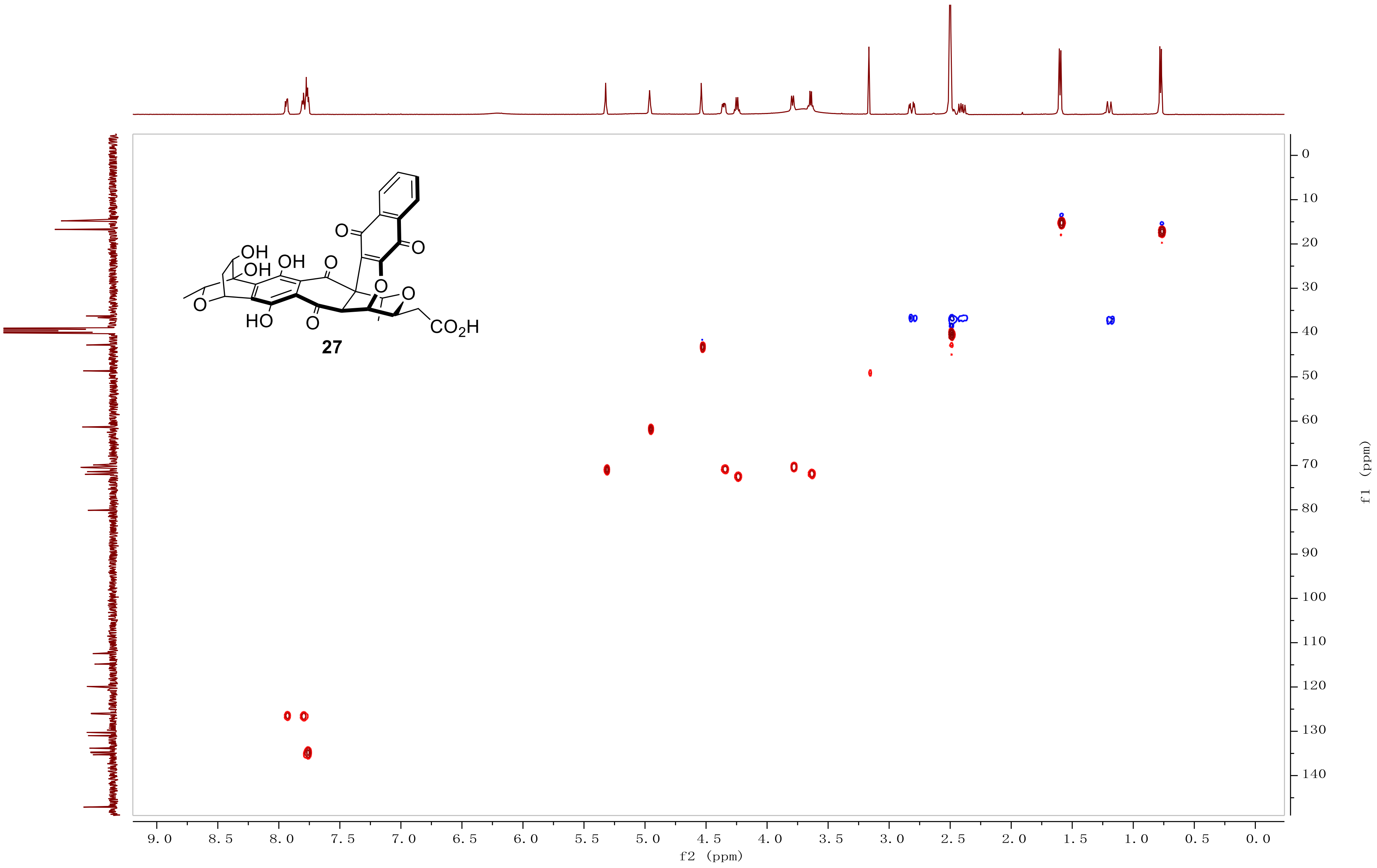

Supplementary Fig. 116. ^{1}H-^{1}H COSY spectrum of sekgranaticin B (**27**) in DMSO-d_6

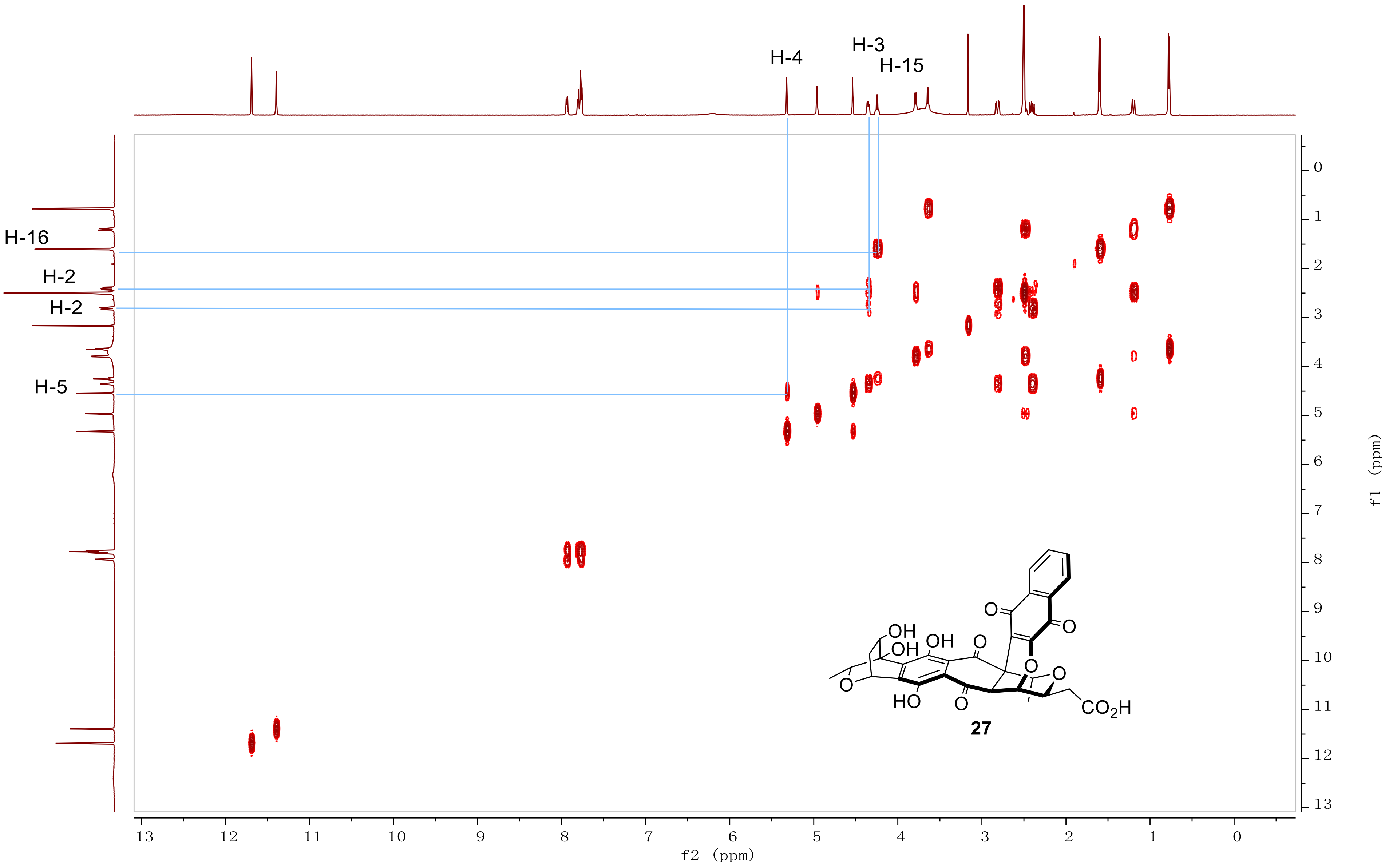

Supplementary Fig. 117. HMBC spectrum of sekgranaticin B (**27**) in DMSO-d_6

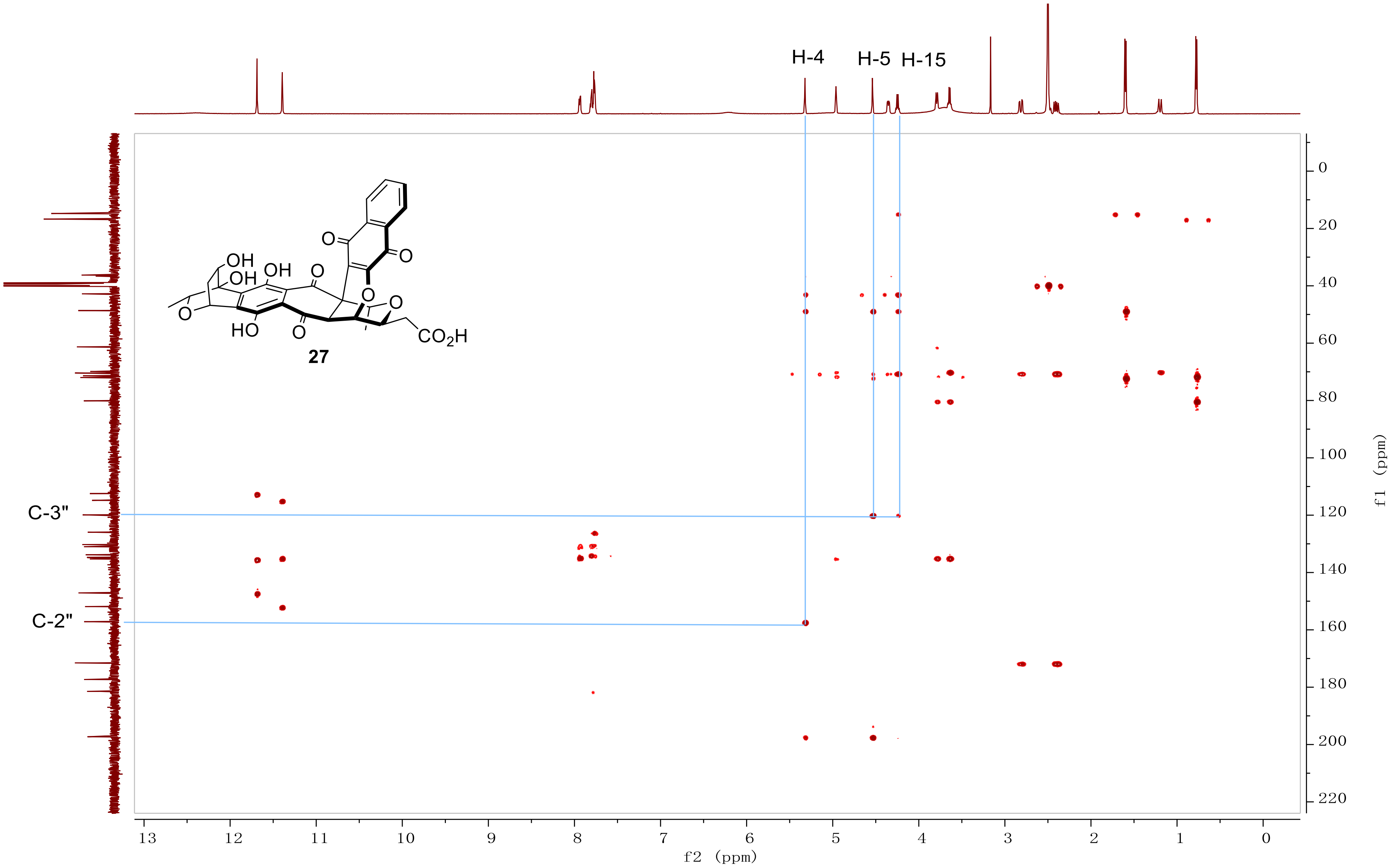

Supplementary Fig. 118. NOESY spectrum of sekgranaticin B (**27**) in DMSO-d_6

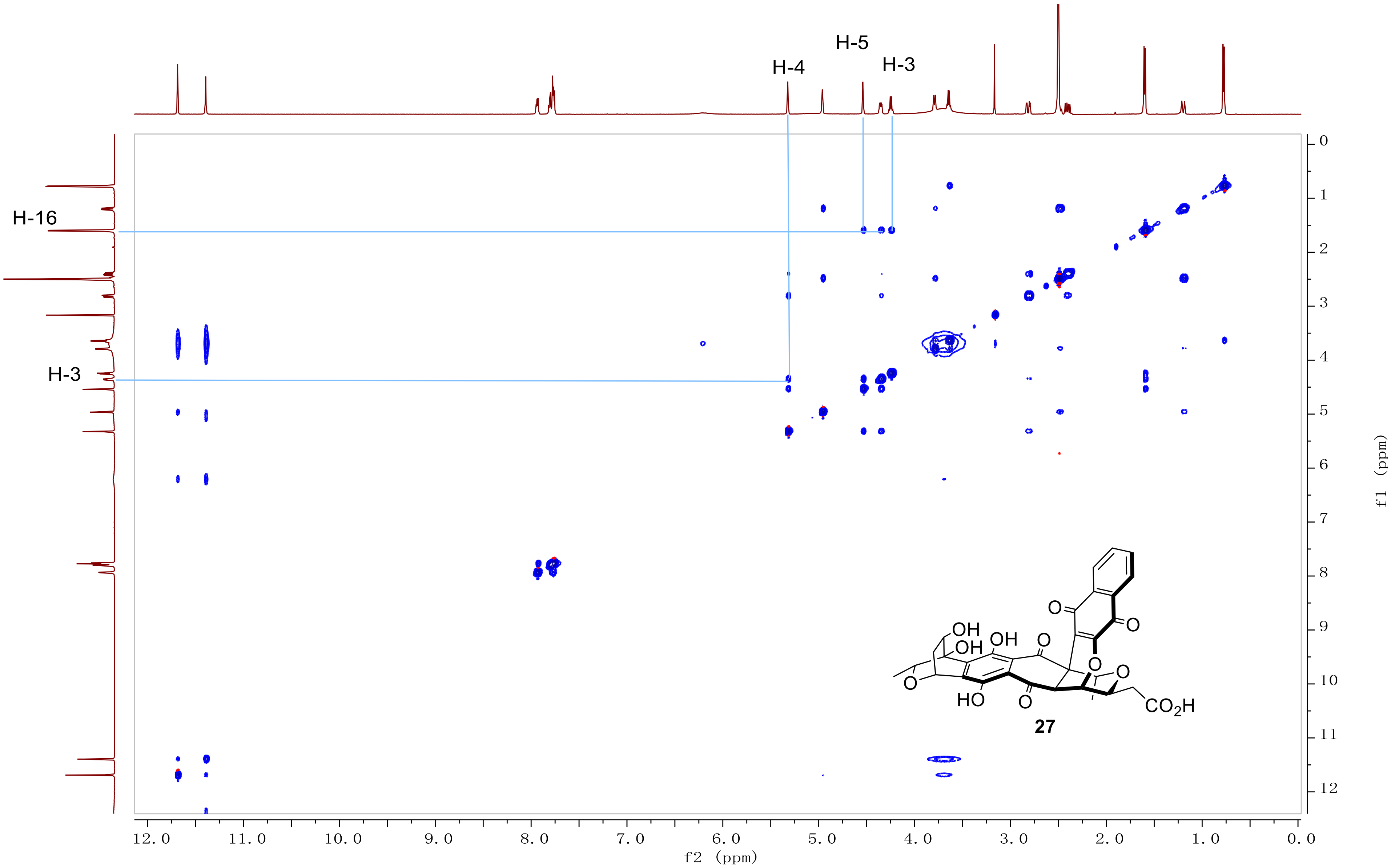

Supplementary Table 6. The calculated ^{13}C NMR data for isomers of chimedermycin A (**1**)

no.	δexp	δcal				δscal				corrected error			
		1A	1B	1C	1D	1A	1B	1C	1D	1A	1B	1C	1D
1	174.1	179.27	180.53	180.57	180.75	171.89	172.93	173.14	173.41	-2.21	-1.17	-0.96	-0.69
2	38.0	38.98	36.63	36.64	36.88	36.63	34.68	34.75	34.98	-1.37	-3.32	-3.25	-3.02
3	72.1	76.63	75.90	76.13	76.04	72.93	72.41	72.72	72.66	0.83	0.31	0.62	0.56
4	71.1	75.61	72.29	72.89	73.03	71.94	68.94	69.60	69.77	0.84	-2.16	-1.50	-1.33
5	43.8	48.87	47.34	47.34	47.72	46.17	44.97	45.04	45.41	2.37	1.17	1.24	1.61
6	192.2	199.95	199.91	199.19	199.30	191.82	191.55	191.03	191.26	-0.38	-0.65	-1.17	-0.94
7	133.7	136.70	137.69	137.02	136.81	130.84	131.77	131.26	131.13	-2.86	-1.93	-2.44	-2.57
8	119.2	123.01	123.15	123.30	123.15	117.65	117.80	118.07	117.99	-1.55	-1.40	-1.13	-1.21
9	135.1	140.83	140.09	140.34	140.56	134.83	134.07	134.46	134.74	-0.27	-1.03	-0.64	-0.36
10	137.9	147.95	147.83	147.98	147.80	141.69	141.51	141.80	141.71	3.79	3.61	3.90	3.81
11	159.2	167.19	167.57	167.02	167.23	160.23	160.47	160.10	160.40	1.03	1.27	0.90	1.20
12	117.3	121.36	120.98	121.02	121.21	116.05	115.71	115.88	116.12	-1.25	-1.59	-1.42	-1.18
13	204.1	213.83	214.61	214.37	214.35	205.20	205.67	205.63	205.74	1.10	1.57	1.53	1.64
14	52.8	58.81	57.32	56.46	57.49	55.74	54.56	53.81	54.81	2.94	1.76	1.01	2.01
15	76.1	81.86	81.64	80.88	81.16	77.97	77.92	77.29	77.59	1.87	1.82	1.19	1.49
16	14.6	14.65	14.32	14.76	14.56	13.17	13.24	13.71	13.50	-1.43	-1.36	-0.89	-1.10
1'	72.3	78.41	79.95	78.67	78.90	74.64	76.30	75.16	75.42	2.34	4.00	2.86	3.12
2'	30.1	30.30	31.61	31.26	30.89	28.26	29.85	29.58	29.22	-1.84	-0.25	-0.52	-0.88
3'	68.4	73.29	73.44	73.62	73.20	69.71	70.05	70.30	69.93	1.31	1.65	1.90	1.53
4'	71.2	75.45	75.71	76.71	76.04	71.79	72.23	73.28	72.66	0.59	1.03	2.08	1.46
5'	78.5	83.78	85.53	84.48	84.35	79.82	81.66	80.74	80.65	1.32	3.16	2.24	2.15
6'	18.2	19.96	19.79	20.40	20.10	18.29	18.50	19.14	18.83	0.09	0.30	0.94	0.63
7'	42.2	45.94	46.39	45.26	45.66	43.33	44.05	43.04	43.43	1.13	1.85	0.84	1.23
8'	37.3	36.89	36.88	36.51	37.24	34.61	34.91	34.63	35.33	-2.69	-2.39	-2.67	-1.97
1"	174.8	180.06	180.35	180.13	180.40	172.65	172.76	172.71	173.07	-2.15	-2.04	-2.09	-1.73
2"	41.9	42.46	42.64	43.17	42.93	39.98	40.45	41.03	40.80	-1.92	-1.45	-0.87	-1.10
3"	65.4	67.74	67.52	67.20	67.49	64.36	64.36	64.13	64.43	-1.04	-1.04	-1.27	-0.97
4"	34.8	38.77	38.29	38.32	37.78	36.42	36.28	36.37	35.85	1.62	1.48	1.57	1.05
5"	135.4	143.33	142.92	143.57	142.26	137.23	136.79	137.56	136.37	1.83	1.39	2.16	0.97
6"	118.8	123.21	120.87	123.17	122.35	117.83	115.61	117.94	117.22	-0.97	-3.19	-0.86	-1.58
7"	136.0	140.99	141.74	140.51	140.67	134.97	135.66	134.61	134.85	-1.03	-0.34	-1.39	-1.15
8"	122.1	125.11	125.64	125.91	125.82	119.67	120.19	120.58	120.55	-2.43	-1.91	-1.52	-1.55
9"	122.9	127.18	126.36	126.29	126.21	121.66	120.88	120.95	120.93	-1.24	-2.02	-1.95	-1.97
10"	114.9	121.95	120.87	120.82	119.80	116.62	115.61	115.69	114.76	1.72	0.71	0.79	-0.14
11"	151.9	159.13	159.52	158.80	158.51	152.47	152.74	152.20	152.01	0.57	0.84	0.30	0.11
12"	113.4	117.26	117.53	117.33	117.36	112.10	112.40	112.33	112.42	-1.30	-1.00	-1.07	-0.98
13"	150.5	159.23	158.74	158.80	159.00	152.56	151.99	152.20	152.49	2.06	1.49	1.70	1.99
14"	123.1	128.85	129.31	128.99	128.76	123.27	123.72	123.54	123.39	0.17	0.62	0.44	0.29
15"	70.1	74.46	75.12	74.58	74.89	70.84	71.65	71.23	71.55	0.74	1.55	1.13	1.45
16"	19.2	18.49	18.95	18.69	18.69	16.87	17.69	17.50	17.48	-2.33	-1.51	-1.70	-1.72

Supplementary Table 7. DFT-optimized structures and thermodynamic parameters for low-energy conformers of **1A**

Conformers	Conf. A	Conf. B	Conf. C
DFT-optimized structures	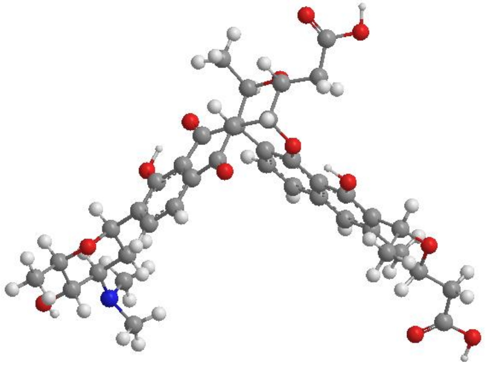	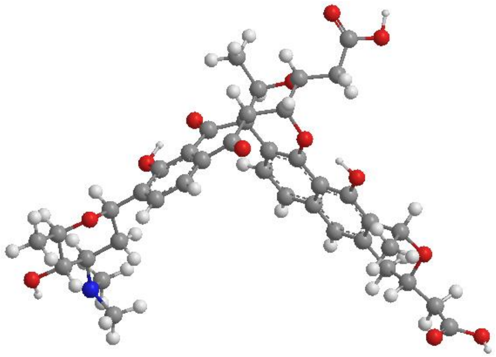	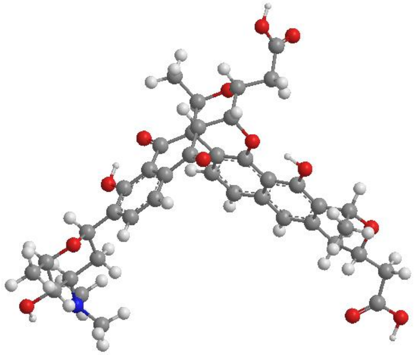
Population	55.95%	35.98%	8.07%
Total energy (a.u.)	-2582.84785597	-2582.84743971	-2582.84603062
Sum of electronic and zero-point energies (a.u.)	-2582.056219	-2582.055822	-2582.054401
Sum of electronic and thermal energies (a.u.)	-2582.007931	-2582.007495	-2582.006119
Sum of electronic and thermal enthalpies (a.u.)	-2582.006987	-2582.006551	-2582.005175
Sum of electronic and thermal free energies (a.u.)	-2582.140794	-2582.140593	-2582.138803

Supplementary Table 8. DFT-optimized structures and thermodynamic parameters for low-energy conformers of **1B**

Conformers	Conf. A	Conf. B	Conf. C
DFT-optimized structures	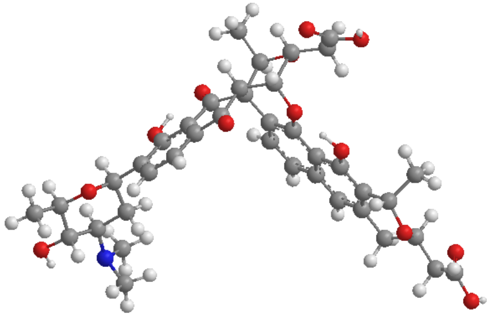	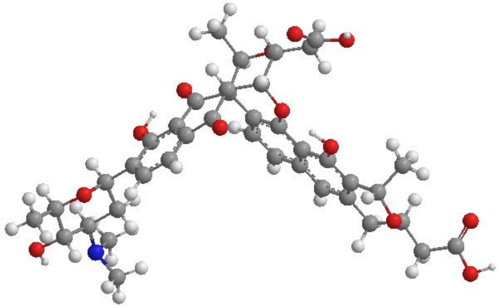	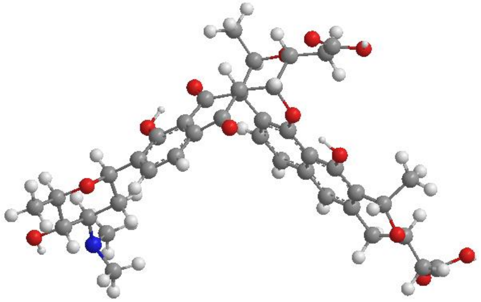
Population	54.86%	36.70%	8.44%
Total energy (a.u.)	-2582.84911807	-2582.84873921	-2582.84735268
Sum of electronic and zero-point energies (a.u.)	-2582.057556	-2582.057017	-2582.055624
Sum of electronic and thermal energies (a.u.)	-2582.009312	-2582.008767	-2582.007340
Sum of electronic and thermal enthalpies (a.u.)	-2582.008368	-2582.007823	-2582.006396
Sum of electronic and thermal free energies (a.u.)	-2582.142292	-2582.141922	-2582.141199

Supplementary Table 9. DFT-optimized structures and thermodynamic parameters for low-energy conformers of **1C**

Conformers	Conf. A	Conf. B	Conf. C	Conf. D
DFT-optimized structures	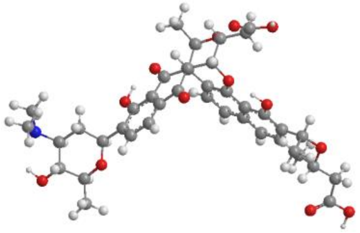	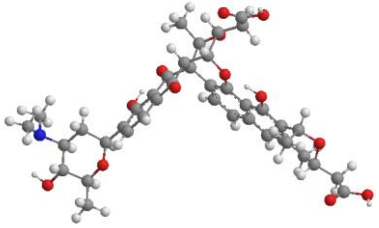	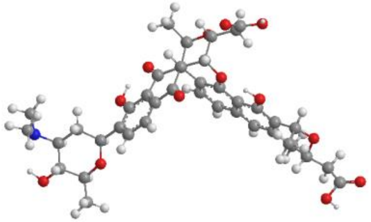	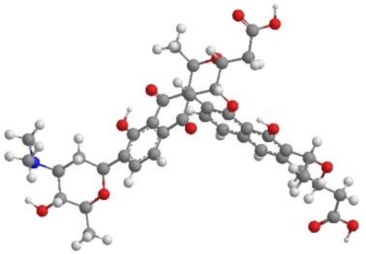
Population	47.73%	29.87%	11.47%	10.93%
Total energy (a.u.)	-2582.84901843	-2582.84857649	-2582.84767373	-2582.84762850
Sum of electronic and zero-point energies (a.u.)	-2582.057391	-2582.056942	-2582.055998	-2582.055986
Sum of electronic and thermal energies (a.u.)	-2582.009124	-2582.008632	-2582.007721	-2582.007735
Sum of electronic and thermal enthalpies (a.u.)	-2582.008180	-2582.007688	-2582.006777	-2582.006791
Sum of electronic and thermal free energies (a.u.)	-2582.142125	-2582.141898	-2582.140577	-2582.140632

Supplementary Table 10. DFT-optimized structures and thermodynamic parameters for low-energy conformers of **1D**

Conformers	Conf. A	Conf. B	Conf. C	Conf. D
DFT-optimized structures	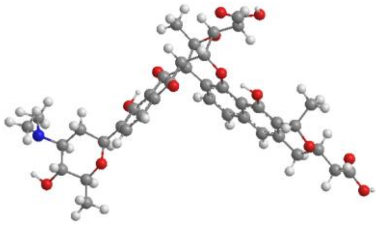	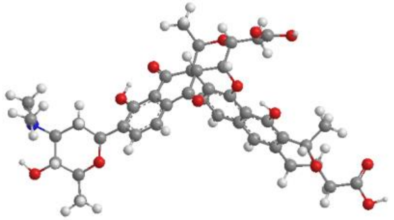	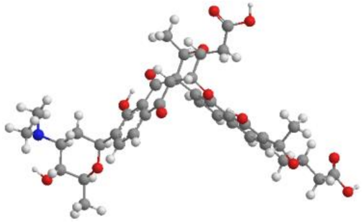	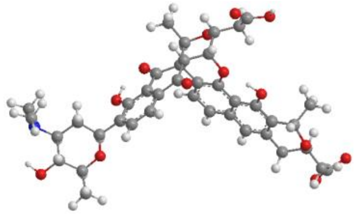
Population	49.06%	32.55%	11.06%	7.33%
Total energy (a.u.)	-2582.84901673	-2582.84862999	-2582.84761214	-2582.84722371
Sum of electronic and zero-point energies (a.u.)	-2582.057337	-2582.056980	-2582.056070	-2582.055330
Sum of electronic and thermal energies (a.u.)	-2582.009105	-2582.008696	-2582.007785	-2582.007069
Sum of electronic and thermal enthalpies (a.u.)	-2582.008161	-2582.007752	-2582.006841	-2582.006125
Sum of electronic and thermal free energies (a.u.)	-2582.142098	-2582.141693	-2582.141010	-2582.140639

Supplementary References

1. Wang, M. et al. Sharing and community curation of mass spectrometry data with global natural products social molecular networking. *Nat. Biotechnol.* **34**, 828−837 (2016).
2. Tatsuta, K., Ozeki, H., Yamaguchi, M., Tanaka, M. & Okui, T. Enantioseletive total synthesis of medermycin (lactoquinomycin). *Tetrahedron Lett.* **31**, 5495−5498 (1990).
3. Takano, S. et al. A new antibiotic, medermycin. *J. Antibiot.* **29**, 765−768 (1976).
4. Tanaka, N. et al. Lactoquinomycin, a novel anticancer antibiotic. i. taxonomy, isolation and biological activity. *J. Antibiot.* **38**, 1327−1332 (1985).
5. Okabe, T. et al. Lactoquinomycin, a novel anticancer antibiotic. ii. physico-chemical properties and structure assignment. *J. Antibiot.* **38**, 1333−1336 (1985).
6. Zhang, Q. & O'Doherty, G. A. *De novo* asymmetric synthesis of (–)-nanaomycin A. *Tetrahedron* **74**, 4994−4999 (2018).
7. Hashimoto, M. et al. Unveiling two consecutive hydroxylations: mechanisms of aromatic hydroxylations catalyzed by flavin-dependent monooxygenases for the biosynthesis of actinorhodin and related antibiotics. *ChemBioChem* **21**, 623−627 (2020).
8. Fu, P. et al. Streptocarbazoles A and B, two novel indolocarbazoles from the marine-derived actinomycete strain *Streptomyces* sp. FMA. *Org. Lett.* **14**, 2422−2425 (2012).
9. Fu, P., Kong, F., Li, X., Wang, Y. & Zhu, W. Cyanogramide with a new spiro[indolinone-pyrroloimidazole] skeleton from *Actinoalloteichus cyanogriseus*. *Org. Lett.* **16**, 3708−3711 (2014).
10. Grimblat, N., Zanardi, M. M. & Sarotti, A. M. Beyond DP4: an improved probability for the stereochemical assignment of isomeric compounds using quantum chemical calculations of nmr shifts. *J. Org. Chem.* **80**, 12526−12534 (2015).
11. Jiang, Y.-J. et al. Medermycin-type naphthoquinones from the marine-derived *Streptomyces* sp. XMA39. *J. Nat. Prod.* **81**, 2120−2124 (2018).
12. Lacret, R. et al. MDN-0171, a new medermycin analogue from *Streptomyces albolongus* CA-186053. *Nat. Prod. Res.* **33**, 66−73 (2019).
13. Huang, Y. et al. A new medermycin analog from the marine-derived actinomycetes *Streptomyces* sp. ZS-A45. *J. Asian Nat. Prod. Res.* **21**, 826−831 (2019).
14. Zhou, B. et al. Purmedermycins A and B, two novel medermycin derivatives from *Streptomyces* sp. SS17A. *Org. Chem. Front.* **6**, 399−404 (2019).
15. Stephens, P. J., Pan, J.-J., Devlin, F. J., Urbanová, M. & Hájíček, J. Determination of the absolute configurations of natural products via density functional theory calculations of vibrational circular dichroism, electronic circular dichroism and optical rotation: the schizozygane alkaloid schizozygine. *J. Org. Chem.* **72**, 2508−2524 (2007).
16. Nakanishi, K., Berova, N. & Woody, R. W. Circular Dichroism: Principles and Application, 2nd ed. (Wiley-VCH, 2000).
17. Wu, C., Ichinose, K., Choi, Y. H. & van Wezel, G. P. Aromatic polyketide GTRI-02 is a previously unidentified product of the *act* gene cluster in *Streptomyces coelicolor* A3(2). *ChemBioChem* **18**, 1428−1434 (2017).
18. Cole, S. P., Rudd, B. A. M., Hopwood, D. A., Chang, C.-J. & Floss, H. G. Biosynthesis of the antibiotic actinorhodin analysis of blocked mutants of *Streptomyces coelicolor*. *J. Antibiot.* **29**, 340−347 (1987).
19. Taguchi, T., Ebizuka, Y., Hopwood, D. A. & Ichinose, K. A new mode of stereochemical control revealed by analysis of the biosynthesis of dihydrogranaticin in *Streptomyces violaceoruber* Tü22. *J. Am. Chem. Soc.* **123**, 11376−11380 (2001).
20. Santiago, C., Rubio, I., Sotomayor, N. & Lete, E. Selective Pd^{II}-catalyzed acylation of pyrrole with aldehydes. Application to the synthesis of celastramycin analogues and tolmetin. *Eur. J. Org. Chem.* **2020**, 4284−4295 (2020).
21. Rao, K. V. & Reddy, G. C. Synthesis and herbicidal activity of the halo analogues of pyoluteorin. *J. Agric. Food Chem.* **38**, 1260−1263 (1990).
22. Lv, Q. et al. Sekgranaticin, a SEK34b-granaticin hybrid polyketide from *Streptomyces* sp.166#. *J. Org. Chem.* **84**, 9087−9092 (2019).
23. Gaussian 09, Revision B.01, M. J. Frisch, G. W. Trucks, H. B. Schlegel, G. E. Scuseria, M. A. Robb, J. R. Cheeseman, G. Scalmani, V. Barone, B. Mennucci, G. A. Petersson, H. Nakatsuji, M. Caricato, X. Li, H. P. Hratchian, A. F. Izmaylov, J. Bloino, G. Zheng, J. L. Sonnenberg, M. Hada, M. Ehara, K. Toyota, R. Fukuda, J. Hasegawa, M. Ishida, T. Nakajima, Y. Honda, O. Kitao, H. Nakai, T. Vreven, J. A. Montgomery, Jr., J. E. Peralta, F. Ogliaro, M. Bearpark, J. J. Heyd, E. Brothers, K. N. Kudin, V. N. Staroverov, T. Keith, R. Kobayashi, J. Normand, K. Raghavachari, A. Rendell, J. C. Burant, S. S. Iyengar, J. Tomasi, M. Cossi, N. Rega, J. M. Millam, M. Klene, J. E. Knox, J. B. Cross, V. Bakken, C. Adamo, J. Jaramillo, R. Gomperts, R. E. Stratmann, O. Yazyev, A. J. Austin, R. Cammi, C. Pomelli, J. W. Ochterski, R. L. Martin, K. Morokuma, V. G. Zakrzewski, G. A. Voth, P. Salvador, J. J. Dannenberg, S. Dapprich, A. D. Daniels, O. Farkas, J. B. Foresman, J. V. Ortiz, J. Cioslowski, and D. J. Fox, Gaussian, Inc., Wallingford CT, 2010.
24. Spartan'14, Wavefunction Inc., Irvine CA, 2013.
25. Miertus, S. & Tomasi, J. Approximate evaluations of the electrostatic free energy and internal changes in solution processes. *Chem. Phys.* **65**, 239−245 (1982).

26. Tomasi, J. & Persico, M. Molecular interactions in solution: an overview of methods based on continuous distributions of the solvent. *Chem. Rev.* **94**, 2027−2094 (1994).
27. Cammi, R. & Tomasi, J. Remarks on the use of the apparent surface charges (ASC) methods in solvation problems: iterative versus matrix-inversion procedures and the renormalization of the apparent charges. *J. Comp. Chem.* **16**, 1449−1458 (1995).
28. Casida, M. E. In Recent Advances in Density Functional Methods, part I; Chong, D. P., Eds.; World Scientific: Singapore, 1995; pp 155−192.
29. Gross, E. K. U., Dobson, J. F. & Petersilka, M. Density functional theory of time-dependent phenomena. *Top. Curr. Chem.* **181**, 81−172 (1996).
30. Gross, E. K. U. & Kohn, W. Time-dependent density-functional theory. *Adv. Quantum Chem.* **21**, 255−291 (1990).
31. Runge, E. & Gross, E. K. U. Density-functional theory for time-dependent systems. *Phys. Rev. Lett.* **52**, 997−1000 (1984).
32. T. Bruhn, A. Schaumlöffel, Y. Hemberger. G. Pescitelli, SpecDis, Version 1.70.1, Berlin, Germany, 2017, https://specdis-software.jimdo.com.
