# Supplementary material for: Chimeric natural products derived from medermycin and the nature-inspired construction of their polycyclic skeletons
Source: Nat Commun. 2022 Sep 2;13:5169. doi: 10.1038/s41467-022-32901-0 (PMC9440243; doi:10.1038/s41467-022-32901-0)
Supplement: Supplementary file 3 — Description of Additional Supplementary Files [file 41467_2022_32901_MOESM3_ESM.pdf]

Description of Supplementary Data files

File name: Supplementary Data 1
Description: Optimized Z-matrixes of **1A** in the gas phase (Å) at B3LYP/6-31G(d) level

File name: Supplementary Data 2
Description: Optimized Z-matrixes of **1B** in the gas phase (Å) at B3LYP/6-31G(d) level

File name: Supplementary Data 3
Description: Optimized Z-matrixes of **1C** in the gas phase (Å) at B3LYP/6-31G(d) level

File name: Supplementary Data 4
Description: Optimized Z-matrixes of **1D** in the gas phase (Å) at B3LYP/6-31G(d) level
